# Kinetic Resolution of 2‐Aryldihydroquinolines Using Lithiation – Synthesis of Chiral 1,2‐ and 1,4‐Dihydroquinolines

**DOI:** 10.1002/chem.202300815

**Published:** 2023-05-05

**Authors:** Song‐Hee Yeo, Anthony Choi, Sophie Greaves, Anthony J. H. M. Meijer, Ilaria Proietti Silvestri, Iain Coldham

**Affiliations:** ^1^ Department of Chemistry University of Sheffield Brook Hill Sheffield S3 7HF UK; ^2^ Liverpool ChiroChem The Heath Business & Technical Park Runcorn Cheshire WA7 4QX UK

**Keywords:** dihydroquinolines, enantioselectivity, kinetic resolution, lithiation, nitrogen heterocycles

## Abstract

Highly enantiomerically enriched dihydrohydroquinolines were prepared in two steps from quinoline. Addition of aryllithiums to quinoline with *tert*‐butoxycarbonyl (Boc) protection gave *N*‐Boc‐2‐aryl‐1,2‐dihydroquinolines. These were treated with *n*‐butyllithium and electrophilic trapping occurred exclusively at C‐4 of the dihydroquinoline, a result supported by DFT studies. Variable temperature NMR spectroscopy gave kinetic data for the barrier to rotation of the carbonyl group (ΔG^≠^≈49 kJ mol^−1^, 195 K). Lithiation using the diamine sparteine allowed kinetic resolutions with high enantioselectivities (enantiomer ratio up to 99 : 1). The enantioenriched 1,2‐dihydroquinolines could be converted to 1,4‐dihydroquinolines with retention of stereochemistry. Further functionalisation led to trisubstituted products. Reduction provided enantioenriched tetrahydroquinolines, whereas acid‐promoted removal of Boc led to quinolines, and this was applied to a synthesis of the antimalarial compound M5717.

## Graphical abstract

The asymmetric synthesis of substituted dihydroquinolines was achieved using a kinetic resolution of 1,2‐dihydroquinolines with *n*‐BuLi and the chiral ligand sparteine. Subsequent lithiation at C‐2 and trapping with an electrophile occurs at C‐4 with retention of stereochemistry to provide an unusual route to enantiomerically enriched 1,4‐dihydroquinolines.

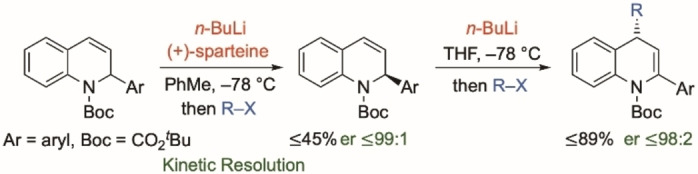

## Introduction

Tetrahydroquinolines and quinolones are one of the most common ring systems in natural products and medicinal drugs.^[1]^ Therefore, it is not surprising that there has been considerable interest in their asymmetric synthesis.^[2]^ In contrast, the related dihydroquinoline ring system has received relatively little attention. This is despite their potential as drugs or prodrugs,[^3^, ^4^, ^5^, ^6^] and their usefulness as intermediates towards the synthesis of quinolines or tetrahydroquinolines.

**Scheme 1:**
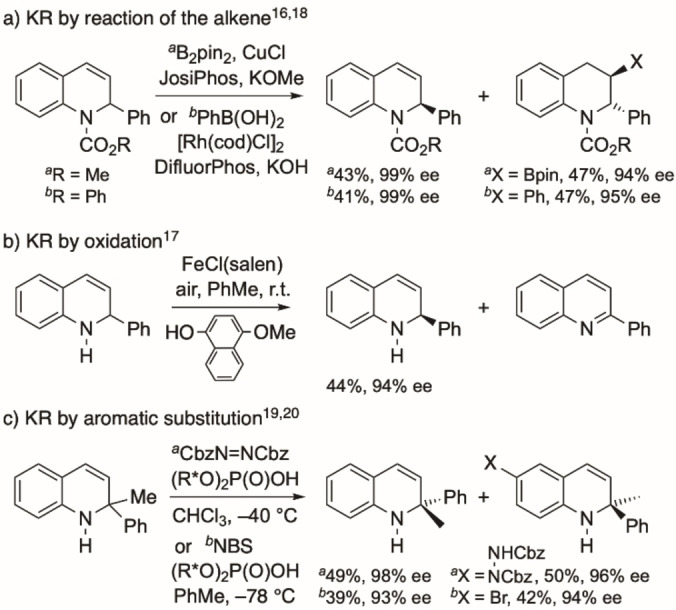
Previous examples of kinetic resolution (KR) of dihydroquinolines.

There are only a few examples of the asymmetric synthesis of 1,2‐dihydroquinolines and these include the addition of a nucleophile (such as a vinylboronic acid) to C‐2 of a quinolinium salt in the presence of a chiral ligand,[^7^, ^8^, ^9^, ^10^, ^11^, ^12^] or conjugate addition of an aniline followed by intramolecular aldol reaction.^[13]^ Direct addition of an organometallic species to quinoline generally gives moderate enantioselectivity with sparteine as a chiral ligand,^[14]^ although an asymmetric Heck reaction with a 1,4‐dihydroquinoline has been successful.^[15]^ Recently, the synthesis of 1,2‐dihydroquinolines has been reported using kinetic resolution.[^16^, ^17^, ^18^, ^19^, ^20^] This strategy has involved the preferential reaction of one enantiomer of the 2‐substituted 1,2‐dihydroquinoline by reaction of the 3,4‐alkene using a borylation, oxidation, or arylation process (Scheme 1A,B),[^16^, ^17^, ^18^] or by electrophilic aromatic substitution (Scheme 1C).[^19^, ^20^] These methods have their limitations, for example in the lack of access to 4‐substituted derivatives.

Kinetic resolution is a powerful method for the synthesis of enantiomerically enriched compounds.[^21^, ^22^] Initial attempts to apply this approach to lithiation adjacent to a nitrogen atom by Beak and co‐workers using sparteine as a chiral ligand resulted in moderate enantioselectivity of the recovered *N*‐*tert*‐butoxycarbonyl (*N*‐Boc) α‐methylbenzylamine [12 % yield, enantiomer ratio (er) 81 : 19].^[23]^ In contrast to this acyclic example, our research group found that very good results were obtained on kinetic resolution of *N‐*Boc‐2‐arylpiperidines.^[24]^ This has recently been extended to related cyclic substrates,[^25^, ^26^] including *N*‐Boc‐2‐aryltetrahydroquinolines.^[27]^ This kinetic resolution chemistry provides a method to access 2‐aryltetrahydroquinolines with good enantioselectivities (relative reactivity, *k*_rel_, or selectivity factor, s ∼20),^[28]^ together with 2,2‐disubstituted tetrahydroquinolines. Here, we describe our work with the related dihydroquinoline substrates that lead to highly enantioenriched 2‐aryldihydroquinolines and, in contrast to the saturated analogs, substitution at C‐4 rather than C‐2 of the heterocycle.

## Results and Discussion

**Scheme 2:**
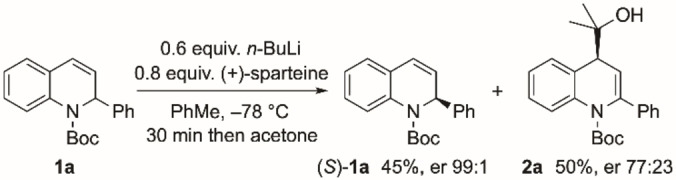
Kinetic resolution of dihydroquinoline **1** **a**.

A simple one‐pot procedure provided access to the desired 1,2‐dihydroquinoline substrates.^[29]^ This involved addition of the aryllithium to quinoline followed directly by addition of di‐*tert*‐butyl dicarbonate (Boc_2_O) (see Supporting Information). Initial investigations into the lithiation chemistry centred on the parent 1,2‐dihydroquinoline **1** **a** (Scheme 2). After optimization, it was found that addition of 0.6 equivalents of *n*‐BuLi to a mixture of **1** **a** and 0.8 equivalents of the chiral ligand (+)‐sparteine in toluene at −78 °C, followed by addition of the electrophile acetone after 30 min, provided good results in the kinetic resolution. Under these conditions, the recovered 1,2‐dihydroquinoline **1** **a** was isolated with excellent enantiomer ratio (er 99 : 1). The electrophile‐trapped product was found to be the 2,3‐dihydroquinoline **2** **a** in which substitution occurred at C‐4. Trapping the intermediate allyllithium species at the gamma position was expected from related chemistry,[^30^, ^31^] and the result therefore allows access to 4‐substituted quinoline derivatives. The enantiomer ratio of the 2,3‐dihydroquinoline **2** **a** was variable and not as high as the recovered 1,2‐dihydroquinoline **1** **a**, suggesting that the organolithium (or the product) racemizes slowly under these conditions, although shorter reaction times improve the er of the 4‐substituted product **2** **a**.

**Scheme 3:**
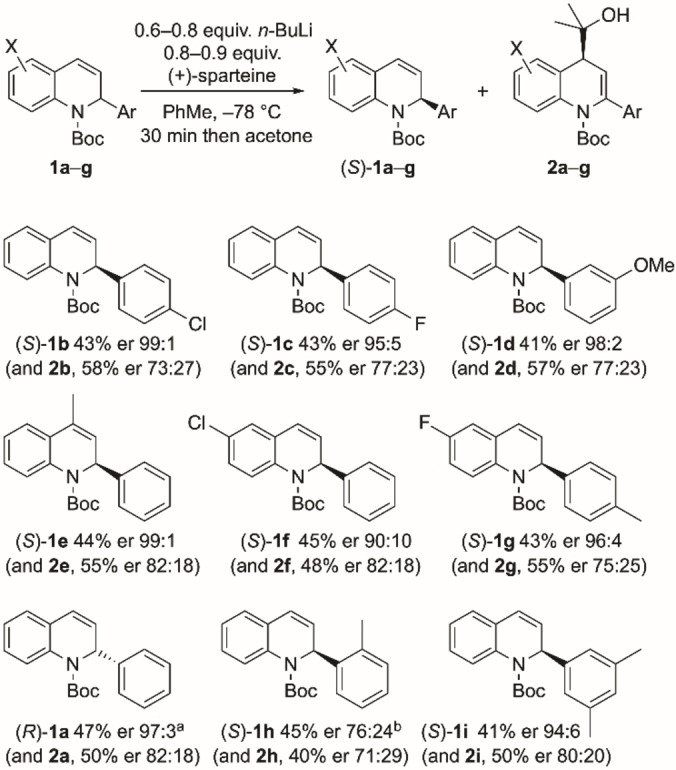
Kinetic resolution of dihydroquinolines **1**.

**Figure 1:**
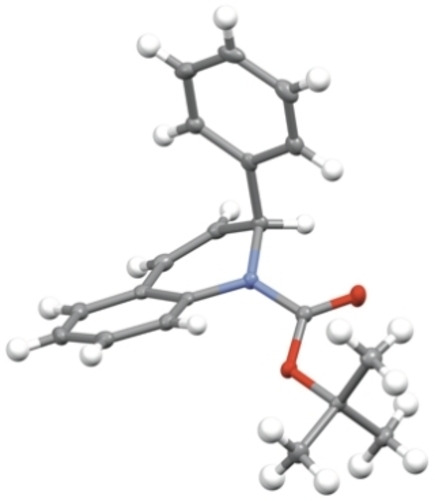
Single crystal X‐ray analysis of (*S*)‐**1** **a**.

The scope of the kinetic resolution was explored with a variety of substituted 1,2‐dihydroquinolines **1** **b**–**i** (Scheme 3). In almost all cases, high selectivities of the recovered 1,2‐dihydroquinolines were obtained. The chemistry therefore provides an efficient kinetic resolution with a selectivity factor, s∼20, in which the combination of *n*‐BuLi and the chiral ligand (+)‐sparteine provides a chiral base that favors deprotonation of one enantiomer of the racemic 1,2‐dihydroquinoline. Single crystal X‐ray analysis of the enantioenriched 1,2‐dihydroquinoline **1** **a** isolated from Scheme 2 verified the absolute configuration as (*S*) (Figure 1).^[32]^ The selectivity matches that expected for the use of sparteine in related lithiations of *N*‐Boc cyclic amines.[^33^, ^34^, ^35^, ^36^] The kinetic resolution tolerates a variety of functional groups, including chloride, fluoride, methoxy, methyl at various locations on the 2‐aryl or dihydroquinoline rings, although a methyl group in the *ortho* position (compound **1** **h**) reduces the selectivity (Scheme 3).

The lithiation of unsymmetrical *N*‐Boc cyclic amines depends on the ability of the Boc group to rotate, since coordination of *n*‐BuLi occurs to the carbonyl oxygen atom prior to reaction (complex induced proximity effect).^[37]^ It is therefore important to know the ratio of rotamers and the rate of rotation of the Boc group.

**Figure 2:**
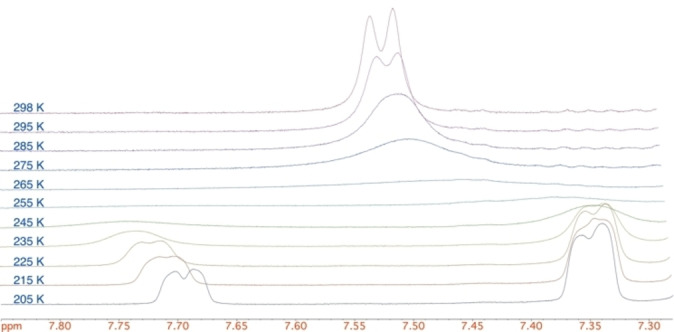
^1^H VT NMR spectroscopy of dihydroquinoline **1** **a** (400 MHz, D_8_‐THF) showing 7.85–7.30 ppm and coalescence of C_8_‐H.

Variable temperature (VT) NMR studies were performed with 1,2‐dihydroquinoline **1** **a** in D_8_‐THF (THF is a suitable solvent for lithiation‐trapping as described below) (Figure 2). From the NMR spectra, the ratio of rotamers is approximately 2 : 1. This could potentially limit the effectiveness of lithiation at C‐2, unless rotation is fast. Line shape and Eyring plot analyses (see Supporting Information) gave activation parameters for rotation of the Boc group of ΔH^≠^ ≈ 47 kJ mol^−1^ and ΔS^≠^ ≈ −13 J K^−1^ mol^−1^ for the major rotamer converting to the minor rotamer, and ΔH^≠^≈47 kJ mol^−1^ and ΔS^≠^ ≈ −7 J K^−1^ mol^−1^ for minor to major. The barrier to rotation, ΔG^≠^, of both rotamers is therefore ≈49 kJ mol^−1^ at −78 °C; this equates to a half‐life for rotation of only about 3 sec at this temperature. Hence rotation of the Boc group is rapid and the lithiation should be unaffected by the presence of both rotamers in solution.

**Figure 3:**
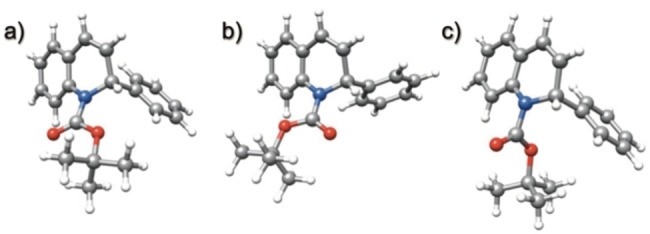
Optimized geometries of **1** **a** in THF: a) and b) minimum energy structures for each rotamer, c) lowest energy transition state for rotation of the Boc group.

These results were supported by density functional theory (DFT) calculations on 1,2‐dihydroquinoline **1** **a** (see Supporting Information). Using the B3LYP functional^[38]^ (as reported previously),[^25^, ^39^] which includes dispersion interactions, and the def2TZVP basis set (B3LYP‐D3BJ//def2‐TZVP),^[40]^ the minimal energy structures for the rotamers of 1,2‐dihydroquinoline **1** **a** were found to be when the phenyl group occupied an axial position (Figure 3a and 3b). The calculations suggest that the rotamer of 1,2‐dihydroquinoline **1** **a** with the Boc carbonyl directed towards C‐8 is lower in energy than when the carbonyl is directed towards C‐2 by 1.2 kJ mol^−1^. Transition state calculations were then carried out to determine the lowest energy transition state for rotation of the Boc group (Figure 3c), where the Gibbs energy of activation was calculated to be ≈43 kJ mol^−1^ at −78 °C (with ΔH^≠^≈38 kJ mol^−1^ and ΔS^≠^≈−25 J K^−1^ mol^−1^). These values matched reasonably well with the results obtained from VT NMR spectroscopy. The low barrier to rotation means that interconversion between the rotamers occurs readily.

The 2 : 1 ratio of rotamers of **1** **a**, in which the carbonyl prefers to be directed towards C‐8, is in contrast with the related indoline containing a 5‐ rather than a 6‐membered ring, which has a ratio of rotamers of 6 : 1.^[25]^ From analysis of the calculated structures, the larger 6‐membered ring clearly favors the partially saturated ring to be puckered. From the X‐ray data (Figure 1), the carbonyl group is directed towards C‐2 and is out of plane of the benzene ring by 44°. Therefore the *tert*‐butyl group can be located away from the *ortho* aromatic proton (C_8_−H). In addition, the carbonyl oxygen atom is located 2.28 Å from the proton at C‐2, indicating a favorable electrostatic interaction in this rotamer. These factors may explain why there is a greater proportion of the rotamer with the carbonyl directed to C‐2 in the six‐membered ring analog.

Finally, a qualitative analysis was carried out by following the carbonyl stretch in 1,2‐dihydroquinoline **1** **a** on addition of *n*‐BuLi using in situ IR spectroscopy.^[41]^ Complete lithiation took place within a few minutes at −78 °C in THF (see Supporting Information). This result also supports the conclusion that rotation of the Boc group is rapid even at low temperature.

**Scheme 4:**
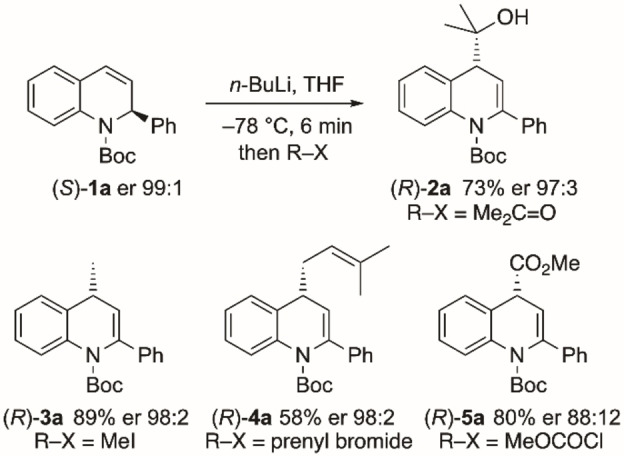
Lithiation‐trapping of dihydroquinoline (*S*)‐**1** **a**.

**Figure 4:**
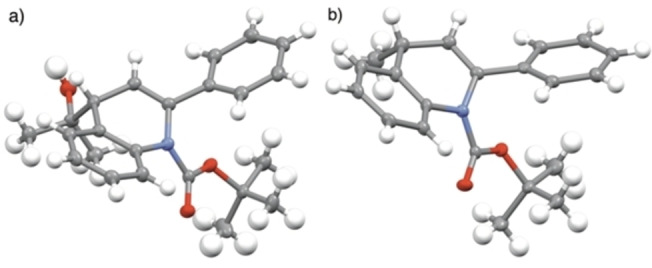
Single crystal X‐ray analyses of: a) (*R*)‐**2** **a** and b) (*R*)‐**3** **a**.

The kinetic resolution chemistry (Scheme 3) provides the recovered starting materials **1** **a**–**i** with high enantioselectivites. If the kinetic resolution was conducted to low conversions the products (*S*)‐**2** or (*R*)‐**2** (depending on the choice of enantiomer of sparteine) could be formed with high enantioselectivities, however these products can alternatively be prepared from the starting material **1** recovered after the kinetic resolution. Hence, treatment of the recovered starting material (*S*)‐**1** **a** with *n*‐BuLi in THF at low temperature followed by trapping the intermediate organolithium species with an electrophile gave the 4‐substituted products (*R*)‐**2** **a**–**5** **a** without significant loss in enantiopurity (Scheme 4). The lithiation is rapid and the electrophilic quench occurs with retention of configuration, as determined by single crystal X‐ray analyses of the products (*R*)‐**2** **a** and (*R*)‐**3** **a** (Figure 4).^[32]^ Best enantioselectivities of the product **5** **a** were obtained when methanol was added a few minutes after the addition of methyl chloroformate, presumably to minimize racemization via the enolate.

**Scheme 5:**
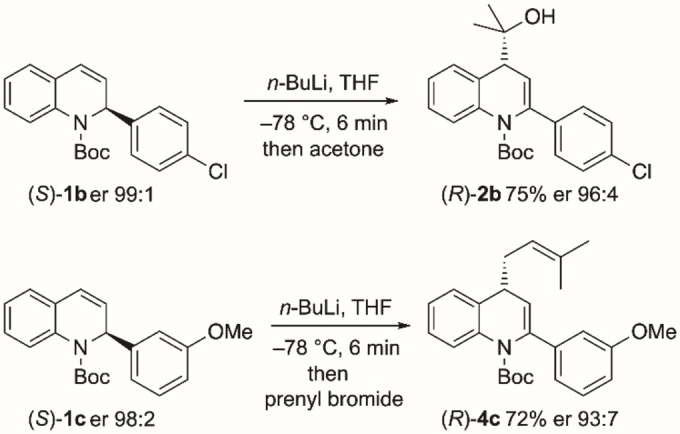
Lithiation‐trapping of dihydroquinolines (*S*)‐**1** **b** and (*S*)‐**1** **c**.

The lithiation‐trapping chemistry was extended to the derivatives (*S*)‐**1** **b** and (*S*)‐**1** **c** (Scheme 5). The 4‐substituted 1,4‐dihydroquinolines (*R*)‐**2** **b** and (*R*)‐**4** **c** were prepared with high enantioselectivity. The absolute configuration shown is that expected for the major enantiomer based on the examples with (*S*)‐**1** **a**. The single chiral ligand (+)‐sparteine was therefore used to prepare either enantiomer of a selection of 1,4‐dihydroquinolines using the kinetic resolution and subsequent lithiation‐trapping of the recovered 1,2‐dihydroquinolines.

**Scheme 6:**
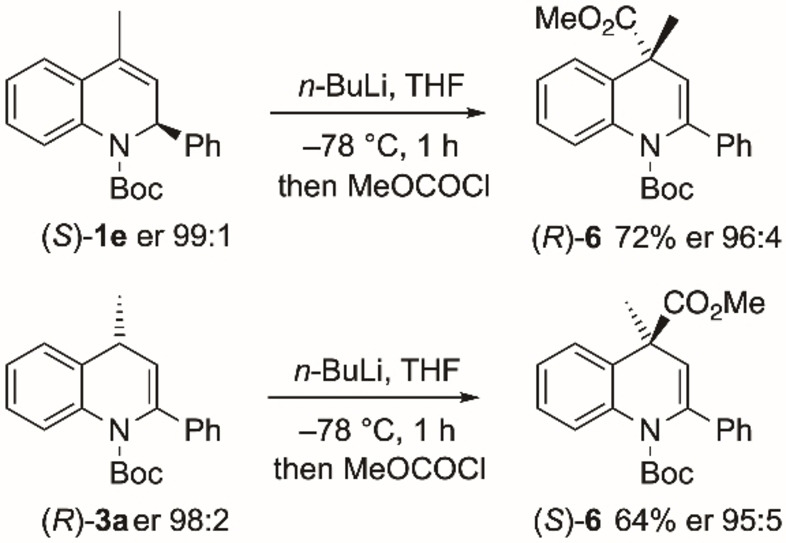
Lithiation‐trapping of 4‐methyldihydroquinolines (*S*)‐**1** **e** and (*R*)‐**3** **a**.

**Figure 5:**
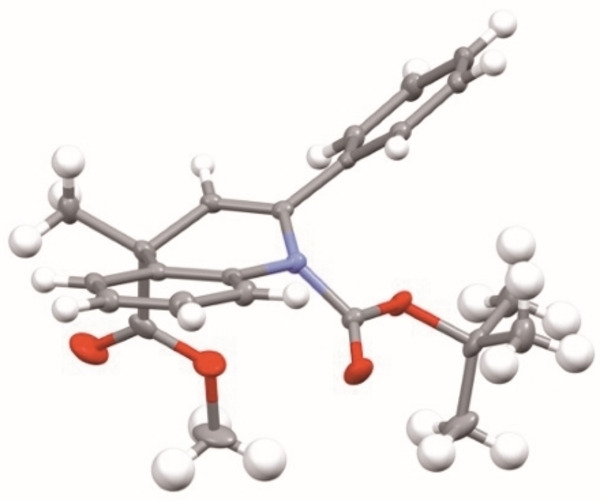
Single crystal X‐ray analysis of (*R*)‐**6**.

In a similar way to the examples in Schemes 4 and 5, lithiation then trapping of the 4‐methyl derivative (*S*)‐**1** **e** was successful (Scheme 6). The lithiation was slower with the 4‐methyl substituent but after 1 h, electrophilic quench with methyl chloroformate resulted in a good yield of the 4,4‐disubstituted product (*R*)‐**6** (er 96 : 4, with er 99 : 1 after recrystallization). The trapping with methyl chloroformate occurred with retention of configuration, as determined by single crystal X‐ray analysis of the product (*R*)‐**6** (Figure 5).^[32]^ We were interested to determine whether lithiation could be conducted on the 1,4‐dihydroquinoline and were pleased to find that the same conditions (*n*‐BuLi, THF, −78 °C, 1 h) allowed formation of the product (*S*)‐**6** from (*R*)‐**3** **a** (Scheme 6). Hence dihydroquinoline **3** **a** can undero stereospecific lithiation‐trapping despite requiring proton abstraction at C‐4 which would not be expected to be directed by the Boc group. The conversion of (*R*)‐**3** **a** to (*S*)‐**6** with high selectivity is remarkable as it must involve the formation of a configurationally stable allylic and benzylic organolithium that also presumably lacks internal coordination from the Boc group. Either enantiomer of these 4,4‐disubstituted dihydroquinolines can be accessed by kinetic resolution followed by further lithiation‐trapping from either the 4‐monosubstituted or 4‐unsubstituted quinoline starting material. For both cases shown in Scheme 6 the same chiral ligand (+)‐sparteine was used to access either enantiomer of the 4,4‐disubstituted product. Hence there is flexibility regarding the choice of substrate, electrophile, and enantiomer of sparteine.

**Figure 6:**
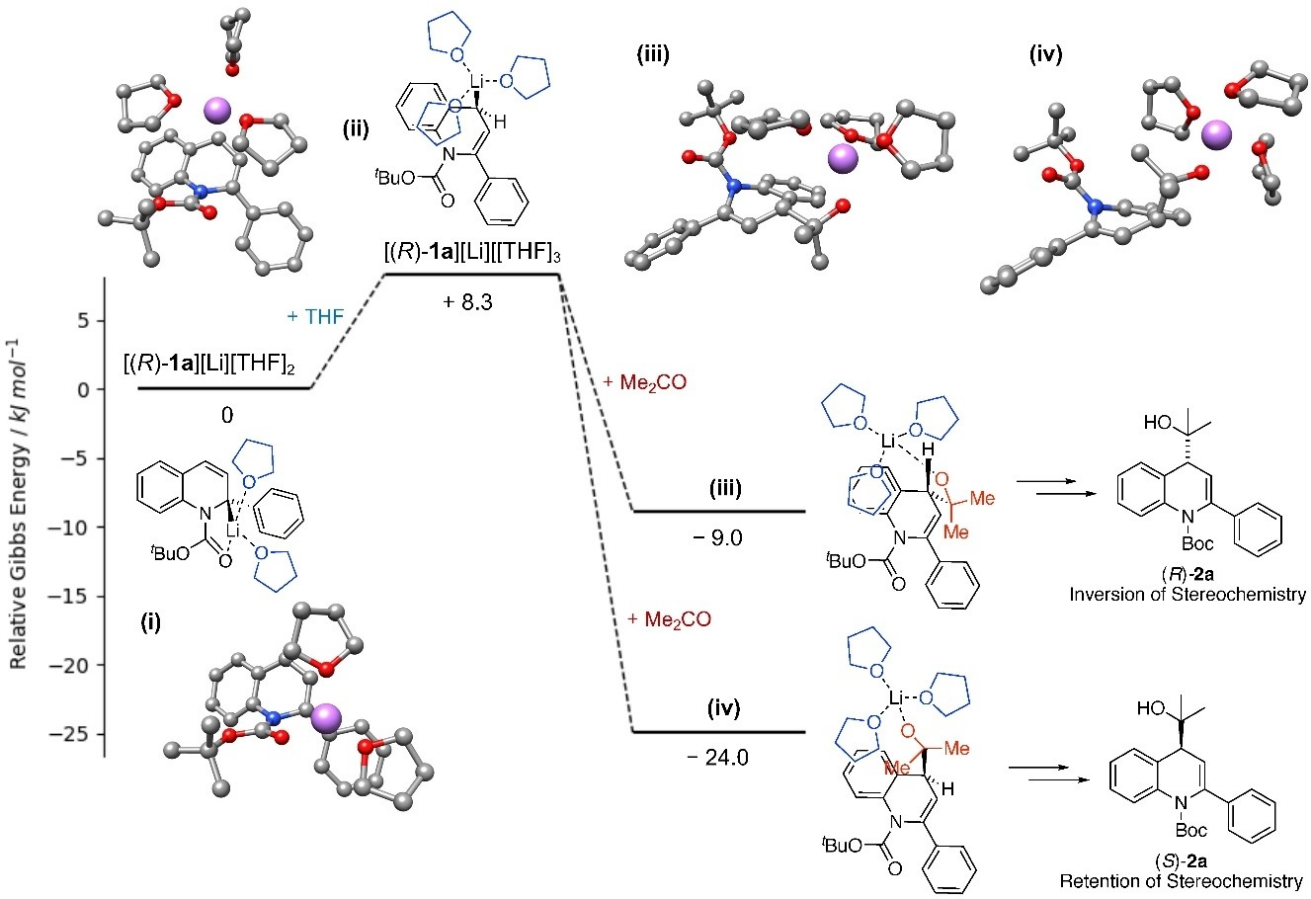
Energy profile for the lithiation‐trapping of (*R*)‐**1** **a** with acetone. Relative Gibbs free energies are in kJ/mol at 195 K.

To rationalize the retention of configuration during the lithiation‐trapping experiments on the enantioenriched 2‐aryldihydroquinolines, DFT calculations were performed on (*R*)‐**1** **a** investigating the lithiation pathway (Figure 6). Using the B3LYP−D3BJ functional with the 6–311G** basis set (B3LYP‐D3BJ//6‐311G**)^[42]^ optimisation of the organolithium formed from (*R*)‐**1** **a** gave a structure where the lithium atom was close to C‐2, coordinated to the carbonyl oxygen atom of the Boc group and two molecules of THF (Figure 6i), i. e. a coordinationally saturated Li‐ion. These calculations assume that the organolithiums are monomers, as found for related structures.^[43]^ To investigate the reaction, we introduced first a molecule of acetone. This leads to a structure that still has Li coordinated to the Boc group, but no longer to the C‐2 position (see Supporting info) with a Gibbs energy decrease of 1.0 kJ mol^−1^. On the other hand, introduction of another molecule of THF (which is more likely, given its concentration) led to the migration of the lithium atom to the C‐4 position. In the optimized structure obtained in this case (Figure 6ii), coordination of the lithium atom to the carbonyl oxygen of the Boc group was no longer present. Instead, the lithium atom was found to be close to the C‐4 position resulting in a double bond forming between the C‐2 and C‐3 positions with only a small increase in Gibbs energy of 8.3 kJ mol^−1^. With these minimized structures of the organolithium intermediate of (*R*)‐**1** **a**, the quenching process with acetone was investigated.

On trapping with acetone, the initial step is formation of the alkoxide intermediate, which can be formed through acetone reacting from either the same face as the lithium atom or the opposite face. The lowest free energy structure (24 kJ mol^−1^ below the organolithium species formed from (*R*)‐**1** **a**) was found when acetone reacted from the same face as the lithium atom (Figure 6iv) which results in the formation of (*S*)‐**2** **a** where the stereochemistry of lithiation had been retained. The alternative geometry, where the stereochemistry of lithiation is inverted has a Gibbs energy which is 15 kJ mol^−1^ higher. Unfortunately, we were not able to find a transition state in either case. If, on the other hand, the lithiated intermediate (Figure 6i) is reacted directly with acetone at C‐2 then the free energy of the resulting intermediate is 46.6 kJ mol^−1^ above the free energy of the structure in Figure 6i, which makes that process very unlikely.

**Scheme 7:**
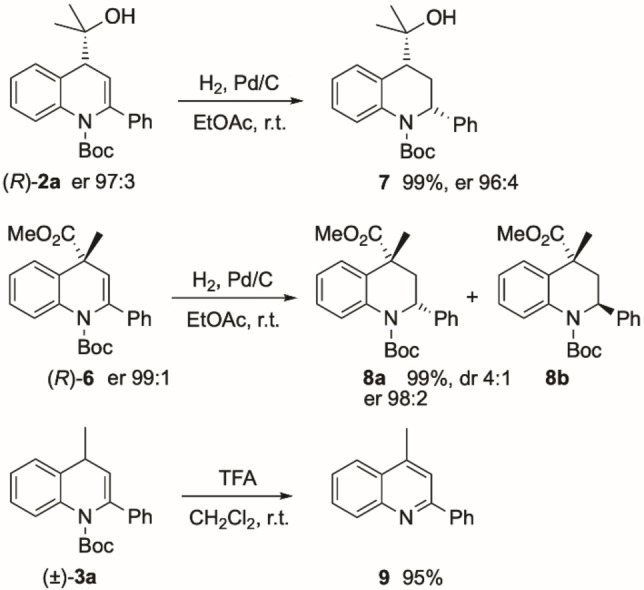
Reduction and oxidation of dihydroquinolines.

**Figure 7:**
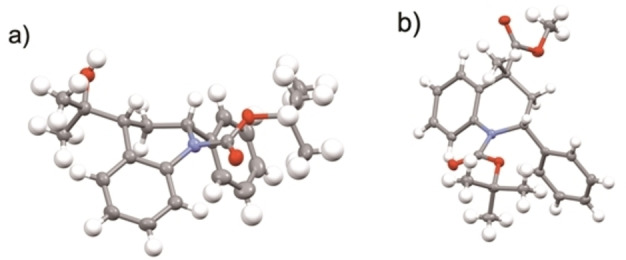
Single crystal X‐ray analyses of a) **7** and b) **8** **a**.

We explored other transformations of the substituted products. Hydrogenation of the alkene (*R*)‐**2** **a** resulted in essentially quantitative yield of the 2,4‐disubstituted tetrahydroquinoline **7** (Scheme 7). This was formed as a single diastereoisomer (*cis*) with almost no loss in enantiopurity. The absolute configuration was verified by single crystal structure analysis (Figure 7a).^[32]^ In the same way, hydrogenation of the 4,4‐disubstituted dihydroquinoline (*R*)‐**6** gave the tetrahydroquinolines **8** **a** and **8** **b**. These were formed as a mixture of diastereoisomers (dr ∼4 : 1, er 98 : 2 of major isomer). The major diastereoisomer **8** **a** crystallized as a racemate and the relative stereochemistry was determined by single crystal X‐ray analysis (Figure 7b).^[32]^ Hydrogenation is preferred opposite to the methyl ester group, presumably for steric reasons. As well as reduction to give the important tetrahydroquinoline structures, we were able to perform an oxidation in air simply by removal of the *N*‐Boc group with acid. Using the substrate **3** **a**, the resulting *NH*‐dihydroquinoline undergoes oxidation in situ to give the quinoline **9** (Scheme 7).

**Scheme 8:**
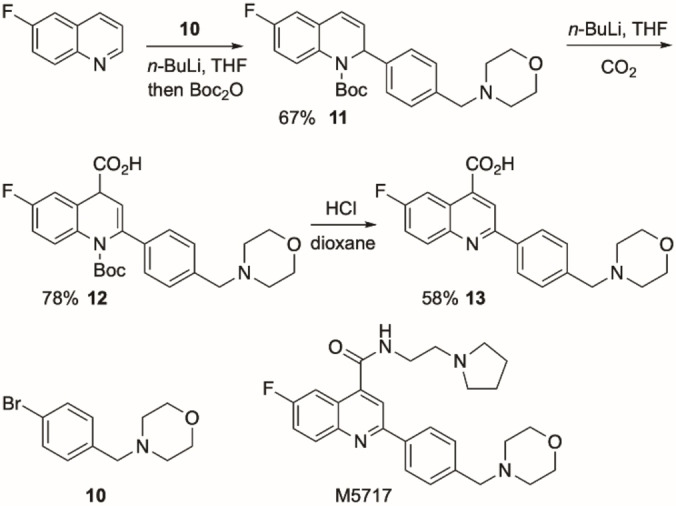
Application to the quinoline M5717.

Considering the ease of reduction or oxidation of the dihydroquinolines, we sought to exploit the chemistry and apply it to a tetrahydroquinoline or quinoline target. We were attracted to the antimalarial drug M5717, which is in clinical trials.^[44]^ A short route to this drug was developed from 6‐fluoroquinoline (Scheme 8). Addition of the aryllithium generated from the arylbromide **10** and direct in situ Boc protection of the intermediate gave the 1,2‐dihydroquinoline **11**. Subsequent deprotonation was followed by trapping the organolithium with milled dry ice.^[45]^ This gave the 1,4‐dihydroquinoline **12**. Acid‐promoted removal of the *N*‐Boc group caused concommitant oxidation to form the quinoline **13**. The quinoline **13** is a known compound that can be converted to M5717 in a single step by amide formation.^[46]^

In addition, it is possible to envisage preparing enantioenriched dihydro‐ and tetrahydroquinoline derivatives of this or related drug compounds by using the kinetic resolution chemistry. Treating 1,2‐dihydroquinoline **11** with *n*‐BuLi and the chiral ligand (+)‐sparteine followed by trapping with acetone resulted in enantioenriched recovered **11** (46 %, er 84 : 16) (see Supporting Information). Alternatively, kinetic resolution of the 1,2‐dihydroquinoline **1** **g** (with a tolyl group at C‐2) with *n*‐BuLi and (+)‐sparteine gave recovered **1** **g** (43 %, er 96 : 4 as shown in Scheme 3) and the 2‐tolyl derivative of M5717 has shown good activity as an antimalarial compound.^[44]^

## Conclusion

The asymmetric synthesis of dihydro‐ and tetrahydroquinolines was achieved using a kinetic resolution of 1,2‐dihydroquinolines with *n*‐BuLi and the chiral ligand sparteine. High enantioselectivities were obtained for the formation of a range of 2‐aryl derivatives. Lithiation at C‐2 and trapping at C‐4 with an electrophile led to enantiomerically enriched 1,4‐dihydroquinolines with retention of stereochemistry.

The rotation of the *N‐*carbonyl (Boc) group was found, from variable temperature NMR spectroscopy and DFT studies, to be fast (ΔG^≠^≈49 kJ mol^−1^ at −78 °C) and therefore the presence of two Boc rotamers does not affect the ability to undergo efficient lithiation at C‐2 even at low temperature.

Both enantiomers of 2,4‐disubstituted 1,4‐dihydroquinolines can be obtained using the same enantiomer of the chiral ligand. The chemistry can be extended to give highly enantioenriched 2,4,4‐trisubstituted 1,4‐dihydroquinolines and tetrahydroquinolines. In addition, removal of the *N*‐Boc group with acid allows the preparation of quinolines and this chemistry can be applied in synthesis, as demonstrated by a formal synthesis of the antimalarial compound M5717.

## Experimental Section

Details of the kinetic resolution are given here, with full experimental details in the Supporting Information.

**tert‐Butyl (2S)‐2‐Phenyl‐2H‐quinoline‐1‐carboxylate (S)‐1** **a and tert‐Butyl 4‐(2‐hydroxypropan‐2‐yl)‐2‐phenyl‐4H‐quinoline‐1‐carboxylate 2** **a**: *n*‐BuLi (0.27 mL, 0.62 mmol, 2.3 M in hexanes) was added to a mixture of freshly distilled (+)‐sparteine (209 mg, 0.89 mmol) and the racemic carbamate **1** **a** (319 mg, 1.0 mmol) in dry PhMe (24 mL) at −78 °C. After 30 min, acetone (0.15 mL, 2.1 mmol) was added. After 30 min, MeOH (2 mL) was added and the mixture was allowed to warm to room temperature. The solvent was evaporated, and the residue was purified by column chromatography on silica gel, eluting with petrol‐EtOAc (88 : 12), to give recovered carbamate (*S*)‐**1** **a** (150 mg, 45 %) as an amorphous solid; m.p. 49–51 °C; R_*f*_ 0.45 [petrol‐Et_2_O (98 : 2)]; *ν*_max_/cm^−1^ (film) 3055, 2987, 1695, 1265; ^1^H NMR (400 MHz, CDCl_3_) δ=7.63‐7.49 (1H, m, ArH), 7.33–7.02 (8H, m, ArH), 6.67 (1H, d, *J* 9 Hz, CH), 6.26–6.12 (2H, m, 2 x CH), 1.59 (9H, s, ^t^Bu); ^13^C NMR (100 MHz, CDCl_3_) δ=153.5, 140.2, 135.3, 128.5, 128.3, 127.7, 127.5, 127.1, 127.0, 126.3, 125.4, 124.8, 123.8, 81.6, 55.3, 28.4; HRMS (ES) found MH^+^, 330.1471. C_20_H_22_NO_2_ requires MH^+^, 330.1465; LRMS (ES) 330 (60 %), 252 (100 %), 208 (10 %); the enantiomers were resolved using CSP‐HPLC (Cellulose‐1, n‐hexane‐isopropanol=99 : 1, flow rate=1.0 mL/min, I=254 nm, t_R_=5.6 min and 6.2 min) and the enantiomeric ratio was determined to be 99 : 1 (major component eluted at 5.4 min); [α]_D_^23^ −609 (1.2, CHCl_3_).

In addition, the carbamate **2** **a** (180 mg, 50 %) was isolated as an amorphous solid; m.p. 109 – 112 °C; R_*f*_ 0.38 [petrol‐EtOAc (60 : 40)]; *ν*_max_/cm^−1^ (film) 3058, 2975, 1713, 1602, 1582, 1266, 1162; ^1^H NMR (400 MHz, CDCl_3_) δ=7.94 (1H, d, *J* 8 Hz, ArH), 7.52‐7.18 (8H, m, ArH), 5.85 (1H, d, *J* 7.5 Hz, CH), 3.55 (1H, d, *J* 7.5 Hz, CH), 1.73 (1H, s, OH), 1.37 (3H, s, CH_3_), 1.21 (3H, s, CH_3_), 1.14 (9H, s, ^t^Bu); ^13^C NMR (100 MHz, CDCl_3_) δ=152.4, 142.5, 139.8, 139.2, 131.7, 129.4, 128.1, 127.6, 126.6, 125.3, 124.9, 124.8, 116.9, 81.6, 74.9, 51.6, 27.6, 27.0, 25.9; HRMS (ES) found MNa^+^, 388.1890. C_23_H_27_NO_3_Na requires MNa^+^, 388.1883; LRMS (ES) found 388 (80 %), 292 (30 %), 266 (100 %); the enantiomers were resolved using CSP‐HPLC (Cellulose‐1, n‐hexane‐isopropanol=99 : 1, flow rate=1.0 mL/min, I=254 nm, t_R_=19.2 min and 22.5 min) and the enantiomeric ratio was determined to be 77 : 23 (major component eluted at 20.8 min; [α]_D_^23^ −41 (0.3, CHCl_3_).

## Supporting Information

We are grateful for support of this research by the EPSRC (grant EP/R024294/1), the Royal Society (Short Industry Fellowship SIF\R2\202031), and the University of Sheffield. We thank Craig Robertson (University of Sheffield) and the UK National Crystallography Service for the single crystal X‐ray analyses and Sandra van Meurs (University of Sheffield) for help with NMR spectra. Daniel Albrighton, Soneni Ndlovu, and Jasmine Hall are thanked for related studies. We acknowledge the Faculty of Science mass spectrometry service at the University of Sheffield. For the purpose of open access, the author has applied a Creative Commons Attribution (CC BY) licence to any Author Accepted Manuscript version arising.

## Conflict of interest

The authors declare no conflict of interest.

1

## Supporting information

As a service to our authors and readers, this journal provides supporting information supplied by the authors. Such materials are peer reviewed and may be re‐organized for online delivery, but are not copy‐edited or typeset. Technical support issues arising from supporting information (other than missing files) should be addressed to the authors.

Supporting Information

## Data Availability

The data that support the findings of this study are available in the supplementary material of this article.
