# Supplementary material for: Kinetic Resolution of 2‐Aryldihydroquinolines Using Lithiation – Synthesis of Chiral 1,2‐ and 1,4‐Dihydroquinolines
Source: Chemistry. 2023 May 5;29(36):e202300815. doi: 10.1002/chem.202300815 (PMC10946909; doi:10.1002/chem.202300815)
Supplement: Supplementary file 1 — Supporting Information [file CHEM-29-0-s001.pdf]

Chemistry–A European Journal

Supporting Information

Kinetic Resolution of 2-Aryldihydroquinolines Using Lithiation – Synthesis of Chiral 1,2- and 1,4-Dihydroquinolines

Song-Hee Yeo, Anthony Choi, Sophie Greaves, Anthony J. H. M. Meijer,
Ilaria Proietti Silvestri, and Iain Coldham*

Contents

1. General procedures S-2
2. Experimental procedures and data S-3
   - 2.1 Synthesis of racemic substrates **1** S-3
   - 2.2 Kinetic resolution of racemic substrates **1** S-9
   - 2.3 Lithiation–trapping of enantioenriched substrates S-18
   - 2.4 Reduction and oxidation of dihydroquinolines S-22
   - 2.5 Application to M5717 S-24
3. Variable temperature ^{1}H NMR spectra and *in situ* IR spectra S-27
4. Single crystal X-ray data S-30
5. References S-72
6. HPLC traces S-73
7. NMR spectra S-99
8. DFT data S-133
   - 8.1 Rotation calculations S-133
   - 8.2 Lithiation pathway calculations S-166

1. General procedures

Reagents were obtained from commercial suppliers and were used without further purification or after distillation; *n*BuLi was titrated before use. Solvents were obtained from a Grubbs dry solvent system. Thin layer chromatography was performed on Merck silica gel $60F_{254}$ plates and visualised by UV irradiation at 254 nm or by staining with an alkaline $KMnO_4$ dip. Flash column chromatography was performed using DAVISIL or Geduran silica gel (40-63 micron mesh). Heating was performed using aluminum heating blocks on a hotplate stirrer. Melting points were recorded on a Gallenkamp hot stage and were uncorrected. InfraRed spectra were recorded on a Perkin Elmer Spectrum RX Fourier Transform – IR System and only selected peaks are reported. ^1H NMR spectra were recorded on a Bruker AC400 (400 MHz) instrument at ambient temperature. Chemical shifts are reported in ppm with respect to the residual $CHCl_3$ or C_6D_5H solvent peaks, with multiplicities given as s = singlet, d = doublet, t = triplet, q = quartet, m = multiplet, br = broad. Coupling constants (*J* values) are quoted to the nearest 0.5 Hz with values in Hertz (Hz). ^{13}C NMR spectra were recorded on the above instrument at 100 MHz. Low and high resolution (accurate mass) mass spectra were recorded on an Agilent Technologies LC-MS quadrupole time-of-fight (Q-TOF) instrument for ElectroSpray (ES). ReactIR infrared spectroscopic monitoring was performed on a Mettler-Toledo ReactIR iC 4000 spectrometer equipped with a diamond-tipped (DiComp) probe.

Intensity data for X-ray crystal structures were collected at 100 K on a Bruker D8 Venture diffractometer using a Cu K_α microfocus X-ray source. Suitable crystals were mounted on a MiTiGen microloop using fomblin oil and transferred directly to the cold nitrogen stream at 100 K for data collection on a Bruker D8 VENTURE diffractometer equipped with an Oxford 700+ cryostream, a PHOTON 100 CMOS detector and using Cu-K_α micro-focus X-ray source. Intensity data was collected in shutterless mode with a final fast scan collected at lower incident beam intensity to enable correction for any detector saturation for low-angle data. Data reduction was performed using the Bruker Apex3 software.[47] Intensity data were corrected for absorption using empirical methods (SADABS) based upon symmetry equivalent reflections combined with measurements at different azimuthal angles.[48] The crystal structure was solved using ShelXT[49] and refined against all F^2 values using the SHELXL[50] accessed via the Olex2 program.[51] Non-hydrogen atoms were refined anisotropically. Hydrogen atoms were placed in calculated positions with idealized geometries and then refined by employing a riding model and isotropic displacement parameters. The intense X-ray beam and high sensitivity of the detector allow determination of absolute configuration of small organic molecules with atoms no heavier than oxygen.

The selectivity factors (s) in the manuscript were calculated using the formula

$$s = \ln[(1-c)(1-ee)] / \ln[(1-c)(1+ee)]$$

where c is the conversion based on the amount of recovered starting material and ee is the enantiomeric excess of the recovered starting material.

All calculations were performed using density functional theory, employing the B3LYP[38] functional as implemented in the D.01 version of Gaussian 09.[52] Calculations included dispersion corrections using the GD3-BJ[42] method. Calculations used the 6-311G(d,p)[53] or def2TZVP[40] basis set. Solvent was included *via* the PCM method[54] as implemented in Gaussian with the default parameters for THF.

2. Experimental procedures and data

2.1 Synthesis of racemic substrates **1**

(±)-*tert*-Butyl 2-Phenyl-2*H*-quinoline-1-carboxylate **1a**

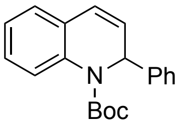

1a

PhLi (29 mL, 29 mmol) was added to quinoline (4.6 mL, 39 mmol) in dry Et_2O (50 mL) at −78 °C. A solution of Boc_2O (14.3 g, 65.5 mmol) in dry Et_2O (25 mL) was added and the mixture was allowed to warm to room temperature over 16 h. Et_2O (20 mL) was added and the mixture was washed with water (3 × 5 mL). The organic layer was dried ($MgSO_4$) and evaporated. Purification by column chromatography on silica gel, eluting with petrol−EtOAc (99:1 to 97:3), gave the carbamate **1a** (8.9 g, 75%) as an amorphous solid; m.p. 67−69 °C; R_f 0.45 [petrol−Et_2O (98:2)]; ν_{max}/cm^{-1} (film) 3055, 2987, 1695, 1265; ^1H NMR (400 MHz, $CDCl_3$) δ = 7.63−7.49 (1H, m, ArH), 7.33−7.02 (8H, m, ArH), 6.67 (1H, d, *J* 9 Hz, CH), 6.26−6.12 (2H, m, 2 x CH), 1.59 (9H, s, ^tBu); ^{13}C NMR (100 MHz, $CDCl_3$) δ = 153.5, 140.2, 135.3, 128.5, 128.3, 127.7, 127.5, 127.1, 127.0, 126.3, 125.4, 124.8, 123.8, 81.6, 55.3, 28.4; HRMS (ES) found MH^+, 330.1471. $C_{20}H_{22}NO_2$ requires MH^+, 330.1465; LRMS (ES) 252 (100%), 330 (60%), 208 (10%); data consistent with the literature ^1H NMR spectrum.[55] The enantiomers were resolved using HPLC (Cellulose–1, n-hexane–isopropanol = 99:1, flow rate = 1.0 mL/min, l = 254 nm), t_R =5.6 min and 6.2 min.

(±)-*tert*-Butyl 2-(4-Chlorophenyl)-2*H*-quinoline-1-carboxylate **1b**

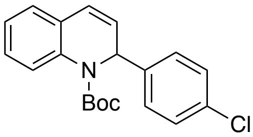

1b

n-BuLi (12 mL, 28 mmol, 2.4 M in hexane) was added to 1-bromo-4-chlorobenzene (5.3 g, 28 mmol) in dry THF (15 mL) at −78 °C. After 1 h, quinoline (2.8 mL, 23 mmol) in dry THF (15 mL) was added at 0 °C. After 1 h, Boc_2O (7.6 g, 35 mmol) in dry THF (5 mL) was added and the mixture was allowed to warm to room temperature over 16 h, then MeOH (5 mL) was added and the solvent was evaporated. Purification by column chromatography on silica gel, eluting with petrol−Et_2O (98:2), gave the carbamate **1b** (4.2 g, 53%) as an amorphous solid; m.p. 88−90 °C; R_f 0.29 [petrol−Et_2O (9:1)]; ν_{max}/cm^{-1} (film) 3042, 2975, 1691, 1158, 872; ^1H NMR (400 MHz, $CDCl_3$) δ = 7.56−7.46 (1H, m, ArH), 7.24−7.02 (7H, m, ArH), 6.68 (1H, d, *J* 9.0 Hz, CH), 6.20−6.09 (2H, m, 2 x CH), 1.56 (9H, s, ^tBu); ^{13}C NMR (100 MHz, $CDCl_3$) δ = 153.4, 138.5, 134.9, 133.5, 128.6, 128.5, 127.7, 126.9, 126.3, 125.8, 124.7, 123.9, 118.6, 81.8, 54.5, 28.4; HRMS (ES) found MNa^+, 364.1085. $C_{20}H_{20}NO_2{}^{35}ClNa$ requires MNa^+, 364.1080; Found MNa^+, 366.1060. $C_{20}H_{20}NO_2{}^{37}ClNa$ requires MNa^+, 366.1051; LRMS (ES) 364 (100%), 366 (33%). The enantiomers were resolved using HPLC (Cellulose–2, n-hexane–isopropanol = 99.3:0.7, flow rate = 1.0 mL/min, l = 254 nm), t_R = 4.5 min and 6.1 min.

(±)-*tert*-Butyl 2-(4-Fluorophenyl)-2*H*-quinoline-1-carboxylate **1c**

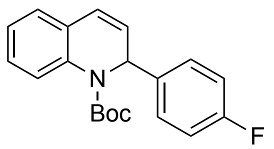

1c

n-BuLi (10 mL, 24 mmol, 2.4 M in hexane) was added to 1-bromo-4-fluorobenzene (2.6 mL, 24 mmol) in dry THF (25 mL) at −78 °C. After 1 h, the mixture was warmed to 0 °C and quinoline (2.4 mL, 20 mmol) in dry THF (20 mL) was added. After 30 min, Boc_2O (6.5 g, 30 mmol) in dry THF (20 mL) was added. The mixture was allowed to warm to room temperature over 16 h. Water (50 mL) was added and the mixture was extracted with Et_2O (100 mL), dried ($MgSO_4$) and the solvent was evaporated. Purification by column chromatography on silica gel, eluting with petrol−Et_2O (96:4), gave the carbamate **1c** (4.3 g, 66%) as an amorphous solid; m.p. 76–78 °C [petrol–EtOAc]; R_f 0.3 [petrol−Et_2O (9:1)]; ν_{max}/cm^{-1} (film) 3074, 3043, 2977, 2929, 1692, 1602, 1504, 1488, 1454, 1367, 1329, 1305, 1272, 1253, 1158, 1124, 1020, 875, 837; ^{1}H NMR (400 MHz, $CDCl_3$) δ = 7.51 (1H, br d, *J* = 7.5 Hz, ArH), 7.30–7.22 (2H, m, ArH), 7.22–7.10 (2H, m, ArH), 7.09–7.02 (1H, m, ArH), 6.97–6.88 (2H, m, ArH), 6.68 (1H, d, *J* = 9.0 Hz, CH), 6.23–6.07 (2H, m, 2 × CH), 1.57 (9H, s, *t*-Bu); ^{13}C NMR (100 MHz, $CDCl_3$) δ = 162.3 (d, *J* = 246.0 Hz), 153.4, 135.8, 135.0, 128.8 (d, *J* = 8.0 Hz), 128.0, 127.6, 126.9, 126.3, 125.6, 124.7, 123.9, 115.3 (d, *J* = 21.5 Hz), 81.7, 54.5, 28.4; ^{19}F NMR (377 MHz, $CDCl_3$) δ −114.78; HRMS (ES) found: MNa^+ 348.1361. $C_{20}H_{20}FNO_2Na$ requires MNa^+, 348.1370; LRMS (ES) 348 (35%), 270 (100%). The enantiomers were resolved using HPLC (Cellulose–2, n-hexane–isopropanol = 99:1, flow rate = 1.0 mL/min, l = 254 nm), t_R = 4.3 min and 5.4 min.

(±)-*tert*-Butyl 2-(3-Methoxyphenyl)-2*H*-quinoline-1-carboxylate **1d**

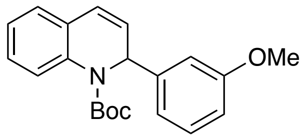

1d

n-BuLi (11 mL, 28 mmol, 2.5 M in hexane) was added to 3-bromoanisole (3.5 mL, 28 mmol) in dry THF (15 mL) at −78 °C. After 1 h, quinoline (2.8 mL, 23 mmol) in dry THF (15 mL) was added at 0 °C. After 1 h, Boc_2O (7.8 g, 35 mmol) in dry THF (5 mL) was added. The mixture was allowed to warm to room temperature over 16 h, then MeOH (5 mL) was added and the solvent was evaporated. Purification by column chromatography on silica gel, eluting with petrol−Et_2O (98:2), gave the carbamate **1d** (4.7 g, 60%) as an oil; R_f 0.65 [petrol−Et_2O (9:1)]; ν_{max}/cm^{-1} (film) 3006, 2973, 1695, 1488, 1159; ^{1}H NMR (400 MHz, $CDCl_3$) δ = 7.55 (1H, d, *J* 6.5 Hz, ArH), 7.17 (2H, m, ArH), 7.13−7.02 (2H, m, ArH), 6.90−6.75 (3H, m, ArH), 6.63 (1H, d, *J* 9.5 Hz, CH), 6.24−6.09 (2H, m, 2 x CH), 3.73 (3H, s, CH_3), 1.57 (9H, s, ^{t}Bu); ^{13}C NMR (100 MHz, $CDCl_3$) δ = 159.6, 153.5, 141.8, 135.3, 129.5, 128.2, 127.5, 127.0, 126.3, 125.4, 124.7,

123.8, 119.3, 112.9, 112.8, 81.6, 55.2 (2 x C), 28.4; HRMS (ES) found MNa^+, 360.1569. $C_{21}H_{23}NO_3Na$ requires MNa^+, 360.1570; LRMS (ES) 360 (26%), 282 (100%), 238 (9%). The enantiomers were resolved using HPLC (Cellulose–1, n-hexane–isopropanol = 99:1, flow rate = 1.0 mL/min, l = 254 nm), t_R = 7.6 min and 8.9 min.

(±)-*tert*-Butyl 4-Methyl-2-phenyl-2*H*-quinoline-1-carboxylate **1e**

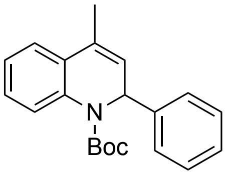

1e

n-BuLi (13 mL, 31 mmol, 2.4 M in hexane) was added to bromobenzene (3.3 mL, 31 mmol) in dry THF (15 mL) at −78 °C. After 1 h, 4-methylquinoline (2.8 mL, 21 mmol) in dry THF (10 mL) was added at 0 °C. After 1 h, Boc_2O (7.6 g, 35 mmol) in dry THF (5 mL) was added, the mixture was allowed to warm to room temperature over 16 h, then MeOH (5 mL) was added and the solvent was evaporated. Purification by column chromatography on silica gel, eluting with petrol−Et_2O (98:2), gave the carbamate **1e** (4.2 g, 62%) as an amorphous solid; m.p. 92–94 °C; R_f 0.52 [petrol−Et_2O (9:1)]; ν_{max}/cm^{-1} (film) 3064, 3036, 2976, 2930, 1689, 1487, 1450, 1366, 1324, 1154, 1145, 1065, 876, 772, 757, 701, 501; ^1H NMR (400 MHz, $CDCl_3$) δ = 7.55 (1H, d, *J* 7.5 Hz, ArH), 7.30 (1H, dd, *J* 7.5, 1 Hz, ArH), 7.27–7.16 (6H, m, ArH), 7.09 (1H, td, *J* 7.5, 1 Hz, ArH), 6.12–6.03 (2H, m, 2 x CH), 2.19 (3H, s, CH_3), 1.58 (9H, s, ^tBu); ^{13}C NMR (100 MHz, $CDCl_3$) δ = 153.5, 140.6, 135.2, 130.5, 128.8, 128.4, 127.4, 127.3, 127.0, 125.1, 124.9, 123.7, 123.2, 81.4, 54.9, 28.4, 18.6; HRMS (ES) found MNa^+, 344.1634. $C_{21}H_{23}NO_2Na$ requires MNa^+, 344.1621; LRMS (ES) 266 (100%), 344 (37%), 222 (11%). The enantiomers were resolved using HPLC (Cellulose–2, n-hexane–isopropanol = 99:1, flow rate = 1.0 mL/min, l = 254 nm), t_R = 4.7 min and 5.3 min.

(±)-*tert*-Butyl 6-Chloro-2-phenyl-2*H*-quinoline-1-carboxylate **1f**

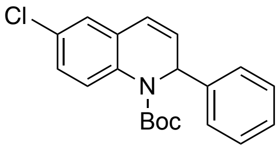

1f

n-BuLi (7.4 mL, 19 mmol, 2.5 M in hexane) was added to bromobenzene (2 mL, 19 mmol) in THF (20 mL) at −78 °C. After 1 h, 6-chloroquinoline (2.5 g, 16 mmol) in THF (20 mL) was added at 0 °C. After 1 h, Boc_2O (5.0 g, 23 mmol) in dry THF (10 mL) was added and the mixture was allowed to warm to room temperature over 16 h. The reaction was quenched with MeOH (10 mL) and the solvent was evaporated. Purification by column chromatography on silica gel, eluting with petrol−EtOAc (95:5),

gave the carbamate **1f** (2.3 g, 43%) as an amorphous solid; m.p. 112–114 °C [petrol−EtOAc]; R_f 0.32 [petrol−EtOAc (9:1)]; ν_{max}/cm^{-1} 2977, 2934, 1695, 1481, 1455, 1368, 1319, 1301, 1252, 1156, 1136, 1089, 1020, 876, 814; ^{1}H NMR (400 MHz, $CDCl_3$) δ = 7.50 (1H, br d, *J* = 7.5 Hz, CH), 7.31–7.22 (5H, m, 5 × CH), 7.16–7.08 (2H, m, 2 × CH), 6.60 (1H, d, *J* = 9.5 Hz, CH), 6.25 (1H, dd, *J* = 9.5, 6.0 Hz, CH), 6.15 (1H, d, *J* = 6.0 Hz, CH), 1.57 (9H, s, *t*-Bu); ^{13}C NMR (100 MHz, $CDCl_3$, one C missing) δ = 153.2, 139.6, 133.7, 129.7, 128.8, 128.6, 127.9, 127.3, 127.0, 126.0, 125.8, 124.4, 82.0, 55.4, 28.4; HRMS *m/z* (ES) Found: MNa^+ 364.1079. $C_{20}H_{20}{}^{35}ClNO_2Na$ requires MNa^+ 364.1075; MNa^+, 366.1054. $C_{20}H_{20}{}^{37}ClNO_2Na$ requires MNa^+ 366.1045; LRMS *m/z* (ES) 364 (100%), 366 (35%). The enantiomers were resolved using HPLC (Cellulose–1, n-hexane–isopropanol = 99.3:0.7, flow rate = 0.7 mL/min, l = 254 nm), t_R = 8.2 min and 9.3 min.

(±)-*tert*-Butyl 6-Fluoro-2-(4-methylphenyl)-2*H*-quinoline-1-carboxylate **1g**

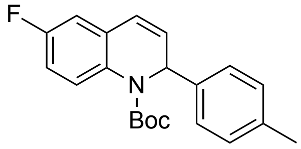

1g

n-BuLi (22 mL, 52 mmol, 2.4 M in hexane) was added to 4-bromotoluene (6.4 mL, 52 mmol) in THF (20 mL) at −78 °C. After 1 h, 6-fluoroquinoline (4.2 mL, 35 mmol) in THF (20 mL) was added at 0 °C. After 1 h, Boc_2O (11 g, 52 mmol) in dry THF (5 mL) was added and the mixture was allowed to warm to room temperature over 16 h. The reaction was quenched with MeOH (10 mL) and the solvent was evaporated. Purification by column chromatography on silica gel, eluting with petrol−EtOAc (96:4), gave the carbamate **1g** (5.5 g, 47%) as needles; m.p. 64–66 °C [petrol−EtOAc]; R_f 0.6 [petrol−EtOAc (9:1)]; ν_{max}/cm^{-1} 3053, 3005, 2981, 2930, 1698, 1493, 1383, 1368, 1307, 1251, 1157, 1122, 1107, 1018, 872, 813, 785, 723, 697, 552, 484; ^{1}H NMR (400 MHz, $CDCl_3$) δ = 7.49 (1H, br, CH), 7.15 (2H, d, *J* 8 Hz, 2 x CH), 7.07 (2H, d, *J* 8 Hz, 2 x CH), 6.87 (1H, dd, *J* 8, 3 Hz, CH), 6.83 (1H, dd, *J* 8, 3 Hz, CH), 6.61 (1H, d, *J* 9.5 Hz, CH), 6.25 (1H, dd, *J* 9.5, 6 Hz, CH), 6.13 (1H, d, *J* 6 Hz, CH), 2.30 (3H, s, CH_3), 1.57 (9H, s, *t*-Bu); ^{13}C NMR (100 MHz, $CDCl_3$) δ = 159.0 (d, *J* 242.5 Hz), 153.4, 137.6, 136.5, 131.0 (d, *J* 2.5 Hz), 130.0, 129.2, 128.7 (d, *J* 9.5 Hz), 127.0, 126.4 (d, *J* 6.5 Hz), 124.6, 113.9 (d, *J* 22.5 Hz), 112.3 (d, *J* 23 Hz), 81.7, 55.0, 28.4, 21.1; ^{19}F NMR (376 MHz, $CDCl_3$) δ = −119.4 (1F, s); HRMS *m/z* (ES) Found: MNa^+ 362.1536. $C_{21}H_{22}FNO_2Na$ requires MNa^+, 362.1527; LRMS *m/z* (ES) 284 (100%), 362 (70%). The enantiomers were resolved using HPLC (Cellulose–2, n-hexane–isopropanol = 99:1, flow rate = 1.0 mL/min, l = 254 nm), t_R = 4.3 min and 5.5 min.

(±)-*tert*-Butyl 2-(2-Methylphenyl)-2*H*-quinoline-1-carboxylate **1h**

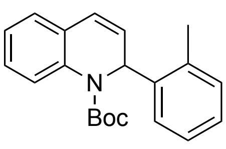

1h

n-BuLi (11 mL, 24 mmol, 2.3 M in hexane) was added to 2-bromotoluene (2.9 mL, 24 mmol) in dry THF (25 mL) at −78 °C. After 1 h, the mixture was warmed to 0 °C and quinoline (2.4 mL, 20 mmol) in dry THF (20 mL) was added. After 30 min, Boc_2O (6.5 g, 30 mmol) in dry THF (20 mL) was added. The mixture was allowed to warm to room temperature over 16 h. MeOH (10 mL) was added and the solvent was evaporated to give a residue that was passed through a silica plug, eluting with petrol−EtOAc (19:1). The solvent was evaporated and the residue was dissolved in EtOH (80 mL) and imidazole (1.4 g, 20 mmol) was added (to remove excess Boc_2O). The mixture was stirred for 1 h and the solvent was evaporated. Aqueous HCl (100 mL, 1 M) was added and the mixture was extracted with Et_2O (150 mL), dried ($MgSO_4$) and the solvent was evaporated. Purification by column chromatography on silica gel, eluting with petrol−Et_2O (95:5), gave the carbamate **1h** (1.2 g, 19%) as an oil; R_f 0.4 [petrol−Et_2O (9:1)]; ν_{max}/cm^{-1} (film) 3062, 3031, 2977, 2934, 2870, 1694, 1602, 1573, 1490, 1454, 1393, 1364, 1317, 1272, 1250, 1155, 1124, 1046, 1020, 991, 873, 842; ^1H NMR (400 MHz, $CDCl_3$) δ = 7.65 (1H, d, *J* = 8.0 Hz, CH), 7.26–7.20 (1H, m, CH), 7.18–7.04 (5H, m, 5 × CH), 7.01–6.95 (1H, m, CH), 6.54 (1H, d, *J* = 9.5 Hz, CH), 6.33 (1H, d, *J* = 6.0 Hz, CH), 6.13 (1H, dd, *J* = 9.5, 6.0 Hz, CH), 2.56 (3H, s, CH_3), 1.51 (9H, s, *t*-Bu); ^{13}C NMR (100 MHz, $CDCl_3$) δ = 153.6, 139.5, 136.2, 134.1, 130.5, 128.2, 127.6, 127.5, 127.0, 126.4, 126.35, 126.3, 124.7, 124.3, 123.9, 81.5, 53.5, 28.3, 19.6; HRMS (ES) found: MNa^+ 344.1623. $C_{21}H_{23}NO_2Na$ requires MNa^+ 344.1621; LRMS (ES) 344 (50%), 266 (70%), 174 (100%). The enantiomers were resolved using HPLC (Cellulose–1, n-hexane–isopropanol = 99:1, flow rate = 1.0 mL/min, l = 254 nm), t_R = 5.9 min and 7.5 min.

(±)-*tert*-Butyl 2-(3,5-Dimethylphenyl)-2*H*-quinoline-1-carboxylate **1i**

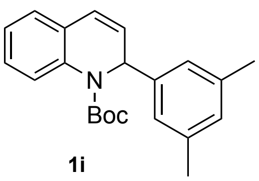

1i

n-BuLi (5.2 mL, 12 mmol, 2.3 M in hexane) was added to 1-bromo-3,5-dimethylbenzene (1.6 mL, 12 mmol) in dry THF (12 mL) at −78 °C. After 1 h, the mixture was warmed to 0 °C and quinoline (1.2 mL, 10 mmol) in dry THF (10 mL) was added. After 30 min, Boc_2O (3.3 g, 15 mmol) in dry THF (10 mL) was added. The mixture was allowed to warm to room temperature over 16 h. Water (50 mL) was added and the mixture was extracted with Et_2O (100 mL), dried ($MgSO_4$) and the solvent was

evaporated. Purification by column chromatography on silica gel, eluting with petrol−Et_2O (95:5), gave the carbamate **1i** (1.2 g, 36%) as an amorphous solid; m.p. 118–120 °C [petrol–EtOAc]; R_f 0.4 [petrol−Et_2O (9:1)]; ν_{max}/cm^{-1} (film) 3041, 3003, 2974, 2917, 2867, 2065, 1910, 1796, 1692 (C=O), 1602, 1570, 1485, 1457, 1390, 1364, 133,. 1305, 1272, 1250, 1157, 1121, 1022, 932, 849; ^1H NMR (400 MHz, $CDCl_3$) δ = 7.57 (1H, d, *J* = 7.5 Hz, CH), 7.22–7.15 (1H, m, CH), 7.14–7.10 (1H, m, CH), 7.07–7.00 (1H, m, CH), 6.92–6.85 (3H, m, 3 × CH), 6.64 (1H, d, *J* = 9.5 Hz, CH), 6.22–6.14 (1H, m, CH), 6.12–6.05 (1H, m, CH), 2.25 (6H, s, 2 × CH_3), 1.58 (9H, s, *t*-Bu); ^{13}C NMR (100 MHz, $CDCl_3$) δ = 153.6, 140.1, 137.9, 135.3, 129.3, 128.5, 127.4, 127.1, 126.2, 125.1, 124.8, 123.7, 81.5, 55.3, 28.4, 21.4; HRMS (ES) found: MNa^+ 358.1790. $C_{22}H_{25}NO_2Na$ requires MNa^+, 358.1778; LRMS (ES) 358 (35%), 280 (100%). The enantiomers were resolved using HPLC (Cellulose–1, n-hexane–isopropanol = 99:1, flow rate = 1.0 mL/min, l = 254 nm), t_R = 5.2 min and 5.9 min.

2.2 Kinetic resolution of racemic substrates **1**

Optimization studies:

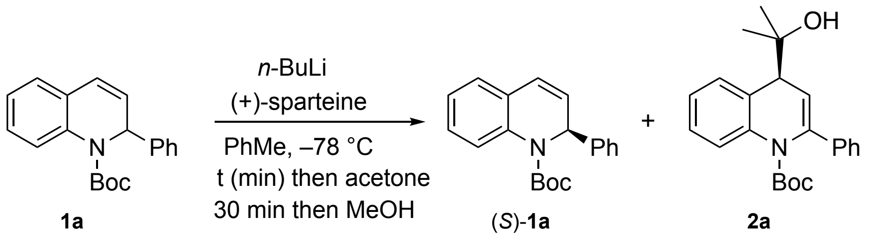

Entry	t (min)	eq. *n*-BuLi	eq. (+)-sp	Yield **1a** (%)	er **1a**	Yield **2a** (%)	er **2a**	s
1	60	0.9	1.0	16	99:1	78	54:46	5
2	60	0.8	0.8	10	94:6	83	51:49	3
3	30	0.6	0.8	45	99:1	50	77:23	41
4[a]	30	0.6	0.8	42	99:1	55	65:35	26
5[a]	30	0.6	0.8	45	98:2	50	79:21	32

[a]Repeat of entry 3.

Table S1. *Optimization of the kinetic resolution of carbamate **1a**.*

tert-Butyl (2*S*)-2-Phenyl-2*H*-quinoline-1-carboxylate (*S*)-**1a** and *tert*-Butyl 4-(2-hydroxypropan-2-yl)-2-phenyl-4*H*-quinoline-1-carboxylate **2a**

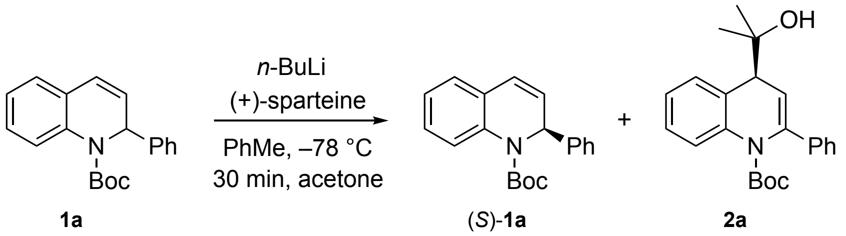

n-BuLi (0.27 mL, 0.62 mmol, 2.3 M in hexanes) was added to a mixture of (+)-sparteine (209 mg, 0.89 mmol) and the racemic carbamate **1a** (319 mg, 1.0 mmol) in dry PhMe (24 mL) at –78 °C. After 30 min, acetone (0.15 mL, 2.1 mmol) was added. After 30 min, MeOH (2 mL) was added and the mixture was allowed to warm to room temperature. The solvent was evaporated, and the residue was purified by column chromatography on silica gel, eluting with petrol–EtOAc (88:12), to give recovered carbamate (*S*)-**1a** (150 mg, 45%) as an amorphous solid; m.p. 49–51 °C; data as above (see page S-3); the enantiomeric ratio was determined to be 99:1 by CSP-HPLC as described above (major component eluted at 5.4 min); $[\alpha]_D^{23}$ –609 (1.2, $CHCl_3$).

In addition, the carbamate **2a** (180 mg, 50%) was isolated as an amorphous solid; m.p. 109−112 °C; R_f 0.38 [petrol−EtOAc (6:4)]; ν_{max}/cm^{-1} (film) 3058, 2975, 1713, 1602, 1582, 1266, 1162; ^{1}H NMR (400 MHz, $CDCl_3$) δ = 7.94 (1H, d, *J* 8 Hz, ArH), 7.52−7.18 (8H, m, ArH), 5.85 (1H, d, *J* 7.5 Hz, CH), 3.55 (1H, d, *J* 7.5 Hz, CH), 1.73 (1H, s, OH), 1.37 (3H, s, CH_3), 1.21 (3H, s, CH_3), 1.14 (9H, s, ^{t}Bu); ^{13}C NMR (100 MHz, $CDCl_3$) δ = 152.4, 142.5, 139.8, 139.2, 131.7, 129.4, 128.1, 127.6, 126.6, 125.3, 124.9, 124.8, 116.9, 81.6, 74.9, 51.6, 27.6, 27.0, 25.9; HRMS (ES) found MNa^+, 388.1890.

$C_{23}H_{27}NO_3Na$ requires MNa^+, 388.1883; LRMS (ES) found 266 (100%), 388 (80%), 292 (30%); the enantiomeric ratio was determined to be 77:23 by CSP-HPLC (major component eluted at 20.8 min); $[\alpha]_D^{23}$ –41 (0.3, $CHCl_3$). The enantiomers were resolved using HPLC (Cellulose–1, n-hexane–isopropanol = 99:1, flow rate = 1.0 mL/min, l = 254 nm), t_R = 19.2 min and 22.5 min.

tert-Butyl (2*R*)-2-Phenyl-2*H*-quinoline-1-carboxylate (*R*)-**1a** and *tert*-Butyl 4-(2-hydroxypropan-2-yl)-2-phenyl-4*H*-quinoline-1-carboxylate **2a**

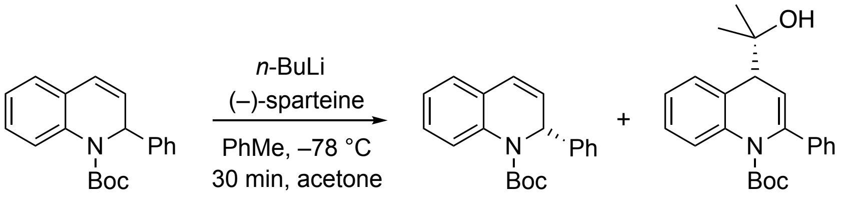

1a (*R*)-**1a** **2a**

n-BuLi (0.25 mL, 0.58 mmol, 2.3 M in hexanes) was added to a mixture of (–)-sparteine (200 mg, 0.85 mmol) and the racemic carbamate **1a** (300 mg, 0.97 mmol) in dry PhMe (24 mL) at –78 °C. After 30 min, acetone (0.14 mL, 1.9 mmol) was added. After 30 min, MeOH (2 mL) was added and the mixture was allowed to warm to room temperature. The solvent was evaporated, and the residue was purified by column chromatography on silica gel, eluting with petrol–EtOAc (88:12), to give recovered carbamate (*R*)-**1a** (140 mg, 47%) as an amorphous solid; m.p. 50–52 °C; data as above (see page S-3); the enantiomeric ratio was determined to be 97:3 by CSP-HPLC as described above (major component eluted at 6.5 min); $[\alpha]_D^{23}$ +652 (1.2, $CHCl_3$).

In addition, the carbamate **2a** (180 mg, 50%) was isolated as an amorphous solid; m.p. 105−107 °C; data as above; the enantiomeric ratio was determined to be 82:18 by CSP-HPLC (major component eluted at 17.7 min); $[\alpha]_D^{23}$ +51 (0.3, $CHCl_3$).

tert-Butyl (2S)-2-(4-Chlorophenyl)-2*H*-quinoline-1-carboxylate (*S*)-**1b** and *tert*-Butyl 2-(4-Chlorophenyl)-4-(2-hydroxypropan-2-yl)-4*H*-quinoline-1-carboxylate **2b**

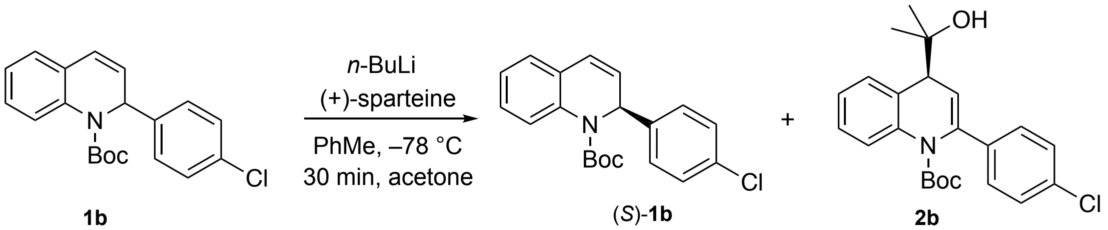

1b (*S*)-**1b** **2b**

n-BuLi (0.27 mL, 0.6 mmol, 2.2 M in hexanes) was added to a mixture of (+)-sparteine (182 mg, 0.86 mmol) and the racemic carbamate **1b** (295 mg, 0.86 mmol) in dry PhMe (24 mL) at –78 °C. After 30 min, acetone (0.13 mL, 1.7 mmol) was added. After 1 h, MeOH (2 mL) was added and the mixture was allowed to warm to room temperature. The solvent was evaporated, and the residue was purified by column chromatography on silica gel, eluting with petrol–EtOAc (88:12), to give recovered carbamate (*S*)-**1b** (130 mg, 43%) as an amorphous solid; m.p. 67–69 °C; data as above (see page S-3); the enantiomeric ratio was determined to be 99:1 by CSP-HPLC as described above (major component eluted at 6.8 min); $[\alpha]_D^{23}$ –216 (1.0, $CHCl_3$).

In addition, the carbamate **2b** (200 mg, 58%) was isolated as an oil; R_f 0.29 [petrol−EtOAc (9:1)]; ν_{max}/cm^{-1} (film) 3438, 2973, 1712, 1459, 1159, 825; ^1H NMR (400 MHz, $CDCl_3$) δ = 7.87 (1H, d, *J* 8 Hz, ArH), 7.44−7.28 (5H, m, ArH), 7.24−7.13 (2H, m, ArH), 5.83 (1H, d, *J* 7.5 Hz, CH), 3.51 (1H, d, *J* 7.5 Hz, CH), 2.17 (1H, s, OH), 1.31 (3H, s, CH_3), 1.25 (3H, s, CH_3), 1.16 (9H, s, ^{t}Bu); ^{13}C NMR (100 MHz, $CDCl_3$) δ =152.3, 141.3, 139.6, 137.8, 133.1, 131.8, 129.5, 128.3, 126.6, 126.5, 125.0, 124.9, 117.9, 81.8, 74.8, 51.6, 27.7, 27.0, 26.0; HRMS (ES) found MNa^+, 422.1500. $C_{23}H_{26}NO_3{}^{35}ClNa$ requires MNa^+, 422.1493; Found MNa^+, 424.1474. $C_{23}H_{26}NO_3{}^{37}ClNa$ requires MNa^+, 424.1469; LRMS (ES) 422 (100%), 424 (36%); the enantiomeric ratio was determined to be 73:27 by CSP-HPLC (major component eluted at 29.8 min); $[\alpha]_D^{23}$ –54 (0.4, $CHCl_3$). The enantiomers were resolved using HPLC (Cellulose–2, n-hexane–isopropanol = 99:1, flow rate = 1.0 mL/min, l = 254 nm), t_R = 21.3 min and 26.7 min.

tert-Butyl (2S)-2-(4-Fluorophenyl)-2*H*-quinoline-1-carboxylate (*S*)-**1c** and *tert*-Butyl 2-(4-Fluorophenyl)-4-(2-hydroxypropan-2-yl)-4*H*-quinoline-1-carboxylate **2c**

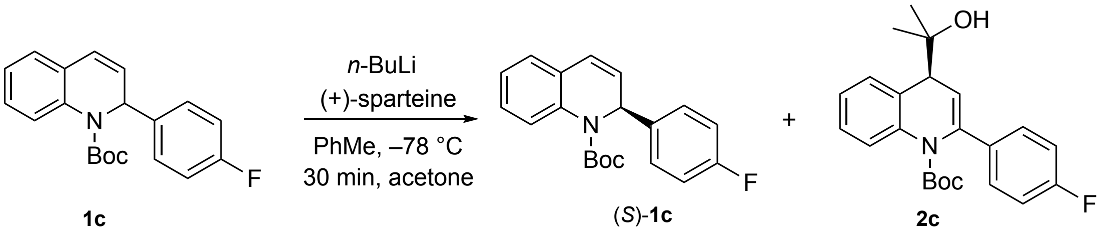

n-BuLi (0.12 mL, 0.28 mmol, 2.3 M in hexanes) was added to a mixture of (+)-sparteine (75 mg, 0.32 mmol) and the racemic carbamate **1c** (130 mg, 0.40 mmol) in dry PhMe (10 mL) at –78 °C. After 30 min, acetone (0.2 mL, 2.8 mmol) was added. After 30 min, MeOH (2 mL) was added and the mixture was allowed to warm to room temperature. The solvent was evaporated, and the residue was purified by column chromatography on silica gel, eluting with petrol–EtOAc (7:3), to give recovered carbamate (*S*)-**1c** (56 mg, 43%) as an oil; data as above (see page S-4); the enantiomeric ratio was determined to be 95:5 by CSP-HPLC as described above (major component eluted at 5.4 min); $[\alpha]_D^{24}$ –404 (1.1, $CHCl_3$).

In addition, the carbamate **2c** (84 mg, 55%) was isolated as an oil; R_f 0.31 [petrol−EtOAc (7:3)]; FT-IR ν_{max} (film)/cm^{-1} 3437, 3072, 3043, 2974, 2934, 2027, 1716, 1654, 1606, 1509, 1488, 1459, 1357, 1312, 1269, 1229, 1162, 1136, 1019, 832, 808; ^{1}H NMR (400 MHz, $CDCl_3$) δ = 7.90 (1H, d, *J* = 8.0 Hz, CH), 7.47–7.41 (2H, m, 2 × CH), 7.40–7.34 (1H, m, CH), 7.25–7.20 (2H, m, 2 × CH), 7.10–7.01 (2H, m, 2 × CH), 5.81 (1H, d, *J* = 7.5 Hz, CH), 3.54 (1H, d, *J* = 7.5 Hz, CH), 1.68 (1H, s, OH), 1.36 (3H, s, CH_3), 1.20 (3H, s, CH_3), 1.18 (9H, s, *t*-Bu); ^{13}C NMR (100 MHz, $CDCl_3$) δ = 162.3 (d, *J* = 246.5 Hz), 152.4, 141.5, 139.7, 135.4, 131.7, 129.4, 126.9 (d, *J* = 8.0 Hz), 126.6, 125.0, 124.9, 117.0, 115.0 (d, *J* = 21.5 Hz), 81.8, 74.8, 51.6, 27.7, 27.0, 26.0; ^{19}F NMR (377 MHz, $CDCl_3$) δ − 114.7; HRMS *m/z* (ES) Found: MNa^+ 406.1801. $C_{23}H_{26}FNO_3Na$ requires MNa^+ 406.1789; LRMS *m/z* (ES) 406 (100%), 310 (20%), 284 (45%); the enantiomeric ratio was determined to be 77:23 by CSP-HPLC (major component eluted at 20.9 min); $[\alpha]_D^{24}$ –34 (1.0, $CHCl_3$). The enantiomers were resolved using HPLC (Cellulose–1, n-hexane–isopropanol = 99:1, flow rate = 1.0 mL/min, l = 254 nm), t_R = 18.0 min and 21.2 min.

tert-Butyl (2S)-2-(3-Methoxyphenyl)-2*H*-quinoline-1-carboxylate (*S*)-**1d** and *tert*-Butyl 4-(2-Hydroxypropan-2-yl)-2-(3-methoxyphenyl)-4*H*-quinoline-1-carboxylate **2d**

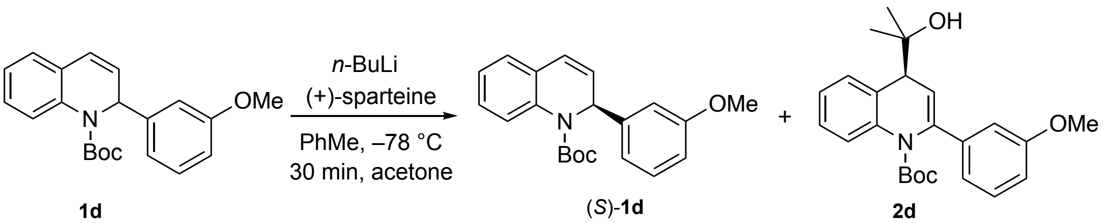

1d (*S*)-**1d** **2d**

n-BuLi (0.32 mL, 0.7 mmol, 2.2 M in hexanes) was added to a mixture of (+)-sparteine (211 mg, 0.90 mmol) and the racemic carbamate **1d** (337 mg, 1.0 mmol) in dry PhMe (24 mL) at –78 °C. After 30 min, acetone (0.15 mL, 2 mmol) was added. After 30 min, MeOH (2 mL) was added and the mixture was allowed to warm to room temperature. The solvent was evaporated, and the residue was purified by column chromatography on silica gel, eluting with petrol–EtOAc (88:12), to give recovered carbamate (*S*)-**1d** (140 mg, 41%) as an oil; data as above (see page S-4); the enantiomeric ratio was determined to be 98:2 by CSP-HPLC as described above (major component eluted at 7.4 min); $[\alpha]_D^{23}$ –440 (0.1, $CHCl_3$).

In addition, the carbamate **2d** (230 mg, 57%) was isolated as an oil; R_f 0.37 [petrol−EtOAc (9:1)]; ν_{max}/cm^{-1} (film) 3463, 3006, 2975, 1711, 1487, 1159; ^{1}H NMR (400 MHz, $CDCl_3$) δ = 7.92 (1H, d, *J* 8 Hz, ArH), 7.37−7.15 (4H, m, ArH), 7.09−6.97 (2H, m, ArH), 6.84 (1H, dd, *J* 8, 1.5 Hz, ArH), 5.85 (1H, d, *J* 7.5 Hz, CH), 3.82 (3H, s, CH_3), 3.52 (1H, d, *J* 7.5 Hz, CH), 2.17 (1H, s, OH), 1.33 (3H, s, CH_3), 1.26 (3H, s, CH_3), 1.16 (9H, s, ^{t}Bu); ^{13}C NMR (100 MHz, $CDCl_3$) δ = 159.5, 152.4, 142.2, 140.7, 139.7, 131.8, 129.5, 129.2, 126.5, 124.9, 124.8, 118.0, 117.2, 112.9, 111.1, 81.6, 74.8, 55.3,

51.6, 27.7, 27.0, 25.9; HRMS (ES) found MNa^+, 418.1997. $C_{24}H_{29}NO_4Na$ requires MNa^+, 418.1989; LRMS (ES) 418 (49%), 296 (100%); the enantiomeric ratio was determined to be 77:23 by CSP-HPLC (major component eluted at 28.4 min); $[\alpha]_D^{23}$ –36 (0.2, $CHCl_3$). The enantiomers were resolved using HPLC (Cellulose–1, n-hexane–isopropanol = 99:1, flow rate = 1.0 mL/min, l = 254 nm), t_R = 26.8 min and 29.1 min.

tert-Butyl (2S)-4-Methyl-2-phenyl-2*H*-quinoline-1-carboxylate (*S*)-**1e** and *tert*-Butyl 4-(2-Hydroxypropan-2-yl)-4-methyl-2-phenyl-4*H*-quinoline-1-carboxylate **2e**

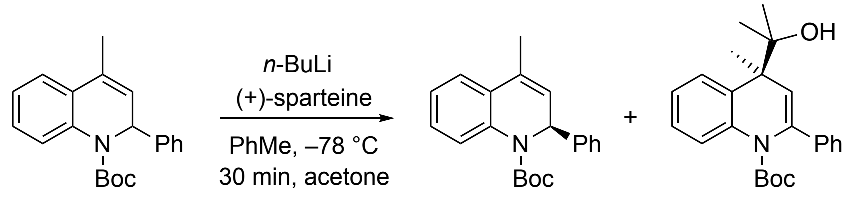

n-BuLi (0.27 mL, 0.6 mmol, 2.2 M in hexanes) was added to a mixture of (+)-sparteine (197 mg, 0.84 mmol) and the racemic carbamate **1e** (319 mg, 0.99 mmol) in dry PhMe (24 mL) at –78 °C. After 30 min, acetone (0.15 mL, 2.0 mmol) was added. After 1 h, MeOH (2 mL) was added and the mixture was allowed to warm to room temperature. The solvent was evaporated, and the residue was purified by column chromatography on silica gel, eluting with petrol–EtOAc (88:12), to give recovered carbamate (*S*)-**1e** (140 mg, 44%) as an amorphous solid; m.p. 82–84 °C; data as above (see page S-5); the enantiomeric ratio was determined to be 99:1 by CSP-HPLC as described above (major component eluted at 5.1 min); $[\alpha]_D^{23}$ –299 (0.4, $CHCl_3$).

In addition, the carbamate **2e** (200 mg, 56%) was isolated as an amorphous solid; m.p. 99–101 °C; R_f 0.28 [petrol−EtOAc (9:1)]; ν_{max}/cm^{-1} (film) 3511, 3064, 3028, 2971, 2933, 1699, 1667, 1485, 1446, 1368, 1349, 1318, 1275, 1159, 1138, 1087, 1018, 844, 754, 698, 637, 586, 497; ^1H NMR (400 MHz, $CDCl_3$) δ = 7.96−7.91 (1H, m, ArH), 7.50−7.45 (2H, m, ArH), 7.41−7.30 (5H, m, ArH), 7.27−7.21 (1H, m, ArH), 5.50 (1H, s, CH), 1.59 (3H, s, CH_3), 1.34 (3H, s, CH_3), 1.22 (3H, s, CH_3), 1.13 (9H, s, ^tBu); ^{13}C NMR (100 MHz, $CDCl_3$) δ =152.5, 140.8, 139.5, 139.4, 136.2, 128.2, 127.5, 127.1, 126.4, 125.3, 124.9, 124.5, 123.9, 81.6, 76.5, 47.3, 27.6, 26.0, 24.7, 24.1; HRMS (ES) found MNa^+, 402.2037. $C_{24}H_{29}NO_3Na$ requires MNa^+, 402.2040; LRMS (ES) 402 (99%), 280 (100%); the enantiomeric ratio was determined to be 82:18 by CSP-HPLC (major component eluted at 14.2 min); $[\alpha]_D^{23}$ –59 (0.8, $CHCl_3$). The enantiomers were resolved using HPLC (Cellulose–1, n-hexane–isopropanol = 99:1, flow rate = 1.0 mL/min, l = 254 nm), t_R = 11.3 min and 16.2 min.

tert-Butyl (2S)-6-Chloro-2-phenyl-2*H*-quinoline-1-carboxylate (*S*)-**1f** and *tert*-Butyl 6-Chloro-4-(2-hydroxypropan-2-yl)-2-phenyl-4*H*-quinoline-1-carboxylate **2f**

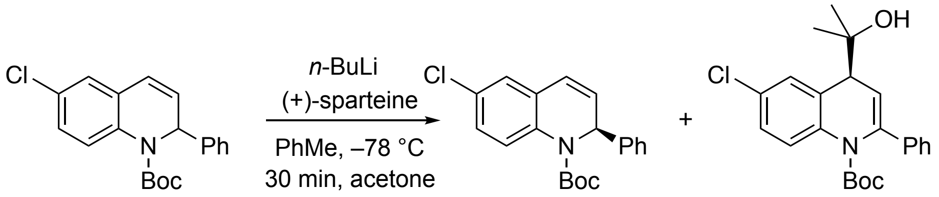

1f (*S*)-**1f** **2f**

n-BuLi (0.12 mL, 0.28 mmol, 2.4 M in hexanes) was added to a mixture of (+)-sparteine (75 mg, 0.32 mmol) and the racemic carbamate **1f** (137 mg, 0.4 mmol) in dry PhMe (10 mL) at –78 °C. After 30 min, acetone (0.2 mL, 2.8 mmol) was added. After 30 min, MeOH (2 mL) was added and the mixture was allowed to warm to room temperature. The solvent was evaporated, and the residue was purified by column chromatography on silica gel, eluting with petrol–EtOAc (87:13), to give recovered carbamate (*S*)-**1f** (61 mg, 45%) as an amorphous solid; m.p. 102–104 °C (petrol–EtOAc); data as above (see page S-5); the enantiomeric ratio was determined to be 90:10 by CSP-HPLC as described above (major component eluted at 9.1 min); $[\alpha]_D^{24}$ –360 (1.5, $CHCl_3$).

In addition, the carbamate **2f** (77 mg, 48%) was isolated as an amorphous solid; m.p. 78–80 °C (petrol–EtOAc); R_f 0.21 [petrol−EtOAc (4:1)]; ν_{max}/cm^{-1} (film) 3480, 2972, 2928, 1716, 1656, 1482, 1369, 1353, 1317, 1267, 1252, 1162, 1134, 1091, 1013, 871, 847; ^1H NMR (400 MHz, $CDCl_3$) δ = 7.87 (1H, d, *J* = 8.5 Hz, CH), 7.46–7.42 (2H, m, 2 × CH), 7.39–7.28 (4H, m, 4 × CH), 7.22 (1H, d, *J* = 2.5 Hz, CH), 5.82 (1H, d, *J* = 7.5 Hz, CH), 3.49 (1H, d, *J* = 7.5 Hz, CH), 1.68 (1H, s, OH), 1.36 (3H, s, CH_3), 1.21 (3H, s, CH_3), 1.13 (9H, s, *t*-Bu); ^{13}C NMR (100 MHz, $CDCl_3$) δ = 152.2, 142.5, 138.8, 138.4, 133.7, 130.2, 129.1, 128.2, 127.8, 126.6, 126.0, 125.3, 116.4, 82.0, 74.8, 51.4, 27.6, 27.1, 25.9; HRMS *m/z* (ES) Found: MNa^+ 422.1507. $C_{23}H_{26}{}^{35}ClNO_3Na$ requires MNa^+ 422.1493; MNa^+, 424.1481. $C_{23}H_{26}{}^{37}ClNO_3Na$ requires MNa^+ 424.1464; LRMS *m/z* (ES) 422 (100%), 424 (35%); the enantiomeric ratio was determined to be 82:18 by CSP-HPLC (major component eluted at 22.2 min); $[\alpha]_D^{24}$ –19 (1.9, $CHCl_3$). The enantiomers were resolved using HPLC (Cellulose–1, n-hexane–isopropanol = 99:1, flow rate = 1.0 mL/min, l = 254 nm), t_R = 19.3 min and 22.2 min.

tert-Butyl (2S)-6-Fluoro-2-(4-methylphenyl)-2*H*-quinoline-1-carboxylate (*S*)-**1g** and *tert*-Butyl 6-Fluoro-4-(2-hydroxypropan-2-yl)-2-(4-methylphenyl)-4*H*-quinoline-1-carboxylate **2g**

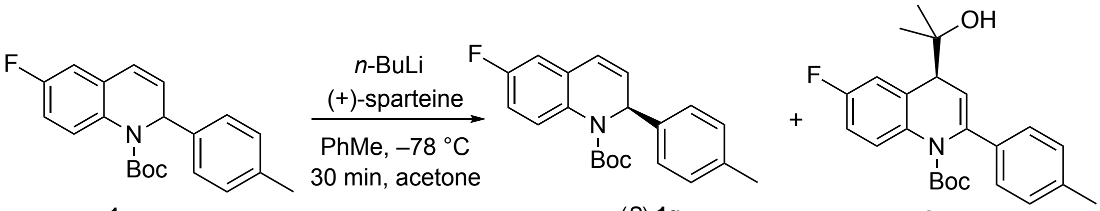

1g (*S*)-**1g** **2g**

n-BuLi (0.26 mL, 0.61 mmol, 2.3 M in hexanes) was added to a mixture of (+)-sparteine (192 mg, 0.82 mmol) and the racemic carbamate **1g** (295 mg, 0.87 mmol) in dry PhMe (24 mL) at –78 °C. After 30 min, acetone (0.13 mL, 1.7 mmol) was added. After 1 h, MeOH (2 mL) was added and the mixture was allowed to warm to room temperature. The solvent was evaporated, and the residue was purified by column chromatography on silica gel, eluting with petrol–EtOAc (88:12), to give recovered carbamate (*S*)-**1g** (130 mg, 43%) as needles; m.p. 62–64 °C; data as above (see page S-6); the enantiomeric ratio was determined to be 96:4 by CSP-HPLC as described above (major component eluted at 5.9 min); $[\alpha]_D^{23}$ –502 (0.3, $CHCl_3$).

In addition, the carbamate **2g** (190 mg, 55%) was isolated as an amorphous solid; m.p. 48–50 °C; R_f 0.2 [petrol−EtOAc (9:1)]; ν_{max}/cm^{-1} (film) 2975, 2933, 1714, 1613, 1495, 1353, 1314, 1243, 1159, 1129, 1026, 849, 812, 764, 740, 489; ^1H NMR (400 MHz, $CDCl_3$) δ = 7.86 (1H, d, *J* 8.5 Hz, CH), 7.34 (2H, d, *J* 8 Hz, 2 x CH), 7.16 (2H, d, *J* 8 Hz, 2 x CH), 7.05 (1H, dd, *J* 8.5, 3 Hz, CH), 6.93 (1H, d, *J* 3 Hz, CH), 5.78 (1H, d, *J* 7.5 Hz, CH), 3.48 (1H, d, *J* 7.5 Hz, CH), 2.39 (3H, s, CH_3), 1.36 (3H, s, CH_3), 1.21 (3H, s, CH_3), 1.15 (9H, s, ^{t}Bu); ^{13}C NMR (100 MHz, $CDCl_3$) δ = 159.8 (d, *J* 244 Hz), 152.5, 142.7, 137.5, 136.0, 135.9 (d, *J* 2.5 Hz), 134.1 (d, *J* 7.5 Hz), 128.8, 126.2 (d, *J* 8.5 Hz), 125.2,115.8, 115.6 (d, *J* 5.5 Hz), 113.3 (d, *J* 22.5 Hz), 81.7, 51.6, 27.6, 27.1, 26.0, 21.2; ^{19}F NMR (376 MHz, $CDCl_3$) δ = −118.4 (1F, s); HRMS *m/z* (ES) Found: MNa^+ 420.1965. $C_{24}H_{28}FNO_3Na$ requires MNa^+, 420.1945; LRMS *m/z* (ES) 420 (100%), 298 (45%); the enantiomeric ratio was determined to be 75:25 by CSP-HPLC (major component eluted at 21.3 min); $[\alpha]_D^{23}$ –33 (0.3, $CHCl_3$). The enantiomers were resolved using HPLC (Cellulose–2, n-hexane–isopropanol = 99:1, flow rate = 1.0 mL/min, l = 254 nm), t_R = 20.4 min and 23.2 min.

tert-Butyl (2S)-2-(2-Methylphenyl)-2*H*-quinoline-1-carboxylate (*S*)-**1h** and *tert*-Butyl 4-(2-Hydroxypropan-2-yl)-2-(2-methylphenyl)-4*H*-quinoline-1-carboxylate **2h**

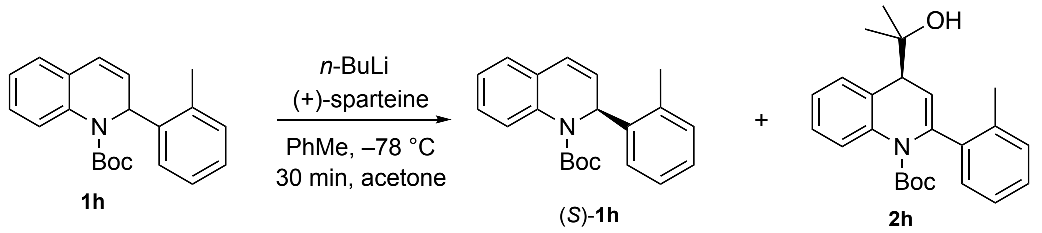

n-BuLi (0.17 mL, 0.40 mmol, 2.3 M in hexanes) was added to a mixture of (+)-sparteine (103 mg, 0.44 mmol) and the racemic carbamate **1h** (129 mg, 0.40 mmol) in dry PhMe (10 mL) at –78 °C. After 4 h, acetone (0.2 mL, 2.8 mmol) was added. After 30 min, MeOH (2 mL) was added and the mixture was allowed to warm to room temperature. The solvent was evaporated, and the residue was purified by column chromatography on silica gel, eluting with petrol–EtOAc (17:3), to give recovered carbamate (*S*)-**1h** (58 mg, 45%) as an oil; data as above (see page S-7); the enantiomeric ratio was determined to be 76:24 by CSP-HPLC as described above (major component eluted at 6.9 min); $[\alpha]_{\mathrm{D}}^{21}$ +349 (1.0, $CHCl_3$).

In addition, the carbamate **2h** (61 mg, 40%) was isolated as an oil; R_f 0.32 [petrol−EtOAc (7:3)]; FT-IR ν_{max} (film)/cm^{-1} 3454, 3064, 2977, 2927, 1711, 1656, 1604, 1585, 1490, 1457, 1350, 1317, 1253, 1165, 1136, 1044, 1015, 965, 918, 842; ^{1}H NMR (400 MHz, $CDCl_3$) δ = 7.94 (1H, d, *J* = 8.0 Hz, CH), 7.41–7.31 (2H, m, 2 × CH), 7.27–7.15 (5H, m, 5 × CH), 5.65 (1H, d, *J* = 7.5 Hz, CH), 3.53 (1H, d, *J* = 7.5 Hz, CH), 2.35 (3H, s, CH_3), 1.71 (1H, br s, OH), 1.40 (3H, s, CH_3), 1.23 (3H, s, CH_3), 1.13 (9H, s, *t*-Bu); ^{13}C NMR (100 MHz, $CDCl_3$) δ = 152.3, 141.7, 139.6, 138.8, 134.8, 131.8, 130.4, 129.4, 128.3, 127.4, 126.6, 125.7, 124.9, 124.5, 119.2, 81.5, 74.9, 51.8, 27.7, 27.2, 25.7, 20.6; HRMS *m/z* (ES) Found: MNa^+ 402.2051. $C_{24}H_{29}NO_3Na$ requires MNa^+ 402.2040; LRMS *m/z* (ES) 402 (65%), 306 (35%), 280 (100%); the enantiomeric ratio was determined to be 71:29 by CSP-HPLC (major component eluted at 18.6 min); $[\alpha]_{\mathrm{D}}^{21}$ +48 (1.0, $CHCl_3$). The enantiomers were resolved using HPLC (Cellulose–1, n-hexane–isopropanol = 99:1, flow rate = 1.0 mL/min, l = 254 nm), t_R = 17.0 min and 20.8 min.

tert-Butyl (2S)-2-(3,5-Dimethylphenyl)-2*H*-quinoline-1-carboxylate (*S*)-**1i** and *tert*-Butyl 4-(2-Hydroxypropan-2-yl)-2-(3,5-dimethylphenyl)-4*H*-quinoline-1-carboxylate **2i**

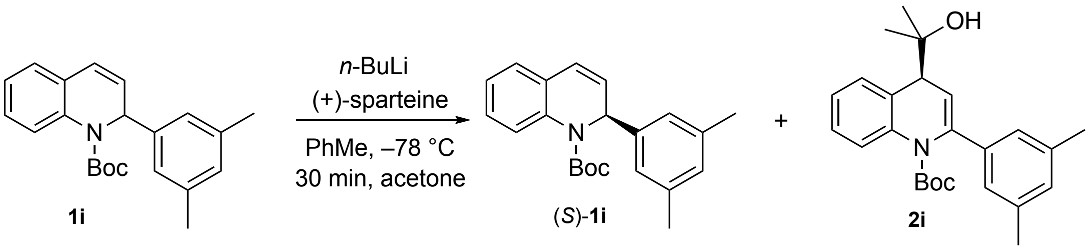

n-BuLi (0.14 mL, 0.32 mmol, 2.3 M in hexanes) was added to a mixture of (+)-sparteine (84 mg, 0.36 mmol) and the racemic carbamate **1i** (134 mg, 0.40 mmol) in dry PhMe (10 mL) at –78 °C. After 30 min, acetone (0.2 mL, 2.8 mmol) was added. After 30 min, MeOH (2 mL) was added and the mixture was allowed to warm to room temperature. The solvent was evaporated, and the residue was purified by column chromatography on silica gel, eluting with petrol–EtOAc (7:3), to give recovered carbamate (*S*)-**1i** (55 mg, 41%) as an oil; data as above (see page S-8); the enantiomeric ratio was determined to be 94:6 by CSP-HPLC as described above (major component eluted at 5.2 min); $[\alpha]_D^{25}$ –502 (1.0, $CHCl_3$).

In addition, the carbamate **2i** (78 mg, 50%) was isolated as an oil; R_f 0.35 [petrol−EtOAc (7:3)]; FT-IR ν_{max} (film)/cm^{-1} 3430, 3074, 3034, 2972, 2922, 2250, 2081, 1708, 1652, 1599, 1485, 1457, 1355, 1314, 1236, 1162, 1134, 1115, 1063, 1032, 908, 856, 837; ^{1}H NMR (400 MHz, $CDCl_3$) δ = 7.98 (1H, br d, *J* = 8.0 Hz, CH), 7.41–7.33 (1H, m, CH), 7.23–7.18 (2H, m, 2 × CH), 7.09–7.04 (2H, m, 2 × CH), 6.95 (1H, br s, CH), 5.82 (1H, d, *J* = 7.5 Hz, CH), 3.53 (1H, d, *J* = 7.5 Hz, CH), 2.34 (6H, s, 2 × CH_3), 1.73 (1H, br s, OH), 1.37 (3H, s, CH_3), 1.21 (3H, s, CH_3), 1.16 (9H, s, *t*-Bu); ^{13}C NMR (100 MHz, $CDCl_3$) δ = 152.4, 142.7, 139.8, 139.1, 137.5, 131.8, 129.3, 129.1, 126.5, 124.8, 124.7, 123.2, 116.2, 81.5, 74.9, 51.6, 27.6, 27.0, 25.9, 21.3; HRMS *m/z* (ES) Found: MNa^+ 416.2214. $C_{25}H_{31}NO_3Na$ requires MNa^+ 416.2196; LRMS *m/z* (ES) 416 (65%), 294 (100%); the enantiomeric ratio was determined to be 80:20 by CSP-HPLC (major component eluted at 25 min); $[\alpha]_D^{25}$ –42 (1.0, $CHCl_3$). The enantiomers were resolved using HPLC (Cellulose–2, n-hexane–isopropanol = 99:1, flow rate = 1.0 mL/min, l = 254 nm), t_R = 19.7 min and 25.3 min.

2.3 Lithiation–trapping of enantioenriched substrates

tert-Butyl (4*R*)-4-(2-hydroxypropan-2-yl)-2-phenyl-4*H*-quinoline-1-carboxylate (*R*)-**2a**

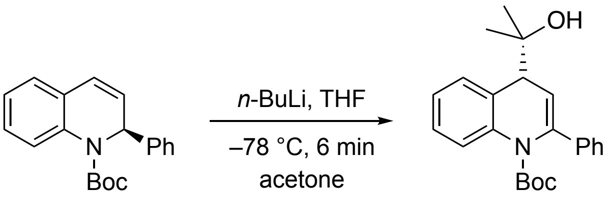

(*S*)-**1a** (*R*)-**2a**

n-BuLi (0.22 mL, 0.55 mmol, 2.5 M in hexane) was added to carbamate (*S*)-**1a** (141 mg, 0.46 mmol, er 99:1) in THF (5 mL) at –78 °C. After 6 min, acetone (0.12 mL, 1.6 mmol) was added. After 30 min, MeOH (2 mL) was added and the mixture was allowed to warm to room temperature. The solvent was evaporated, and the residue was purified by column chromatography on silica gel, eluting with petrol–EtOAc (88:12), to give carbamate (*R*)-**2a** (120 mg, 73%) as an an amorphous solid; m.p. 106–108 °C; data as above (see page S-9); the enantiomeric ratio was determined to be 97:3 by CSP-HPLC (major component eluted at 16.5 min); $[\alpha]_D^{23}$ +84 (0.3, $CHCl_3$).

tert-Butyl (4*R*)-4-Methyl-2-phenyl-4*H*-quinoline-1-carboxylate (*R*)-**3a**

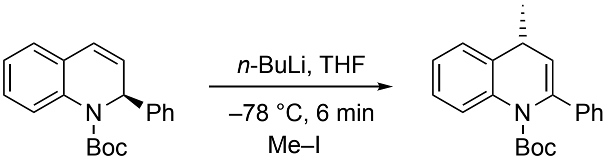

(*S*)-**1a** (*R*)-**3a**

n-BuLi (0.15 mL, 0.36 mmol, 2.4 M in hexane) was added to carbamate (*S*)-**1a** (92 mg, 0.30 mmol, er 99:1) in THF (4 mL) at –78 °C. After 6 min, MeI (0.07 mL, 1.1 mmol) was added. After 30 min, MeOH (2 mL) was added and the mixture was allowed to warm to room temperature. The solvent was evaporated, and the residue was purified by column chromatography on silica gel, eluting with petrol–EtOAc (98:2), to give carbamate (*R*)-**3a** (85 mg, 89%) as needles; m.p. 68−70 °C; R_f 0.47 [petrol−EtOAc (9:1)]; ν_{max}/cm^{-1} 3058, 2974, 1711, 1486, 1349, 1316, 1134, 1269, 752; ^{1}H NMR (400 MHz, $CDCl_3$) δ = 7.90 (1H, d, *J* 8 Hz, ArH), 7.49−7.43 (2H, m, ArH), 7.38−7.26 (4H, m, ArH), 7.24−7.20 (2H, m, ArH), 5.83 (1H, d, *J* 5 Hz, C=CH), 3.57−3.48 (1H, m, CH), 1.44 (3H, d, *J* 7 Hz, CH_3), 1.19 (9H, s, ^{t}Bu); ^{13}C NMR (100 MHz, $CDCl_3$) δ = 152.5, 140.9, 139.1, 139.0, 137.8, 130.1, 128.1, 127.3, 125.7, 125.3, 125.2, 124.2, 122.3, 81.4, 33.3, 27.7, 19.9; HRMS (ES) found MNa^+, 344.1632. $C_{21}H_{23}NO_2Na$ requires MNa^+, 344.1621; LRMS (ES) found 266 (100%), 344 (16%), 222 (8%), 322 (2%); the enantiomeric ratio was determined to be 98:2 by CSP-HPLC (major component eluted at 6.1 min); $[\alpha]_D^{23}$ –79 (0.2, $CHCl_3$). The enantiomers were resolved using HPLC (Cellulose–1, n-hexane–isopropanol = 99:1, flow rate = 1.0 mL/min, l = 254 nm), t_R = 4.8 min and 6.1 min.

tert-Butyl (4*R*)-4-(3,3-Dimethylallyl)-2-phenyl-4*H*-quinoline-1-carboxylate (*R*)-**4a**

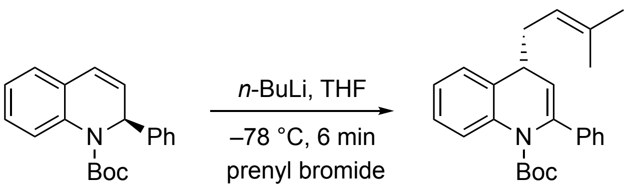

(*S*)-**1a** (*R*)-**4a**

n-BuLi (0.30 mL, 0.75 mmol, 2.5 M in hexane) was added to carbamate (*S*)-**1a** (193 mg, 0.629 mmol, er 99:1) in THF (8 mL) at –78 °C. After 6 min, prenyl bromide (0.25 mL, 2.2 mmol) was added. After 30 min, MeOH (2 mL) was added and the mixture was allowed to warm to room temperature. The solvent was evaporated, and the residue was purified by column chromatography on silica gel, eluting with petrol–EtOAc (97:3), to give carbamate (*R*)-**4a** (148 mg, 63%) which was recrystallised (hexane–CH_2Cl_2) to give (*R*)-**4a** (136 mg, 58%) as needles; m.p. 59–61 °C; R_f 0.62 [petrol−EtOAc (9:1)]; ν_{max}/cm^{-1} 3060, 3031, 2971, 2930, 2861, 1713, 1601, 1447, 1367, 1316, 1272, 1236, 1160, 1135, 1016, 846, 754, 697; ^{1}H NMR (400 MHz, $CDCl_3$) δ = 7.93 (1H, d, *J* 8 Hz, ArH), 7.45−7.41 (2H, m, ArH), 7.38−7.29 (4H, m, ArH), 7.20−7.13 (2H, m, ArH), 5.87 (1H, d, *J* 6.5 Hz, CH), 5.33−5.22 (1H, m, CH), 3.44 (1H, dt, *J* 8.5, 6.5 Hz, CH), 2.49−2.25 (2H, m, CH_2), 1.76 (3H, s, CH_3), 1.56 (3H, s, CH_3), 1.16 (9H, s, ^{t}Bu); ^{13}C NMR (100 MHz, $CDCl_3$) δ = 152.5, 141.1, 139.3, 139.2, 136.2, 133.9, 128.1, 127.3, 126.7, 125.7, 125.3, 124.9, 124.2, 121.4, 120.4, 81.3, 39.9, 34.1, 27.7, 25.9, 17.8; HRMS (ES) found MNa^+, 398.2097. $C_{25}H_{29}NO_2Na$ requires MNa^+, 398.2091; LRMS (ES) found 320 (100%), 398 (50%), 276 (20%); the enantiomeric ratio was determined to be 91:9 prior to recrystallisation and 98:2 after recrystallisation (hexane–CH_2Cl_2) by CSP-HPLC (major component eluted at 4.2 min); $[\alpha]_D^{23}$ +21 (0.3, $CHCl_3$). The enantiomers were resolved using HPLC (Cellulose–1, n-hexane–isopropanol = 99:1, flow rate = 1.0 mL/min, l = 254 nm), t_R = 3.9 min and 4.2 min.

1-*tert*-Butyl (4*R*)-4-Methyl 2-Phenyl-4*H*-quinoline-1,4-dicarboxylate (*R*)-**5a**

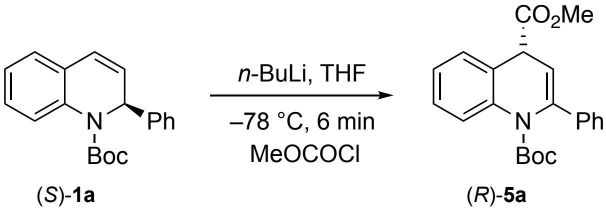

(*S*)-**1a** (*R*)-**5a**

n-BuLi (0.16 mL, 0.4 mmol, 2.5 M in hexane) was added to carbamate (*S*)-**1a** (102 mg, 0.33 mmol, er 99:1) in THF (4 mL) at –78 °C. After 6 min, methyl chloroformate (0.09 mL, 1.0 mmol) was added. After 5 min, MeOH (2 mL) was added and the mixture was allowed to warm to room temperature. The solvent was evaporated, and the residue was purified by column chromatography on silica gel, eluting with petrol–EtOAc (96:4), to give carbamate (*R*)-**5a** (97 mg, 80%) as needles; m.p. 89–91 °C; R_f 0.37 [petrol−EtOAc (9:1)]; ν_{max}/cm^{-1} 2973, 2950, 1732, 1713, 1488, 1354, 1321, 1207, 1137, 1157, 1009, 846, 756, 700, 625, 566; ^{1}H NMR (400 MHz, $CDCl_3$) δ = 7.95 (1H, d, *J* 8

Hz, ArH), 7.53−7.46 (2H, m, ArH), 7.42−7.31 (4H, m, ArH), 7.26−7.19 (2H, m, ArH), 5.90 (1H, d, *J* 6.5 Hz, CH), 4.45 (1H, d, *J* 6.5 Hz, CH), 3.70 (3H, s, CH_3), 1.18 (9H, s, ^{t}Bu); ^{13}C NMR (100 MHz, $CDCl_3$) δ = 171.3, 152.3, 143.4, 139.0, 138.5, 130.3, 128.2, 127.9, 127.2, 127.1, 125.5, 125.3, 124.9, 113.0, 81.6, 52.6, 45.9, 27.7; HRMS (ES) found MNa^+, 388.1537. $C_{22}H_{23}NO_4Na$ requires MNa^+, 388.1519; LRMS (ES) found 388 (100%), 266 (59%); the enantiomeric ratio was determined to be 88:12 by CSP-HPLC (major component eluted at 12.7 min); $[\alpha]_D^{23}$ –30 (0.3, $CHCl_3$). The enantiomers were resolved using HPLC (Cellulose–1, n-hexane–isopropanol = 99:1, flow rate = 1.0 mL/min, l = 254 nm), t_R = 10.4 min and 12.9 min.

tert-Butyl (4*R*)-2-(4-Chlorophenyl)-4-(2-hydroxypropan-2-yl)-4*H*-quinoline-1-carboxylate (*R*)-**2b**

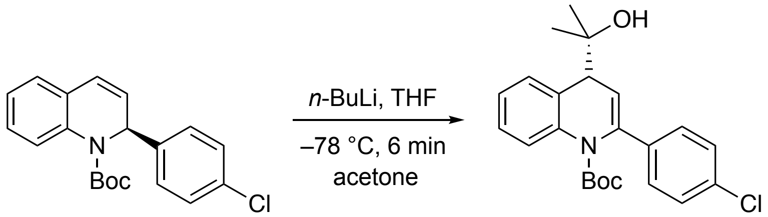

(*S*)-**1b** (*R*)-**2b**

n-BuLi (0.17 mL, 0.37 mmol, 2.2 M in hexane) was added to carbamate (*S*)-**1b** (105 mg, 0.31 mmol, er 99:1) in THF (4 mL) at –78 °C. After 6 min, acetone (0.08 mL, 1.0 mmol) was added. After 30 min, MeOH (2 mL) was added and the mixture was allowed to warm to room temperature. The solvent was evaporated, and the residue was purified by column chromatography on silica gel, eluting with petrol–EtOAc (88:12), to give carbamate (*R*)-**2b** (92 mg, 75%) as an oil; data as above (see page S-9); the enantiomeric ratio was determined to be 96:4 by CSP-HPLC as described above (major component eluted at 22.1 min); $[\alpha]_D^{23}$ +105 (0.4, $CHCl_3$).

tert-Butyl (4*R*)-4-(3,3-Dimethylallyl)-2-(3-methoxyphenyl)-4*H*-quinoline-1-carboxylate (*R*)-**4c**

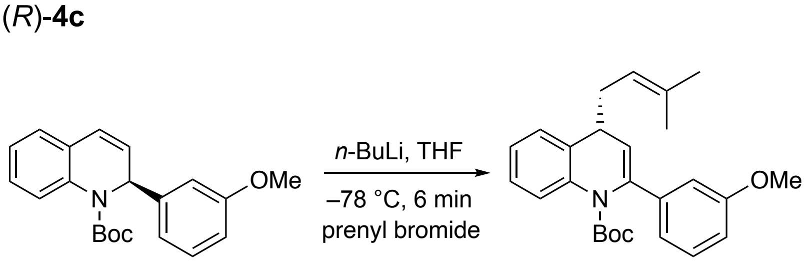

(*S*)-**1c** (*R*)-**4c**

n-BuLi (0.21 mL, 0.47 mmol, 2.2 M in hexane) was added to carbamate (*S*)-**1c** (131 mg, 0.39 mmol, er 98:2) in THF (5 mL) at –78 °C. After 6 min, prenyl bromide (0.16 mL, 1.4 mmol) was added. After 30 min, MeOH (2 mL) was added and the mixture was

allowed to warm to room temperature. The solvent was evaporated, and the residue was purified by column chromatography on silica gel, eluting with petrol–EtOAc (97:3), to give carbamate (*R*)-**4c** (110 mg, 72%) as an oil; R_f 0.77 [petrol−EtOAc (9:1)]; ν_{max}/cm^{-1} (film) 3069, 2975, 2933, 2835, 1712, 1600, 1486, 1313, 1159, 1131, 1044, 849, 770, 755, 697; ^{1}H NMR (400 MHz, $CDCl_3$) δ = 7.93 (1H, d, *J* 8 Hz, ArH), 7.34−7.29 (1H, m, ArH), 7.27−7.23 (1H, m, ArH), 7.20−7.13 (2H, m, ArH), 7.06−7.02 (1H, m, ArH), 7.00−6.97 (1H, m, ArH), 6.85 (1H, ddd, *J* 8, 2.5, 0.5 Hz, ArH), 5.89 (1H, d, *J* 6.5 Hz, CH), 5.31−5.22 (1H, m, CH), 3.84 (3H, s, CH_3), 3.43 (1H, dt, *J* 8, 6.5 Hz, CH), 2.48−2.23 (2H, m, 2 x CH), 1.76 (3H, s, CH_3), 1.56 (3H, s, CH_3), 1.20 (9H, s, ^{t}Bu); ^{13}C NMR (100 MHz, $CDCl_3$) δ = 159.5, 152.5, 140.9, 140.8, 139.2, 136.1, 133.9, 129.1, 126.7, 125.7, 124.9, 124.2, 121.4, 120.5, 118.0, 112.7, 111.1, 81.4, 55.3, 39.9, 34.1, 27.8, 25.9, 17.8; HRMS (ES) found MNa^+, 428.2215. $C_{26}H_{31}NO_3$ requires MNa^+, 428.2196; LRMS (ES) 428 (41%), 350 (100%), 306 (22%); the enantiomeric ratio was determined to be 93:7 by CSP-HPLC (major component eluted at 5.4 min); $[\alpha]_D^{23}$ +124 (0.1, $CHCl_3$). The enantiomers were resolved using HPLC (Cellulose–1, n-hexane–isopropanol = 99:1, flow rate = 1.0 mL/min, l = 254 nm), t_R = 4.5 min and 5.1 min.

1-*tert*-Butyl (4*R*)-4-Methyl 4-Methyl-2-phenyl-4*H*-quinoline-1,4-dicarboxylate (*R*)-**6**

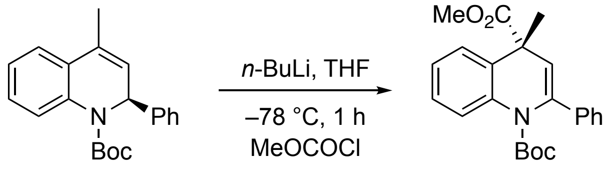

(*S*)-**1e** (*R*)-**6**

n-BuLi (0.18 mL, 0.4 mmol, 2.2 M in hexane) was added to carbamate (*S*)-**1e** (106 mg, 0.33 mmol, er 99:1) in THF (4 mL) at –78 °C. After 1 h, methyl chloroformate (0.09 mL, 1.0 mmol) was added. After 5 min, MeOH (2 mL) was added and the mixture was allowed to warm to room temperature. The solvent was evaporated, and the residue was purified by column chromatography on silica gel, eluting with petrol–EtOAc (96:4), to give carbamate (*R*)-**6** (90 mg, 72%) as an amorphous solid; m.p. 122–124 °C; R_f 0.26 [petrol−EtOAc (9:1)]; ν_{max}/cm^{-1} 3053, 2978, 2950, 2930, 2872, 1728, 1717, 1637, 1488, 1458, 1448, 1430, 1350, 1318, 1271, 1248, 1222, 1155, 1131, 1017, 827, 774, 757, 697, 628; ^{1}H NMR (400 MHz, $CDCl_3$) δ = 7.94 (1H, dd, *J* 8, 0.5 Hz, ArH), 7.52−7.47 (2H, m, ArH), 7.41−7.18 (6H, m, ArH), 5.70 (1H, s, CH), 3.66 (3H, s, CH_3), 1.76 (3H, s, CH_3), 1.17 (9H, s, ^{t}Bu); ^{13}C NMR (100 MHz, $CDCl_3$) δ = 174.0, 152.4, 141.8, 138.6, 138.5, 135.4, 128.2, 127.8, 126.9, 125.5, 125.3, 124.8, 124.4, 120.3, 81.5, 52.8, 46.6, 27.7, 23.7; HRMS (ES) found MNa^+, 402.1683. $C_{23}H_{25}NO_4Na$ requires MNa^+, 402.1676; LRMS (ES) 402 (98%), 280 (100%); the enantiomeric ratio was determined to be 96:4 by CSP-HPLC (major component eluted at 16.3 min); $[\alpha]_D^{23}$ –14 (0.4, $CHCl_3$). The enantiomers were resolved using HPLC (Cellulose–1, n-hexane–isopropanol = 99:1, flow rate = 0.5 mL/min, l = 254 nm), t_R = 14.6 min and 15.9 min. Recrystallization (hexane–CH_2Cl_2) gave the carbamate (*R*)-**6** with er 99:1 as determined by CSP-HPLC as above.

1-*tert*-Butyl (4*S*)-4-Methyl 4-Methyl-2-phenyl-4*H*-quinoline-1,4-dicarboxylate (*S*)-**6**

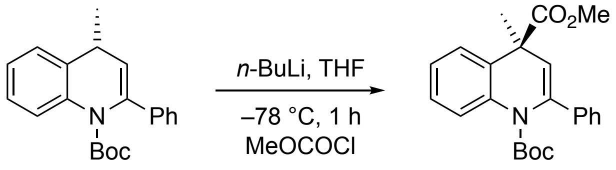

(*R*)-**3a** (*S*)-**6**

n-BuLi (0.27 mL, 0.63 mmol, 2.3 M in hexane) was added to carbamate (*S*)-**3a** (177 mg, 0.55 mmol, er 98:2) in THF (6 mL) at –78 °C. After 1 h, methyl chloroformate (0.26 mL, 1.8 mmol) was added. After 5 min, MeOH (2 mL) was added and the mixture was allowed to warm to room temperature. The solvent was evaporated, and the residue was purified by column chromatography on silica gel, eluting with petrol–EtOAc (96:4), to give carbamate (*S*)-**6** (130 mg, 64%) as an an amorphous solid; m.p. 120–122 °C; data as above; the enantiomeric ratio was determined to be 95:5 by CSP-HPLC as described above (major component eluted at 13.2 min); $[\alpha]_D^{23}$ +23 (0.4, $CHCl_3$).

2.4 Reduction and oxidation of dihydroquinolines

1-*tert*-Butyl (2*R*,4*R*)-4-(2-hydroxypropan-2-yl)-2-phenyl-1,2,3,4-tetrahydroquinoline-1-carboxylate **7**

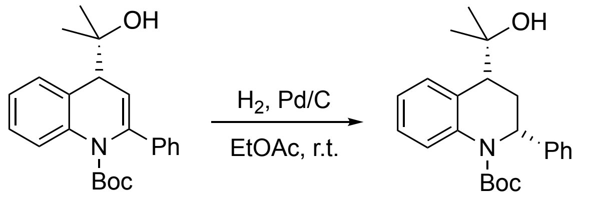

(*R*)-**2a** **7**

The carbamate (*R*)-**2a** (129 mg, 0.35 mmol, er 97:3) and 10% palladium on charcoal (46 mg) in EtOAc (12 mL) was stirred under hydrogen gas (1 atm) at room temperature. After 18 h, the mixture was filtered through celite. The solvent was evaporated to give carbamate **7** (130 mg, 99%) as an an amorphous solid; m.p. 108–110 °C; R_f 0.38 [petrol−EtOAc (3:2)]; ν_{max}/cm^{-1} 3476, 3415, 3033, 2971, 2936, 2872, 1693, 1680, 1486, 1453, 1334, 1143, 1022, 913, 864, 828, 774, 756, 738, 606; ^{1}H NMR (400 MHz, $CDCl_3$) δ = 7.81 (1H, d, *J* 7.5 Hz, ArH), 7.65 (1H, d, *J* 8 Hz, ArH), 7.33−7.28 (2H, m, ArH), 7.28−7.26 (1H, m, ArH), 7.25−7.18 (3H, m, ArH), 7.16−7.11 (1H, m, ArH), 5.25 (1H, dd, *J* 11.5, 8 Hz, CH), 2.78 (1H, br d, *J* 12 Hz, CH), 2.67−2.55 (1H, m, CH), 1.66−1.61 (1H, m, CH), 1.50 (3H, s, CH_3), 1.48 (3H, s, CH_3), 1.38 (9H, s, ^{t}Bu); ^{13}C NMR (100 MHz, $CDCl_3$) δ = 153.9, 145.1, 138.8, 135.8, 128.4, 126.7, 126.4, 125.9, 125.8, 125.6, 123.7, 80.8, 72.4, 59.7, 47.6, 39.8, 28.2; HRMS (ES) found MH^+, 368.2219. $C_{23}H_{30}NO_3$ requires MH^+, 368.2220; LRMS (ES) found 390 (100%), 312 (89%), 268 (78%), 368 (46%); the enantiomeric ratio was determined to be 96:4 by by CSP-HPLC (major component eluted at 30.2 min); $[\alpha]_D^{19}$ +118 (0.4, $CHCl_3$). The enantiomers were resolved using HPLC (ChiralPakIA, n-hexane–isopropanol = 99:1, flow rate = 1.5 mL/min, l = 254 nm), t_R = 27.6 min and 30.5 min.

1-*tert*-Butyl 4-Methyl 4-Methyl-2-phenyl-1,2,3,4-tetrahydroquinoline-1,4-dicarboxylate **8**

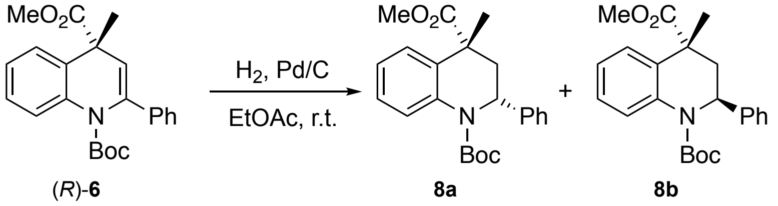

The carbamate (*R*)-**6** (36 mg, 0.1 mmol, er 99:1) and 10% palladium on charcoal (14 mg) in EtOAc (5 mL) was stirred under hydrogen gas (1 atm) at room temperature. After 18 h, the mixture was filtered through celite. The solvent was evaporated to give carbamate **8** (36 mg, 99%) as a mixture of diastereoisomers (dr ~4:1) as determined by CSP-HPLC (four peaks) and ^{1}H NMR spectroscopy. Recrystallisation (hexane–CH_2Cl_2) gave a trace amount of the racemic major diastereoisomer **8a** as an amorphous solid [or this solid could be prepared by recrystallization of (±)-**8**]; data for **8a**: m.p. 136−138 °C; R_f 0.26 [petrol−EtOAc (9:1)]; ν_{max}/cm^{-1} 3028, 3010, 2971, 2949, 2873, 1728, 1698, 1366, 1329, 1231, 1207, 1145, 747, 706, 507; ^{1}H NMR (400 MHz, C_6D_6) δ = 8.05 (1H, d, *J* 8 Hz, ArH), 7.21−7.12 (3H, m, ArH), 7.10−7.04 (3H, m, ArH), 7.03−6.97 (1H, m, ArH), 6.90 (1H, td, *J* 7.5, 1 Hz, ArH), 5.24 (1H, dd, *J* 11.5, 7 Hz, CH), 3.25 (3H, s, CH_3), 2.49 (1H, dd, *J* 13.5, 11.5 Hz, CH), 2.13 (1H, dd, *J* 13.5, 7 Hz, CH), 1.55 (3H, s, CH_3), 1.24 (9H, s, ^{t}Bu); ^{13}C NMR (100 MHz, C_6D_6) δ = 174.5, 153.2, 144.9, 137.1, 135.1, 128.3, 127.0, 126.6, 126.3, 126.0, 124.7, 123.6, 80.4, 56.3, 51.3, 46.2, 43.3, 27.8, 20.5; HRMS (ES) found MNa^+, 404.1851. $C_{23}H_{27}NO_4Na$ requires MNa^+, 404.1832; LRMS (ES) 404 (100%), 282 (31%); the enantiomeric ratio of the major isomer **8a** was determined to be 98:2 by CSP-HPLC (major component eluted at 6.9 min); $[\alpha]_D^{22}$ +136 (0.1, $CHCl_3$). The enantiomers were resolved using HPLC (ChiralPakIA, n-hexane–isopropanol = 99:1, flow rate = 1.0 mL/min, l = 254 nm), t_R = 7.0 min and 10.0 min. The enantiomers of the minor diastereomer **8b** could be resolved using HPLC (Cellulose–1, n-hexane–isopropanol = 99:1, flow rate = 1.0 mL/min, l = 254 nm), t_R = 20.8 min and 24.6 min.

4-Methyl-2-phenylquinoline **9**

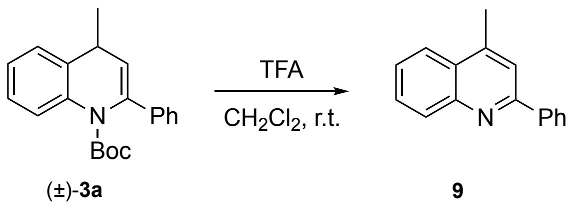

Trifluoroacetic acid (1.1 mL, 14 mmol) was added to the carbamate (±)-**3a** (230 mg, 0.75 mmol) in CH_2Cl_2 (25 mL) at room temperature. After 24 h, the solvent was evaporated and the residue was purified by column chromatography on silica gel, eluting with petrol–EtOAc (9:1), to give quinoline **9** (150 mg, 95%) as an oil; ^{1}H NMR (400 MHz, CD_3OD) δ = 8.45 (1H, d, *J* 8.5 Hz, ArH), 8.34 (1H, d, *J* 8.5 Hz, ArH), 8.25 (1H, s, ArH), 8.18−8.09 (3H, m, ArH), 8.02−7.91 (1H, m, ArH), 7.82−7.67 (3H, m, ArH),

3.07 (3H, s, CH_3); HRMS (ES) found MH^+, 220.1121. $C_{16}H_{14}N$ requires MH^+, 220.1121; data corresponds with the literature.[56]

Alternatively, hydrochloric acid (1.8 mL, 7 mmol, 4 M in dioxane) was added to the carbamate (±)-**3a** (226 mg, 0.70 mmol) at room temperature. After 24 h, the solvent was evaporated and the residue was purified by column chromatography on silica gel, eluting with petrol–EtOAc (9:1), to give quinoline **9** (150 mg, 95%) as an oil.

2.5 Application to M5717

tert-Butyl 6-Fluoro-2-{4-[(morpholin-4-yl)methyl]phenyl)]-2*H*-quinoline-1-carboxylate **11**

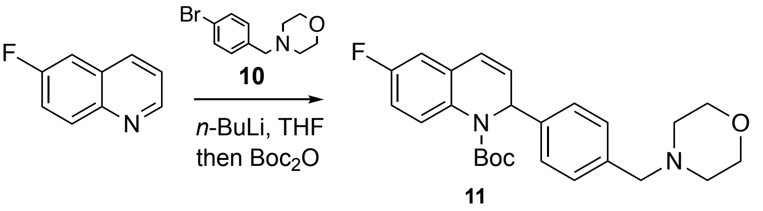

n-BuLi (28 mL, 62 mmol, 2.2 M in hexane) was added to 4-[(4-bromophenyl)methyl]morpholine[57] (17 g, 62 mmol) in dry THF (32 mL) at −78 °C. After 30 min, 6-fluoroquinoline (5.0 mL, 41 mmol) in THF (15 mL) was added at 0 °C. After 1 h, Boc_2O (15 g, 62 mmol) in dry THF (5 mL) was added dropwise and the mixture was allowed to warm to room temperature over 16 h. MeOH (5 mL) was added and the solvent was evaporated. Purification by column chromatography on silica gel, eluting with CH_2Cl_2−MeOH (98:2), gave carbamate **11** (12 g, 67%) as an oil; R_f 0.7 [CH_2Cl_2−MeOH (9:1)]; ν_{max}/cm^{-1} (film) 3007, 2971, 2931, 2855, 2806, 1698, 1490, 1368, 1253, 1160, 1114, 1008, 866, 700; ^1H NMR (400 MHz, $CDCl_3$) δ = 7.54−7.43 (1H, m, ArH), 7.23−7.18 (4H, m, ArH), 6.92−6.81 (2H, m, ArH), 6.61 (1H, d, *J* 9.5 Hz, C=CH), 6.26 (1H, dd, *J* 9.5 and 6.0 Hz, C=CH), 6.15 (1H, d, *J* 6.0 Hz, CH), 3.72−3.67 (4H, m, 2 x CH_2), 3.44 (2H, s, CH_2), 2.48−2.36 (4H, m, 2 x CH_2), 1.56 (9H, s, ^{t}Bu); ^{13}C NMR (100 MHz, $CDCl_3$) δ = 159.0 (d, *J* 242.5 Hz), 153.4, 141.7, 138.4, 131.0 (d, *J* 2.5 Hz), 128.6 (d, *J* 8.5 Hz), 129.6, 129.3, 127.0, 126.3 (d, *J* 2.5 Hz), 124.7, 114.0 (d, *J* 22.5 Hz), 112.3 (d, *J* 23.0 Hz), 81.7), 67.0, 63.0, 54.9, 53.6, 28.4; ^{19}F NMR (376 MHz, $CDCl_3$) δ = −119.3 (1F, s); HRMS (ES) found MH^+, 425.2247. $C_{25}H_{30}FN_2O_3$ requires MH^+, 425.2235; LRMS (ES) found 425 (100%).

1-*tert*-Butoxycarbonyl-6-fluoro-2-{4-[(morpholin-4-yl)methyl]phenyl}-1,4-dihydroquinoline-4-carboxylic acid **12**

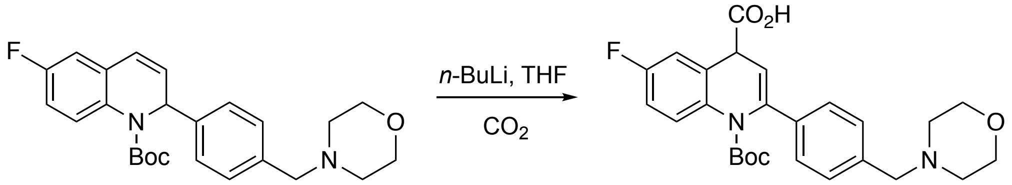

n-BuLi (2.6 mL, 6.5 mmol, 2.5 M in hexane) was added to carbamate **11** (2.3 g, 5.4 mmol) in dry THF (30 mL) at −78 °C. After 6 min, milled dry ice[45] was added and the mixture was warmed to room temperature. After 1 h, the solvent was evaporated. Purification by column chromatography on silica gel, eluting with CH_2Cl_2−MeOH (9:1), gave carbamate **12** (1.97 g, 78%) as an amorphous solid; m.p 104–106 °C; R_f 0.17 [CH_2Cl_2−MeOH (9:1)]; ν_{max}/cm^{-1} (film) 2974, 2935, 2873, 2810, 1710, 1493, 1349, 1316, 1251, 1159, 1128, 1113, 1025, 865, 819, 768, 745, 452; ^{1}H NMR (400 MHz, DMSO-d_6) δ = 7.80 (1H, dd, *J* 8.5 and 5.0 Hz, ArH), 7.39−7.27 (4H, m, ArH), 7.19−7.09 (2H, m, ArH), 6.01 (1H, d, *J* 6.5 Hz, C=CH), 4.35 (1H, d, *J* 6.5 Hz, CH), 3.62−3.52 (4H, m, 2 x CH_2), 3.47 (2H, s, CH_2), 2.42−2.27 (4H, m, 2 x CH_2), 1.06 (9H, s, ^{t}Bu); ^{13}C NMR (100 MHz, DMSO-d_6) δ = 164.6, 159.8 (d, *J* 241.5 Hz), 151.9, 141.3, 137.5, 136.0 (d, *J* 8.0 Hz), 135.8 (d, *J* 2.0 Hz), 135.2, 129.4, 126.1 (d, *J* 8.5 Hz), 125.2, 116.1, 113.8 (d, *J* 22.5 Hz), 112.7 (d, *J* 22.5 Hz), 81.4, 71.8, 66.6, 62.5, 53.5, 27.7; ^{19}F NMR (376 MHz, DMSO-d_6) δ = −120.2 (1F, s); HRMS (ES) found MH^+, 469.2133. $C_{26}H_{30}FN_2O_5$ requires MH^+, 469.2142; LRMS (ES) found 469 (100%).

6-Fluoro-2-{4-[(morpholin-4-yl)methyl]phenyl}-quinoline-4-carboxylic acid **13**

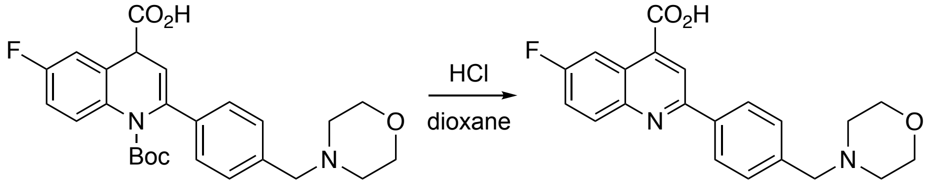

Hydrochloric acid (2.4 mL, 9.6 mmol, 4.0 M in dioxane) was added to the carbamate **12** (431 mg, 0.92 mmol) at room temperature. After 1 day, the solvent was evaporated. Aqueous NaOH (2 M) was added to adjust to ~pH 7–8. The resulting precipitate was filtered and was washed with water (5 mL) and ethyl acetate (5 mL) to give the product **13** (196 mg, 58%) as an amorphous solid; m.p. 130−132 °C; R_f 0.55 [DCM−MeOH (8:2)]; FT-IR ν_{max} (film)/cm^{-1} 3405, 3219, 1575, 1473, 1381, 1353, 1262, 1203, 1124, 969, 917, 869, 793, 740, 674, 533, 492; ^{1}H NMR (400 MHz, DMSO-d_6) δ = 8.57−8.43 (2H, m, ArH), 8.30−8.18 (3H, m, ArH), 7.79 (1H, t, *J* 8.5 Hz, ArH), 7.53 (2H, d, *J* 8.0 Hz, ArH), 3.77−3.49 (6H, m, 3 x CH_2), 2.47−2.43 (4H, m, 2 x CH_2); ^{13}C NMR (100 MHz, DMSO-d_6) δ = 173.4, 160.7 (d, *J* 244.0 Hz), 141.1, 140.8, 138.8 (d, *J* 8.0 Hz), 137.5, 137.0 (d, *J* 3.5 Hz), 135.4, 129.5, 128.8 (d, *J* 8.5 Hz), 127.3, 125.9, 115.8 (d, *J* 23.0 Hz), 115.2 (d, *J* 22.5 Hz), 66.6, 62.4, 53.6; ^{19}F NMR (376 MHz, DMSO-d_6) δ = −111.2 (1F, s); HRMS (ES) found MH^+, 367.1453. $C_{21}H_{20}FN_2O_3$ requires MH^+, 367.1456; LRMS (ES) found 367 (100%, MH^+). Data conforms with the literature.[44,46]

Kinetic resolution of *tert*-butyl 6-fluoro-2-{4-[(morpholin-4-yl)methyl]phenyl)]-2*H*-quinoline-1-carboxylate **11**

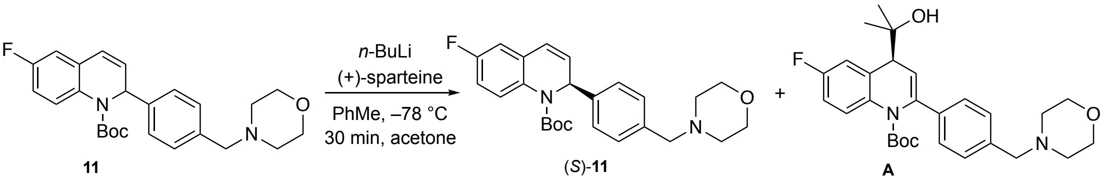

11 (*S*)-**11** **A**

n-BuLi (0.18 mL, 0.46 mmol, 2.5 M in hexanes) was added to a mixture of (+)-sparteine (133 mg, 0.55 mmol) and the racemic carbamate **11** (196 mg, 0.46 mmol) in dry PhMe (16 mL) at –78 °C. After 30 min, acetone (0.07 mL, 0.92 mmol) was added. After 1 h, MeOH (2 mL) was added and the mixture was allowed to warm to room temperature. The solvent was evaporated, and the residue was purified by column chromatography on silica gel, eluting with petrol–EtOAc (40:60), to give recovered carbamate (*S*)-**11** (90 mg, 46%) as an oil; data as above (see page S-26); the enantiomeric ratio was determined to be 84:16 by CSP-HPLC (major component eluted at 9.4 min); $[\alpha]_{\mathrm{D}}^{23}$ –364 (0.4, $CHCl_3$). The enantiomers were resolved using HPLC (Cellulose–2, n-hexane–isopropanol = 95:5, flow rate = 1.0 mL/min, l = 254 nm), t_R = 7.6 min and 8.7 min.

In addition, the carbamate **A** (111 mg, 50%) was isolated as an oil; R_f 0.15 [petrol−EtOAc (2:3)]; ν_{max}/cm^{-1} (film) 3425, 2974, 2932, 2812, 1707, 1495, 1351, 1313, 1243, 1160, 1113, 909, 729; ^{1}H NMR (400 MHz, $CDCl_3$) δ = 7.86 (1H, dd, *J* 9.0 and 8.0 Hz, ArH), 7.42−7.29 (4H, m, ArH), 7.04 (1H, td, *J* 8.5 and 3.0 Hz, ArH), 6.92 (1H, dd, *J* 9.0 and 3.0 Hz, ArH), 5.81 (1H, d, *J* 7.5 Hz, C=CH), 3.77−3.68 (4H, m, 2 x CH_2), 3.53 (2H, s, CH_2), 3.49 (1H, d, *J* 7.5 Hz, CH), 2.50−2.41 (4H, m, 2 x CH_2), 1.35 (3H, s, CH_3), 1.20 (3H, s, CH_3), 1.56 (9H, s, ^{t}Bu); ^{13}C NMR (100 MHz, $CDCl_3$) δ = 159.8 (d, *J* 244.5 Hz), 152.4, 142.4, 137.9, 137.3, 135.8, 134.0, 129.1, 126.2 (d, *J* 8.5 Hz), 125.2, 116.3, 115.8 (d, *J* 22.5 Hz), 113.3 (d, *J* 22.5 Hz), 81.7, 74.8, 67.0, 63.1, 53.6, 51.6, 27.6, 27.2, 26.0; ^{19}F NMR (376 MHz, $CDCl_3$) δ = −118.3 (1F, s); HRMS *m/z* (ES) Found: MH^+ 483.2654. $C_{28}H_{36}FN_2O_4$ requires MH^+, 483.2666; LRMS *m/z* (ES) 483 (100%); the enantiomeric ratio was determined to be 82:18 by CSP-HPLC (major component eluted at 23.7 min); $[\alpha]_{\mathrm{D}}^{23}$ –51 (0.3, $CHCl_3$). The enantiomers were resolved using HPLC (Cellulose–2, n-hexane–isopropanol = 95:5, flow rate = 1.0 mL/min, l = 254 nm), t_R = 20.4 min and 24.0 min.

3. Variable temperature ^{1}H NMR spectra and *in situ* IR spectra

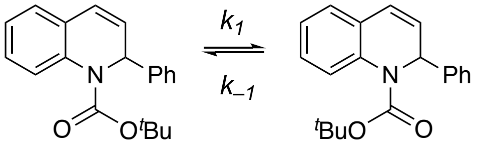

1a-1 **1a-2**

ratio 2 : 1
so $2k_1 = k_{-1}$

Coalescence of signals in the ^{1}H NMR spectrum of carbamate **1a** in D_8-THF was followed by taking spectra at various temperatures. The ratio of the rotamers is ~ 2:1 (major rotamer assumed to be **1a-1** from DFT studies – see Section 8). The ^{1}H NMR spectra in the region 7.85–7.30 ppm are shown below:

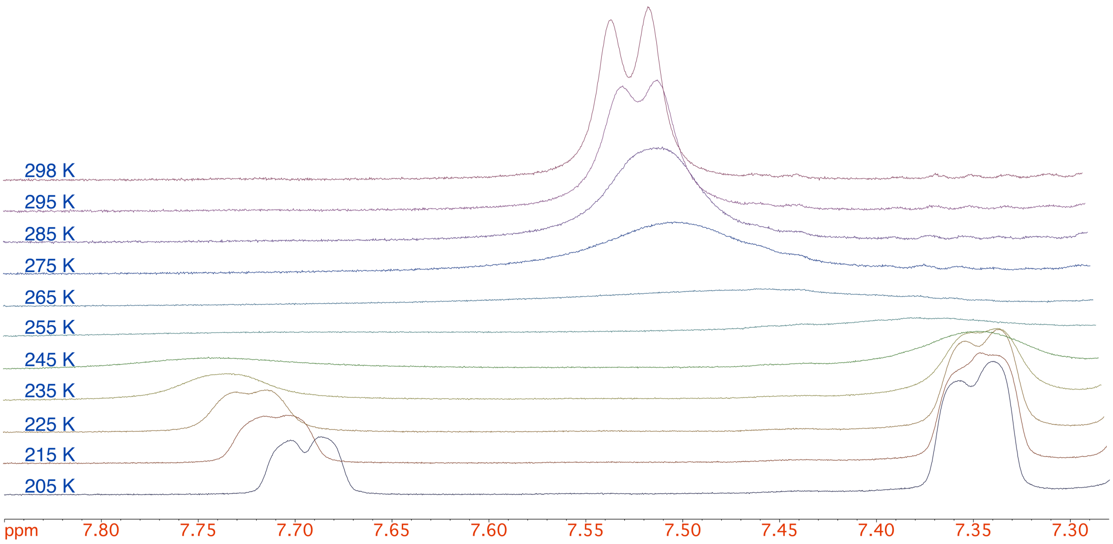

By using line shape analysis by DNMR (with the software iNMR) on the C-8 hydrogen atom peaks (at 7.73 and 7.40 ppm), the rate constants k_1 that fit best by overlaying the simulated spectra with the spectroscopic data can be estimated as follows:

T/K	1/T	k_1	ln(k_1/T)		k_{-1}	ln(k_{-1}/T)
205	0.004878	1	-5.32301		2	-4.62986
215	0.004651	5	-3.7612		10	-3.06805
225	0.004444	12	-2.93119		24	-2.23805
235	0.004255	45	-1.65292		90	-0.95978
245	0.004082	150	-0.49062		300	0.202524
255	0.003922	250	-0.0198		500	0.673345
265	0.003774	440	0.507045		880	1.200192
275	0.003636	1220	1.489835		2440	2.182982
285	0.003509	3300	2.449189		6600	3.142336
295	0.00339	8000	3.300221		16000	3.993369
298	0.003356	11000	3.608557		22000	4.301704

Table S2. *Line shape analysis from VT-NMR spectroscopy of carbamate **1a**.*

Eyring plot for major rotamer to minor rotamer (k_1):

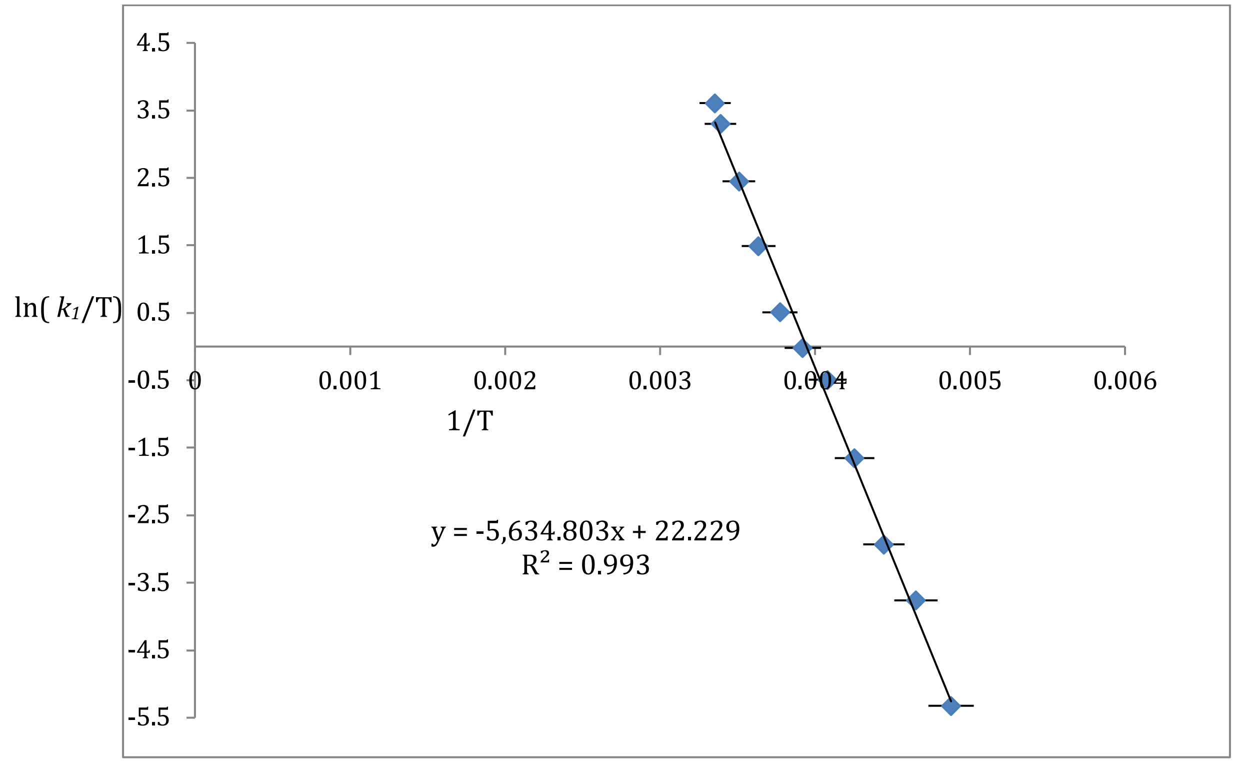

Eyring plot for minor rotamer to major rotamer (k_{-1}):

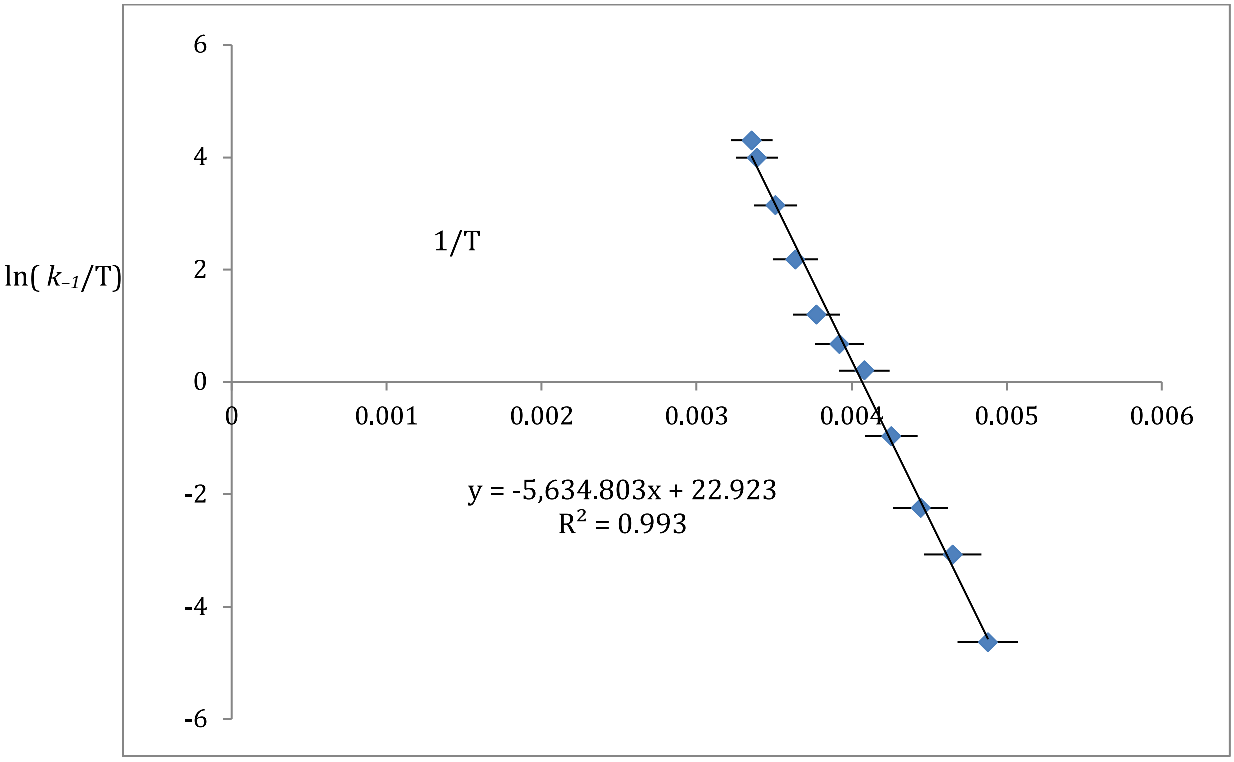

By using the Eyring equation, these plots gave the following data:

Forward direction (major to minor rotamer, k_1):
slope –5634.8, intercept 22.23
Approximate activation parameters for Boc rotation in THF:
$\Delta H^{\ddagger}$ 46.8 kJ/mol
$\Delta S^{\ddagger}$ –12.7 J/K·mol.
Hence the barrier to rotation $\Delta G^{\ddagger} \approx$ 49.3 kJ/mol at –78 °C.
The half-life for rotation is about 3 sec at –78 °C.

Reverse direction (minor to major rotamer, k_{-1}):
slope –5634.8, intercept 22.92
Approximate activation parameters for Boc rotation in THF:
$\Delta H^{\ddagger}$ 46.8 kJ/mol
$\Delta S^{\ddagger}$ –7 J/K·mol.
Hence the barrier to rotation $\Delta G^{\ddagger} \approx$ 48.2 kJ/mol at –78 °C.
The half-life for rotation is about 1.5 sec at –78 °C.

In situ IR spectroscopy:

The change in the carbonyl stretch in the IR spectrum of 1,2-dihydroquinoline **1a** was followed using *in situ* IR spectroscopy on addition of *n*-BuLi in THF (see Figure below). This shows that complete lithiation took place within a few minutes at –78 °C and therefore that rotation of the Boc group is rapid at this temperature.

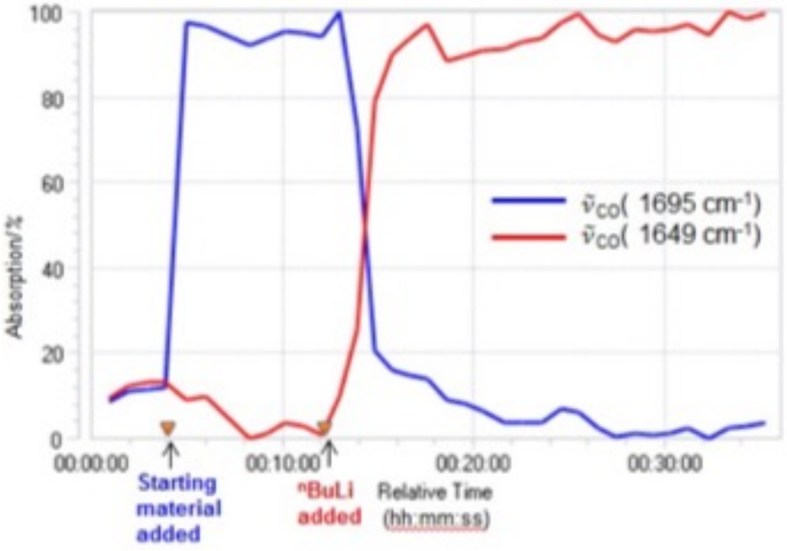

In situ IR spectroscopy of the deprotonation of dihydroquinoline **1a** with *n*-BuLi, THF at –78 °C; the blue line represents **1a** $\nu_{C=O}$ 1695 cm^{-1}, and red represents lithiated **1a** $\nu_{C=O}$ 1649 cm^{-1}.

4. Single crystal X-ray data

X-ray for compound (*S*)-1a

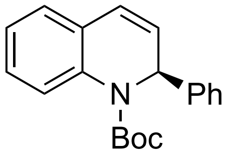

(*S*)-**1a**

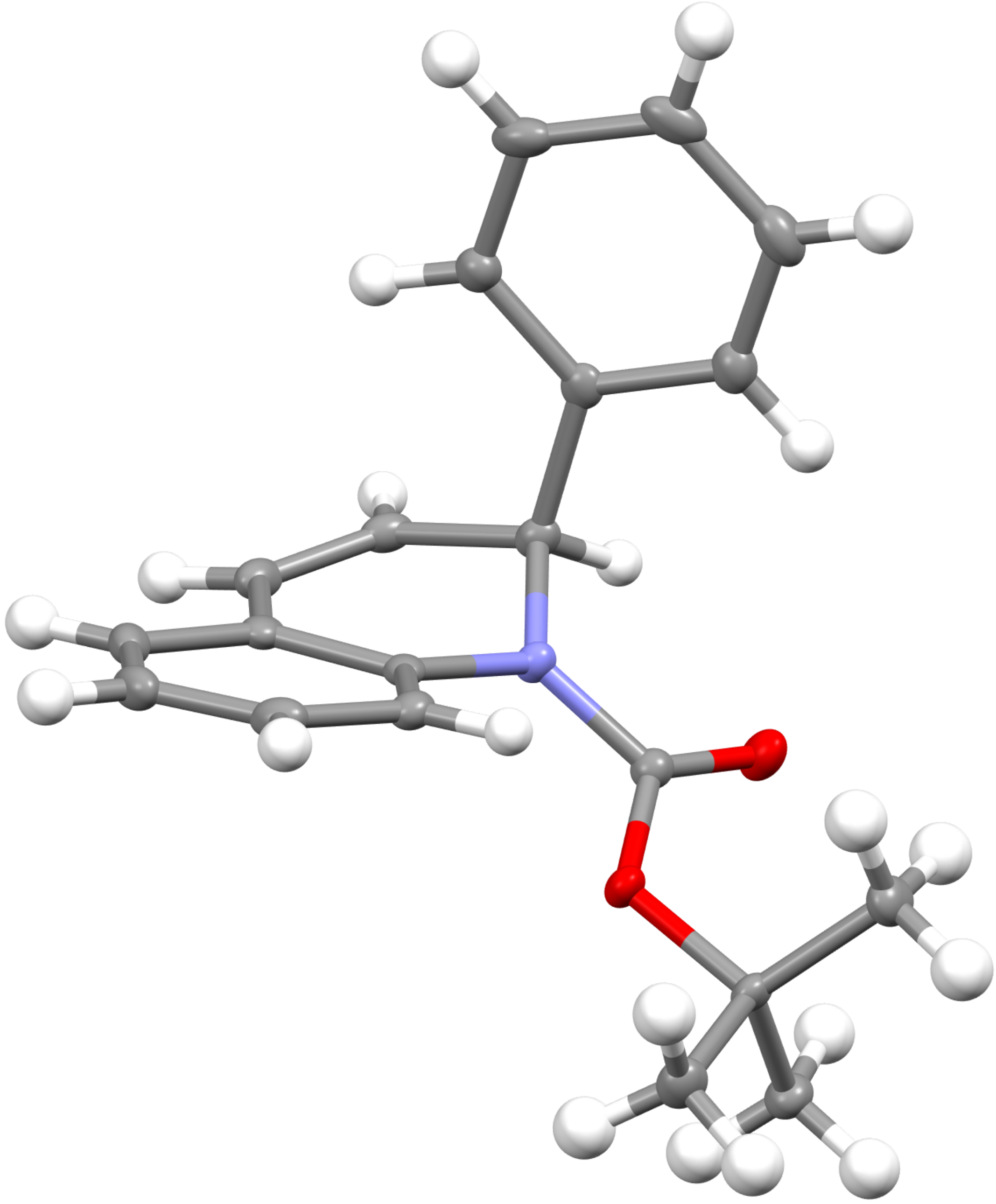

Thermal ellipsoid plot displayed at 50% probability level

CCDC 2221943

Table S3 Crystal data and structure refinement for (*S*)-**1a** (oic286v_0m_a).

Identification code	oic286v_0m_a
Empirical formula	$C_{20}H_{21}NO_2$
Formula weight	307.38
Temperature/K	99.99
Crystal system	trigonal
Space group	$P3_2$
a/Å	9.1131(7)
b/Å	9.1131(7)
c/Å	16.9762(13)
α/°	90
β/°	90
γ/°	120
Volume/Å^3	1221.0(2)
Z	3
ρ_{calc}g/cm^3	1.254
μ/mm^{-1}	0.637
F(000)	492.0
Crystal size/mm^3	0.5 × 0.35 × 0.1
Radiation	CuKα (λ = 1.54178)
2Θ range for data collection/°	11.21 to 132.964
Index ranges	$-10 \le h \le 10$, $-10 \le k \le 10$, $-20 \le l \le 20$
Reflections collected	14856
Independent reflections	2827 [R_{int} = 0.0270, R_{sigma} = 0.0213]
Data/restraints/parameters	2827/1/211
Goodness-of-fit on F^2	1.089
Final R indexes [I>=2σ (I)]	R_1 = 0.0244, wR_2 = 0.0623
Final R indexes [all data]	R_1 = 0.0245, wR_2 = 0.0624
Largest diff. peak/hole / e Å^{-3}	0.14/-0.16
Flack parameter	0.09(5)

Table S4 Fractional Atomic Coordinates (×10[4]) and Equivalent Isotropic Displacement Parameters (Å[2]×10[3]) for oic286v_0m_a. U_{eq} is defined as 1/3 of of the trace of the orthogonalised U_{IJ} tensor.

Atom	*x*	*y*	*z*	U(eq)
O1	6948.6(17)	9899.0(15)	4612.1(8)	21.1(3)
O2	7785.4(15)	8533.7(15)	5467.9(7)	15.6(3)
N1	5557.2(17)	7013.6(18)	4674.0(8)	13.9(3)
C1	5293(2)	5461(2)	4999(1)	13.8(3)
C2	5314(2)	5198(2)	5808.6(10)	16.2(4)
C3	5075(2)	3661(2)	6083.7(11)	20.3(4)
C4	4825(2)	2378(2)	5561.7(11)	21.7(4)
C5	4760(2)	2625(2)	4758.5(11)	19.7(4)
C6	4962(2)	4151(2)	4466.9(10)	15.6(4)
C7	4813(2)	4413(2)	3626.5(10)	18.2(4)
C8	4591(2)	5682(2)	3388.4(10)	17.0(4)
C9	4532(2)	6890(2)	3972.9(9)	15.0(3)
C10	2739(2)	6426(2)	4228.0(9)	14.8(3)
C11	2521(2)	7652(2)	4620.6(10)	19.0(4)
C12	940(3)	7277(3)	4897.3(11)	23.9(4)
C13	-450(2)	5664(3)	4794.5(10)	23.6(4)
C14	-249(2)	4443(2)	4404.7(11)	22.7(4)
C15	1342(2)	4829(2)	4118.6(10)	18.5(4)
C16	6797(2)	8611(2)	4903.7(9)	14.5(3)
C17	8894(2)	10067(2)	5942.4(10)	15.2(3)
C18	7869(2)	10832(2)	6265.9(11)	21.5(4)
C19	10388(2)	11309(2)	5446.6(11)	20.2(4)
C20	9460(2)	9340(2)	6607.3(10)	21.3(4)

Table S5 Anisotropic Displacement Parameters (Å[2]×10[3]) for oic286v_0m_a. The Anisotropic displacement factor exponent takes the form: -$2\pi^2[h^2a^{*2}U_{11}+2hka^*b^*U_{12}+\ldots]$.

Atom	U_{11}	U_{22}	U_{33}	U_{23}	U_{13}	U_{12}
O1	22.9(6)	13.1(6)	23.2(6)	1.3(5)	-5.3(5)	6.0(5)
O2	15.3(6)	11.8(6)	17.3(6)	-2.5(4)	-3.6(4)	5.0(5)
N1	14.7(7)	12.6(7)	13.3(7)	-0.1(5)	-2.3(5)	5.9(6)
C1	8.0(7)	12.9(8)	18.8(8)	0.9(6)	0.8(6)	4.0(6)
C2	12.5(8)	15.6(8)	17.9(9)	-0.9(6)	-0.2(6)	5.0(7)
C3	14.6(8)	20.9(9)	21.6(9)	6.0(7)	-1.4(6)	6.0(7)
C4	17.2(9)	15.8(9)	32.5(10)	4.5(7)	-1.6(7)	8.5(7)
C5	14.7(8)	16.2(8)	29(1)	-3.1(7)	-0.2(7)	8.5(7)

C6	9.4(8)	16.7(8)	20.0(9)	-1.5(7)	0.3(6)	6.0(6)
C7	14.0(8)	21.0(9)	18.0(8)	-6.0(7)	-0.4(6)	7.6(7)
C8	13.2(8)	21.3(9)	12.5(8)	-1.7(6)	-0.6(6)	5.6(7)
C9	15.4(8)	14.9(8)	12.7(8)	0.7(6)	-2.7(6)	6.2(7)
C10	19.1(9)	15.4(8)	11.1(8)	2.0(6)	-2.3(6)	9.6(7)
C11	23.8(9)	18.4(9)	16.9(8)	1.0(7)	-2.1(7)	12.1(8)
C12	33.1(11)	31.1(11)	18.1(9)	1.3(7)	1.3(8)	24.0(9)
C13	22.6(9)	39.6(11)	16.3(9)	6.7(8)	1.2(7)	21.4(9)
C14	14.4(9)	25.5(10)	24.4(9)	5.2(7)	-3.2(7)	7.1(7)
C15	18.2(9)	18.2(9)	20.5(9)	0.0(7)	-2.7(7)	10.0(7)
C16	13.9(8)	14.2(8)	14.0(7)	0.3(6)	-0.1(6)	6.0(7)
C17	13.2(8)	13.4(8)	16.2(8)	-4.2(6)	-3.0(6)	4.6(7)
C18	21.7(9)	22.6(9)	21.7(9)	-6.0(7)	-0.5(7)	12.3(8)
C19	16.3(9)	16.4(9)	24.1(9)	-0.2(7)	0.3(7)	5.5(7)
C20	23.8(9)	21.6(9)	17.8(9)	-3.0(7)	-5.4(7)	10.7(8)

Table S6 Bond Lengths for oic286v_0m_a.

Atom	Atom	Length/Å	Atom	Atom	Length/Å
O1	C16	1.216(2)	C7	C8	1.333(3)
O2	C16	1.341(2)	C8	C9	1.502(2)
O2	C17	1.4867(19)	C9	C10	1.531(2)
N1	C1	1.422(2)	C10	C11	1.398(3)
N1	C9	1.482(2)	C10	C15	1.386(3)
N1	C16	1.379(2)	C11	C12	1.386(3)
C1	C2	1.397(2)	C12	C13	1.391(3)
C1	C6	1.405(2)	C13	C14	1.382(3)
C2	C3	1.386(3)	C14	C15	1.397(3)
C3	C4	1.392(3)	C17	C18	1.520(2)
C4	C5	1.388(3)	C17	C19	1.518(2)
C5	C6	1.398(3)	C17	C20	1.523(2)
C6	C7	1.464(3)			

Table S7 Bond Angles for oic286v_0m_a.

Atom	Atom	Atom	Angle/°	Atom	Atom	Atom	Angle/°
C16	O2	C17	119.76(13)	C8	C9	C10	114.12(14)
C1	N1	C9	116.68(13)	C11	C10	C9	118.20(16)
C16	N1	C1	125.59(14)	C15	C10	C9	123.01(15)
C16	N1	C9	117.37(13)	C15	C10	C11	118.73(16)

C2	C1	N1	122.86(15)	C12	C11	C10	120.65(17)
C2	C1	C6	120.14(15)	C11	C12	C13	120.22(17)
C6	C1	N1	116.99(14)	C14	C13	C12	119.58(17)
C3	C2	C1	119.76(16)	C13	C14	C15	120.15(19)
C2	C3	C4	120.74(16)	C10	C15	C14	120.67(17)
C5	C4	C3	119.39(16)	O1	C16	O2	125.89(16)
C4	C5	C6	120.96(16)	O1	C16	N1	122.77(15)
C1	C6	C7	119.05(15)	O2	C16	N1	111.33(14)
C5	C6	C1	118.89(15)	O2	C17	C18	110.02(13)
C5	C6	C7	122.05(16)	O2	C17	C19	109.58(13)
C8	C7	C6	120.28(16)	O2	C17	C20	102.21(13)
C7	C8	C9	120.79(15)	C18	C17	C20	110.54(14)
N1	C9	C8	109.50(13)	C19	C17	C18	112.71(15)
N1	C9	C10	109.82(13)	C19	C17	C20	111.27(15)

Table S8 Hydrogen Atom Coordinates (Å×10^4) and Isotropic Displacement Parameters (Å^2×10^3) for oic286v_0m_a.

**Atom**	***x***	***y***	***z***	**U(eq)**
H2	5491	6068	6169	19
H3	5082	3481	6635	24
H4	4701	1342	5753	26
H5	4575	1746	4402	24
H7	4875	3676	3249	22
H8	4468	5825	2843	20
H9	5066	8034	3721	18
H11	3466	8754	4698	23
H12	805	8124	5158	29
H13	-1530	5402	4991	28
H14	-1195	3341	4331	27
H15	1467	3987	3846	22
H18A	7632	11412	5841	32
H18B	8516	11646	6682	32
H18C	6799	9930	6483	32
H19A	11010	10759	5257	30
H19B	11143	12298	5767	30
H19C	9977	11674	4996	30
H20A	8480	8589	6933	32
H20B	10304	10267	6932	32
H20C	9959	8697	6384	32

X-ray for compound (*R*)-2a

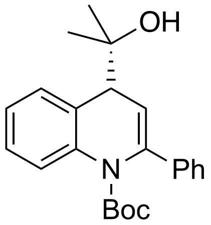

(*R*)-**2a**

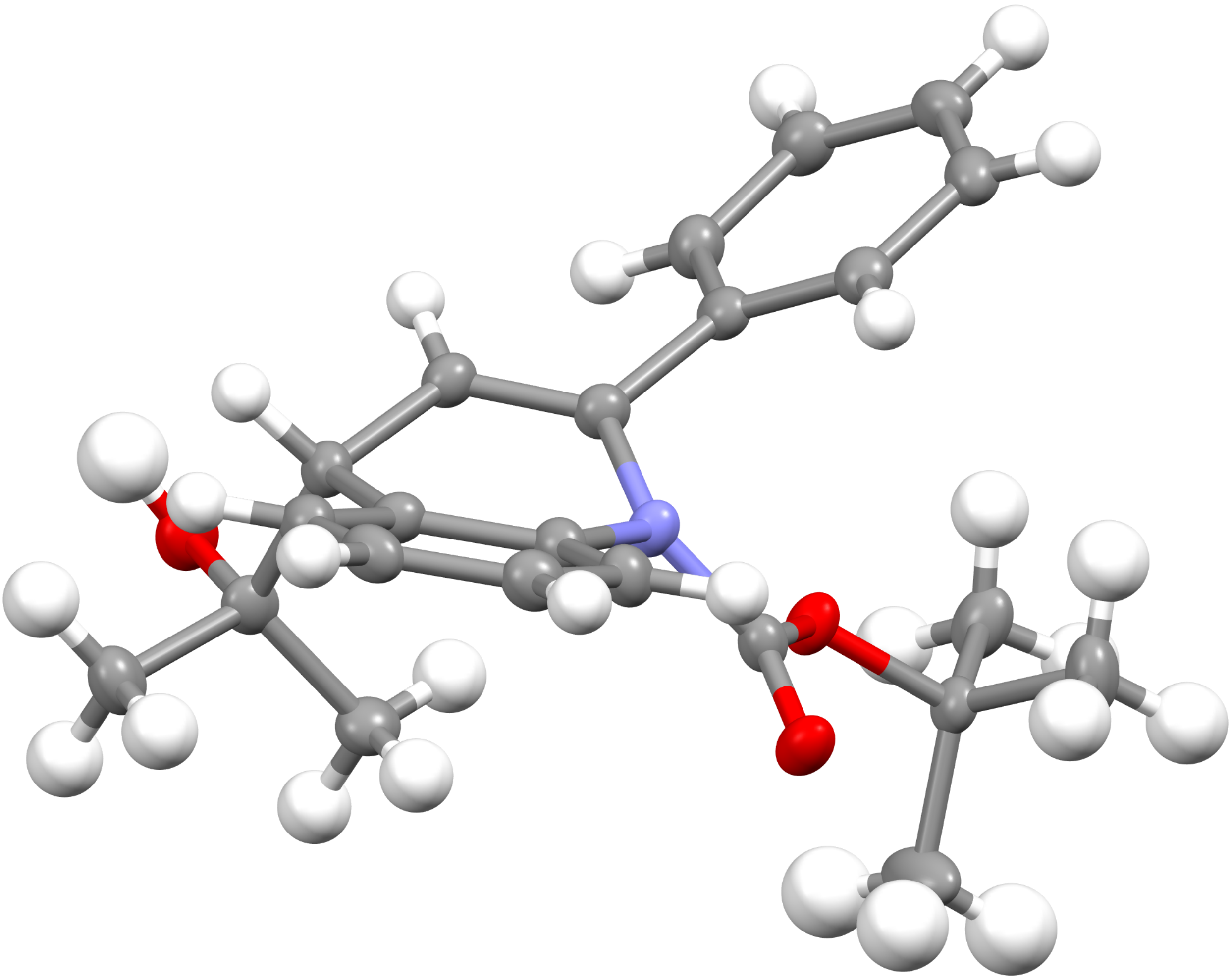

Thermal ellipsoid plot displayed at 50% probability level

CCDC 2221944

Table S9 Crystal data and structure refinement for (*R*)-**2a** (oic308v_0m).

Identification code	oic308v_0m
Empirical formula	$C_{23}H_{27}NO_3$
Formula weight	365.45
Temperature/K	99.97
Crystal system	orthorhombic
Space group	$P2_12_12_1$
a/Å	8.7574(9)
b/Å	9.9155(11)
c/Å	22.645(2)
α/°	90
β/°	90
γ/°	90
Volume/Å^3	1966.4(4)
Z	4
ρ_{calc}g/cm^3	1.234
μ/mm^{-1}	0.645
F(000)	784.0
Crystal size/mm^3	0.2 × 0.1 × 0.1
Radiation	CuKα (λ = 1.54178)
2Θ range for data collection/°	7.808 to 134.366
Index ranges	$-10 \leq h \leq 10$, $-11 \leq k \leq 11$, $-23 \leq l \leq 27$
Reflections collected	13552
Independent reflections	3466 [R_{int} = 0.0570, R_{sigma} = 0.0474]
Data/restraints/parameters	3466/0/253
Goodness-of-fit on F^2	1.061
Final R indexes [I>=2σ (I)]	R_1 = 0.0405, wR_2 = 0.1065
Final R indexes [all data]	R_1 = 0.0438, wR_2 = 0.1091
Largest diff. peak/hole / e Å^{-3}	0.18/-0.23
Flack parameter	0.11(12)

Table S10 Fractional Atomic Coordinates (×10^4) and Equivalent Isotropic Displacement Parameters (Å^2×10^3) for oic308v_0m. U_{eq} is defined as 1/3 of of the trace of the orthogonalised U_{IJ} tensor.

Atom	*x*	*y*	*z*	U(eq)
O1	4757(2)	6509(2)	3925.0(9)	26.9(5)
O2	2474(2)	5418(2)	3970.7(9)	24.0(5)
O3	1965(2)	3957(2)	1675.7(9)	30.8(5)
N1	4371(3)	4484(3)	3455.9(10)	21.2(5)
C1	3354(3)	3399(3)	3305.8(12)	21.9(6)
C2	3304(3)	2965(3)	2749.4(12)	22.1(6)
C3	4243(3)	3611(3)	2266.5(12)	22.7(6)
C4	5711(3)	4146(3)	2531.9(12)	21.3(6)

C5	5761(3)	4558(3)	3119.7(12)	21.8(6)
C6	7142(3)	4933(3)	3380.9(13)	25.6(6)
C7	8476(3)	4905(3)	3052.4(14)	26.9(6)
C8	8456(3)	4496(3)	2466.5(13)	26.8(7)
C9	7082(3)	4117(3)	2214.6(13)	24.2(6)
C10	3929(3)	5565(3)	3802.2(12)	21.5(6)
C11	1816(3)	6251(3)	4452.1(12)	21.7(6)
C12	2815(4)	6172(4)	4995.6(13)	34.9(8)
C13	1602(5)	7680(4)	4240.0(17)	43.2(9)
C14	305(3)	5540(4)	4569.5(14)	31.3(7)
C15	2572(3)	2678(3)	3795.2(12)	22.0(6)
C16	3269(3)	2538(3)	4344.4(12)	24.0(6)
C17	2579(4)	1788(3)	4786.5(13)	27.4(7)
C18	1203(4)	1140(3)	4684.0(14)	27.7(7)
C19	491(4)	1266(3)	4140.3(14)	29.0(7)
C20	1169(3)	2043(3)	3700.2(13)	26.8(7)
C21	3299(3)	4652(3)	1899.8(13)	24.2(6)
C22	4234(4)	5173(3)	1382.9(13)	29.8(7)
C23	2704(3)	5802(3)	2269.9(14)	27.9(7)

Table S11 Anisotropic Displacement Parameters ($Å^2 \times 10^3$) for oic308v_0m. The Anisotropic displacement factor exponent takes the form: $-2\pi^2[h^2a^{*2}U_{11}+2hka^*b^*U_{12}+\ldots]$.

**Atom**	**U_{11}**	**U_{22}**	**U_{33}**	**U_{23}**	**U_{13}**	**U_{12}**
O1	25.1(11)	26.3(11)	29.3(11)	-5.9(9)	6.0(9)	-7.2(9)
O2	17.7(9)	28.5(11)	25.8(10)	-7.5(8)	4.3(8)	-2.4(9)
O3	24.5(11)	37.0(13)	30.9(11)	-2.4(10)	-5.1(9)	-7.0(10)
N1	16.5(11)	24.2(12)	23.0(11)	-4.0(10)	2.6(9)	-3.2(10)
C1	17.2(13)	23.4(14)	25.2(14)	-1.5(11)	0.1(11)	-2.2(11)
C2	18.8(13)	22.7(14)	24.6(14)	-3.2(12)	-0.5(12)	-0.9(11)
C3	22.1(14)	25.0(15)	21.0(13)	-1.8(12)	3.0(11)	-0.6(12)
C4	18.6(14)	20.4(14)	24.8(14)	-1.0(11)	1.3(11)	-1.2(11)
C5	17.7(13)	24.1(14)	23.6(14)	-0.5(11)	2.7(11)	0.2(11)
C6	22.2(14)	31.3(16)	23.3(14)	1.7(12)	-1.1(12)	-2.6(12)
C7	18.8(14)	30.3(16)	31.6(15)	1.7(13)	-1.5(12)	-3.2(12)
C8	21.4(14)	28.8(15)	30.2(15)	5.5(13)	7.6(12)	2.1(12)
C9	24.0(14)	23.5(14)	25.3(13)	-1.4(12)	3.8(12)	-0.3(12)
C10	18.4(13)	26.7(14)	19.4(13)	0.1(11)	0.8(11)	-3.3(12)
C11	19.7(14)	25.9(15)	19.6(13)	-3.7(11)	5.0(11)	0.3(12)
C12	28.7(17)	53(2)	23.3(14)	-3.9(14)	1.5(13)	-5.6(16)
C13	47(2)	32.9(19)	50(2)	7.4(16)	26.0(18)	13.1(17)
C14	21.0(15)	39.8(18)	33.2(16)	-10.3(14)	6.5(13)	-5.2(14)

C15	20.2(14)	22.0(14)	23.9(14)	-2.4(11)	4.7(11)	1.0(11)
C16	18.5(14)	26.2(15)	27.3(14)	-2.2(12)	1.1(11)	2.4(12)
C17	29.5(16)	28.7(16)	24.1(14)	-0.2(13)	1.3(12)	6.5(13)
C18	28.2(15)	25.3(15)	29.5(15)	1.8(12)	8.9(13)	0.2(13)
C19	22.8(14)	31.3(17)	33.1(16)	-4.5(13)	5.9(13)	-4.8(13)
C20	23.5(15)	31.6(16)	25.4(14)	-2.8(13)	0.3(12)	-1.0(13)
C21	20.2(14)	27.0(15)	25.5(14)	-0.8(12)	-5.3(12)	-5.9(12)
C22	26.5(15)	37.8(18)	25.2(15)	3.2(13)	-3.3(12)	-4.7(14)
C23	23.5(15)	27.7(16)	32.7(15)	0.8(13)	-3.5(12)	2.9(12)

Table S12 Bond Lengths for oic308v_0m.

Atom	Atom	Length/Å	Atom	Atom	Length/Å
O1	C10	1.217(4)	C6	C7	1.385(4)
O2	C10	1.338(3)	C7	C8	1.388(4)
O2	C11	1.484(3)	C8	C9	1.384(4)
O3	C21	1.448(3)	C11	C12	1.512(4)
N1	C1	1.437(4)	C11	C13	1.508(5)
N1	C5	1.437(3)	C11	C14	1.523(4)
N1	C10	1.383(4)	C15	C16	1.393(4)
C1	C2	1.332(4)	C15	C20	1.397(4)
C1	C15	1.486(4)	C16	C17	1.386(4)
C2	C3	1.511(4)	C17	C18	1.385(4)
C3	C4	1.515(4)	C18	C19	1.386(5)
C3	C21	1.561(4)	C19	C20	1.393(4)
C4	C5	1.393(4)	C21	C22	1.519(4)
C4	C9	1.399(4)	C21	C23	1.509(4)
C5	C6	1.397(4)			

Table S13 Bond Angles for oic308v_0m.

Atom	Atom	Atom	Angle/°	Atom	Atom	Atom	Angle/°
C10	O2	C11	121.2(2)	O1	C10	N1	124.0(3)
C1	N1	C5	116.0(2)	O2	C10	N1	110.1(2)
C10	N1	C1	122.7(2)	O2	C11	C12	110.2(2)
C10	N1	C5	119.8(2)	O2	C11	C13	109.7(2)
N1	C1	C15	118.0(2)	O2	C11	C14	102.0(2)
C2	C1	N1	119.1(3)	C12	C11	C14	109.7(2)
C2	C1	C15	122.3(3)	C13	C11	C12	112.3(3)
C1	C2	C3	122.0(3)	C13	C11	C14	112.5(3)
C2	C3	C4	108.8(2)	C16	C15	C1	120.8(3)
C2	C3	C21	112.1(2)	C16	C15	C20	118.6(3)
C4	C3	C21	115.4(2)	C20	C15	C1	120.5(3)

C5	C4	C3	120.5(2)	C17	C16	C15	120.5(3)
C5	C4	C9	118.0(3)	C18	C17	C16	120.5(3)
C9	C4	C3	121.1(3)	C17	C18	C19	119.9(3)
C4	C5	N1	117.7(2)	C18	C19	C20	119.6(3)
C4	C5	C6	120.6(2)	C19	C20	C15	121.0(3)
C6	C5	N1	121.5(2)	O3	C21	C3	107.4(2)
C7	C6	C5	119.8(3)	O3	C21	C22	109.1(2)
C6	C7	C8	120.6(3)	O3	C21	C23	106.0(2)
C9	C8	C7	119.0(3)	C22	C21	C3	110.5(2)
C8	C9	C4	121.9(3)	C23	C21	C3	112.8(2)
O1	C10	O2	125.9(3)	C23	C21	C22	110.9(3)

Table S14 Hydrogen Atom Coordinates (Å×10^4) and Isotropic Displacement Parameters (Å^2×10^3) for oic308v_0m.

**Atom**	***x***	***y***	***z***	**U(eq)**
H3	2320(50)	3230(50)	1480(20)	64(14)
H2	2658.85	2227.04	2653.42	26
H3A	4539.06	2872.58	1988.42	27
H6	7166.77	5205.87	3782.94	31
H7	9412.07	5169.69	3229.69	32
H8	9372.02	4475.11	2241.94	32
H9	7068.68	3829.75	1814.47	29
H12A	2937.9	5226.72	5112.84	52
H12B	2336.71	6676.79	5318.34	52
H12C	3818.45	6561.22	4907.84	52
H13A	2601.46	8090.07	4165.43	65
H13B	1060.92	8201.36	4542.42	65
H13C	1003.11	7677.6	3874.34	65
H14A	-349.77	5613.15	4220.1	47
H14B	-203.15	5962.51	4907.71	47
H14C	496.07	4586.33	4656.31	47
H16	4225.79	2959.13	4416.52	29
H17	3052.34	1718.45	5162.74	33
H18	748.98	608.94	4985.9	33
H19	-454.37	825.15	4068.26	35
H20	671.34	2142.13	3330.06	32
H22A	4592.23	4410.92	1144.93	45
H22B	5114.5	5680.25	1531.87	45
H22C	3599.34	5765.8	1138.56	45
H23A	2100.27	6411.14	2021.35	42
H23B	3564.82	6298.25	2441.12	42
H23C	2060.06	5445.62	2587.44	42

X-ray for compound (*R*)-3a

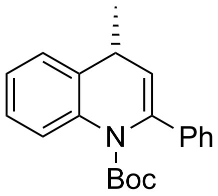

(*R*)-**3a**

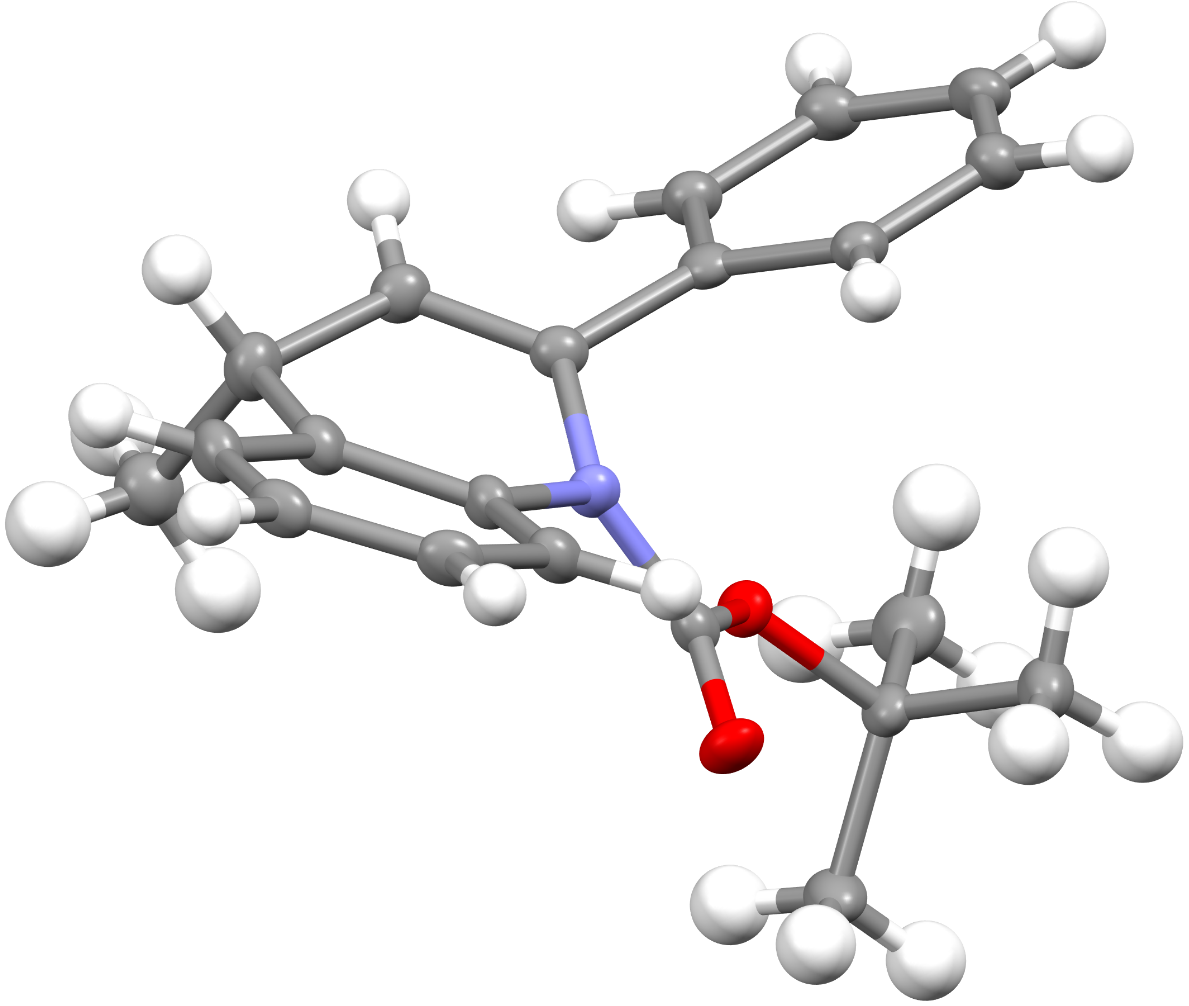

Thermal ellipsoid plot displayed at 50% probability level

CCDC 2221945

Table S15 Crystal data and structure refinement for (*R*)-**3a** (oic318v_0m).

Identification code	OIC318v_0m
Empirical formula	$C_{21}H_{23}NO_2$
Formula weight	321.40
Temperature/K	100.01
Crystal system	monoclinic
Space group	$P2_1$
a/Å	9.8984(4)
b/Å	16.1478(6)
c/Å	11.9552(5)
α/°	90
β/°	112.850(2)
γ/°	90
Volume/Å^3	1760.93(12)
Z	4
ρ_{calc}g/cm^3	1.212
μ/mm^{-1}	0.610
F(000)	688.0
Crystal size/mm^3	0.3 × 0.25 × 0.14
Radiation	CuKα (λ = 1.54178)
2Θ range for data collection/°	8.024 to 133.27
Index ranges	$-11 \leq h \leq 11$, $-19 \leq k \leq 19$, $-14 \leq l \leq 14$
Reflections collected	87863
Independent reflections	6219 [R_{int} = 0.0463, R_{sigma} = 0.0172]
Data/restraints/parameters	6219/1/441
Goodness-of-fit on F^2	1.084
Final R indexes [I>=2σ (I)]	R_1 = 0.0420, wR_2 = 0.1080
Final R indexes [all data]	R_1 = 0.0429, wR_2 = 0.1085
Largest diff. peak/hole / e Å^{-3}	0.27/-0.21
Flack parameter	0.02(7)

Table S16 Fractional Atomic Coordinates (×10^4) and Equivalent Isotropic Displacement Parameters (Å^2×10^3) for OIC318v_0m. U_{eq} is defined as 1/3 of of the trace of the orthogonalised U_{IJ} tensor.

**Atom**	***x***	***y***	***z***	**U(eq)**
O1	9453(3)	6021.6(16)	8166(2)	23.5(5)
O2	8584(2)	5891.5(14)	6110(2)	19.6(5)
N1	8694(3)	4771.4(18)	7215(2)	16.6(6)
C1	8625(3)	4346(2)	8248(3)	16.3(7)
C2	9766(4)	4390(2)	9382(3)	19.1(7)

C3	9658(4)	3932(2)	10329(3)	21.9(7)
C4	8430(4)	3448(2)	10144(3)	21.9(7)
C5	7297(4)	3416(2)	9005(3)	20.4(7)
C6	7378(3)	3866(2)	8044(3)	17.4(7)
C7	6198(3)	3878(2)	6766(3)	18.0(6)
C8	6957(3)	3858(2)	5881(3)	19.0(7)
C9	8143(3)	4309(2)	6092(3)	16.9(7)
C10	5030(3)	3205(2)	6517(3)	25.3(7)
C11	9020(4)	4352(2)	5328(3)	18.0(7)
C12	10541(4)	4433(2)	5848(3)	22.3(8)
C13	11345(4)	4504(2)	5128(4)	24.6(8)
C14	10637(4)	4488(2)	3869(4)	27.1(8)
C15	9130(4)	4395(2)	3340(3)	26.8(8)
C16	8319(4)	4331(2)	4062(3)	22.0(7)
C17	8966(3)	5617(2)	7243(3)	17.6(7)
C18	8936(4)	6748(2)	5861(3)	22.2(8)
C19	8136(4)	7367(2)	6322(4)	26.9(8)
C20	10581(4)	6877(3)	6389(4)	30.1(9)
C21	8353(5)	6747(3)	4474(4)	31.8(9)
O3	4272(3)	4010.0(15)	3117(2)	24.0(5)
O4	3447(3)	4144.2(15)	1064(2)	19.5(5)
N2	3654(3)	5281.2(19)	2188(3)	18.4(6)
C22	3611(3)	5702(2)	3230(3)	18.1(7)
C23	4688(4)	5571(2)	4377(3)	21.2(7)
C24	4637(4)	6016(2)	5359(3)	21.4(7)
C25	3526(4)	6583(2)	5198(3)	22.6(8)
C26	2479(4)	6725(2)	4046(3)	23.6(8)
C27	2516(4)	6296(2)	3044(3)	21.5(7)
C28	1447(4)	6513(2)	1769(3)	26.3(8)
C29	2147(4)	6324(2)	887(3)	21.4(7)
C30	3171(3)	5745(2)	1077(3)	18.6(7)
C31	-21(4)	6100(3)	1429(3)	32.2(8)
C32	3995(4)	5635(2)	286(3)	19.1(7)
C33	3260(4)	5692(2)	-974(3)	21.7(7)
C34	4039(4)	5614(2)	-1719(3)	24.6(8)
C35	5526(4)	5479(2)	-1227(3)	25.3(8)
C36	6262(4)	5419(2)	20(4)	25.0(8)
C37	5500(4)	5498(2)	778(3)	20.0(7)
C38	3837(3)	4428(2)	2204(3)	17.4(7)
C39	3862(4)	3301(2)	803(3)	21.0(7)
C40	3035(4)	2646(2)	1204(4)	28.0(8)
C41	5499(4)	3187(3)	1412(4)	28.9(9)
C42	3358(5)	3318(3)	-567(4)	33.2(9)

Table S17 Anisotropic Displacement Parameters (Å^2×10^3) for OIC318v_0m. The Anisotropic displacement factor exponent takes the form: -2π^2[h^{2}a*^{2}U$_{11}$+2hka*b*U$_{12}$+…].

Atom	U_{11}	U_{22}	U_{33}	U_{23}	U_{13}	U_{12}
O1	26.0(12)	25.1(13)	17.6(12)	-2.5(11)	6.4(9)	-2.9(10)
O2	25.0(12)	15.3(12)	17.8(12)	0.9(10)	7.5(9)	-0.6(10)
N1	19.0(13)	17.6(15)	11.9(13)	0.9(11)	4.7(11)	-1.5(11)
C1	18.7(15)	16.5(16)	15.2(16)	-0.4(14)	8.2(13)	2.4(13)
C2	18.4(16)	21.9(18)	16.6(16)	-0.8(14)	6.3(13)	-0.9(13)
C3	24.5(17)	23.3(18)	20.6(17)	-2.2(15)	11.7(14)	3.3(14)
C4	26.6(17)	20.2(18)	23.8(18)	2.2(15)	15.1(14)	2.6(14)
C5	19.8(16)	18.7(17)	25.7(18)	0.0(14)	12.1(14)	1.2(13)
C6	15.6(14)	18.5(16)	18.3(16)	-2.4(13)	6.8(12)	1.8(13)
C7	14.5(14)	17.2(15)	20.7(16)	-0.3(13)	5.3(12)	1.2(12)
C8	16.7(15)	20.1(16)	15.0(15)	0.9(13)	0.6(12)	3.6(13)
C9	18.9(16)	15.8(16)	12.3(16)	0.8(13)	1.9(12)	1.7(13)
C10	17.7(15)	30.5(19)	26.8(17)	-5.9(14)	7.5(13)	-6.2(14)
C11	23.0(16)	14.8(17)	15.7(16)	-3.0(13)	6.8(13)	0.4(14)
C12	24.7(17)	20.0(18)	21.7(18)	0.1(14)	8.4(14)	2.3(14)
C13	23.3(17)	20.5(17)	30.2(19)	-1.6(15)	10.5(15)	0.8(13)
C14	38(2)	22.4(18)	29(2)	2.3(15)	21.5(16)	4.5(16)
C15	38(2)	27(2)	16.1(17)	0.4(15)	11.5(15)	5.2(17)
C16	23.7(17)	21.1(18)	19.0(17)	-0.5(15)	6.0(14)	1.7(15)
C17	12.6(14)	20.8(18)	19.5(17)	0.2(14)	6.5(12)	-0.5(13)
C18	27.9(19)	18.7(18)	21.9(18)	4.5(15)	11.9(15)	0.7(15)
C19	28.3(19)	21.3(19)	32(2)	2.7(16)	13.3(16)	3.5(15)
C20	23.1(18)	30(2)	41(2)	7.8(18)	16.0(17)	0.1(16)
C21	46(2)	27(2)	26(2)	4.8(17)	16.7(18)	1.5(18)
O3	31.1(13)	19.9(13)	20.0(12)	5.6(11)	8.7(10)	4.7(10)
O4	22.1(11)	18.7(13)	17.6(12)	-2.8(10)	7.6(9)	0.1(9)
N2	19.1(13)	20.2(15)	15.6(14)	2.6(12)	6.3(11)	2.2(11)
C22	19.5(16)	19.7(17)	17.1(16)	-1.9(15)	9.3(13)	0.0(14)
C23	18.7(16)	26.1(19)	19.8(17)	2.8(15)	8.5(13)	0.1(14)
C24	23.0(16)	26.8(19)	11.3(15)	1.3(14)	3.2(12)	-3.6(14)
C25	26.9(18)	26.3(19)	17.3(17)	-4.9(14)	11.6(14)	-5.8(15)
C26	22.7(17)	25.8(18)	25.2(18)	-3.7(15)	12.7(14)	0.6(14)
C27	18.0(16)	25.9(18)	20.9(17)	-1.9(14)	8.0(14)	-1.9(13)
C28	24.0(17)	30(2)	23.5(17)	1.7(15)	7.4(14)	5.4(14)
C29	22.6(17)	22.7(17)	16.4(16)	0.0(13)	4.8(13)	0.8(14)
C30	19.1(16)	19.8(17)	14.2(16)	0.0(14)	3.7(13)	-1.9(14)
C31	27.7(18)	38(2)	29.3(18)	1.3(16)	8.8(14)	0.4(15)
C32	22.9(16)	12.5(16)	21.4(18)	-0.9(13)	8.3(14)	-2.4(13)
C33	23.0(16)	21.6(18)	19.3(17)	2.8(14)	7.0(14)	0.7(14)
C34	29.5(18)	23.6(18)	20.9(18)	1.4(15)	10.0(15)	-1.8(15)
C35	34.4(19)	21.7(18)	26.4(19)	3.5(15)	19.1(16)	1.6(15)

C36	24.1(17)	24.6(19)	28.2(19)	3.1(15)	12.2(15)	0.2(14)
C37	20.0(16)	18.3(17)	20.3(17)	2.7(13)	6.2(14)	-0.2(13)
C38	14.4(14)	21.4(18)	16.4(16)	1.1(14)	6.0(12)	0.5(13)
C39	23.3(17)	15.3(17)	27.3(19)	-1.8(15)	12.8(15)	1.4(14)
C40	28.4(19)	19.3(18)	40(2)	-3.0(17)	17.1(17)	-4.8(15)
C41	26.6(19)	23.9(19)	40(2)	-2.0(17)	17.2(17)	1.2(16)
C42	45(2)	28(2)	28(2)	-8.0(17)	15.4(18)	1.0(18)

Table S18 Bond Lengths for OIC318v_0m.

Atom	Atom	Length/Å	Atom	Atom	Length/Å
O1	C17	1.210(4)	O3	C38	1.212(4)
O2	C17	1.332(4)	O4	C38	1.344(4)
O2	C18	1.484(4)	O4	C39	1.490(4)
N1	C1	1.437(4)	N2	C22	1.435(4)
N1	C9	1.446(4)	N2	C30	1.435(4)
N1	C17	1.390(5)	N2	C38	1.389(5)
C1	C2	1.389(5)	C22	C23	1.387(5)
C1	C6	1.396(5)	C22	C27	1.399(5)
C2	C3	1.391(5)	C23	C24	1.394(5)
C3	C4	1.388(5)	C24	C25	1.386(5)
C4	C5	1.388(5)	C25	C26	1.384(5)
C5	C6	1.388(5)	C26	C27	1.396(5)
C6	C7	1.521(4)	C27	C28	1.521(5)
C7	C8	1.517(5)	C28	C29	1.500(5)
C7	C10	1.529(4)	C28	C31	1.504(5)
C8	C9	1.320(5)	C29	C30	1.332(5)
C9	C11	1.486(5)	C30	C32	1.480(5)
C11	C12	1.394(5)	C32	C33	1.398(5)
C11	C16	1.399(5)	C32	C37	1.391(5)
C12	C13	1.385(5)	C33	C34	1.392(5)
C13	C14	1.392(6)	C34	C35	1.374(5)
C14	C15	1.384(5)	C35	C36	1.385(5)
C15	C16	1.392(5)	C36	C37	1.392(5)
C18	C19	1.506(5)	C39	C40	1.525(5)
C18	C20	1.515(5)	C39	C41	1.508(5)
C18	C21	1.529(5)	C39	C42	1.516(5)

Table S19 Bond Angles for OIC318v_0m.

Atom	Atom	Atom	Angle/°	Atom	Atom	Atom	Angle/°
C17	O2	C18	121.2(3)	C38	O4	C39	122.0(3)
C1	N1	C9	115.1(3)	C22	N2	C30	116.4(3)

C17	N1	C1	121.5(3)	C38	N2	C22	120.4(3)
C17	N1	C9	122.0(3)	C38	N2	C30	121.7(3)
C2	C1	N1	121.4(3)	C23	C22	N2	121.0(3)
C2	C1	C6	121.8(3)	C23	C22	C27	120.8(3)
C6	C1	N1	116.7(3)	C27	C22	N2	117.9(3)
C1	C2	C3	118.5(3)	C22	C23	C24	119.3(3)
C4	C3	C2	120.6(3)	C25	C24	C23	120.6(3)
C3	C4	C5	120.1(3)	C26	C25	C24	119.6(3)
C6	C5	C4	120.6(3)	C25	C26	C27	120.9(3)
C1	C6	C7	116.8(3)	C22	C27	C28	120.8(3)
C5	C6	C1	118.5(3)	C26	C27	C22	118.7(3)
C5	C6	C7	124.7(3)	C26	C27	C28	120.3(3)
C6	C7	C10	114.5(3)	C29	C28	C27	109.0(3)
C8	C7	C6	107.7(2)	C29	C28	C31	112.4(3)
C8	C7	C10	113.0(3)	C31	C28	C27	113.0(3)
C9	C8	C7	120.0(3)	C30	C29	C28	123.5(3)
N1	C9	C11	116.5(3)	N2	C30	C32	117.4(3)
C8	C9	N1	116.7(3)	C29	C30	N2	118.8(3)
C8	C9	C11	126.7(3)	C29	C30	C32	123.2(3)
C12	C11	C9	121.2(3)	C33	C32	C30	119.7(3)
C12	C11	C16	118.7(3)	C37	C32	C30	121.0(3)
C16	C11	C9	120.1(3)	C37	C32	C33	119.3(3)
C13	C12	C11	120.8(3)	C34	C33	C32	119.9(3)
C12	C13	C14	120.0(3)	C35	C34	C33	120.5(3)
C15	C14	C13	119.8(3)	C34	C35	C36	120.0(3)
C14	C15	C16	120.2(3)	C35	C36	C37	120.2(3)
C15	C16	C11	120.4(3)	C32	C37	C36	120.1(3)
O1	C17	O2	127.1(3)	O3	C38	O4	125.8(3)
O1	C17	N1	123.8(3)	O3	C38	N2	124.3(3)
O2	C17	N1	109.0(3)	O4	C38	N2	109.9(3)
O2	C18	C19	110.3(3)	O4	C39	C40	110.0(3)
O2	C18	C20	110.0(3)	O4	C39	C41	110.0(3)
O2	C18	C21	101.5(3)	O4	C39	C42	102.0(3)
C19	C18	C20	112.9(3)	C41	C39	C40	111.8(3)
C19	C18	C21	111.5(3)	C41	C39	C42	111.4(3)
C20	C18	C21	110.1(3)	C42	C39	C40	111.1(3)

Table S20 Hydrogen Atom Coordinates (Å×10[4]) and Isotropic Displacement Parameters (Å[2]×10[3]) for OIC318v_0m.

**Atom**	***x***	***y***	***z***	**U(eq)**
H2	10602.11	4724.4	9507.26	23
H3	10430.84	3950.53	11109.72	26
H4	8363.89	3139.35	10797.19	26

H5	6460.05	3083.72	8882.74	24
H7	5684.75	4423.61	6658.72	22
H8	6579.1	3520.19	5176.14	23
H10A	5496.55	2658.96	6634.01	38
H10B	4313.97	3254.77	5679.25	38
H10C	4531.78	3270.46	7077.36	38
H12	11032.81	4439.83	6706.41	27
H13	12380.5	4562.79	5493.13	30
H14	11185.97	4541.83	3374.51	32
H15	8646.8	4374.33	2481.37	32
H16	7283.69	4272.89	3693.16	26
H19A	7098.04	7213.35	6035.33	40
H19B	8224.91	7919.18	6018.48	40
H19C	8563.88	7369.81	7211.15	40
H20A	10943.04	6877.06	7277.13	45
H20B	10811.06	7408.94	6109.69	45
H20C	11052.58	6428.53	6121.61	45
H21A	8875	6328.31	4202.71	48
H21B	8507.38	7294.32	4188.26	48
H21C	7302.59	6619.56	4141.87	48
H23	5450.68	5182.43	4491.41	25
H24	5371.17	5929.01	6146.65	26
H25	3482.82	6872.32	5875.42	27
H26	1725.77	7119.35	3934.72	28
H28	1279.38	7124.8	1745.97	32
H29	1844.1	6633.78	155.07	26
H31A	-649.12	6243.97	591.54	48
H31B	-481.66	6287.95	1976.56	48
H31C	112.41	5497.82	1498.64	48
H33	2231.59	5784.45	-1320.24	26
H34	3538.19	5654.71	-2574.86	29
H35	6049.82	5427.02	-1741.89	30
H36	7289.4	5323.55	358.84	30
H37	6008.93	5458.46	1633.24	24
H40A	3338.81	2670.22	2087.3	42
H40B	3254.32	2096.29	970.75	42
H40C	1978.53	2750.72	811.58	42
H41A	5993.96	3649.08	1196.61	43
H41B	5778	2665.61	1140.02	43
H41C	5789.33	3172.41	2293.98	43
H42A	2299.7	3420.84	-934.23	50
H42B	3574.43	2784.11	-850.37	50
H42C	3873.68	3760	-802.08	50

X-ray for compound (*R*)-6

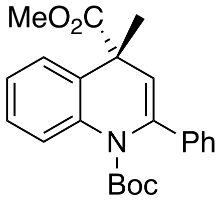

(*R*)-**6**

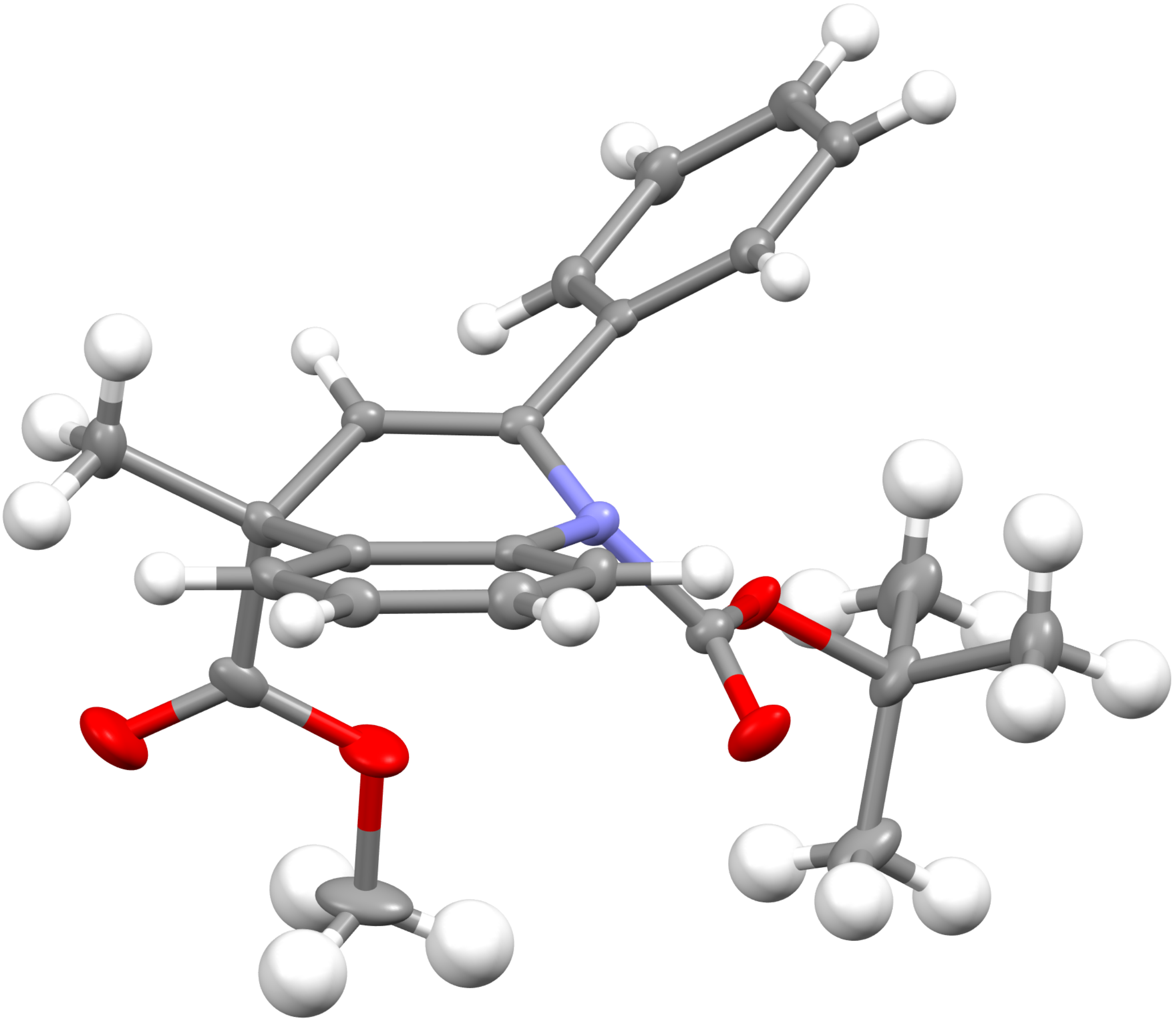

Thermal ellipsoid plot displayed at 50% probability level

CCDC 2221946

Table S21 Crystal data and structure refinement for (*R*)-**6** (oic321v_0m).

Identification code	OIC321v_0m
Empirical formula	$C_{23}H_{25}NO_4$
Formula weight	379.44
Temperature/K	100.01
Crystal system	orthorhombic
Space group	$P2_12_12_1$
a/Å	7.6782(4)
b/Å	12.9220(6)
c/Å	20.5879(10)
α/°	90
β/°	90
γ/°	90
Volume/Å^3	2042.68(17)
Z	4
ρ_{calc}g/cm^3	1.234
μ/mm^{-1}	0.680
F(000)	808.0
Crystal size/mm^3	0.246 × 0.222 × 0.086
Radiation	CuKα (λ = 1.54178)
2Θ range for data collection/°	8.078 to 133.422
Index ranges	$-9 \le h \le 9$, $-15 \le k \le 15$, $-24 \le l \le 24$
Reflections collected	66030
Independent reflections	3604 [R_{int} = 0.0422, R_{sigma} = 0.0122]
Data/restraints/parameters	3604/0/258
Goodness-of-fit on F^2	1.130
Final R indexes [I>=2σ (I)]	R_1 = 0.0272, wR_2 = 0.0675
Final R indexes [all data]	R_1 = 0.0277, wR_2 = 0.0679
Largest diff. peak/hole / e Å^{-3}	0.15/-0.23
Flack parameter	0.03(4)

Table S22 Fractional Atomic Coordinates (×10^4) and Equivalent Isotropic Displacement Parameters ($Å^2$×10^3) for OIC321v_0m. U_{eq} is defined as 1/3 of of the trace of the orthogonalised U_{IJ} tensor.

**Atom**	***x***	***y***	***z***	**U(eq)**
O1	4930.2(17)	7154.1(9)	3391.2(6)	24.5(3)
O2	4507.9(15)	5538.2(8)	3789.2(5)	19.5(3)
O3	4039(2)	4729.5(13)	1107.0(7)	45.5(4)
O4	3781.6(16)	5412.0(12)	2096.0(7)	32.1(3)
N1	6575.3(18)	5778.4(10)	3044.6(6)	14.5(3)
C1	7320(2)	6369.9(12)	2526.0(8)	14.4(3)
C2	8039(2)	7336.0(12)	2648.9(8)	19.1(3)
C3	8786(2)	7897.1(14)	2146.0(8)	22.1(4)
C4	8835(2)	7487.7(13)	1523.8(8)	20.1(4)
C5	8139(2)	6517.8(12)	1406.8(8)	17.9(3)
C6	7371(2)	5942.9(12)	1901.5(8)	15.0(3)
C7	6608(2)	4861.7(12)	1784.0(7)	16.5(3)
C8	6780(2)	4246.3(11)	2406.5(7)	14.9(3)
C9	6713(2)	4675.6(11)	2993.1(7)	13.6(3)
C10	5272(2)	6242.1(12)	3414.5(8)	16.4(3)
C11	3349(2)	5845.2(13)	4327.2(8)	23.2(4)
C12	1669(3)	6281.0(14)	4049.4(11)	31.9(4)
C13	4271(3)	6596.3(15)	4779.8(9)	32.9(5)
C14	3039(3)	4813.8(15)	4661.1(10)	33.2(5)
C15	7517(3)	4284.3(14)	1231.4(8)	24.2(4)
C16	4672(2)	4980.6(12)	1614.7(8)	21.4(4)
C17	1939(3)	5563.4(19)	1986.2(12)	40.4(5)
C18	6956(2)	4068.5(12)	3598.4(7)	14.8(3)
C19	6263(2)	3075.9(12)	3649.1(8)	17.9(3)
C20	6571(2)	2486.2(13)	4203.0(8)	22.0(4)
C21	7563(2)	2876.7(13)	4707.4(8)	22.0(4)
C22	8237(2)	3872.4(13)	4662.9(7)	19.1(3)
C23	7926(2)	4465.4(13)	4113.5(8)	16.2(3)

Table S23 Anisotropic Displacement Parameters (Å2×103) for OIC321v_0m. The Anisotropic displacement factor exponent takes the form: -2π2[h2a*2U11+2hka*b*U12+…].

Atom	U_{11}	U_{22}	U_{33}	U_{23}	U_{13}	U_{12}
O1	27.4(6)	13.8(6)	32.3(7)	2.4(5)	11.9(5)	2.9(5)
O2	21.3(6)	14.0(5)	23.2(6)	1.1(4)	10.9(5)	1.4(5)
O3	46.1(9)	52.3(9)	38.2(8)	-8.2(7)	-27.7(7)	8.3(8)
O4	13.0(6)	49.5(9)	33.8(7)	2.8(6)	-3.8(5)	1.1(6)
N1	15.8(6)	11.4(6)	16.2(6)	1.2(5)	2.5(5)	-0.3(5)
C1	11.6(7)	14.2(7)	17.4(8)	3.5(6)	1.3(6)	2.1(6)
C2	20.5(8)	17.4(8)	19.4(8)	-1.2(6)	3.0(7)	-2.6(7)
C3	22.4(8)	17.8(8)	26.2(9)	2.0(7)	1.8(7)	-5.3(7)
C4	17.9(8)	21.2(8)	21.3(8)	7.0(7)	3.4(6)	-1.4(7)
C5	16.3(8)	21.4(8)	16.1(7)	3.4(6)	-1.5(7)	1.5(7)
C6	12.8(7)	15.1(8)	16.9(7)	2.0(6)	-1.4(6)	1.4(6)
C7	19.7(8)	14.3(7)	15.4(7)	2.5(6)	-2.9(6)	-1.5(7)
C8	15.6(7)	11.9(7)	17.2(7)	1.6(6)	-0.6(6)	0.6(6)
C9	10.9(7)	11.9(7)	18.1(7)	1.5(6)	1.2(6)	-0.1(6)
C10	15.6(8)	15.0(8)	18.6(7)	0.7(6)	2.6(6)	0.5(6)
C11	25.0(9)	20.6(8)	24.0(8)	-2.8(7)	13.7(7)	-0.7(7)
C12	20.4(9)	23.7(9)	51.5(12)	-4.6(8)	13.5(9)	-1.6(8)
C13	41.6(11)	32.2(11)	24.8(9)	-4.4(8)	8.3(9)	-5.9(9)
C14	41.6(12)	25.4(9)	32.5(10)	4.2(8)	20.6(9)	-2.6(9)
C15	37.9(10)	18.8(8)	16.0(8)	-0.5(6)	2.4(7)	-0.2(7)
C16	25.4(9)	15.8(8)	23.0(8)	8.3(7)	-7.4(7)	-3.7(7)
C17	12.5(9)	49.1(13)	59.6(14)	23.1(11)	-5.0(9)	-0.9(9)
C18	13.0(7)	15.7(7)	15.8(7)	0.3(6)	3.9(6)	3.7(6)
C19	20.1(8)	16.8(8)	16.6(7)	-1.8(6)	2.7(6)	-1.1(6)
C20	31.5(9)	13.7(7)	20.9(8)	2.1(6)	8.3(7)	0.6(7)
C21	30.6(10)	20.9(8)	14.5(8)	4.5(6)	4.9(7)	8.0(7)
C22	19.6(8)	24.0(8)	13.9(7)	-1.5(6)	0.2(6)	4.7(7)
C23	15.8(8)	15.9(7)	17.0(7)	-0.6(6)	2.8(6)	0.6(6)

Table S24 Bond Lengths for OIC321v_0m.

Atom	Atom	Length/Å	Atom	Atom	Length/Å
O1	C10	1.208(2)	C6	C7	1.534(2)
O2	C10	1.329(2)	C7	C8	1.514(2)
O2	C11	1.4752(19)	C7	C15	1.529(2)
O3	C16	1.197(2)	C7	C16	1.535(2)
O4	C16	1.326(2)	C8	C9	1.330(2)
O4	C17	1.446(2)	C9	C18	1.484(2)
N1	C1	1.4324(19)	C11	C12	1.519(3)
N1	C9	1.4328(19)	C11	C13	1.520(3)

Table S24 Bond Lengths for OIC321v_0m.

Atom	Atom	Length/Å	Atom	Atom	Length/Å
N1	C10	1.393(2)	C11	C14	1.518(2)
C1	C2	1.388(2)	C18	C19	1.393(2)
C1	C6	1.400(2)	C18	C23	1.393(2)
C2	C3	1.388(2)	C19	C20	1.392(2)
C3	C4	1.387(2)	C20	C21	1.383(3)
C4	C5	1.383(2)	C21	C22	1.390(3)
C5	C6	1.392(2)	C22	C23	1.387(2)

Table S25 Bond Angles for OIC321v_0m.

Atom	Atom	Atom	Angle/°	Atom	Atom	Atom	Angle/°
C10	O2	C11	121.21(13)	C8	C9	N1	119.01(13)
C16	O4	C17	116.37(16)	C8	C9	C18	122.48(13)
C1	N1	C9	116.48(12)	O1	C10	O2	126.47(15)
C10	N1	C1	117.70(13)	O1	C10	N1	123.64(14)
C10	N1	C9	121.43(13)	O2	C10	N1	109.88(13)
C2	C1	N1	120.18(14)	O2	C11	C12	109.22(14)
C2	C1	C6	120.73(14)	O2	C11	C13	110.54(15)
C6	C1	N1	119.05(14)	O2	C11	C14	101.44(13)
C1	C2	C3	119.87(15)	C12	C11	C13	112.93(16)
C4	C3	C2	120.07(16)	C14	C11	C12	111.27(16)
C5	C4	C3	119.74(15)	C14	C11	C13	110.84(16)
C4	C5	C6	121.32(15)	O3	C16	O4	123.84(18)
C1	C6	C7	119.56(13)	O3	C16	C7	124.34(18)
C5	C6	C1	118.26(14)	O4	C16	C7	111.81(14)
C5	C6	C7	122.17(14)	C19	C18	C9	120.11(14)
C6	C7	C16	108.33(13)	C19	C18	C23	119.08(14)
C8	C7	C6	108.15(12)	C23	C18	C9	120.77(14)
C8	C7	C15	109.50(13)	C20	C19	C18	120.06(15)
C8	C7	C16	109.22(13)	C21	C20	C19	120.59(15)
C15	C7	C6	112.81(14)	C20	C21	C22	119.56(15)
C15	C7	C16	108.77(14)	C23	C22	C21	120.06(15)
C9	C8	C7	123.11(13)	C22	C23	C18	120.63(15)
N1	C9	C18	118.21(13)				

Table S26 Torsion Angles for OIC321v_0m.

A	B	C	D	Angle/°	A	B	C	D	Angle/°
N1	C1	C2	C3	178.87(15)	C8	C9	C18	C19	-38.5(2)
N1	C1	C6	C5	-178.40(14)	C8	C9	C18	C23	139.27(17)
N1	C1	C6	C7	0.8(2)	C9	N1	C1	C2	-147.75(15)
N1	C9	C18	C19	147.79(15)	C9	N1	C1	C6	29.9(2)
N1	C9	C18	C23	-34.4(2)	C9	N1	C10	O1	-167.74(16)
C1	N1	C9	C8	-28.8(2)	C9	N1	C10	O2	12.9(2)
C1	N1	C9	C18	145.10(14)	C9	C18	C19	C20	176.56(15)
C1	N1	C10	O1	-12.1(2)	C9	C18	C23	C22	-176.15(15)
C1	N1	C10	O2	168.55(13)	C10	O2	C11	C12	70.64(19)
C1	C2	C3	C4	-0.8(3)	C10	O2	C11	C13	-54.2(2)
C1	C6	C7	C8	-29.17(19)	C10	O2	C11	C14	-171.81(16)
C1	C6	C7	C15	-150.43(15)	C10	N1	C1	C2	55.4(2)
C1	C6	C7	C16	89.10(17)	C10	N1	C1	C6	-127.02(16)
C2	C1	C6	C5	-0.8(2)	C10	N1	C9	C8	127.16(17)
C2	C1	C6	C7	178.44(14)	C10	N1	C9	C18	-58.9(2)
C2	C3	C4	C5	-0.1(3)	C11	O2	C10	O1	-12.5(3)
C3	C4	C5	C6	0.6(2)	C11	O2	C10	N1	166.88(14)
C4	C5	C6	C1	-0.1(2)	C15	C7	C8	C9	154.82(16)
C4	C5	C6	C7	-179.37(14)	C15	C7	C16	O3	-5.4(2)
C5	C6	C7	C8	150.05(15)	C15	C7	C16	O4	175.57(13)
C5	C6	C7	C15	28.8(2)	C16	C7	C8	C9	-86.17(19)
C5	C6	C7	C16	-91.68(17)	C17	O4	C16	O3	0.8(3)
C6	C1	C2	C3	1.3(2)	C17	O4	C16	C7	179.81(15)
C6	C7	C8	C9	31.5(2)	C18	C19	C20	C21	0.0(3)
C6	C7	C16	O3	117.50(19)	C19	C18	C23	C22	1.7(2)
C6	C7	C16	O4	-61.48(17)	C19	C20	C21	C22	0.9(3)
C7	C8	C9	N1	-3.8(2)	C20	C21	C22	C23	-0.5(2)
C7	C8	C9	C18	-177.43(15)	C21	C22	C23	C18	-0.8(2)
C8	C7	C16	O3	-124.92(18)	C23	C18	C19	C20	-1.3(2)
C8	C7	C16	O4	56.10(18)					

Table S27 Hydrogen Atom Coordinates (Å×10^4) and Isotropic Displacement Parameters (Å^2×10^3) for OIC321v_0m.

**Atom**	***x***	***y***	***z***	**U(eq)**
H2	8018.65	7612.67	3076.33	23
H3	9264.13	8562.36	2228.32	27
H4	9344.31	7871.61	1179.18	24
H5	8186.91	6238.7	980.31	22
H8	6943.75	3519.11	2376.66	18
H12A	1910.99	6939.57	3831.21	48
H12B	832.36	6394.79	4401.72	48
H12C	1179.59	5789.85	3735.92	48
H13A	5409.17	6312.99	4901.35	49
H13B	3565.82	6699.21	5171.24	49
H13C	4432.38	7260.87	4558.46	49
H14A	2500.68	4330.6	4354.1	50
H14B	2263.77	4914.4	5033.91	50
H14C	4153.12	4530.79	4810.87	50
H15A	8762.96	4225.56	1327.24	36
H15B	7357.02	4666.24	824.47	36
H15C	7012.28	3591.07	1188.18	36
H17A	1768.97	5983.51	1595.02	61
H17B	1426.22	5919.11	2360.69	61
H17C	1371.8	4890.09	1928.63	61
H19	5579.98	2800.99	3305.26	21
H20	6095.04	1809.44	4234.94	26
H21	7781.34	2467.14	5081.81	26
H22	8912.33	4146.91	5009.06	23
H23	8377.22	5148.68	4088.23	19

X-ray for compound 7

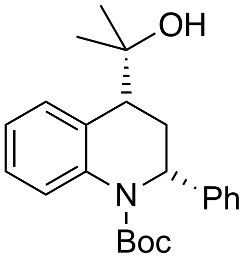

7

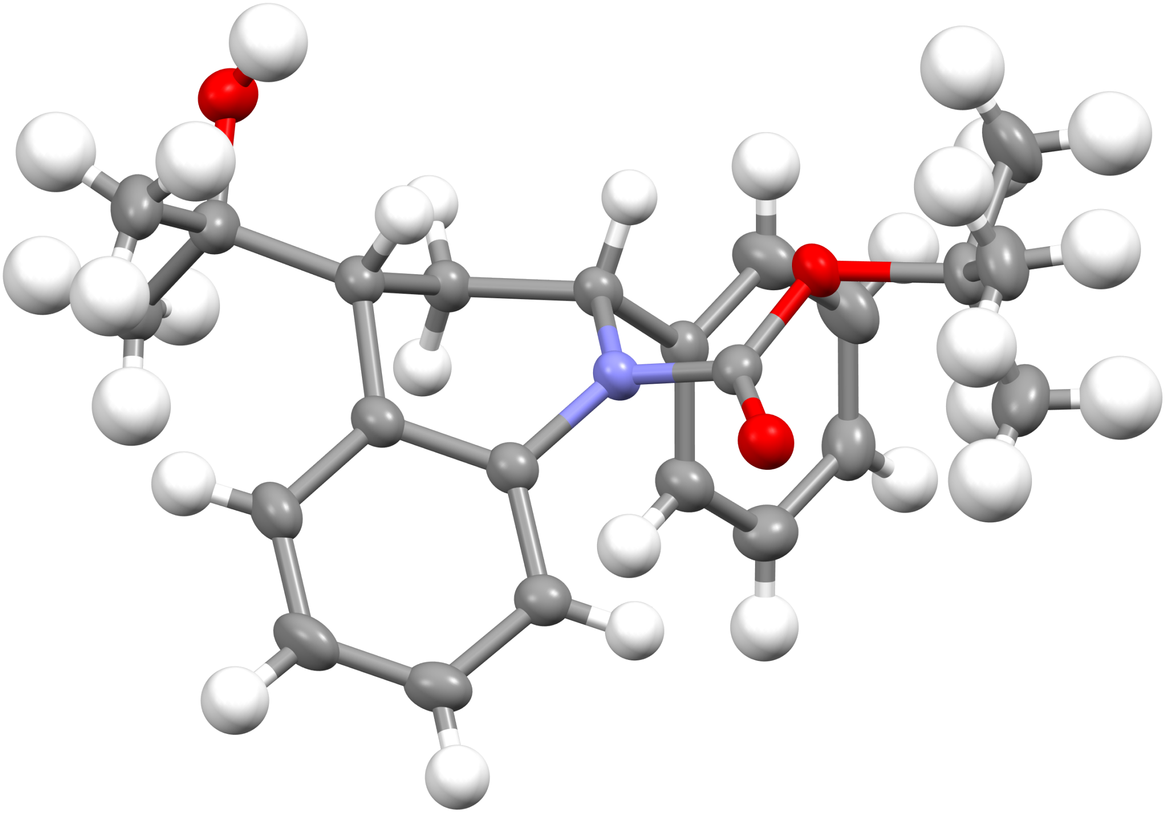

Thermal ellipsoid plot displayed at 50% probability level

CCDC 2221947

Table S28 Crystal data and structure refinement for **7** (oic325ncs_2022NCS0094r1).

Identification code	OIC325ncs_2022NCS0094r1
Empirical formula	$C_{23}H_{29}NO_3$
Formula weight	367.47
Temperature/K	100(2)
Crystal system	orthorhombic
Space group	$P2_12_12_1$
a/Å	10.1059(2)
b/Å	10.7256(2)
c/Å	37.6613(6)
α/°	90
β/°	90
γ/°	90
Volume/Å^3	4082.18(13)
Z	8
ρ_{calc}g/cm^3	1.196
μ/mm^{-1}	0.622
F(000)	1584.0
Crystal size/mm^3	0.140 × 0.060 × 0.010
Radiation	Cu Kα (λ = 1.54178)
2Θ range for data collection/°	4.692 to 140.122
Index ranges	$-12 \leq h \leq 12$, $-13 \leq k \leq 10$, $-45 \leq l \leq 45$
Reflections collected	82266
Independent reflections	7726 [R_{int} = 0.0728, R_{sigma} = 0.0260]
Data/restraints/parameters	7726/0/499
Goodness-of-fit on F^2	1.041
Final R indexes [I>=2σ (I)]	R_1 = 0.0394, wR_2 = 0.0988
Final R indexes [all data]	R_1 = 0.0431, wR_2 = 0.1007
Largest diff. peak/hole / e Å^{-3}	0.20/-0.20
Flack parameter	0.02(7)

Table S29 Fractional Atomic Coordinates (×10⁴) and Equivalent Isotropic Displacement Parameters (Å²×10³) for OIC325ncs_2022NCS0094r1. U_{eq} is defined as 1/3 of the trace of the orthogonalised U_{IJ} tensor.

Atom	*x*	*y*	*z*	U(eq)
O4	9931(2)	4250(2)	4591.8(5)	42.5(5)
O5	7186.5(19)	4529.7(17)	2915.4(5)	32.5(4)
O6	7663.8(18)	2608.8(16)	3135.0(5)	30.7(4)
N2	8792(2)	4246.4(19)	3336.9(5)	26.3(4)
C009	7833(2)	3847(2)	3108.4(6)	26.8(5)
C24	9100(3)	5541(2)	3371.6(7)	27.5(5)
C25	9152(3)	6315(2)	3076.0(7)	30.2(6)
C26	9441(3)	7578(3)	3121.2(8)	35.9(6)
C27	9691(3)	8025(3)	3455.3(8)	40.3(7)
C28	9679(3)	7245(3)	3747.1(8)	36.2(6)
C29	9385(3)	5981(2)	3713.6(7)	29.1(5)
C30	9456(3)	4991(2)	4003.2(7)	30.0(6)
C31	10331(3)	3967(2)	3839.9(7)	29.5(5)
C32	9607(3)	3318(2)	3534.2(6)	26.8(5)
C33	9898(3)	5372(3)	4381.2(7)	36.4(6)
C34	11326(3)	5849(3)	4397.6(8)	42.7(7)
C35	8910(3)	6270(3)	4550.4(8)	44.1(7)
C36	10544(3)	2658(2)	3282.4(7)	27.7(5)
C37	10634(3)	1369(3)	3285.9(8)	39.9(7)
C38	11465(3)	742(3)	3054.3(9)	43.5(7)
C39	12232(3)	1398(3)	2815.9(8)	37.2(6)
C40	12162(3)	2691(3)	2814.3(8)	39.8(7)
C41	11325(3)	3316(3)	3044.0(7)	34.6(6)
C42	7059(3)	1890(3)	2838.5(7)	33.1(6)
C43	7099(4)	568(3)	2980.9(10)	48.5(8)
C44	7931(3)	2037(3)	2516.7(8)	48.1(8)
C45	5643(3)	2279(3)	2770.4(8)	43.8(7)
O3	6885.1(18)	1753.7(17)	4173.2(5)	32.8(4)
O1	4593(2)	5471.3(19)	3064.6(5)	36.1(4)
O2	7394.4(19)	3148.4(18)	4605.4(5)	35.9(4)
N1	5777(2)	3533.7(19)	4193.3(6)	27.4(5)
C16	2530(3)	-23(3)	4243.5(10)	48.5(8)
C14	3806(3)	987(3)	3791.6(8)	41.0(7)
C12	5583(3)	7249(3)	3345.0(8)	38.3(6)
C20	7563(3)	672(3)	4339.5(8)	35.6(6)
C10	4627(3)	6157(2)	3394.5(7)	31.4(6)
C18	3565(3)	1947(3)	4356.1(9)	45.6(7)
C1	5469(3)	4759(2)	4322.9(7)	28.6(5)
C22	6979(4)	393(3)	4698.8(9)	50.0(8)
C2	5421(3)	5004(3)	4684.1(7)	33.3(6)
C11	3208(3)	6590(3)	3448.8(8)	35.3(6)

Table S29 Fractional Atomic Coordinates (×10^4) and Equivalent Isotropic Displacement Parameters (Å^2×10^3) for OIC325ncs_2022NCS0094r1. U_{eq} is defined as 1/3 of the trace of the orthogonalised U_{IJ} tensor.

Atom	*x*	*y*	*z*	U(eq)
C3	5156(3)	6204(3)	4800.1(7)	39.1(7)
C13	4095(3)	1966(2)	4018.2(7)	30.8(6)
C4	4914(3)	7136(3)	4556.6(8)	39.5(7)
C15	3007(3)	1(3)	3903.7(10)	47.4(8)
C5	4912(3)	6885(3)	4197.6(7)	34.3(6)
C17	2793(3)	951(3)	4471.5(10)	55.0(9)
C6	5186(3)	5684(2)	4069.1(7)	29.3(5)
C19	6743(3)	2831(2)	4349.6(7)	29.8(6)
C7	5102(3)	5243(2)	3687.3(7)	29.0(5)
C21	7236(4)	-351(3)	4074.3(9)	49.5(8)
C8	4244(3)	4061(2)	3705.5(7)	29.8(5)
C9	4999(3)	3014(2)	3892.4(7)	28.8(5)
C23	9042(3)	903(3)	4354.2(9)	44.5(7)

Table S30 Anisotropic Displacement Parameters (Å^2×10^3) for OIC325ncs_2022NCS0094r1. The Anisotropic displacement factor exponent takes the form: $-2\pi^2[h^2a^{*2}U_{11}+2hka^*b^*U_{12}+\ldots]$.

Atom	U_{11}	U_{22}	U_{33}	U_{23}	U_{13}	U_{12}
O4	45.4(12)	50.7(13)	31.4(10)	6.4(9)	-6.9(9)	-14.0(10)
O5	32.5(10)	31.3(10)	33.8(10)	2.3(8)	-4.1(8)	-0.9(8)
O6	36.0(10)	23.6(9)	32.4(9)	-1.6(7)	-2.8(8)	-5.3(8)
N2	29.5(11)	21.9(10)	27.5(10)	0.3(8)	-2.7(8)	-2.1(9)
C009	26.8(12)	28.9(13)	24.6(12)	-0.9(10)	1.9(10)	-0.4(11)
C24	26.7(12)	24.9(12)	31.0(13)	0.1(10)	0.6(10)	1.0(10)
C25	28.5(13)	28.9(13)	33.1(13)	2.0(11)	2.9(11)	0.6(11)
C26	35.0(14)	27.9(14)	44.8(16)	7.8(12)	7.8(12)	2.5(12)
C27	44.8(16)	20.0(13)	56.2(18)	-1.3(12)	11.0(14)	-1.5(12)
C28	39.7(15)	27.9(14)	41.0(15)	-7.5(12)	4.1(12)	-5.3(12)
C29	29.8(13)	23.8(12)	33.8(13)	-1.4(11)	4.3(10)	-0.8(11)
C30	33.6(14)	28.1(13)	28.2(13)	-1.4(11)	1.2(10)	-5.4(11)
C31	33.9(13)	25.5(12)	28.9(12)	0.7(10)	-3.0(10)	-2.1(11)
C32	29.8(13)	22.5(12)	28.2(12)	1.3(10)	-3.6(10)	-0.1(10)
C33	40.6(15)	39.2(15)	29.4(13)	-1.6(12)	-0.1(12)	-12.1(13)
C34	44.3(16)	48.4(18)	35.5(15)	-2.6(14)	-5.1(12)	-14.1(15)
C35	48.7(18)	48.1(18)	35.5(15)	-12.3(14)	5.2(13)	-11.2(14)
C36	28.9(13)	26.3(13)	27.9(13)	-1.0(10)	-4.3(10)	-1.5(10)

Table S30 Anisotropic Displacement Parameters ($Å^2×10^3$) for OIC325ncs_2022NCS0094r1. The Anisotropic displacement factor exponent takes the form: $-2\pi^2[h^2a^{*2}U_{11}+2hka^*b^*U_{12}+…]$.

Atom	U_{11}	U_{22}	U_{33}	U_{23}	U_{13}	U_{12}
C37	41.6(16)	26.4(14)	51.8(17)	1.4(12)	8.6(14)	2.5(12)
C38	43.4(17)	27.1(15)	60(2)	-8.9(13)	4.9(14)	1.3(13)
C39	30.7(14)	41.7(16)	39.2(15)	-11.1(12)	-1.5(12)	2.9(12)
C40	37.5(15)	44.0(17)	37.9(15)	3.6(13)	4.9(12)	-3.1(13)
C41	36.6(14)	28.2(14)	39.0(15)	1.4(12)	1.7(12)	-2.2(12)
C42	32.3(13)	32.9(14)	34.0(14)	-9.7(12)	-1.3(11)	-3.9(12)
C43	53.7(19)	27.6(15)	64(2)	-7.6(14)	-7.3(16)	-6.6(14)
C44	47.1(17)	58(2)	38.9(16)	-13.9(15)	5.9(13)	-4.2(16)
C45	35.2(15)	47.3(18)	48.9(17)	-15.4(14)	-3.7(13)	-5.9(14)
O3	33.5(10)	26.1(9)	38.9(10)	2.2(8)	-2.8(8)	6.3(8)
O1	41.5(11)	36.2(10)	30.6(9)	-1.8(8)	-0.7(8)	5.7(9)
O2	36.8(10)	36.6(10)	34.1(10)	2.7(8)	-6.1(8)	0.0(9)
N1	27.4(11)	24.2(11)	30.6(11)	-2.2(8)	-3.6(9)	0.0(9)
C16	35.3(16)	35.5(16)	75(2)	8.6(16)	1.3(15)	-7.4(13)
C14	46.0(17)	32.4(15)	44.4(17)	-3.6(13)	-3.0(13)	-1.9(13)
C12	43.1(16)	31.7(15)	40.2(15)	2.7(12)	4.7(13)	1.0(13)
C20	34.7(15)	28.8(14)	43.4(15)	9.0(12)	1.3(12)	7.9(12)
C10	34.6(14)	28.5(13)	31.2(13)	1.1(11)	0.7(11)	1.4(11)
C18	44.0(17)	44.3(18)	48.6(17)	-9.7(15)	9.2(14)	-11.8(15)
C1	25.6(12)	25.9(13)	34.1(13)	-2.4(10)	0.3(10)	-2.4(10)
C22	57(2)	40.5(17)	52.4(19)	15.9(15)	13.9(16)	6.9(15)
C2	35.6(14)	31.1(14)	33.3(14)	1.0(11)	3.5(11)	-2.1(12)
C11	38.1(15)	29.4(14)	38.3(15)	0.9(11)	-1.9(12)	6.4(12)
C3	48.3(17)	39.2(15)	29.8(14)	-8.1(12)	5.8(12)	-1.9(13)
C13	28.6(13)	23.9(13)	40.0(14)	0.3(11)	-4.9(11)	2.5(11)
C4	52.4(18)	25.3(14)	40.9(15)	-5.8(12)	8.2(13)	-1.3(13)
C15	44.6(17)	26.8(14)	71(2)	-5.0(15)	-6.3(16)	-4.8(14)
C5	40.0(15)	24.0(13)	39.1(14)	-1.0(11)	3.6(12)	-0.6(12)
C17	47.6(19)	60(2)	57(2)	2.5(17)	13.6(16)	-14.6(17)
C6	28.4(13)	27.7(13)	31.8(13)	-0.9(10)	0.9(10)	-1.6(11)
C19	31.6(13)	27.8(13)	29.9(13)	3.2(11)	0.7(11)	-0.1(11)
C7	28.7(13)	27.2(13)	31.3(13)	-0.7(10)	-0.2(10)	3.5(11)
C21	54(2)	29.6(15)	65(2)	-0.9(14)	-0.2(17)	11.9(14)
C8	31.3(13)	24.0(12)	34.0(13)	-1.7(11)	-5.4(11)	1.3(11)
C9	30.6(13)	26.9(13)	28.8(12)	-3.7(11)	-2.8(10)	1.4(11)
C23	32.9(15)	46.9(18)	53.8(18)	17.8(15)	1.8(13)	9.8(14)

Table S31 Bond Lengths for OIC325ncs_2022NCS0094r1.

Atom	Atom	Length/Å	Atom	Atom	Length/Å
O4	C33	1.441(3)	O3	C20	1.486(3)
O5	C009	1.221(3)	O3	C19	1.341(3)
O6	C009	1.343(3)	O1	C10	1.444(3)
O6	C42	1.488(3)	O2	C19	1.216(3)
N2	C009	1.365(3)	N1	C1	1.436(3)
N2	C24	1.429(3)	N1	C19	1.367(3)
N2	C32	1.491(3)	N1	C9	1.488(3)
C24	C25	1.389(4)	C16	C15	1.368(5)
C24	C29	1.402(4)	C16	C17	1.378(5)
C25	C26	1.396(4)	C14	C13	1.384(4)
C26	C27	1.370(4)	C14	C15	1.396(4)
C27	C28	1.381(4)	C12	C10	1.530(4)
C28	C29	1.394(4)	C20	C22	1.507(4)
C29	C30	1.524(4)	C20	C21	1.520(4)
C30	C31	1.538(4)	C20	C23	1.516(4)
C30	C33	1.547(4)	C10	C11	1.521(4)
C31	C32	1.531(3)	C10	C7	1.552(4)
C32	C36	1.516(4)	C18	C13	1.381(4)
C33	C34	1.533(4)	C18	C17	1.392(5)
C33	C35	1.527(4)	C1	C2	1.386(4)
C36	C37	1.385(4)	C1	C6	1.407(4)
C36	C41	1.389(4)	C2	C3	1.386(4)
C37	C38	1.385(4)	C3	C4	1.379(4)
C38	C39	1.380(4)	C13	C9	1.524(4)
C39	C40	1.388(4)	C4	C5	1.379(4)
C40	C41	1.383(4)	C5	C6	1.403(4)
C42	C43	1.516(4)	C6	C7	1.516(4)
C42	C44	1.507(4)	C7	C8	1.538(4)
C42	C45	1.512(4)	C8	C9	1.529(4)

Table S32 Bond Angles for OIC325ncs_2022NCS0094r1.

Atom	Atom	Atom	Angle/°	Atom	Atom	Atom	Angle/°
C009	O6	C42	120.6(2)	C19	O3	C20	120.9(2)
C009	N2	C24	121.1(2)	C1	N1	C9	119.2(2)
C009	N2	C32	119.8(2)	C19	N1	C1	120.9(2)
C24	N2	C32	118.9(2)	C19	N1	C9	119.9(2)
O5	C009	O6	124.7(2)	C15	C16	C17	120.0(3)
O5	C009	N2	124.6(2)	C13	C14	C15	120.7(3)
O6	C009	N2	110.7(2)	O3	C20	C22	110.7(2)

Table S32 Bond Angles for OIC325ncs_2022NCS0094r1.

Atom	Atom	Atom	Angle/°	Atom	Atom	Atom	Angle/°
C25	C24	N2	121.0(2)	O3	C20	C21	100.7(2)
C25	C24	C29	121.9(2)	O3	C20	C23	110.0(2)
C29	C24	N2	117.1(2)	C22	C20	C21	111.2(3)
C24	C25	C26	119.3(3)	C22	C20	C23	112.7(3)
C27	C26	C25	119.4(3)	C23	C20	C21	110.9(3)
C26	C27	C28	121.2(3)	O1	C10	C12	107.4(2)
C27	C28	C29	121.2(3)	O1	C10	C11	104.4(2)
C24	C29	C30	115.7(2)	O1	C10	C7	107.3(2)
C28	C29	C24	117.0(2)	C12	C10	C7	112.0(2)
C28	C29	C30	127.1(2)	C11	C10	C12	112.2(2)
C29	C30	C31	103.8(2)	C11	C10	C7	112.9(2)
C29	C30	C33	119.2(2)	C13	C18	C17	121.1(3)
C31	C30	C33	113.0(2)	C2	C1	N1	121.0(2)
C32	C31	C30	110.5(2)	C2	C1	C6	121.7(2)
N2	C32	C31	109.6(2)	C6	C1	N1	117.3(2)
N2	C32	C36	110.2(2)	C3	C2	C1	119.5(3)
C36	C32	C31	112.6(2)	C4	C3	C2	119.9(3)
O4	C33	C30	107.0(2)	C14	C13	C9	119.6(2)
O4	C33	C34	103.6(2)	C18	C13	C14	118.3(3)
O4	C33	C35	108.2(2)	C18	C13	C9	122.0(2)
C34	C33	C30	113.4(2)	C5	C4	C3	120.7(3)
C35	C33	C30	111.2(2)	C16	C15	C14	120.1(3)
C35	C33	C34	112.8(2)	C4	C5	C6	121.2(3)
C37	C36	C32	120.1(2)	C16	C17	C18	119.7(3)
C37	C36	C41	118.4(3)	C1	C6	C7	115.8(2)
C41	C36	C32	121.5(2)	C5	C6	C1	116.9(2)
C38	C37	C36	121.2(3)	C5	C6	C7	127.0(2)
C39	C38	C37	120.2(3)	O3	C19	N1	109.8(2)
C38	C39	C40	118.9(3)	O2	C19	O3	125.2(2)
C41	C40	C39	120.9(3)	O2	C19	N1	125.0(2)
C40	C41	C36	120.3(3)	C6	C7	C10	119.6(2)
O6	C42	C43	102.0(2)	C6	C7	C8	104.3(2)
O6	C42	C44	108.0(2)	C8	C7	C10	112.2(2)
O6	C42	C45	111.9(2)	C9	C8	C7	110.2(2)
C44	C42	C43	111.5(3)	N1	C9	C13	110.9(2)
C44	C42	C45	112.8(3)	N1	C9	C8	109.8(2)
C45	C42	C43	110.0(3)	C13	C9	C8	112.7(2)

Table S33 Torsion Angles for OIC325ncs_2022NCS0094r1.

A	B	C	D	Angle/°	A	B	C	D	Angle/°
N2	C24	C25	C26	179.2(2)	O1	C10	C7	C6	-177.6(2)
N2	C24	C29	C28	-179.7(2)	O1	C10	C7	C8	-55.0(3)
N2	C24	C29	C30	5.5(3)	N1	C1	C2	C3	178.3(3)
N2	C32	C36	C37	-131.2(3)	N1	C1	C6	C5	-178.8(2)
N2	C32	C36	C41	48.7(3)	N1	C1	C6	C7	6.0(3)
C009	O6	C42	C43	178.7(2)	C14	C13	C9	N1	-148.1(2)
C009	O6	C42	C44	61.1(3)	C14	C13	C9	C8	88.4(3)
C009	O6	C42	C45	-63.7(3)	C12	C10	C7	C6	64.8(3)
C009	N2	C24	C25	-40.5(4)	C12	C10	C7	C8	-172.6(2)
C009	N2	C24	C29	141.4(2)	C20	O3	C19	O2	19.5(4)
C009	N2	C32	C31	-164.9(2)	C20	O3	C19	N1	-162.0(2)
C009	N2	C32	C36	70.7(3)	C10	C7	C8	C9	159.7(2)
C24	N2	C009	O5	1.1(4)	C18	C13	C9	N1	30.8(4)
C24	N2	C009	O6	-176.5(2)	C18	C13	C9	C8	-92.7(3)
C24	N2	C32	C31	19.8(3)	C1	N1	C19	O3	-177.3(2)
C24	N2	C32	C36	-104.7(3)	C1	N1	C19	O2	1.2(4)
C24	C25	C26	C27	1.2(4)	C1	N1	C9	C13	-109.0(2)
C24	C29	C30	C31	47.4(3)	C1	N1	C9	C8	16.2(3)
C24	C29	C30	C33	174.1(2)	C1	C2	C3	C4	1.5(4)
C25	C24	C29	C28	2.2(4)	C1	C6	C7	C10	172.5(2)
C25	C24	C29	C30	-172.6(2)	C1	C6	C7	C8	46.1(3)
C25	C26	C27	C28	0.8(4)	C2	C1	C6	C5	2.8(4)
C26	C27	C28	C29	-1.4(5)	C2	C1	C6	C7	-172.3(2)
C27	C28	C29	C24	-0.1(4)	C2	C3	C4	C5	0.9(5)
C27	C28	C29	C30	174.0(3)	C11	C10	C7	C6	-63.1(3)
C28	C29	C30	C31	-126.8(3)	C11	C10	C7	C8	59.5(3)
C28	C29	C30	C33	-0.1(4)	C3	C4	C5	C6	-1.5(5)
C29	C24	C25	C26	-2.8(4)	C13	C14	C15	C16	-1.8(5)
C29	C30	C31	C32	-68.9(3)	C13	C18	C17	C16	-1.3(5)
C29	C30	C33	O4	-177.4(2)	C4	C5	C6	C1	-0.4(4)
C29	C30	C33	C34	-63.8(3)	C4	C5	C6	C7	174.2(3)
C29	C30	C33	C35	64.6(3)	C15	C16	C17	C18	-1.1(5)
C30	C31	C32	N2	36.1(3)	C15	C14	C13	C18	-0.6(4)
C30	C31	C32	C36	159.2(2)	C15	C14	C13	C9	178.4(3)
C31	C30	C33	O4	-55.1(3)	C5	C6	C7	C10	-2.1(4)
C31	C30	C33	C34	58.5(3)	C5	C6	C7	C8	-128.5(3)
C31	C30	C33	C35	-173.1(2)	C17	C16	C15	C14	2.6(5)
C31	C32	C36	C37	106.1(3)	C17	C18	C13	C14	2.1(5)
C31	C32	C36	C41	-74.0(3)	C17	C18	C13	C9	-176.9(3)
C32	N2	C009	O5	-174.1(2)	C6	C1	C2	C3	-3.4(4)
C32	N2	C009	O6	8.2(3)	C6	C7	C8	C9	-69.4(3)
C32	N2	C24	C25	134.8(2)	C19	O3	C20	C22	51.8(3)
C32	N2	C24	C29	-43.3(3)	C19	O3	C20	C21	169.4(2)

Table S33 Torsion Angles for OIC325ncs_2022NCS0094r1.

A	B	C	D	Angle/°	A	B	C	D	Angle/°
C32	C36	C37	C38	178.8(3)	C19	O3	C20	C23	-73.5(3)
C32	C36	C41	C40	-179.3(2)	C19	N1	C1	C2	-40.8(4)
C33	C30	C31	C32	160.5(2)	C19	N1	C1	C6	140.8(3)
C36	C37	C38	C39	0.6(5)	C19	N1	C9	C13	69.0(3)
C37	C36	C41	C40	0.6(4)	C19	N1	C9	C8	-165.8(2)
C37	C38	C39	C40	0.4(5)	C7	C8	C9	N1	38.6(3)
C38	C39	C40	C41	-0.9(5)	C7	C8	C9	C13	162.8(2)
C39	C40	C41	C36	0.4(5)	C9	N1	C1	C2	137.2(3)
C41	C36	C37	C38	-1.0(5)	C9	N1	C1	C6	-41.2(3)
C42	O6	C009	O5	24.3(4)	C9	N1	C19	O3	4.7(3)
C42	O6	C009	N2	-158.1(2)	C9	N1	C19	O2	-176.8(2)

Table S34 Hydrogen Atom Coordinates (Å×10^4) and Isotropic Displacement Parameters (Å^2×10^3) for OIC325ncs_2022NCS0094r1.

Atom	*x*	*y*	*z*	U(eq)
H4	9167.75	3942.88	4602.88	64
H25	8993.27	5987.4	2845.47	36
H26	9463.01	8121.8	2922.32	43
H27	9875.54	8885.92	3486.75	48
H28	9875.79	7576.96	3975.02	43
H30	8547.41	4631.07	4026.14	36
H31A	11162.96	4340.15	3750.75	35
H31B	10561.23	3345.86	4024.39	35
H32	8997.03	2681.21	3638.09	32
H34A	11430.21	6553.62	4234.26	64
H34B	11933.08	5178.42	4328.96	64
H34C	11528.72	6120.71	4640.05	64
H35A	9080.68	6326.36	4806.01	66
H35B	8007.98	5963.62	4510.99	66
H35C	9006.15	7096.84	4442.67	66
H37	10116.77	907.05	3450.31	48
H38	11505.88	-142.46	3059.64	52
H39	12799.59	972.16	2655.39	45
H40	12695.56	3151.69	2653.36	48
H41	11284.67	4200.96	3038.56	42
H43A	8021.06	303.13	3007.9	73
H43B	6645.62	9.86	2814.59	73
H43C	6654.7	537.6	3212.04	73
H44A	7877.98	2897.82	2430.28	72
H44B	7634.89	1466.3	2329.78	72
H44C	8848.57	1843.73	2580.67	72

Table S34 Hydrogen Atom Coordinates (Å×10^4) and Isotropic Displacement Parameters (Å^2×10^3) for OIC325ncs_2022NCS0094r1.

Atom	*x*	*y*	*z*	U(eq)
H45A	5151.69	2280.33	2994.77	66
H45B	5229.84	1690.3	2605.04	66
H45C	5629.34	3117.51	2667.22	66
H1	5367.48	5274.28	3005.81	54
H16	2016.3	-711.15	4322.42	58
H14	4154.34	986.62	3557.36	49
H12A	6490.04	6930.94	3326.91	57
H12B	5516.68	7812.42	3549	57
H12C	5356.08	7701.75	3127.44	57
H18	3728.9	2624.31	4512.44	55
H22A	7228.46	1051.57	4866.15	75
H22B	7315	-410.19	4784.16	75
H22C	6012.43	355.04	4679.77	75
H2	5568.57	4353.59	4850.79	40
H11A	2987.56	7222.31	3270.43	53
H11B	3115.02	6949.31	3686.78	53
H11C	2607.17	5878.31	3424.18	53
H3	5142.42	6384.77	5047.07	47
H4A	4745.53	7960.89	4637.03	47
H15	2794.12	-653.73	3743.78	57
H5	4721.57	7535.92	4034.36	41
H17	2450.3	942.54	4706.34	66
H7	6012.18	4968.21	3618.39	35
H21A	6274.33	-455.11	4060.33	74
H21B	7642.49	-1134.93	4151.42	74
H21C	7581.24	-121.48	3839.99	74
H8A	3417.33	4242.51	3836.62	36
H8B	4003.72	3793.81	3462.26	36
H9	5638.24	2651.46	3717.94	35
H23A	9227.21	1586.39	4519.56	67
H23B	9363.76	1125.64	4117.06	67
H23C	9492.16	145.25	4435.52	67

X-ray for compound 8a

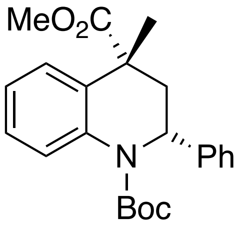

8a

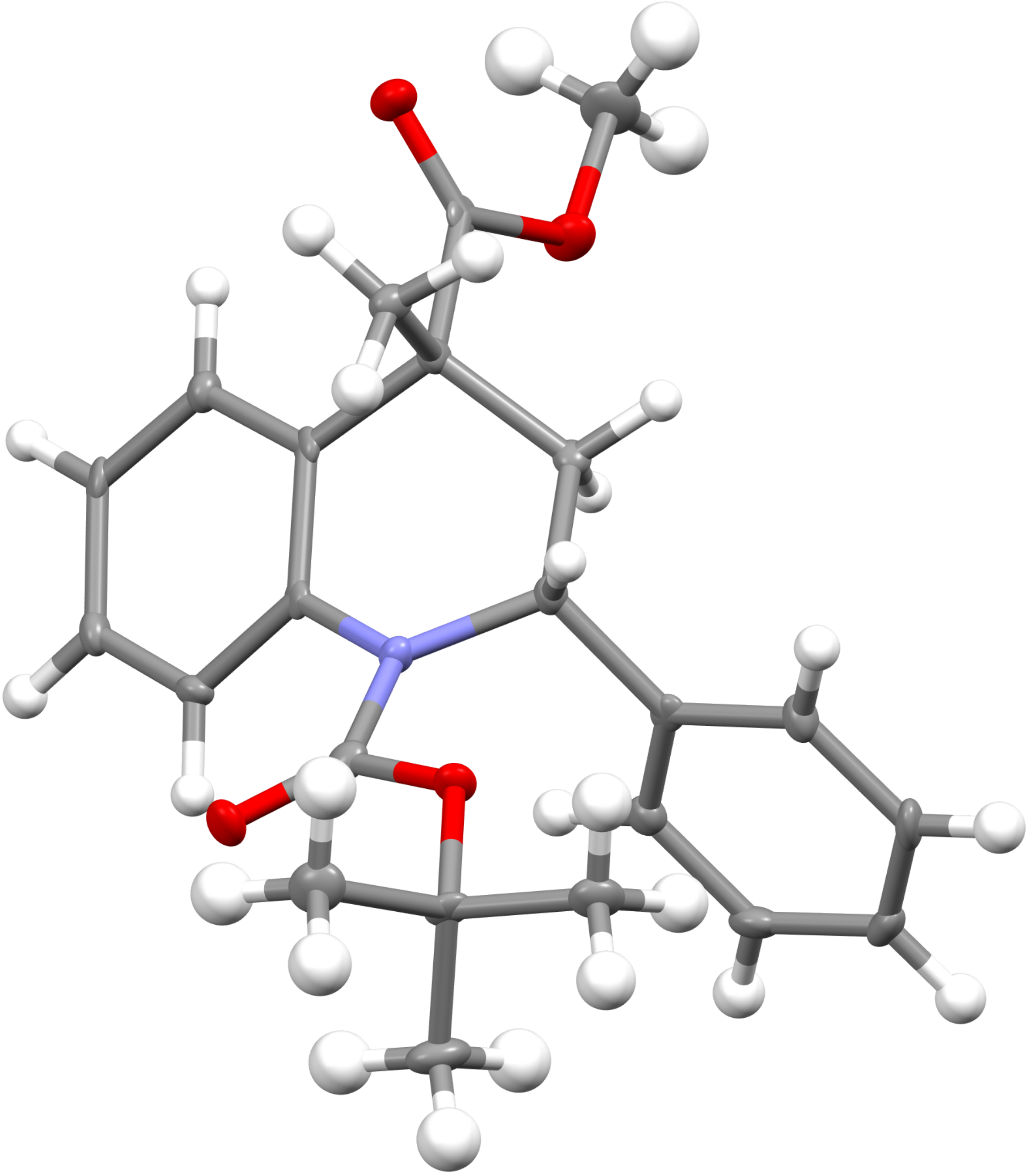

Thermal ellipsoid plot displayed at 50% probability level

CCDC 2221948

Table S35 Crystal data and structure refinement for **8a** (oic324ncs_2022NCS0093s).

Identification code	OIC324ncs_2022NCS0093s
Empirical formula	$C_{23}H_{27}NO_4$
Formula weight	381.45
Temperature/K	100(2)
Crystal system	monoclinic
Space group	$P2_1/c$
a/Å	16.5540(2)
b/Å	10.47210(10)
c/Å	11.48900(10)
α/°	90
β/°	88.2370(10)
γ/°	90
Volume/Å^3	1990.73(4)
Z	4
ρ_{calc}g/cm^3	1.273
μ/mm^{-1}	0.698
F(000)	816.0
Crystal size/mm^3	0.19 × 0.02 × 0.015
Radiation	Cu Kα (λ = 1.54178)
2Θ range for data collection/°	5.34 to 140.068
Index ranges	$-20 \leq h \leq 20$, $-12 \leq k \leq 12$, $-10 \leq l \leq 13$
Reflections collected	44743
Independent reflections	3760 [R_{int} = 0.0281, R_{sigma} = 0.0133]
Data/restraints/parameters	3760/0/258
Goodness-of-fit on F^2	1.044
Final R indexes [I>=2σ (I)]	R_1 = 0.0319, wR_2 = 0.0835
Final R indexes [all data]	R_1 = 0.0350, wR_2 = 0.0859
Largest diff. peak/hole / e Å^{-3}	0.20/-0.21

Table S36 Fractional Atomic Coordinates (×10[4]) and Equivalent Isotropic Displacement Parameters (Å[2]×10[3]) for oic324ncs_2022NCS0093s. U_{eq} is defined as 1/3 of the trace of the orthogonalised U_{IJ} tensor.

Atom	*x*	*y*	*z*	U(eq)
O1	4372.1(5)	2062.7(8)	7699.4(7)	28.9(2)
O2	4671.7(5)	3450.9(8)	6269.7(7)	27.13(19)
O3	8606.2(5)	1411.7(7)	7152.6(7)	24.44(18)
O4	8490.3(4)	3453.1(7)	7816.6(6)	21.23(18)
N1	7452.8(5)	2607.7(8)	6904.1(8)	17.99(19)
C1	6996.5(6)	1595.4(10)	6414.1(9)	17.4(2)
C2	7361.8(6)	681.7(10)	5689.7(9)	20.6(2)
C3	6910.6(7)	-327.2(10)	5268.2(9)	21.7(2)
C4	6095.3(7)	-436.4(10)	5561.9(9)	21.1(2)
C5	5723.6(6)	504.3(10)	6239.9(9)	19.0(2)
C6	6159.6(6)	1535.3(10)	6661.2(8)	16.9(2)
C7	5775.3(6)	2643.6(10)	7336.0(9)	17.9(2)
C8	6185.3(6)	3853.6(10)	6836.5(9)	18.0(2)
C9	7086.8(6)	3890.0(10)	7082.3(9)	17.8(2)
C10	4863.1(6)	2673.5(10)	7140.5(9)	19.5(2)
C11	3816.7(7)	3593.5(13)	6065.1(12)	31.9(3)
C12	5899.3(7)	2478.3(11)	8649.1(9)	22.4(2)
C13	7512.2(6)	4885.3(10)	6321.5(9)	18.5(2)
C14	7625.5(7)	6115.0(11)	6743.9(10)	22.6(2)
C15	8006.5(7)	7039.0(11)	6055.0(11)	26.6(3)
C16	8270.5(7)	6742.9(12)	4929.5(11)	27.0(3)
C17	8155.2(7)	5523.0(12)	4493.8(10)	26.1(2)
C18	7777.9(6)	4600.0(11)	5188.9(10)	22.1(2)
C19	8228.9(6)	2398.6(10)	7279.3(9)	18.9(2)
C20	9348.1(6)	3599.1(11)	8117.4(9)	21.7(2)
C21	9369.4(7)	4959.7(12)	8577.0(10)	27.2(3)
C22	9884.3(7)	3481.6(13)	7026.3(10)	30.3(3)
C23	9560.1(7)	2645.3(12)	9052.8(10)	29.0(3)

Table S37 Anisotropic Displacement Parameters (Å^2×10^3) for oic324ncs_2022NCS0093s. The Anisotropic displacement factor exponent takes the form: $-2\pi^2[h^2a^{*2}U_{11}+2hka^*b^*U_{12}+\ldots]$.

Atom	U_{11}	U_{22}	U_{33}	U_{23}	U_{13}	U_{12}
O1	20.6(4)	30.1(4)	35.7(5)	5.2(4)	3.9(3)	-2.6(3)
O2	18.4(4)	33.8(5)	29.5(4)	7.9(3)	-5.9(3)	-1.0(3)
O3	20.6(4)	23.5(4)	29.5(4)	-0.6(3)	-4.2(3)	3.7(3)
O4	16.4(4)	23.5(4)	24.1(4)	-2.0(3)	-4.5(3)	-0.6(3)
N1	16.5(4)	17.7(4)	19.9(4)	-0.7(3)	-2.3(3)	0.7(3)
C1	19.3(5)	18.2(5)	14.8(5)	2.1(4)	-2.4(4)	-0.2(4)
C2	19.3(5)	23.1(5)	19.4(5)	1.0(4)	0.1(4)	2.6(4)
C3	25.7(5)	21.0(5)	18.3(5)	-2.4(4)	-0.6(4)	3.9(4)
C4	25.6(5)	19.2(5)	18.8(5)	-0.3(4)	-3.4(4)	-1.7(4)
C5	19.0(5)	21.0(5)	16.9(5)	2.1(4)	-0.9(4)	-0.9(4)
C6	19.0(5)	18.4(5)	13.3(5)	2.3(4)	-0.6(4)	1.0(4)
C7	17.6(5)	19.2(5)	16.8(5)	-0.5(4)	0.0(4)	0.0(4)
C8	16.7(5)	18.6(5)	18.6(5)	-0.4(4)	0.0(4)	1.3(4)
C9	17.7(5)	18.1(5)	17.7(5)	-1.9(4)	-0.6(4)	1.2(4)
C10	19.8(5)	18.2(5)	20.6(5)	-4.2(4)	0.7(4)	0.6(4)
C11	20.2(6)	35.5(7)	40.6(7)	1.6(5)	-10.3(5)	0.9(5)
C12	24.6(5)	26.1(6)	16.4(5)	-0.6(4)	0.8(4)	-0.4(4)
C13	13.5(5)	21.0(5)	21.3(5)	1.1(4)	-3.2(4)	1.5(4)
C14	23.1(5)	21.9(5)	22.8(5)	-0.8(4)	-2.0(4)	1.4(4)
C15	26.1(6)	20.4(5)	33.6(6)	1.4(5)	-5.2(5)	-1.2(4)
C16	21.0(5)	27.4(6)	32.5(6)	8.6(5)	0.3(5)	-2.9(4)
C17	21.4(5)	32.3(6)	24.2(6)	2.2(5)	3.6(4)	0.4(5)
C18	19.5(5)	23.1(5)	23.7(5)	-1.6(4)	0.1(4)	-0.5(4)
C19	18.1(5)	21.8(5)	16.7(5)	1.5(4)	-0.6(4)	-0.8(4)
C20	15.1(5)	29.7(6)	20.5(5)	0.1(4)	-3.4(4)	-3.4(4)
C21	22.9(5)	31.8(6)	27.1(6)	-2.6(5)	-3.7(4)	-4.9(5)
C22	24.3(6)	41.5(7)	24.7(6)	-3.6(5)	3.2(5)	-7.3(5)
C23	24.9(6)	36.6(7)	25.9(6)	4.1(5)	-7.6(5)	0.7(5)

Table S38 Bond Lengths for oic324ncs_2022NCS0093s.

Atom	Atom	Length/Å	Atom	Atom	Length/Å
O1	C10	1.2042(14)	C6	C7	1.5240(14)
O2	C10	1.3354(14)	C7	C8	1.5399(14)
O2	C11	1.4497(13)	C7	C10	1.5339(14)
O3	C19	1.2140(13)	C7	C12	1.5385(14)
O4	C19	1.3432(13)	C8	C9	1.5278(14)
O4	C20	1.4797(12)	C9	C13	1.5194(14)
N1	C1	1.4275(13)	C13	C14	1.3908(15)
N1	C9	1.4847(13)	C13	C18	1.3931(15)
N1	C19	1.3851(13)	C14	C15	1.3891(16)
C1	C2	1.3939(15)	C15	C16	1.3873(18)
C1	C6	1.4069(14)	C16	C17	1.3874(17)
C2	C3	1.3896(15)	C17	C18	1.3898(16)
C3	C4	1.3858(16)	C20	C21	1.5204(16)
C4	C5	1.3879(15)	C20	C22	1.5186(15)
C5	C6	1.3938(15)	C20	C23	1.5162(16)

Table S39 Bond Angles for oic324ncs_2022NCS0093s.

Atom	Atom	Atom	Angle/°	Atom	Atom	Atom	Angle/°
C10	O2	C11	116.07(9)	N1	C9	C13	111.17(8)
C19	O4	C20	121.15(8)	C13	C9	C8	110.42(8)
C1	N1	C9	120.49(8)	O1	C10	O2	123.47(10)
C19	N1	C1	120.83(9)	O1	C10	C7	124.23(10)
C19	N1	C9	118.59(8)	O2	C10	C7	112.30(9)
C2	C1	N1	121.34(9)	C14	C13	C9	119.97(9)
C2	C1	C6	119.75(9)	C14	C13	C18	118.84(10)
C6	C1	N1	118.90(9)	C18	C13	C9	121.17(10)
C3	C2	C1	120.13(10)	C15	C14	C13	120.66(10)
C4	C3	C2	120.52(10)	C16	C15	C14	119.99(11)
C3	C4	C5	119.32(10)	C15	C16	C17	119.95(11)
C4	C5	C6	121.29(10)	C16	C17	C18	119.83(11)
C1	C6	C7	117.50(9)	C17	C18	C13	120.73(10)
C5	C6	C1	118.80(9)	O3	C19	O4	125.67(9)
C5	C6	C7	123.65(9)	O3	C19	N1	125.18(10)
C6	C7	C8	105.35(8)	O4	C19	N1	109.15(9)
C6	C7	C10	109.78(8)	O4	C20	C21	102.16(8)
C6	C7	C12	110.30(8)	O4	C20	C22	109.86(9)
C10	C7	C8	110.69(8)	O4	C20	C23	110.01(9)
C10	C7	C12	107.91(8)	C22	C20	C21	110.08(10)
C12	C7	C8	112.79(9)	C23	C20	C21	111.28(9)
C9	C8	C7	111.93(8)	C23	C20	C22	112.93(10)
N1	C9	C8	110.41(8)				

Table S40 Torsion Angles for oic324ncs_2022NCS0093s.

A	B	C	D	Angle/°	A	B	C	D	Angle/°
N1	C1	C2	C3	177.00(9)	C8	C9	C13	C14	93.97(11)
N1	C1	C6	C5	-176.19(9)	C8	C9	C13	C18	-84.53(12)
N1	C1	C6	C7	6.43(14)	C9	N1	C1	C2	145.56(10)
N1	C9	C13	C14	-143.13(9)	C9	N1	C1	C6	-33.69(13)
N1	C9	C13	C18	38.38(13)	C9	N1	C19	O3	-177.45(10)
C1	N1	C9	C8	7.81(12)	C9	N1	C19	O4	3.36(12)
C1	N1	C9	C13	-115.10(10)	C9	C13	C14	C15	-179.38(10)
C1	N1	C19	O3	5.92(16)	C9	C13	C18	C17	178.98(9)
C1	N1	C19	O4	-173.27(8)	C10	C7	C8	C9	176.58(8)
C1	C2	C3	C4	0.00(16)	C11	O2	C10	O1	3.75(16)
C1	C6	C7	C8	40.12(11)	C11	O2	C10	C7	-176.68(9)
C1	C6	C7	C10	159.33(9)	C12	C7	C8	C9	55.56(11)
C1	C6	C7	C12	-81.88(11)	C12	C7	C10	O1	-34.61(14)
C2	C1	C6	C5	4.56(15)	C12	C7	C10	O2	145.82(9)
C2	C1	C6	C7	-172.82(9)	C13	C14	C15	C16	0.69(17)
C2	C3	C4	C5	2.89(16)	C14	C13	C18	C17	0.47(15)
C3	C4	C5	C6	-2.03(15)	C14	C15	C16	C17	-0.14(17)
C4	C5	C6	C1	-1.68(15)	C15	C16	C17	C18	-0.23(17)
C4	C5	C6	C7	175.53(9)	C16	C17	C18	C13	0.06(16)
C5	C6	C7	C8	-137.13(10)	C18	C13	C14	C15	-0.85(16)
C5	C6	C7	C10	-17.91(13)	C19	O4	C20	C21	174.26(9)
C5	C6	C7	C12	100.88(11)	C19	O4	C20	C22	57.43(12)
C6	C1	C2	C3	-3.76(15)	C19	O4	C20	C23	-67.48(12)
C6	C7	C8	C9	-64.81(10)	C19	N1	C1	C2	-37.88(14)
C6	C7	C10	O1	85.64(12)	C19	N1	C1	C6	142.88(10)
C6	C7	C10	O2	-93.93(10)	C19	N1	C9	C8	-168.83(9)
C7	C8	C9	N1	41.65(11)	C19	N1	C9	C13	68.25(11)
C7	C8	C9	C13	164.99(8)	C20	O4	C19	O3	12.15(15)
C8	C7	C10	O1	-158.48(10)	C20	O4	C19	N1	-168.67(8)
C8	C7	C10	O2	21.95(12)					

Table S41 Hydrogen Atom Coordinates (Å×10^4) and Isotropic Displacement Parameters (Å^2×10^3) for oic324ncs_2022NCS0093s.

**Atom**	***x***	***y***	***z***	**U(eq)**
H2	7908.65	747.38	5488.08	25
H3	7157.69	-933.93	4785.25	26
H4	5800.23	-1132.9	5307.31	25
H5	5172.78	445.53	6416.53	23
H8A	6115.11	3882.54	6001.84	22
H8B	5925.28	4601.51	7176.42	22
H9	7147.63	4132.78	7898.69	21
H11A	3747.18	4027.95	5339.26	48
H11B	3567.67	4081.15	6686.12	48
H11C	3568.88	2765.83	6034.53	48
H12A	5590.54	1760.21	8931.06	34
H12B	5723.01	3235.85	9053.05	34
H12C	6461.83	2335.75	8782.4	34
H14	7444.4	6320.68	7494.9	27
H15	8084.53	7855.97	6348.33	32
H16	8524.49	7361.7	4467.32	32
H17	8329.95	5323.41	3738.49	31
H18	7702	3782.82	4894.75	27
H21A	9180.47	5534.15	7993.78	41
H21B	9913.63	5178.99	8762.64	41
H21C	9027.68	5025.13	9264.73	41
H22A	9867.2	2619.94	6743.01	45
H22B	10429.98	3698.75	7205.93	45
H22C	9694.86	4052.02	6440.18	45
H23A	9176.74	2712.86	9693.91	43
H23B	10092.67	2822.37	9318.83	43
H23C	9545.18	1796.83	8737.81	43

5. References

47. Bruker (2017) Apex3 v2017.3-0. Bruker AXS Inc., Madison, Wisconsin, USA.
48. L. Krause, R. Herbst-Irmer, G. M. Sheldrick, D. Stalke, *J. Appl. Cryst.*, **2015**, *48*, 3.
49. G. M. Sheldrick, *Acta Cryst.*, **2015**, *A71*, 3.
50. G. M. Sheldrick, *Acta Cryst.*, **2015**, *C71*, 3.
51. O.V. Dolomanov, L. J. Bourhis, R. J. Gildea, J. A. K. Howard, H. Puschmann, *J. Appl. Cryst.*, **2009**, *42*, 339.
52. M. J. Frisch, G. W. Trucks, H. B. Schlegel, G. E. Scuseria, M. A. Robb, J. R. Cheeseman, G. Scalmani, V. Barone, B. Mennucci, G. A. Petersson, H. Nakatsuji, M. Caricato, X. Li, H. P. Hratchian, A. F. Izmaylov, J. Bloino, G. Zheng, J. L. Sonnenberg, M. Hada, M. Ehara, K. Toyota, R. Fukuda, J. Hasegawa, M. Ishida, T. Nakajima, Y. Honda, O. Kitao, H. Nakai, T. Vreven, J. A. Montgomery Jr, J. E. Peralta, F. Ogliaro, M. Bearpark, J. J. Heyd, E. Brothers, K. N. Kudin, V. N. Staroverov, R. Kobayashi, J. Normand, K. Raghavachari, A. Rendell, J. C. Burant, S. S. Iyengar, J. Tomasi, M. Cossi, N. Rega, J. M. Millam, M. Klene, J. E. Knox, J. B. Cross, V. Bakken, C. Adamo, J. Jaramillo, R. Gomperts, R. E. Stratmann, O. Yazyev, A. J. Austin, R. Cammi, C. Pomelli, J. W. Ochterski, R. L. Martin, K. Morokuma, V. G. Zakrzewski, G. A. Voth, P. Salvador, J. J. Dannenberg, S. Dapprich, A. D. Daniels, Ö. Farkas, J. B. Foresman, J. V. Ortiz, J. Cioslowski, D. J. Fox, *Gaussian 09, Revision D.01*, Gaussian, Inc., Wallingford CT, 2009.
53. *a*) A. D. McLean, G. S. Chandler, *J. Chem. Phys.* **1980**, *72*, 5639; *b*) K. Raghavachari, J. S. Binkley, R. Seeger, J. A. Pople, *J. Chem. Phys.* **1980**, *72*, 650.
54. *a*) G. Scalmani, M. J. Frisch, *J. Chem. Phys.* **2010**, *132*, 114110; *b*) M. Cossi, N. Rega, G. Scalmani, V. Barone, *J. Comput. Chem.* **2003**, *24*, 669 and references therein.
55. F. Kelleher, R. T. Lewis *UK Patent GB2269590A* **1994**.
56. P. Molina, M. Alajarín, P. Sánchez-Andrada, *Synthesis* **1993**, 225.
57. J. H. Ferguson, Z. D. L. Santos, S. N. Devi, S. Kaluz, E. G. V. Meir, S. K. Zingales, B. Wang, *J. Enzyme Inhib. Med. Chem.* **2017**, *32*, 992.

6. HPLC traces

For specific HPLC columns and conditions, see experimental above.

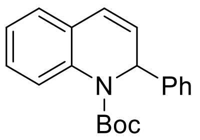

	R_t/ min	Area/ mV.s	Area/ %
1	5.560	8277.832	50
2	6.217	8286.300	50
	total	16564.132	100

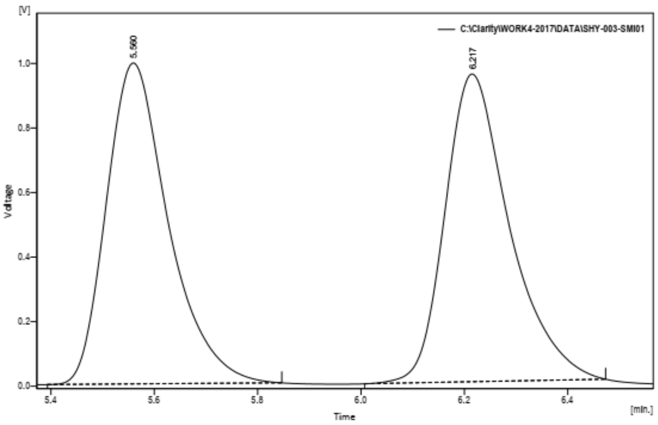

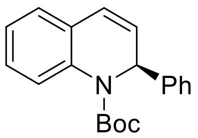

	R_t/ min	Area/ mV.s	Area/ %
1	5.427	11671.573	99
2	6.187	103.751	1
	total	11775.325	100

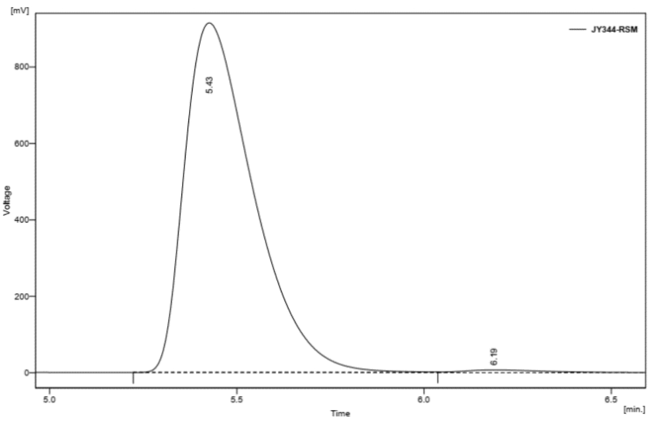

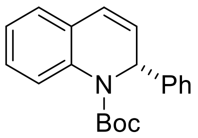

	R_t/ min	Area/ mV.s	Area/ %
1	5.537	205.387	3
2	6.463	7620.844	97
	total	10651.317	100

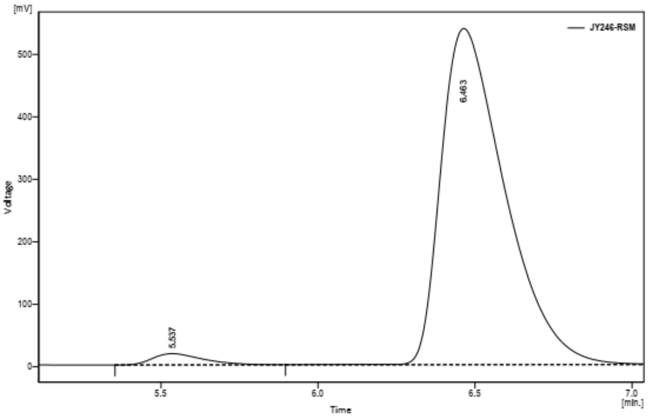

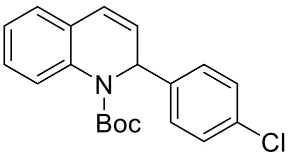

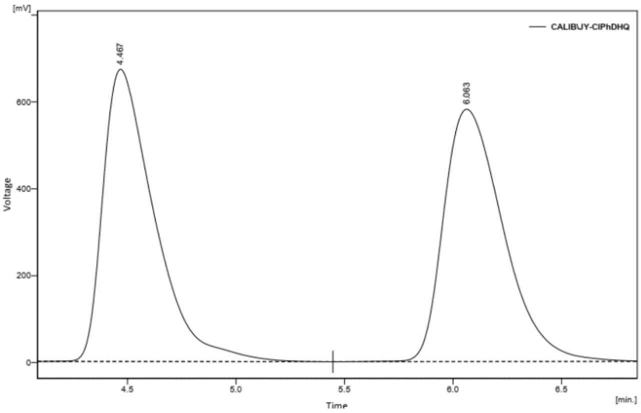

	R_t/ min	Area/ mV.s	Area/ %
1	4.467	11048.992	50
2	6.063	11053.724	50
	total	22102.716	100

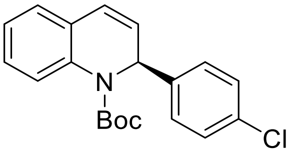

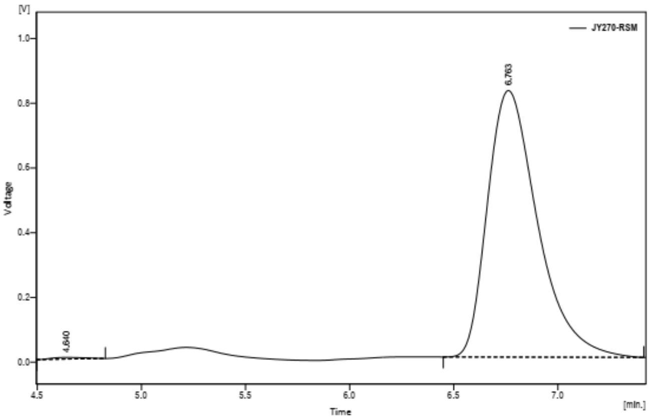

	R_t/ min	Area/ mV.s	Area/ %
1	4.640	59.715	1
2	6.763	13788.622	99
	total	13848.337	100

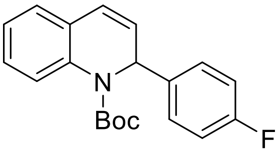

	R_t/ min	Area/ mV.s	Area/ %
1	4.290	8636.265	49.3
2	5.417	8882.653	50.7
	total	17518.919	100.0

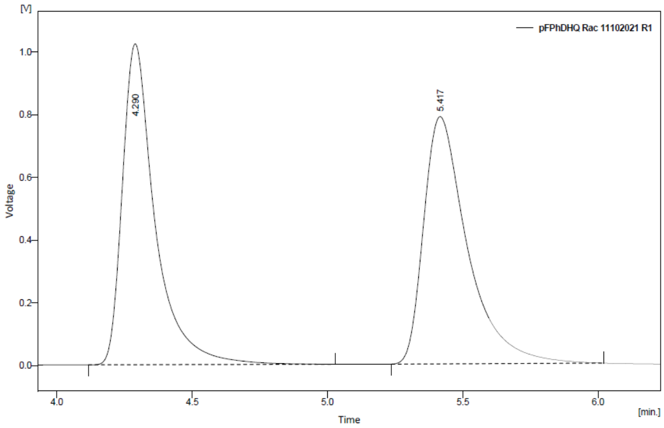

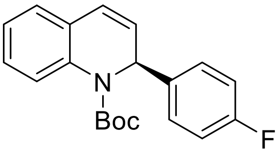

	R_t/ min	Area/ mV.s	Area/ %
1	4.293	412.602	4.9
2	5.440	8048.617	95.1
	Total	8461.219	100.0

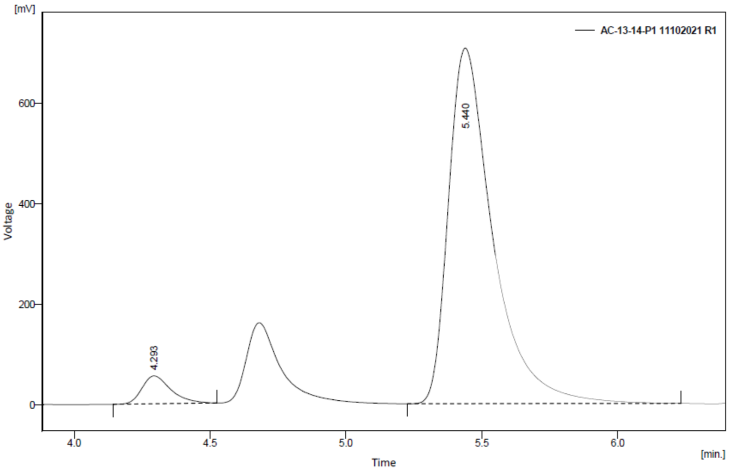

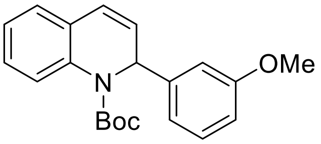

	R_t/ min	Area/ mV.s	Area/ %
1	7.550	8671.531	50
2	8.850	8681.147	50
	total	17352.678	100

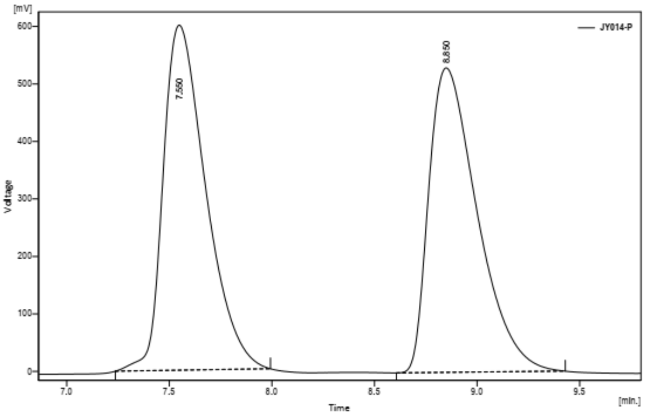

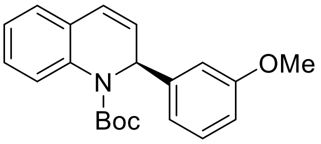

	R_t/ min	Area/ mV.s	Area/ %
1	7.380	7914.832	98
2	9.203	134.624	2
	total	8049.456	100

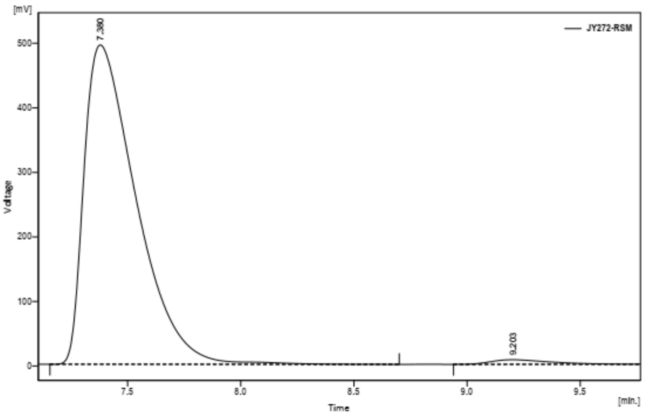

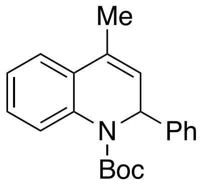

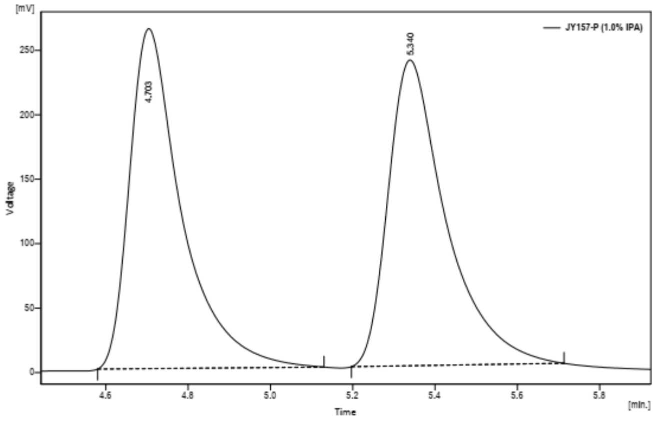

	R_t/ min	Area/ mV.s	Area/ %
1	4.703	2319.974	50
2	5.340	2315.800	50
	total	4635.774	100

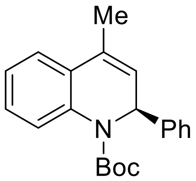

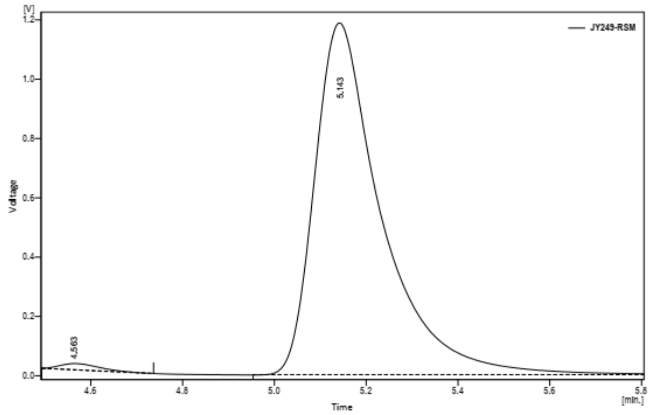

	R_t/ min	Area/ mV.s	Area/ %
1	4.563	128.597	1
2	5.143	12080.304	99
	total	12208.900	100

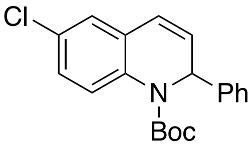

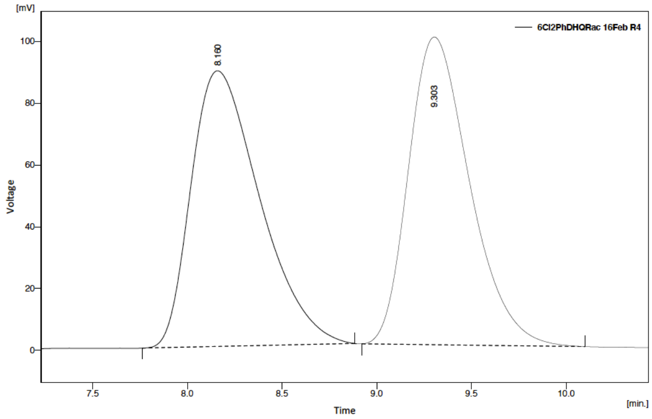

	R_t/ min	Area/ mV.s	Area/ %
1	8.160	2249.118	50.1
2	9.303	2236.446	49.9
	Total	4485.563	100.0

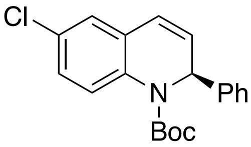

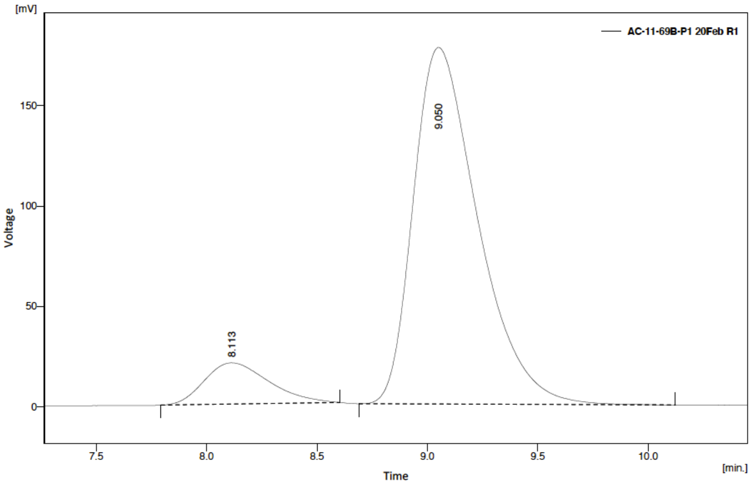

	R_t/ min	Area/ mV.s	Area/ %
1	8.113	417.366	10.4
2	9.050	3611.679	89.6
	Total	4029.045	100.0

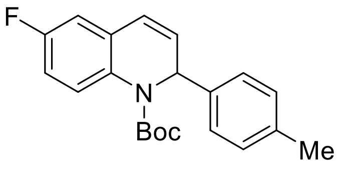

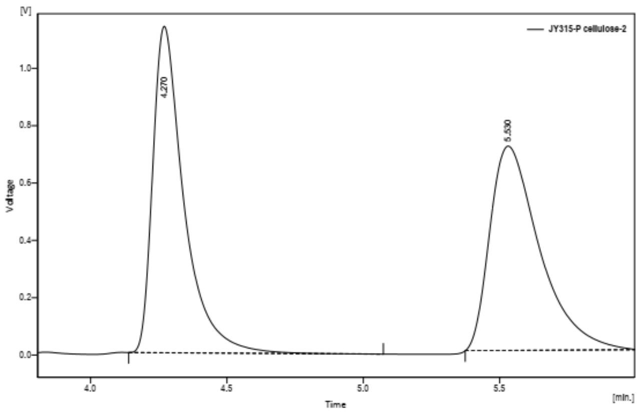

	R_t/ min	Area/ mV.s	Area/ %
1	4.270	9064.415	50
2	5.530	9054.962	50
	total	18119.376	100

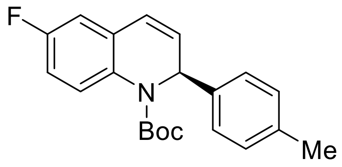

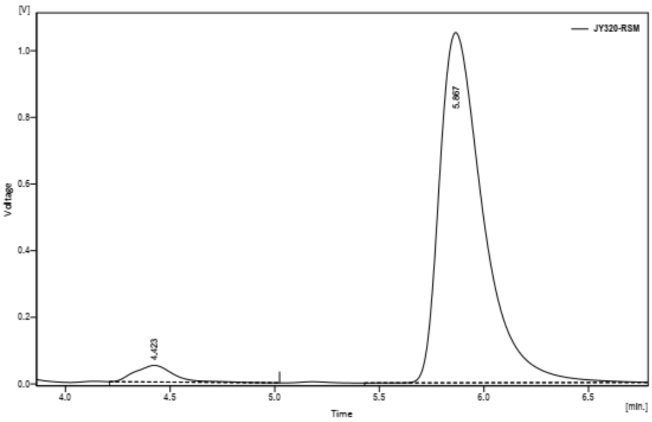

	R_t/ min	Area/ mV.s	Area/ %
1	4.423	644.842	4
2	5.867	15219.498	96
	total	15864.340	100

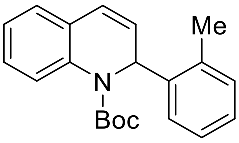

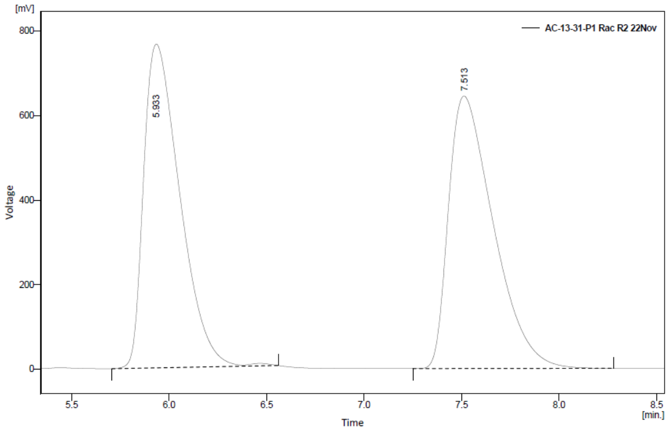

	R_t/ min	Area/ mV.s	Area/ %
1	5.933	9904.209	48.9
2	7.513	10333.237	51.1
	Total	20237.446	100.0

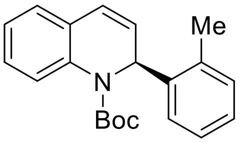

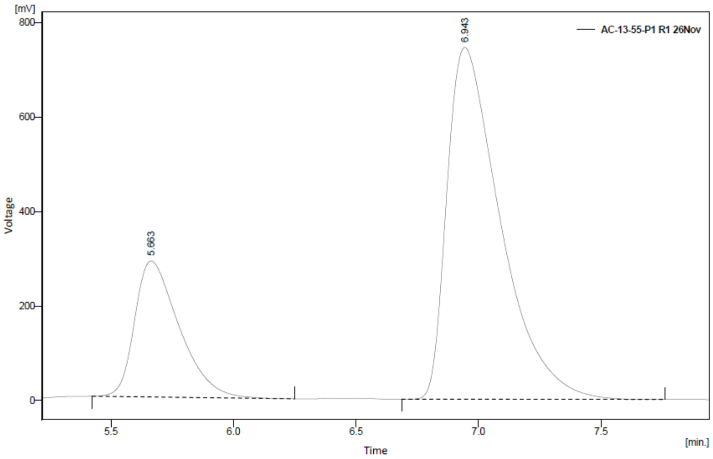

	R_t/ min	Area/ mV.s	Area/ %
1	5.663	3640.159	24.4
2	6.943	11300.275	75.6
	Total	14940.434	100.0

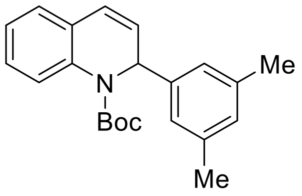

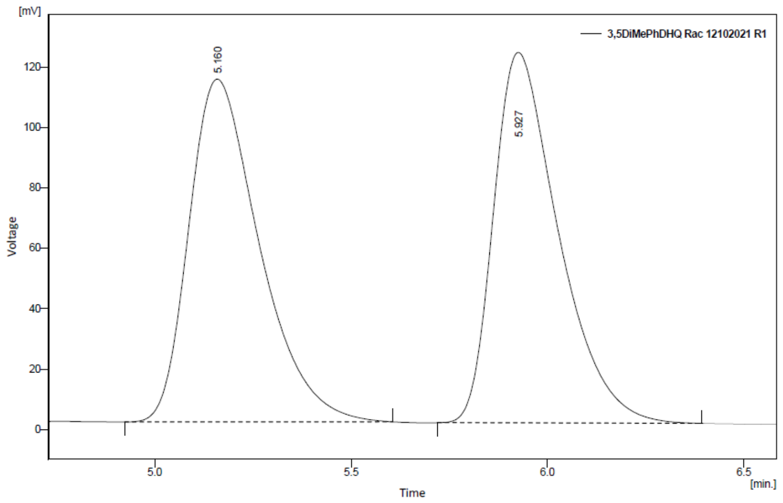

	R_t/ min	Area/ mV.s	Area/ %
1	5.160	1418.203	50.1
2	5.927	1410.977	49.9
	Total	2829.180	100.0

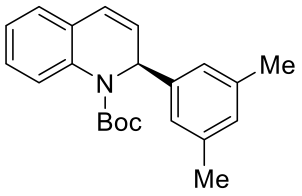

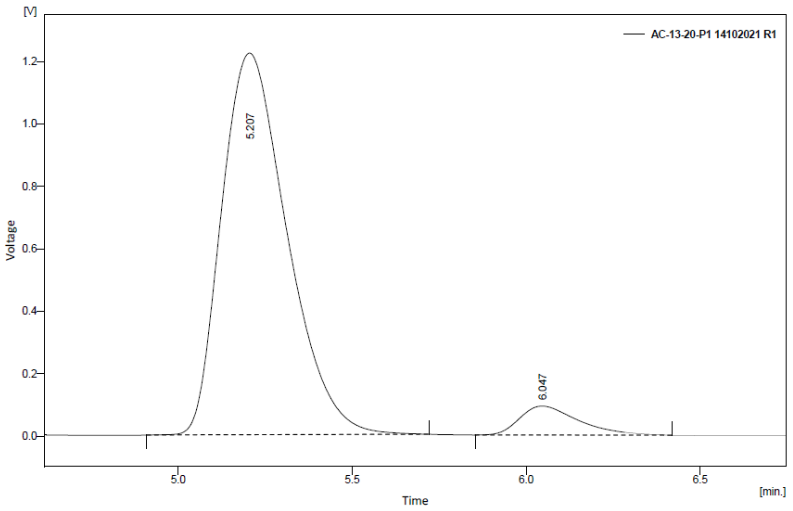

	R_t/ min	Area/ mV.s	Area/ %
1	5.207	16002.121	93.6
2	6.047	1092.730	6.4
	Total	17094.850	100.0

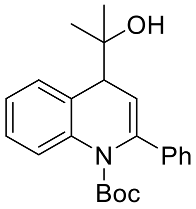

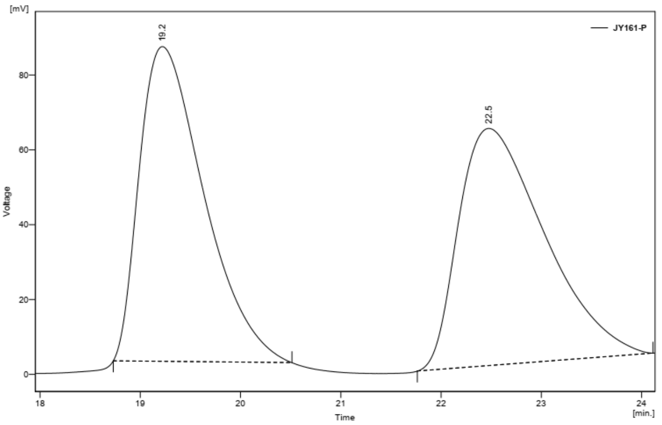

	R_t/ min	Area/ mV.s	Area/ %
1	19.220	7608.833	50
2	22.477	7600.755	50
	total	15209.588	100

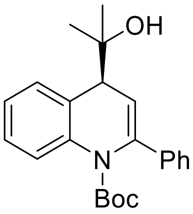

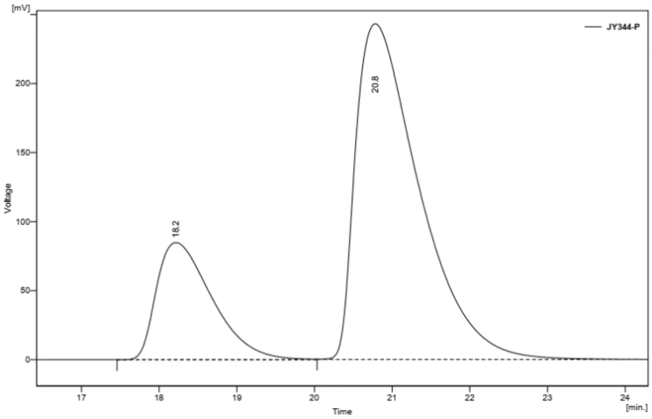

	R_t/ min	Area/ mV.s	Area/ %
1	18.217	4199.737	23
2	20.783	13890.980	77
	total	18090.717	100

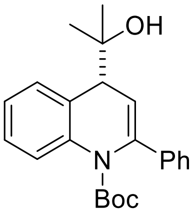

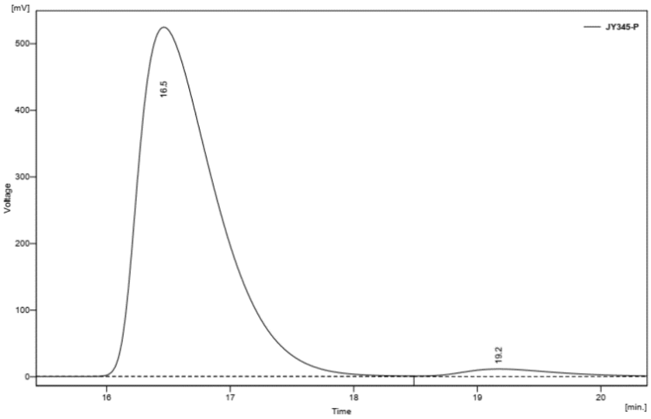

	R_t/ min	Area/ mV.s	Area/ %
1	16.463	22643.422	97
2	19.173	606.260	3
	total	23249.682	100

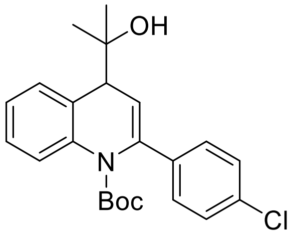

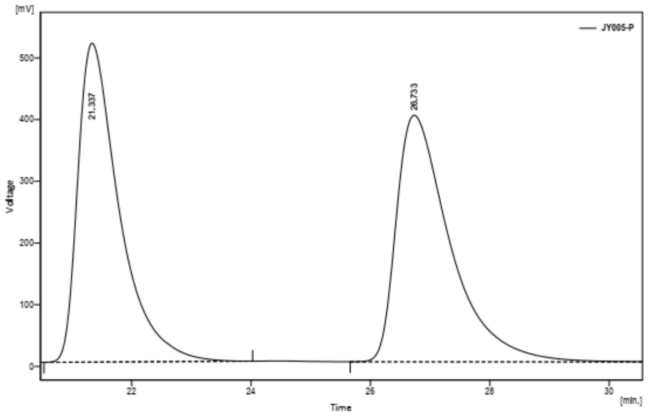

	R_t/ min	Area/ mV.s	Area/ %
1	21.337	24791.257	50
2	26.733	25023.517	50
	total	49814.775	100

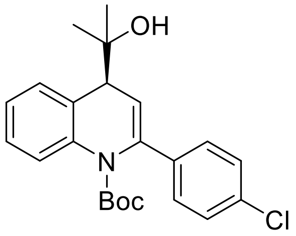

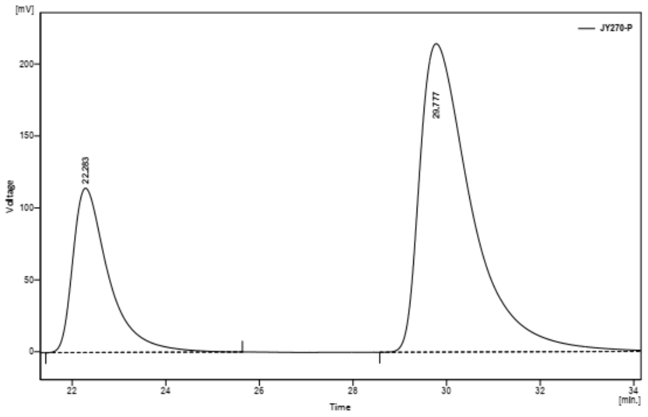

	R_t/ min	Area/ mV.s	Area/ %
1	22.283	6076.582	27
2	29.777	16516.750	73
	total	22593.332	100

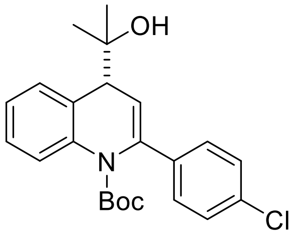

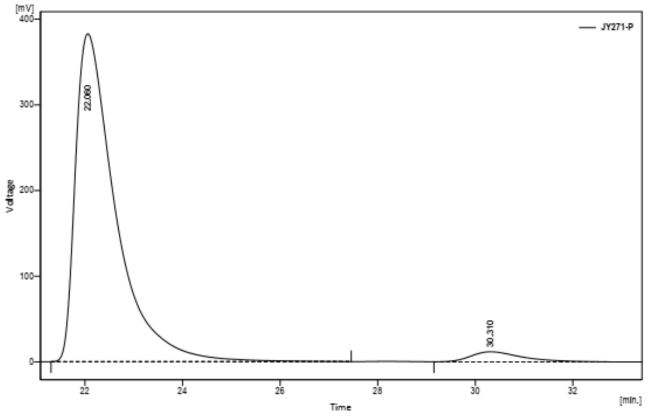

	R_t/ min	Area/ mV.s	Area/ %
1	22.060	21827.652	96
2	30.310	863.305	4
	total	22690.957	100

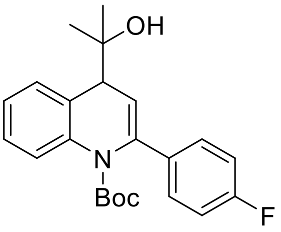

	R_t/ min	Area/ mV.s	Area/ %
1	18.000	5575.894	50
2	21.187	5569.202	50
	total	11145.096	100

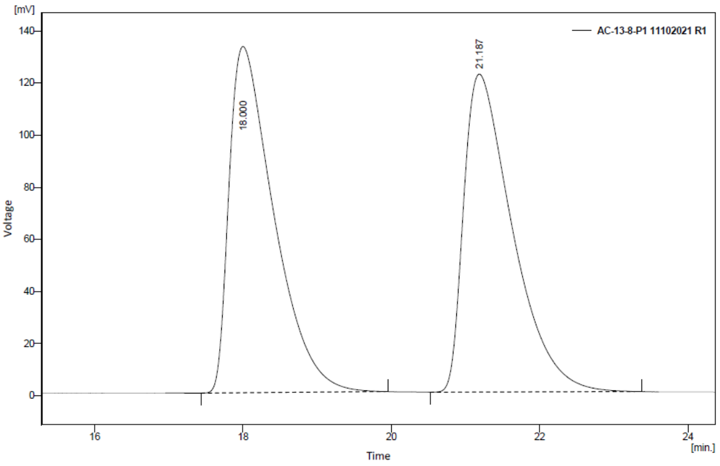

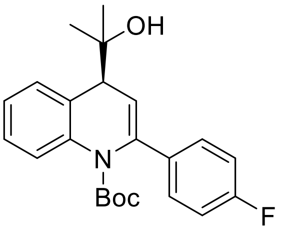

	R_t/ min	Area/ mV.s	Area/ %
1	17.937	21802.324	22.6
2	20.853	74561.345	77.4
	Total	96363.670	100.0

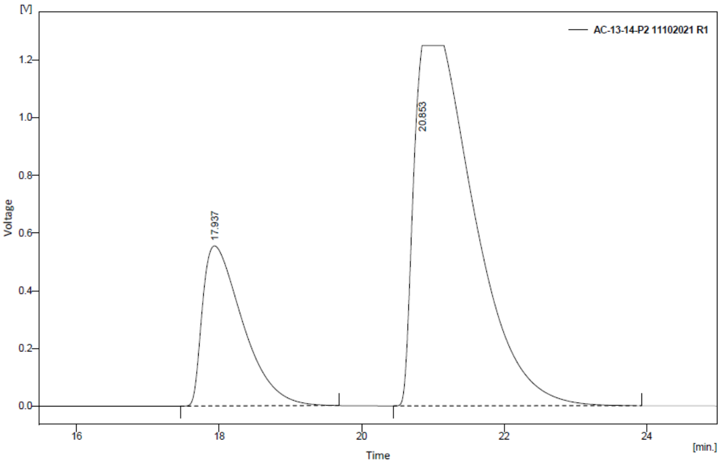

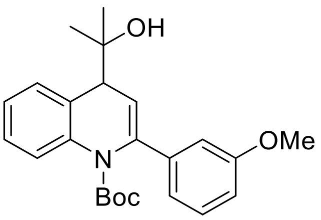

	R_t/ min	Area/ mV.s	Area/ %
1	26.763	10351.339	50
2	29.140	10345.496	50
	total	20696.835	100

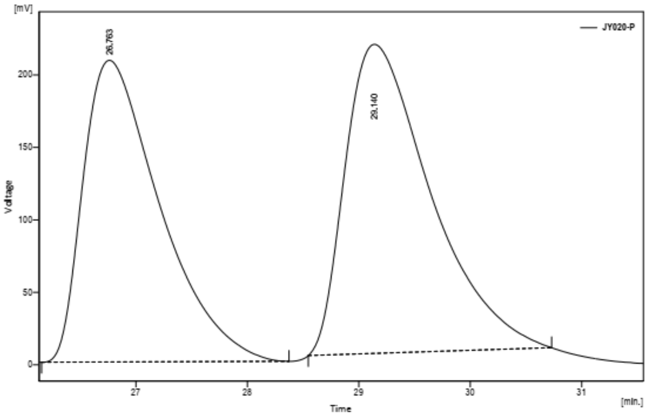

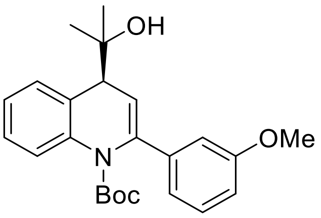

	R_t/ min	Area/ mV.s	Area/ %
1	28.420	18917.263	77
2	32.140	5777.444	23
	total	24694.708	100

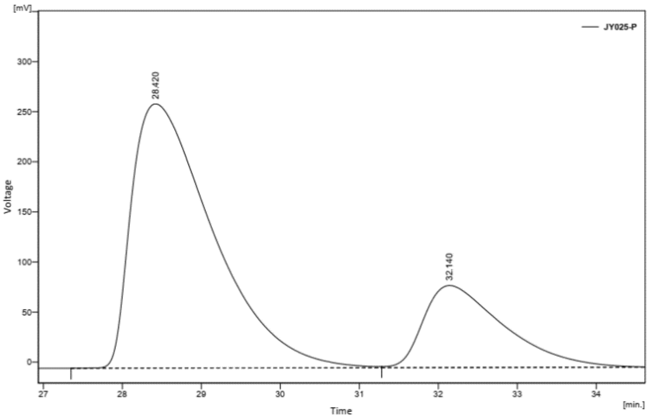

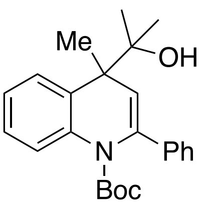

	Rt/ min	Area/ mV.s	Area/ %
1	11.300	14725.208	50
2	16.233	14668.849	50
	total	29394.057	100

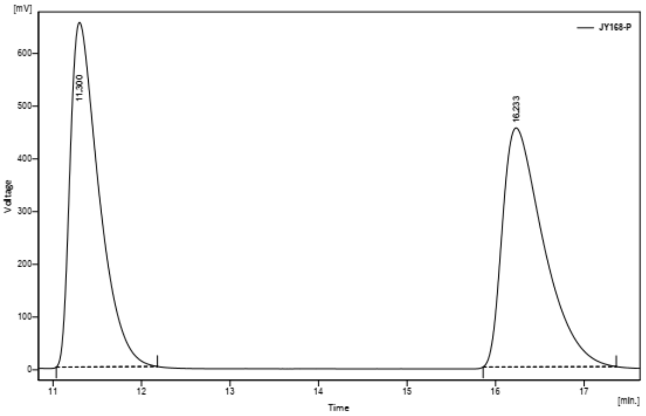

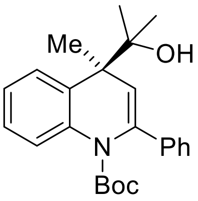

	Rt/ min	Area/ mV.s	Area/ %
1	11.243	4894.442	18
2	14.223	22084.322	82
	total	26978.763	100

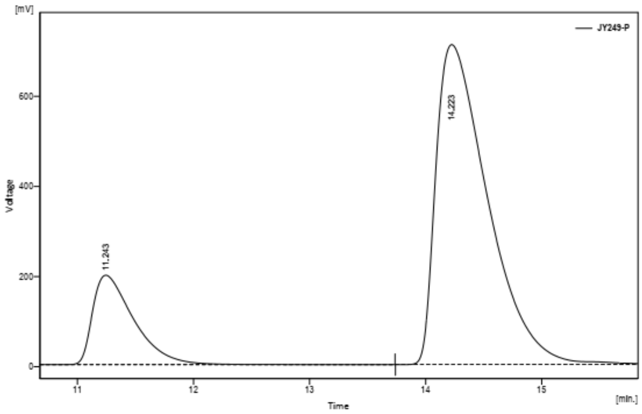

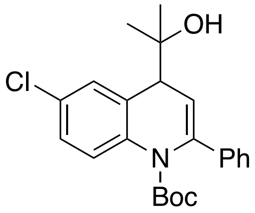

	R_t/ min	Area/ mV.s	Area/ %
1	19.303	8068.067	50.1
2	22.177	8038.618	49.9
	Total	16106.685	100.0

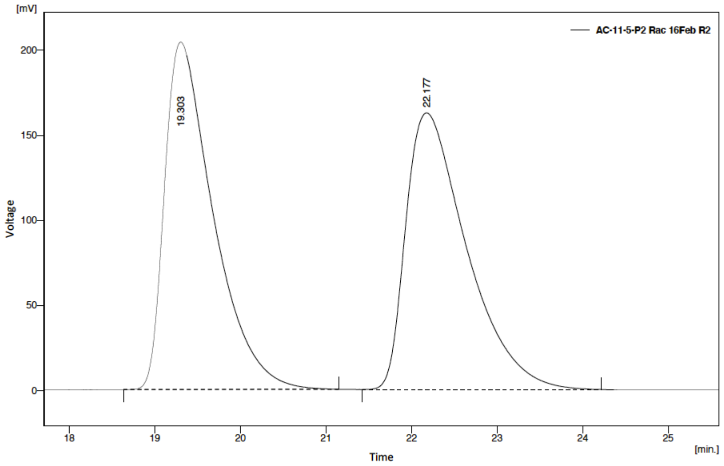

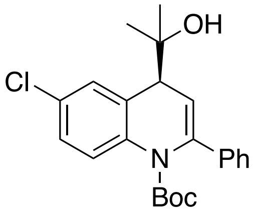

	R_t/ min	Area/ mV.s	Area/ %
1	19.333	4460.176	18.0
2	22.190	20289.304	82.0
	Total	24749.479	100.0

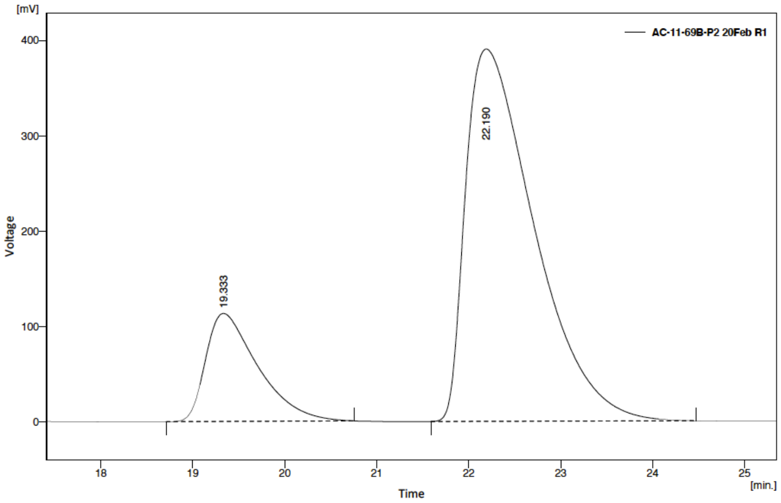

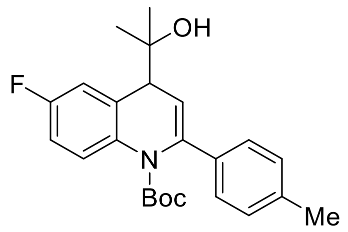

	R_t/ min	Area/ mV.s	Area/ %
1	20.430	13876.392	50
2	23.157	13866.089	50
	total	27742.481	100

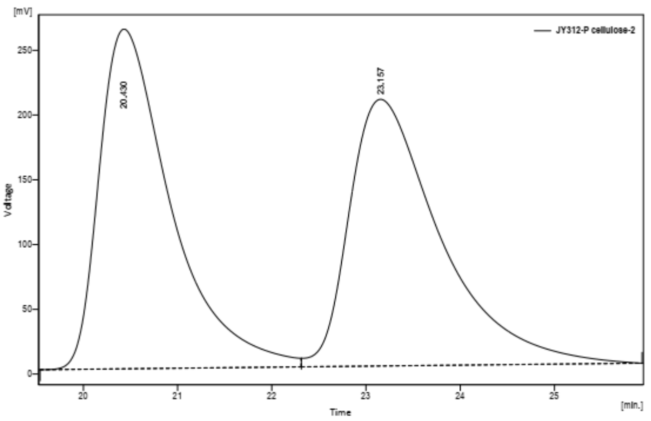

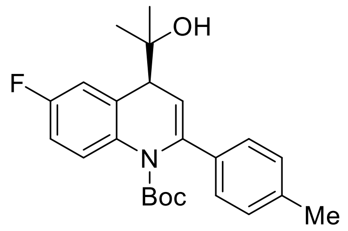

	R_t/ min	Area/ mV.s	Area/ %
1	21.320	40323.235	75
2	24.553	13777.611	25
	total	54100.846	100

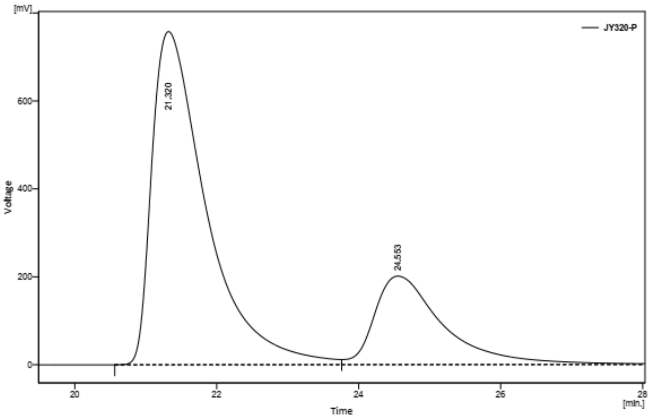

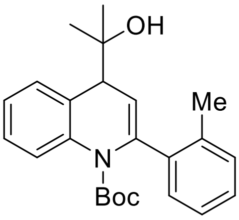

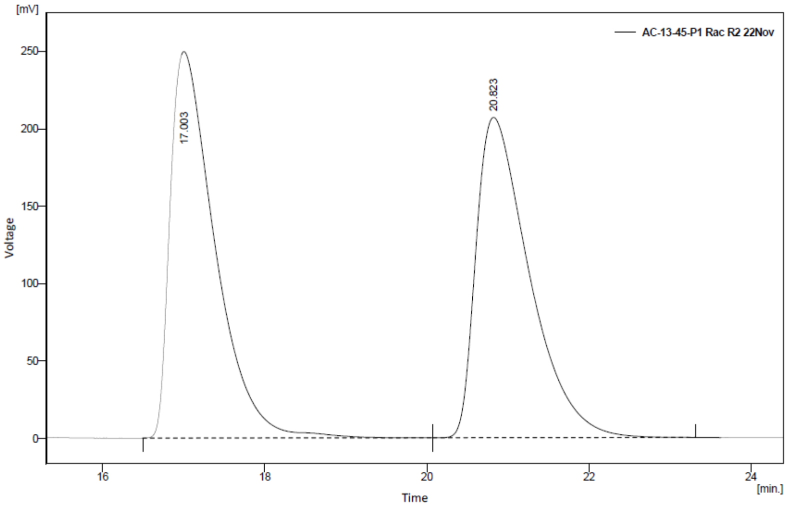

	R_t/ min	Area/ mV.s	Area/ %
1	17.003	9558.448	50.3
2	20.823	9442.581	49.7
	Total	19001.029	100.0

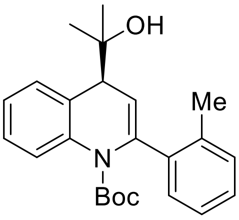

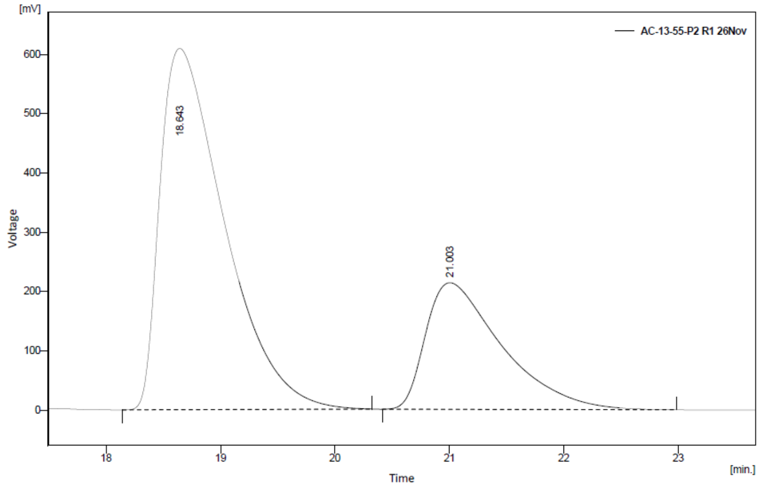

	R_t/ min	Area/ mV.s	Area/ %
1	18.643	23856.189	70.7
2	21.003	9886.732	29.3
	Total	33742.921	100.0

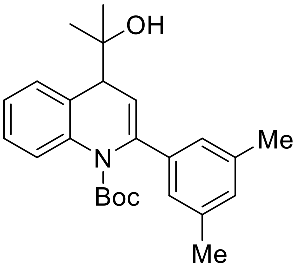

	R_t/ min	Area/ mV.s	Area/ %
1	19.723	3558.509	50.1
2	25.337	3541.361	49.9
	Total	7099.870	100.0

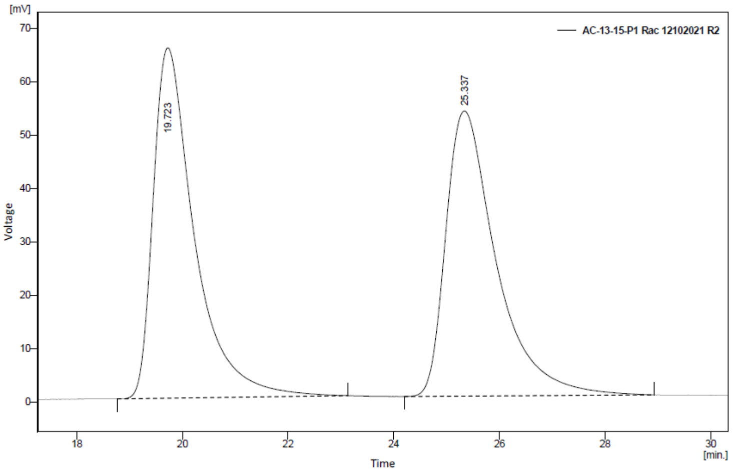

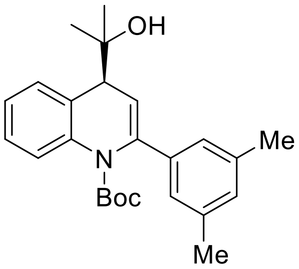

	R_t/ min	Area/ mV.s	Area/ %
1	19.720	3894.527	20.4
2	24.963	15179.321	79.6
	Total	19073.848	100.0

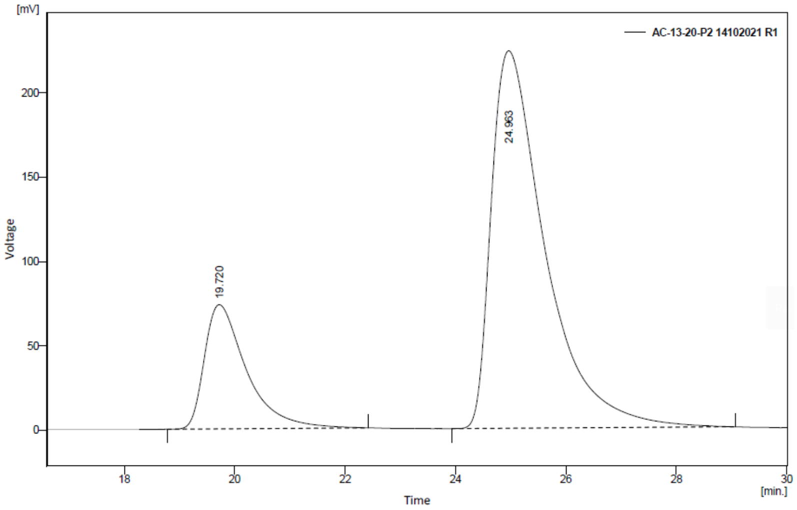

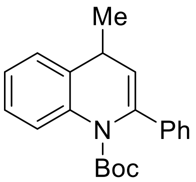

	R_t/ min	Area/ mV.s	Area/ %
1	4.813	701.793	50
2	6.147	701.934	50
	total	1403.727	100

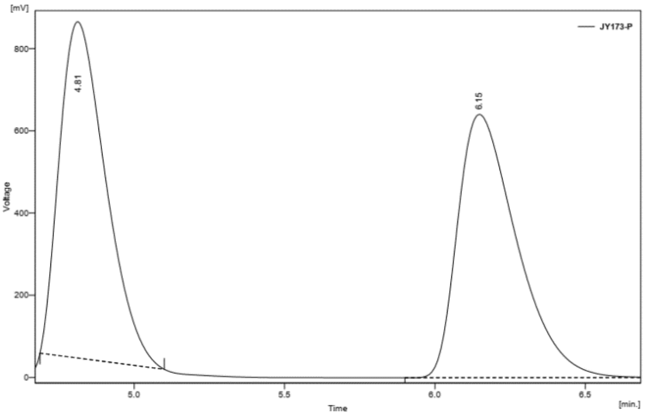

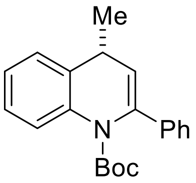

	R_t/ min	Area/ mV.s	Area/ %
1	4.800	213.242	2
2	6.050	11649.334	98
	total	11862.576	100

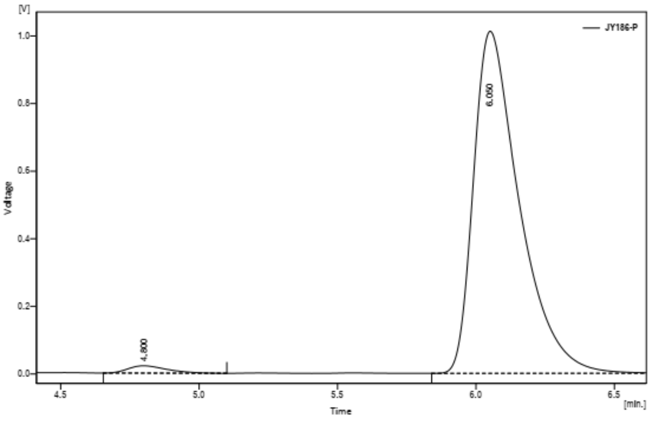

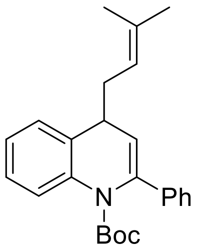

	R_t/ min	Area/ mV.s	Area/ %
1	3.887	2131.417	50
2	4.170	2127.404	50
	total	4258.820	100

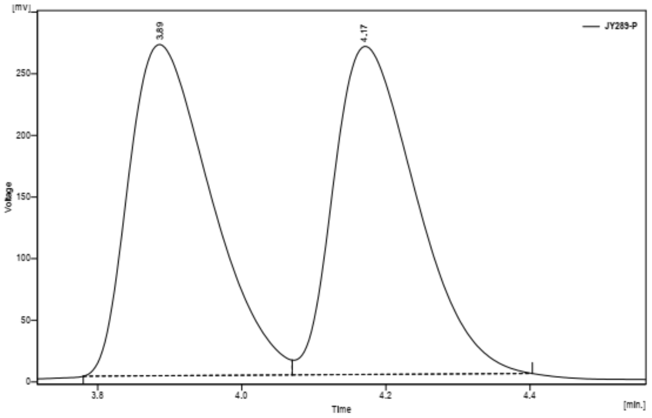

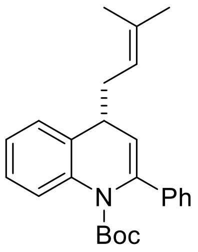

Prior to recrystallization:

	R_t/ min	Area/ mV.s	Area/ %
1	3.953	1019.834	9
2	4.220	10311.658	91
	total	11331.492	100

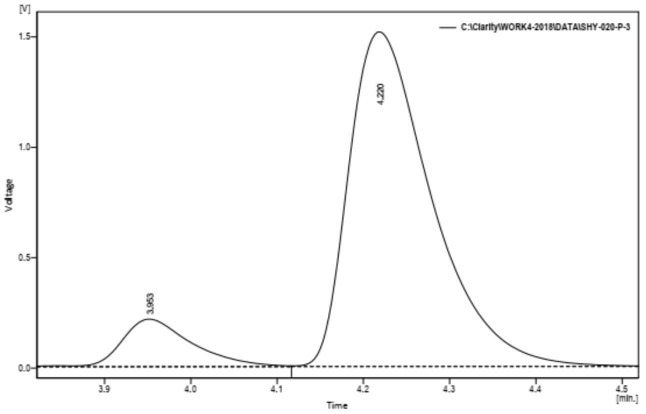

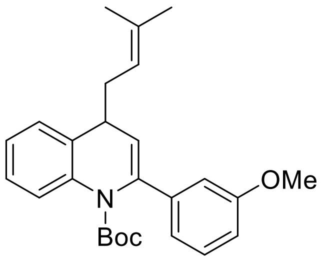

	R_t/ min	Area/ mV.s	Area/ %
1	4.523	3361.657	50
2	5.097	3363.636	50
	total	6725.293	100

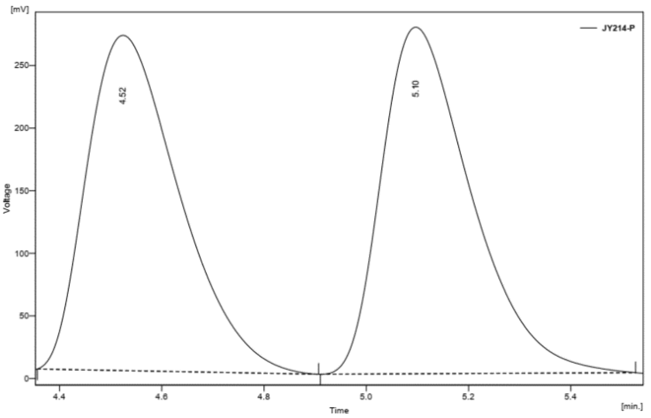

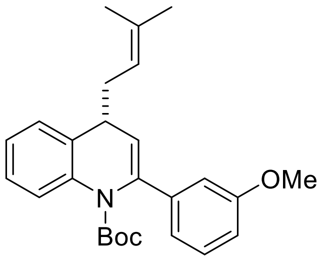

	R_t/ min	Area/ mV.s	Area/ %
1	5.060	1319.635	7
2	5.357	16461.287	93
	total	17780.922	100

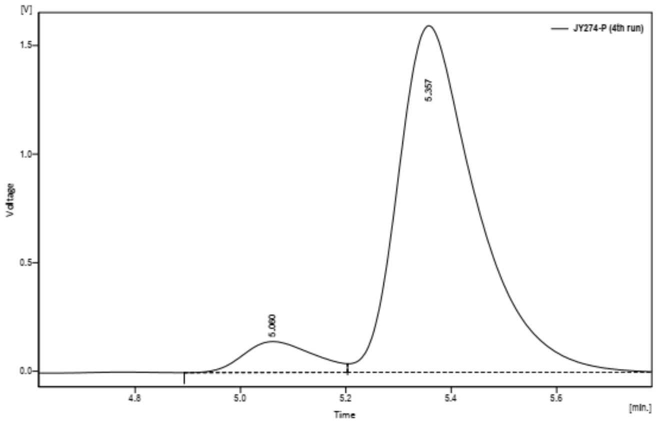

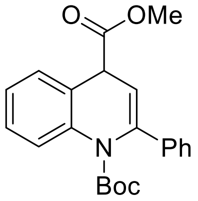

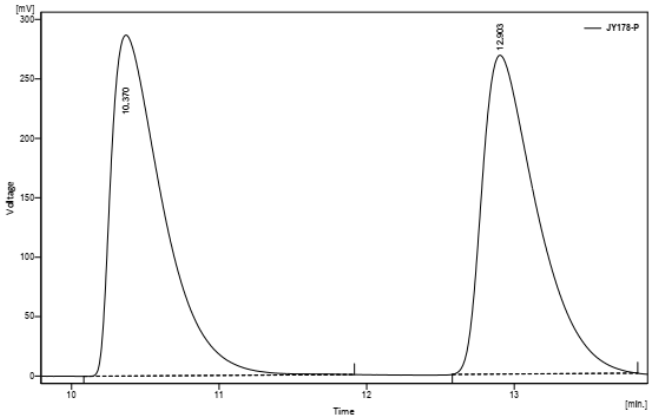

	R_t/ min	Area/ mV.s	Area/ %
1	10.370	6930.881	50
2	12.903	6933.940	50
	total	13864.821	100

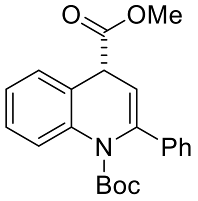

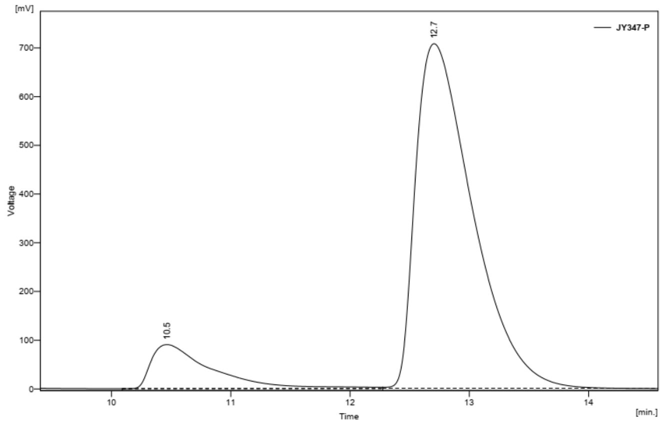

	R_t/ min	Area/ mV.s	Area/ %
1	10.463	3192.163	12
2	12.703	23509.716	88
	total	26701.878	100

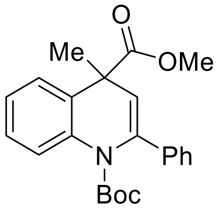

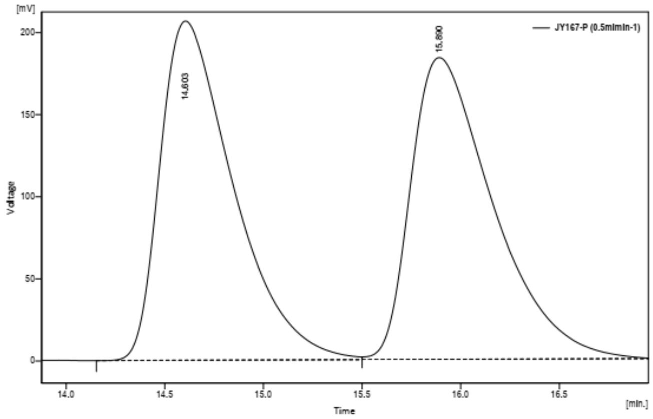

	R_t/ min	Area/ mV.s	Area/ %
1	14.603	5331.608	50
2	15.890	5323.549	50
	total	10655.157	100

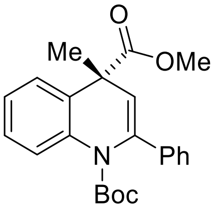

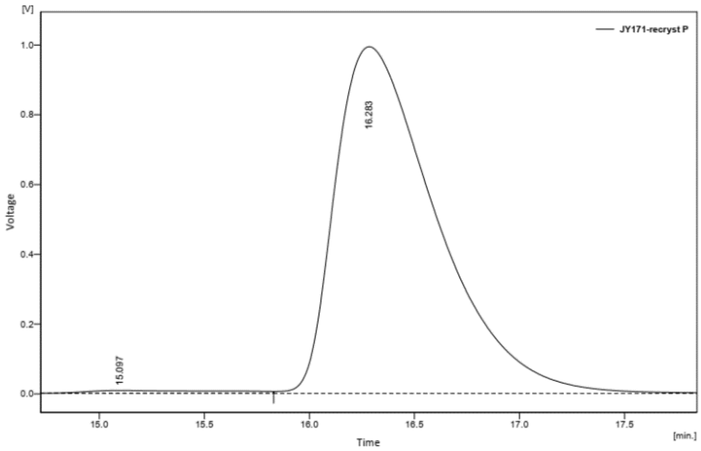

	R_t/ min	Area/ mV.s	Area/ %
1	15.097	382.368	1
2	16.283	33105.083	99
	total	33487.450	100

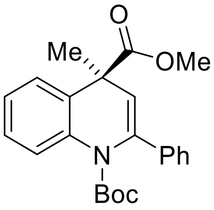

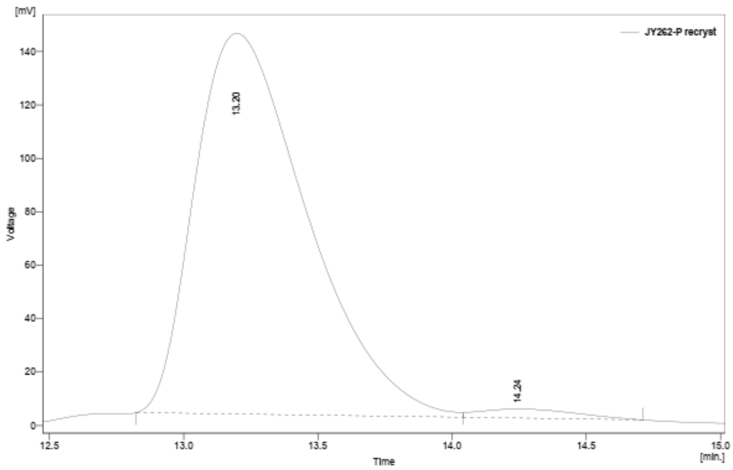

	R_t/ min	Area/ mV.s	Area/ %
1	13.197	4169.016	98
2	14.243	85.062	2
	total	4254.078	100

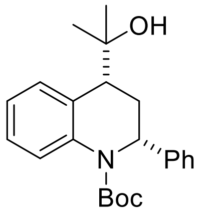

	R_t/ min	Area/ mV.s	Area/ %
1	27.553	3579.377	44
2	30.500	4559.316	56
	total	8138.693	100

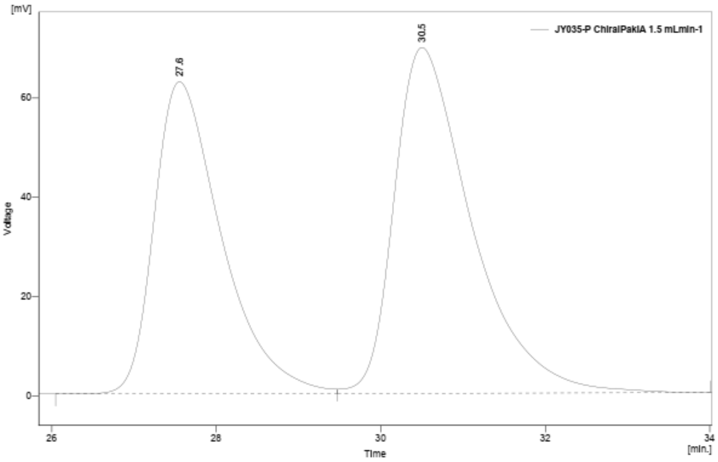

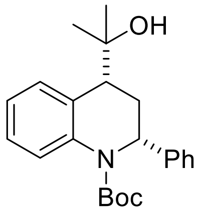

	R_t/ min	Area/ mV.s	Area/ %
1	27.593	181.525	4
2	30.207	4397.610	96
	total	4579.136	100

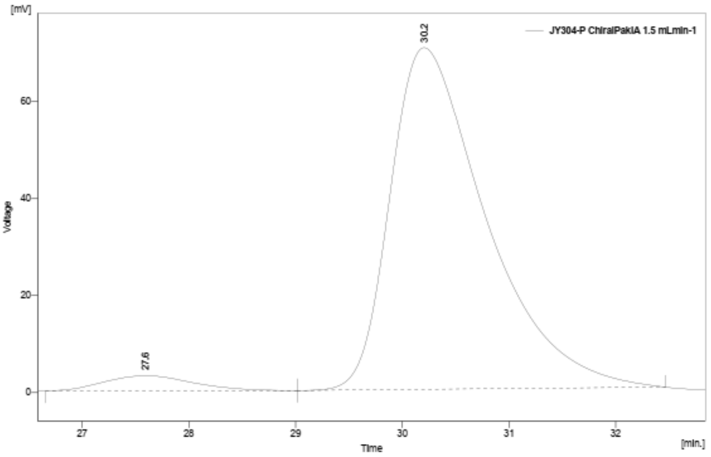

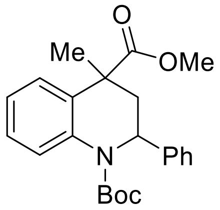

Crude racemic **8**
Using Cellulose-2
column

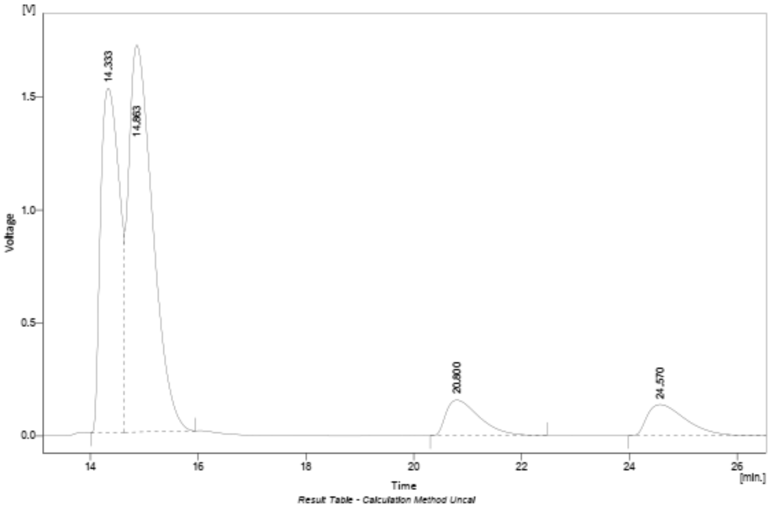

Result Table - Calculation Method Uncal

	Reten. Time [min]	Area [mV.s]	Height [mV]	Area [%]	Height [%]	W05 [min]
1	14.333	37105.621	1525.723	35.3	43.2	0.47
2	14.863	54211.925	1713.317	51.6	48.5	0.55
3	20.800	6808.401	157.770	6.5	4.5	0.67
4	24.570	6891.405	137.920	6.6	3.9	0.78
	Total	105017.353	3534.731	100.0	100.0	

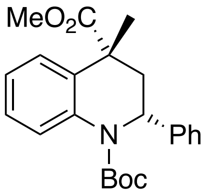

8a

racemic
using ChiralPak IA column

	R_t/ min	Area/ mV.s	Area/ %
1	7.010	961.828	50
2	9.990	962.453	50
	total	1924.281	100

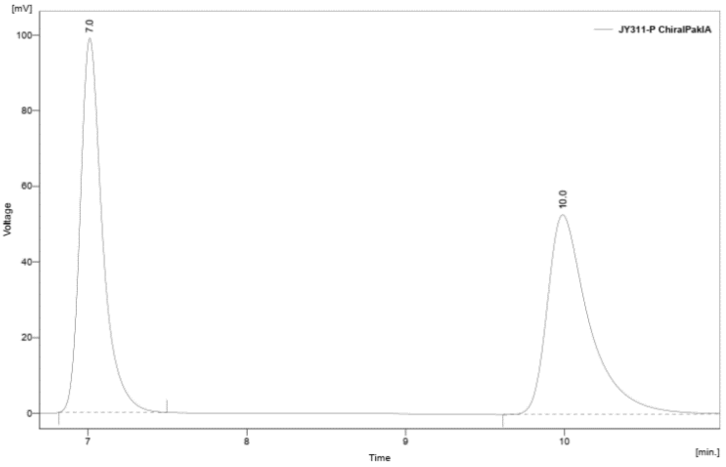

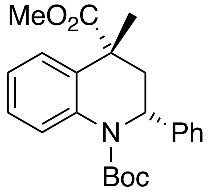

8a

	R_t/ min	Area/ mV.s	Area/ %
1	6.900	6948.013	98
2	9.280	139.718	2
	total	7087.731	100

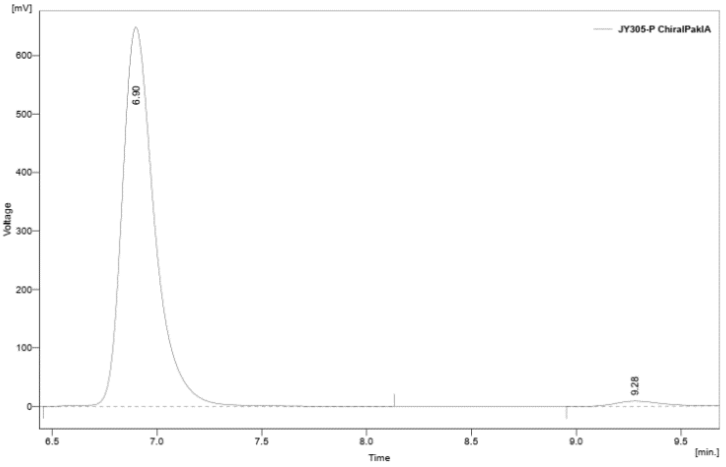

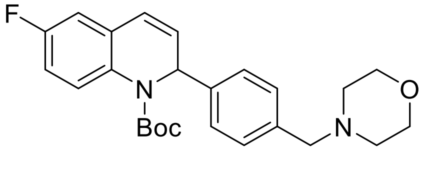

	R_t/ min	Area/ mV.s	Area/ %
1	7.633	8548.505	50
2	8.720	8537.826	50
	total	17086.332	100

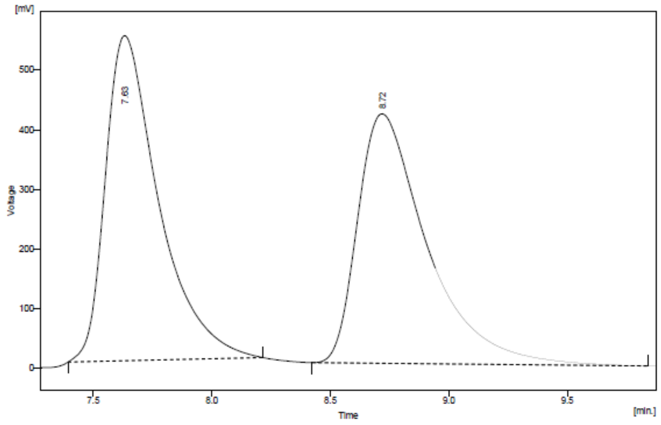

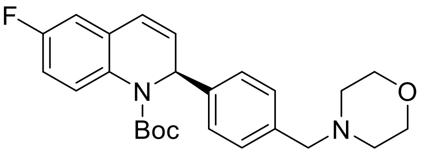

	R_t/ min	Area/ mV.s	Area/ %
1	8.153	1529.148	16
2	9.417	8186.975	84
	total	9716.123	100

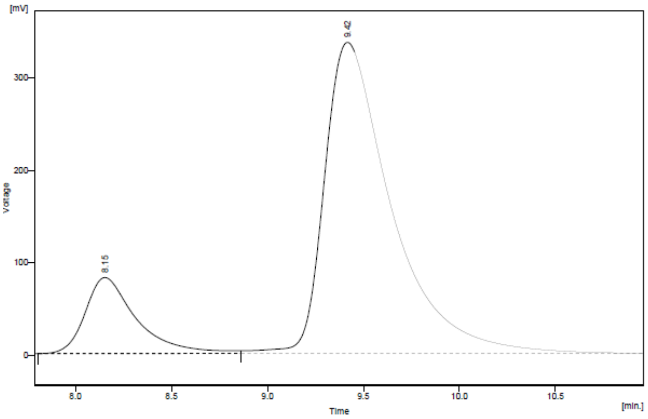

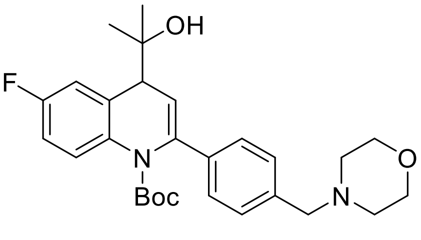

	R_t/ min	Area/ mV.s	Area/ %
1	20.350	6697.244	50
2	23.987	6703.029	50
	total	13400.273	100

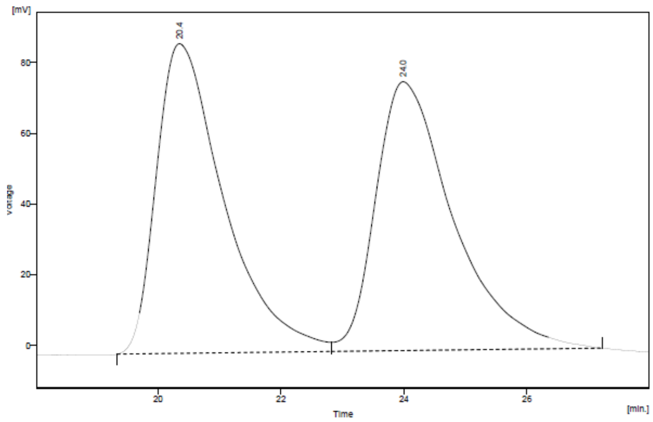

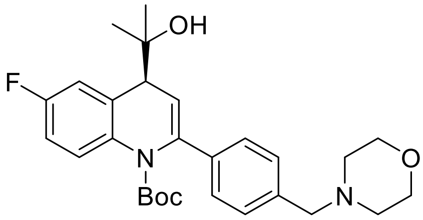

	R_t/ min	Area/ mV.s	Area/ %
1	20.693	3563.870	18
2	23.683	15948.043	82
	total	19511.914	100

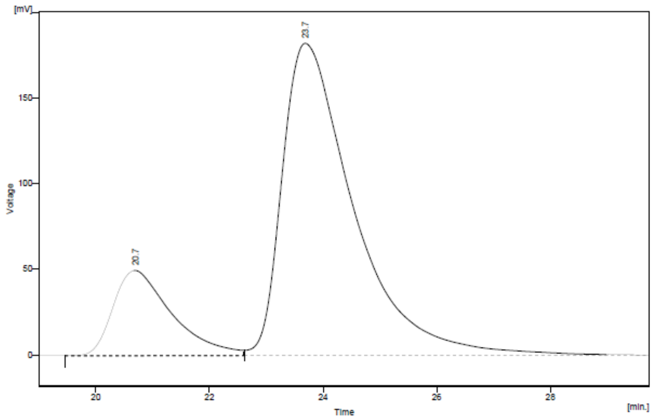

7. NMR spectra (room temperature unless stated)

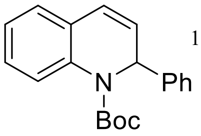

^{1}H NMR spectrum ($CDCl_3$, 400 MHz):

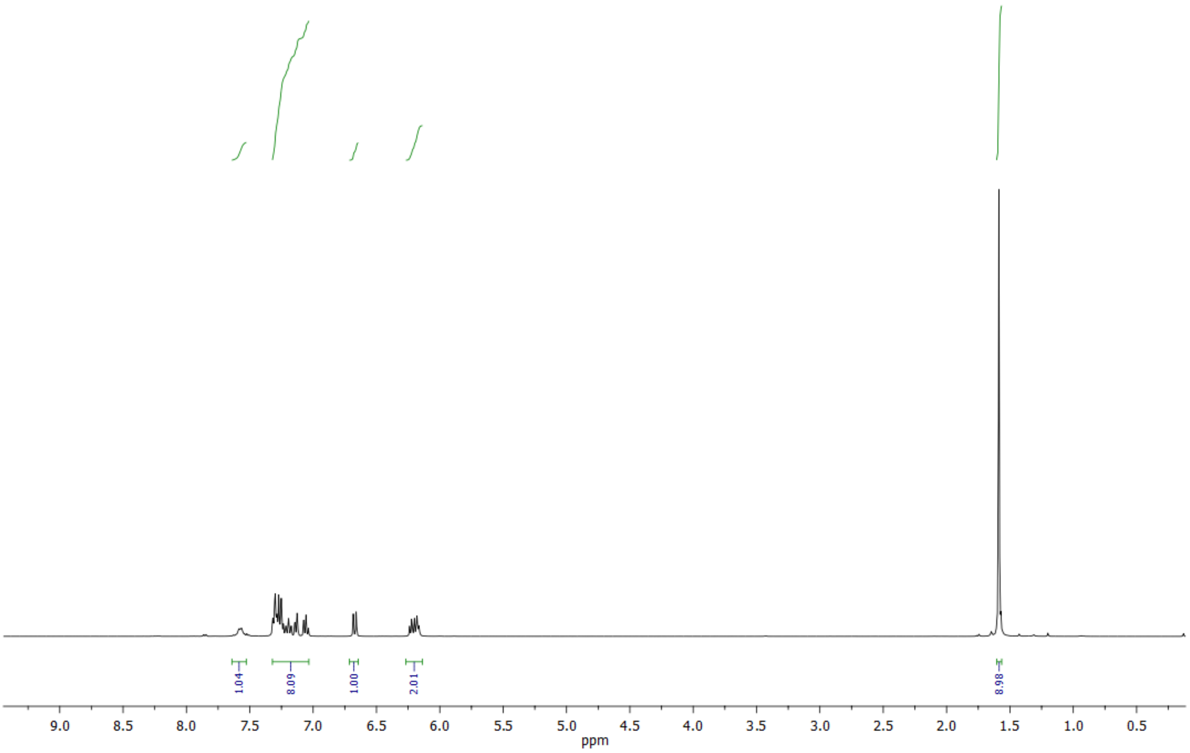

^{13}C{1H} NMR spectrum ($CDCl_3$, 100 MHz):

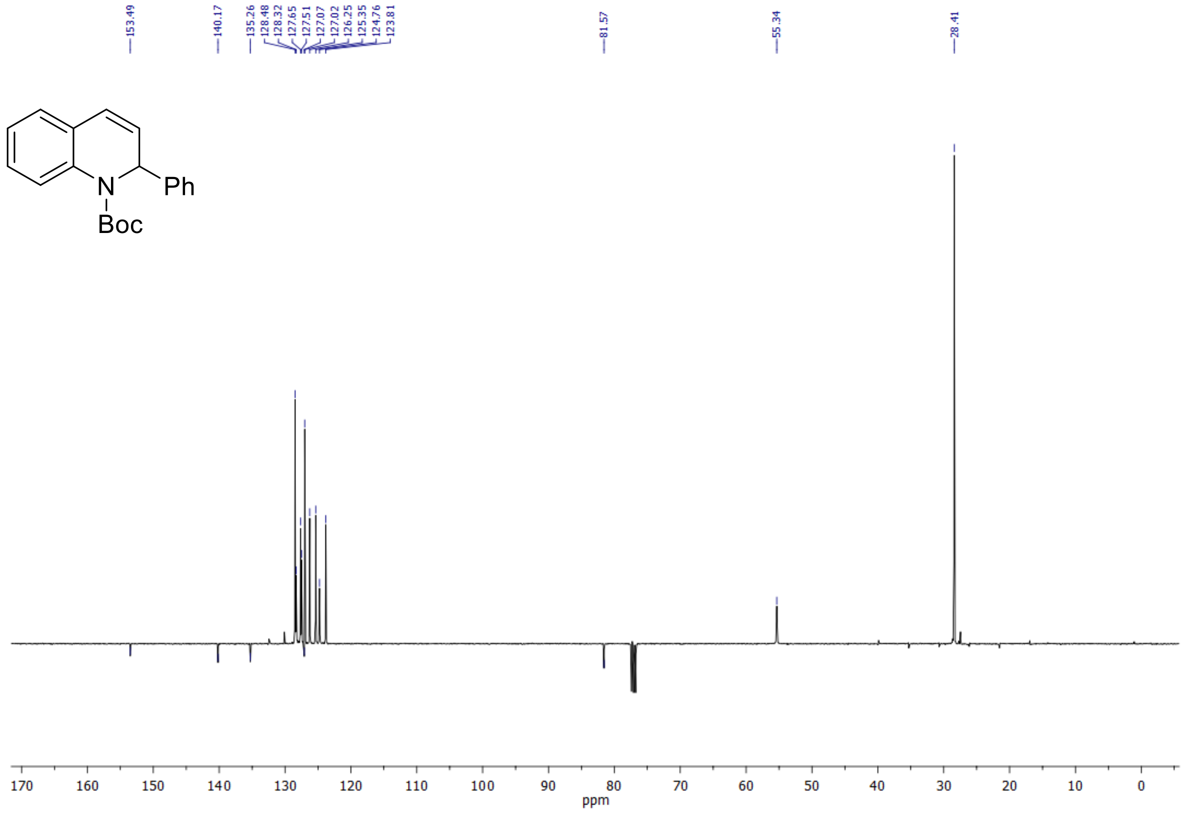

^{1}H NMR spectrum (CDCl$_3$, 400 MHz):

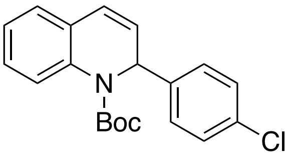

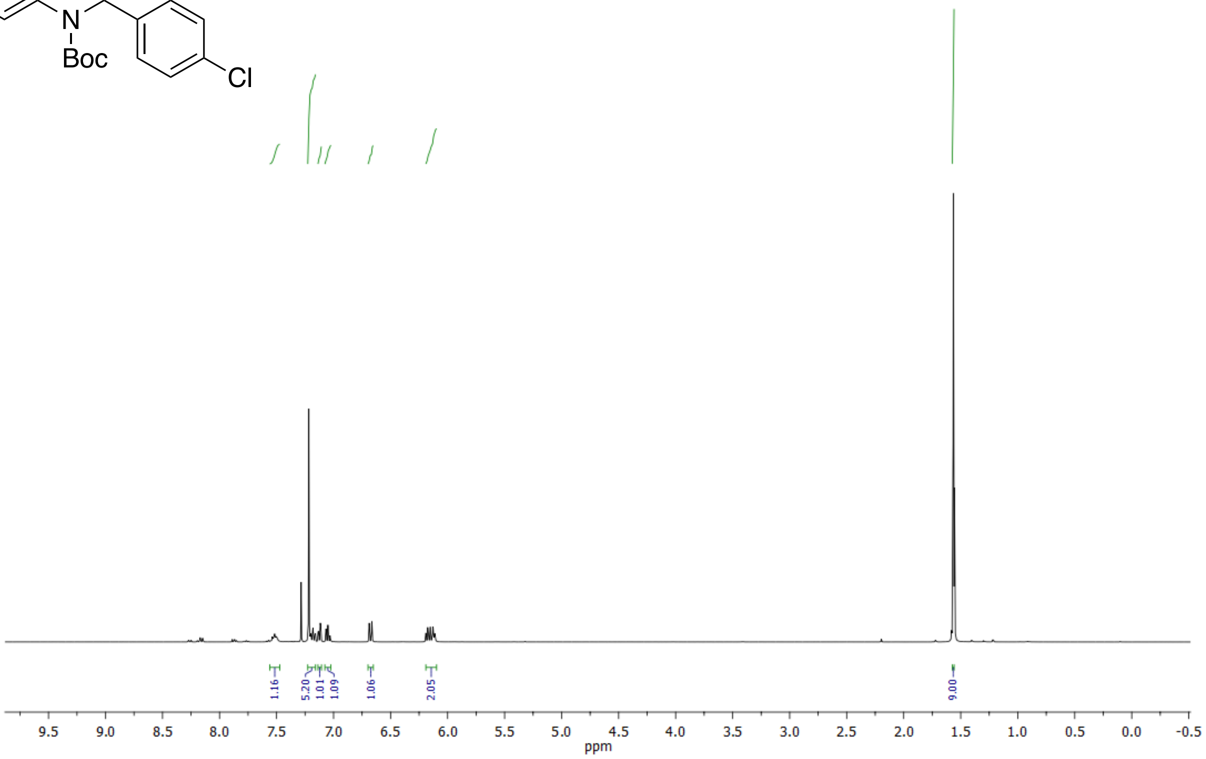

^{13}C{1H} NMR spectrum (CDCl$_3$, 100 MHz):

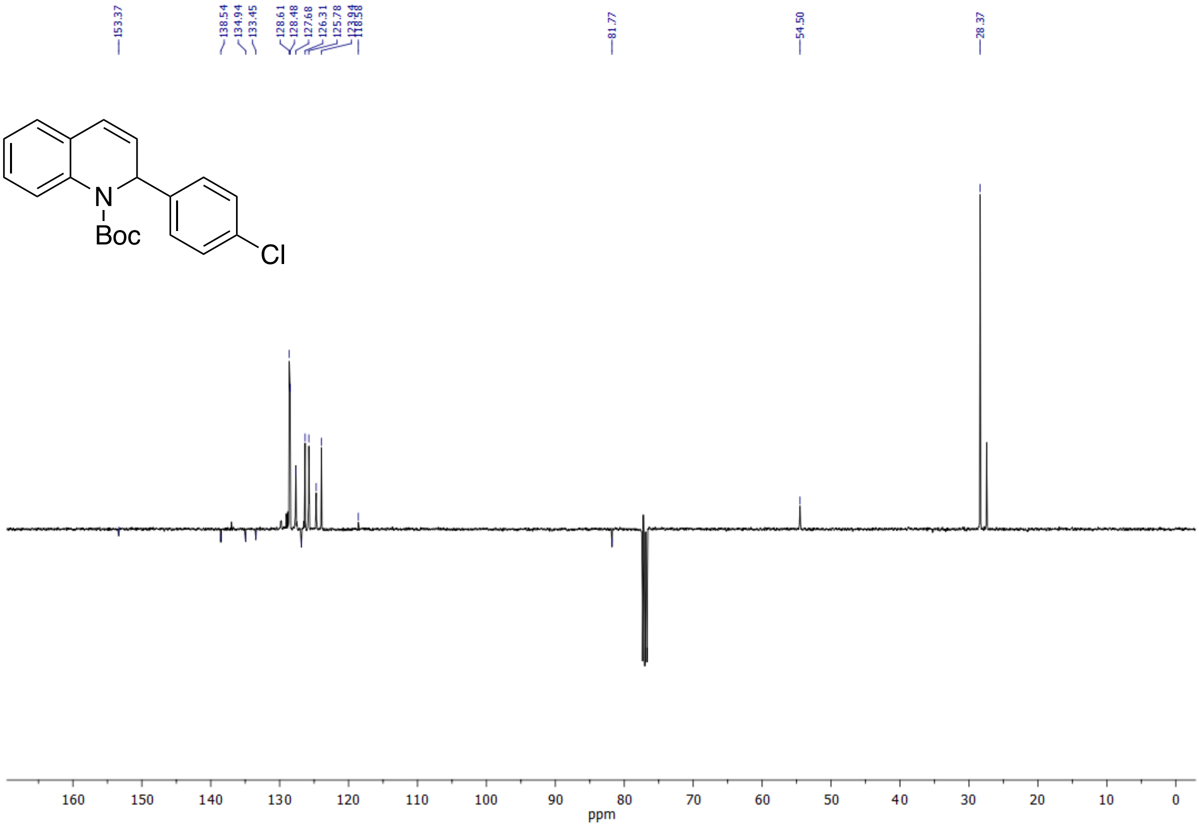

^{1}H NMR spectrum ($CDCl_3$, 400 MHz):

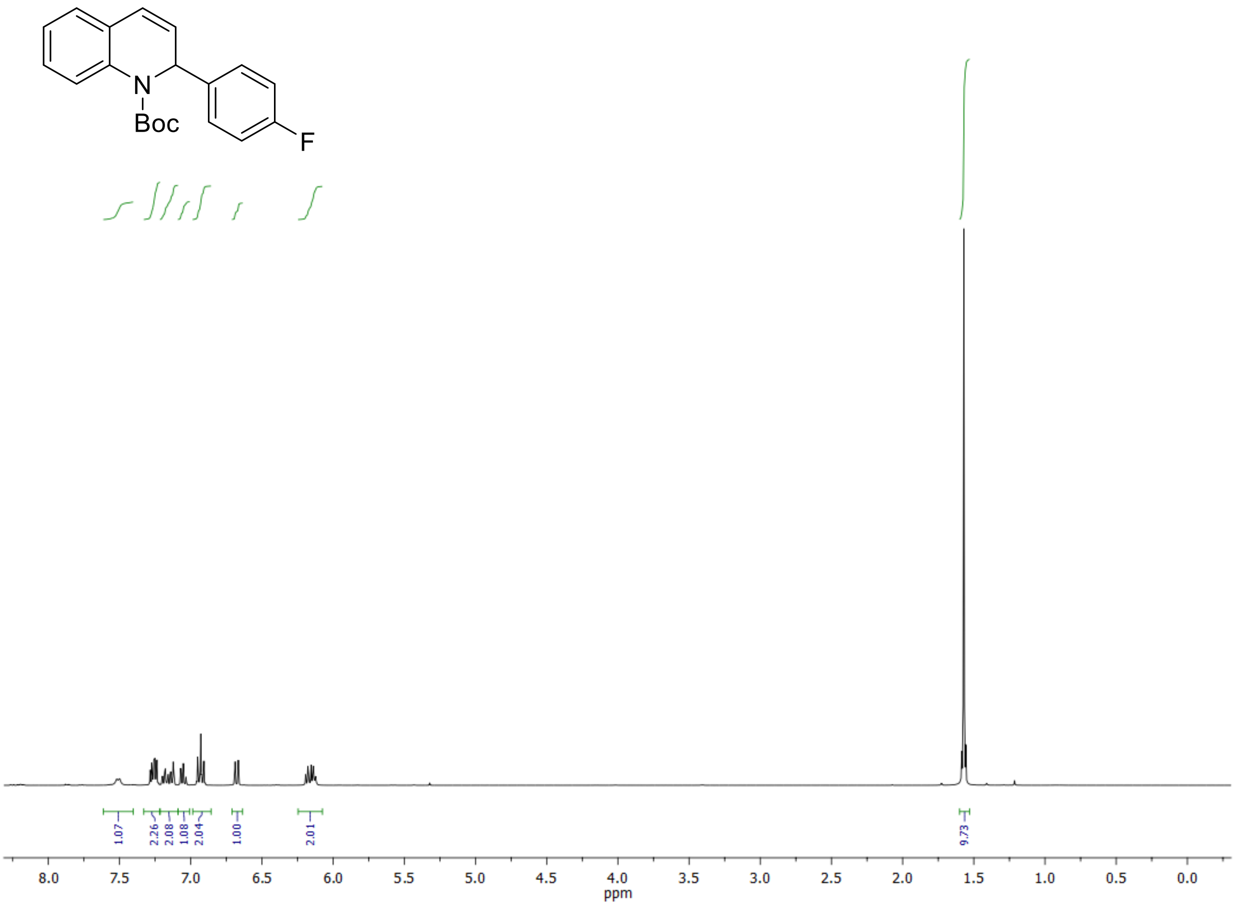

$^{13}C\{1H\}$ NMR spectrum ($CDCl_3$, 100 MHz):

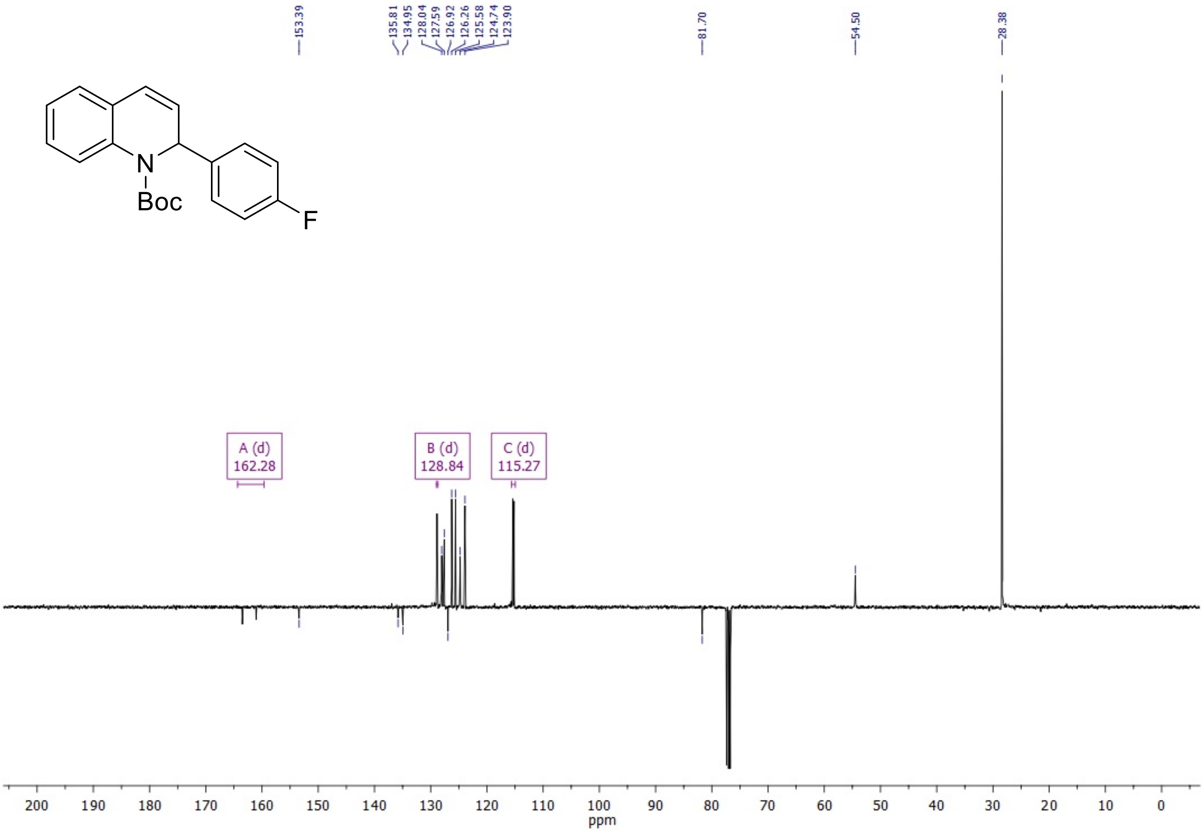

^{19}F NMR spectrum ($CDCl_3$, 377 MHz):

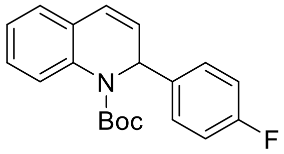

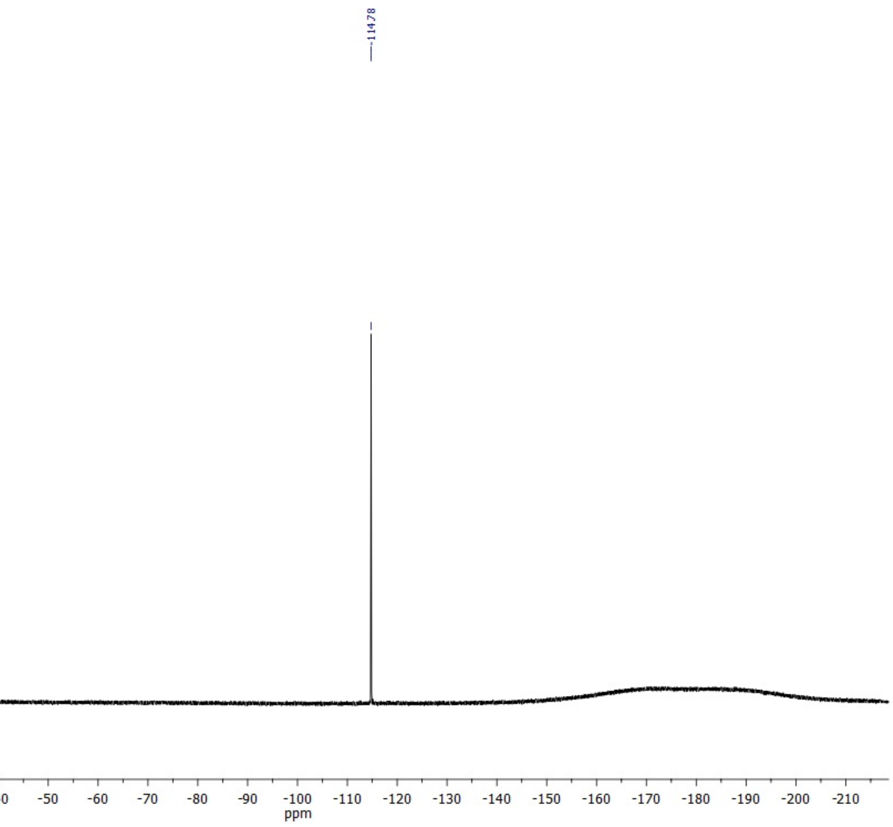

^{1}H NMR spectrum ($CDCl_3$, 400 MHz):

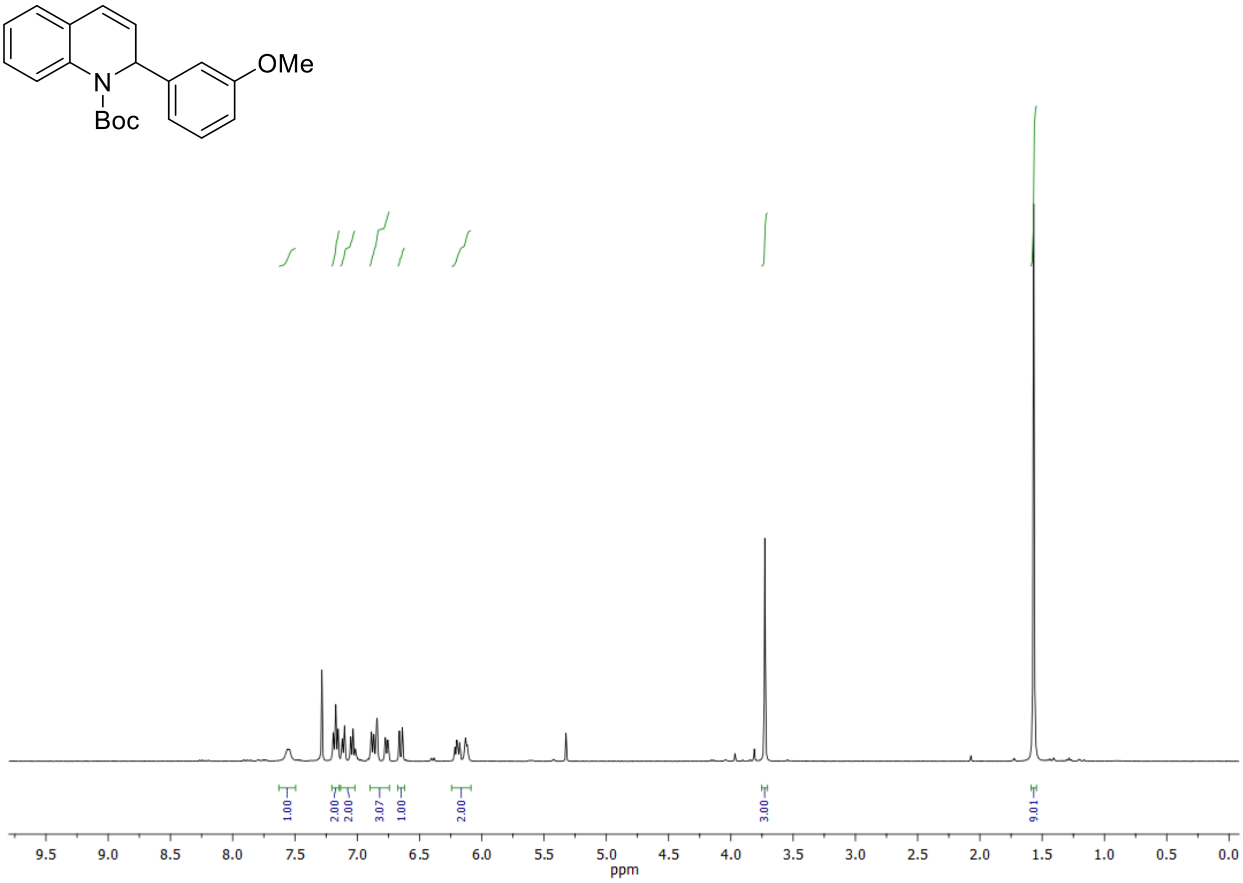

$^{13}C\{1H\}$ NMR spectrum ($CDCl_3$, 100 MHz):

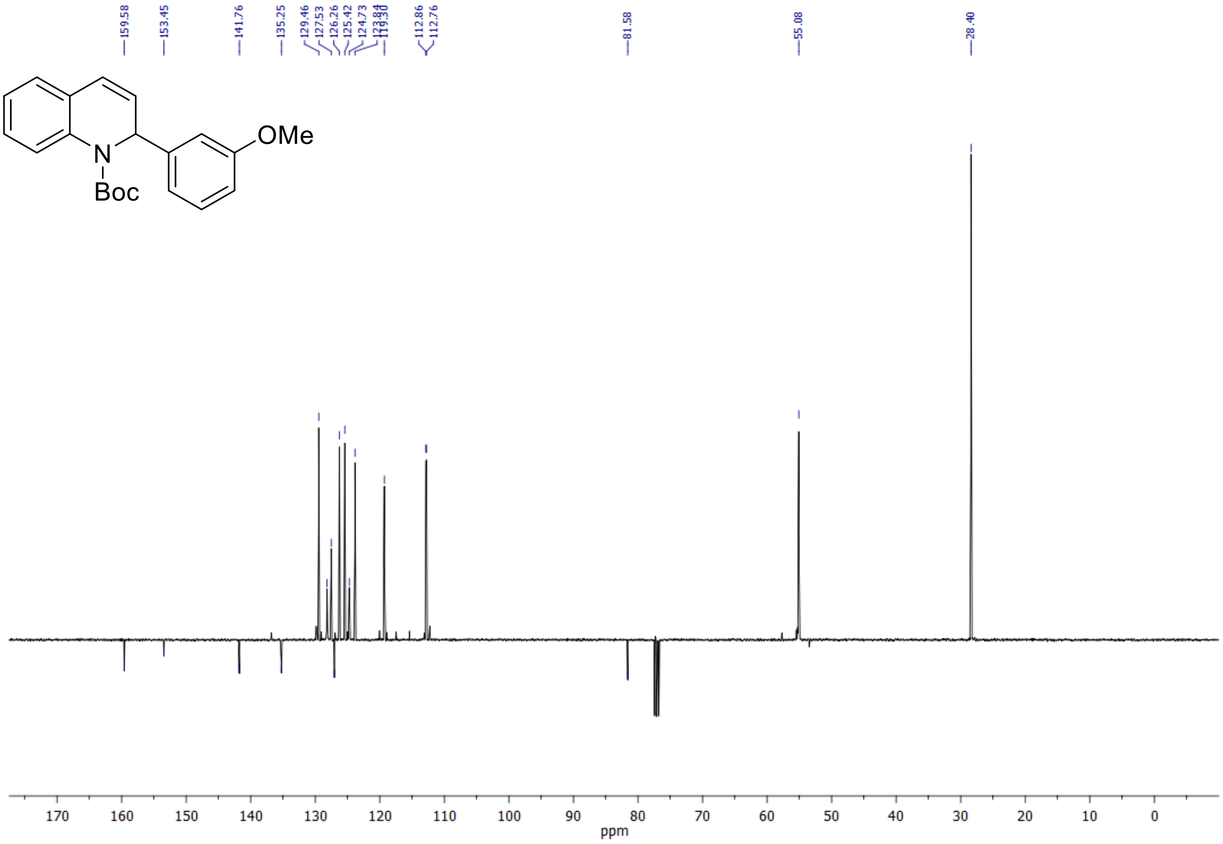

^{1}H NMR spectrum ($CDCl_3$, 400 MHz):

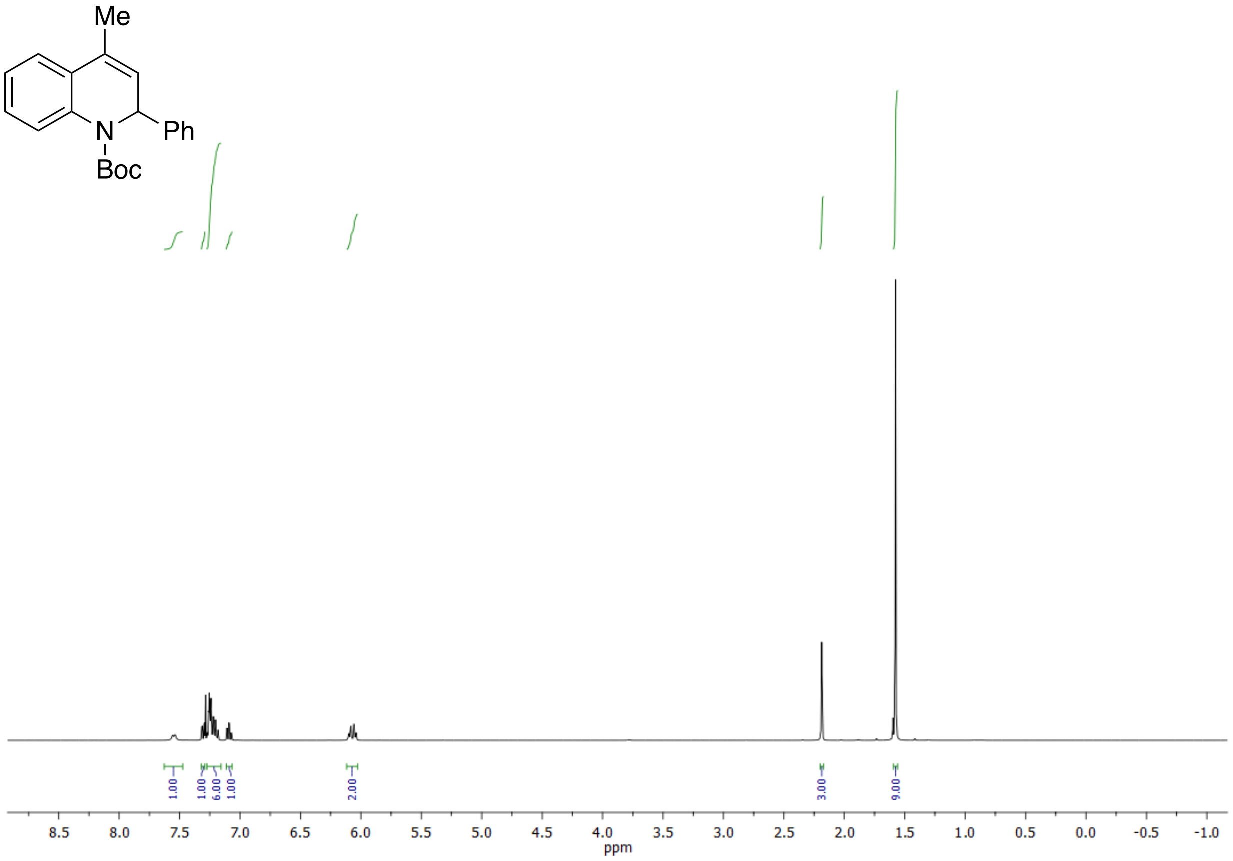

^{13}C{1H} NMR spectrum ($CDCl_3$, 100 MHz):

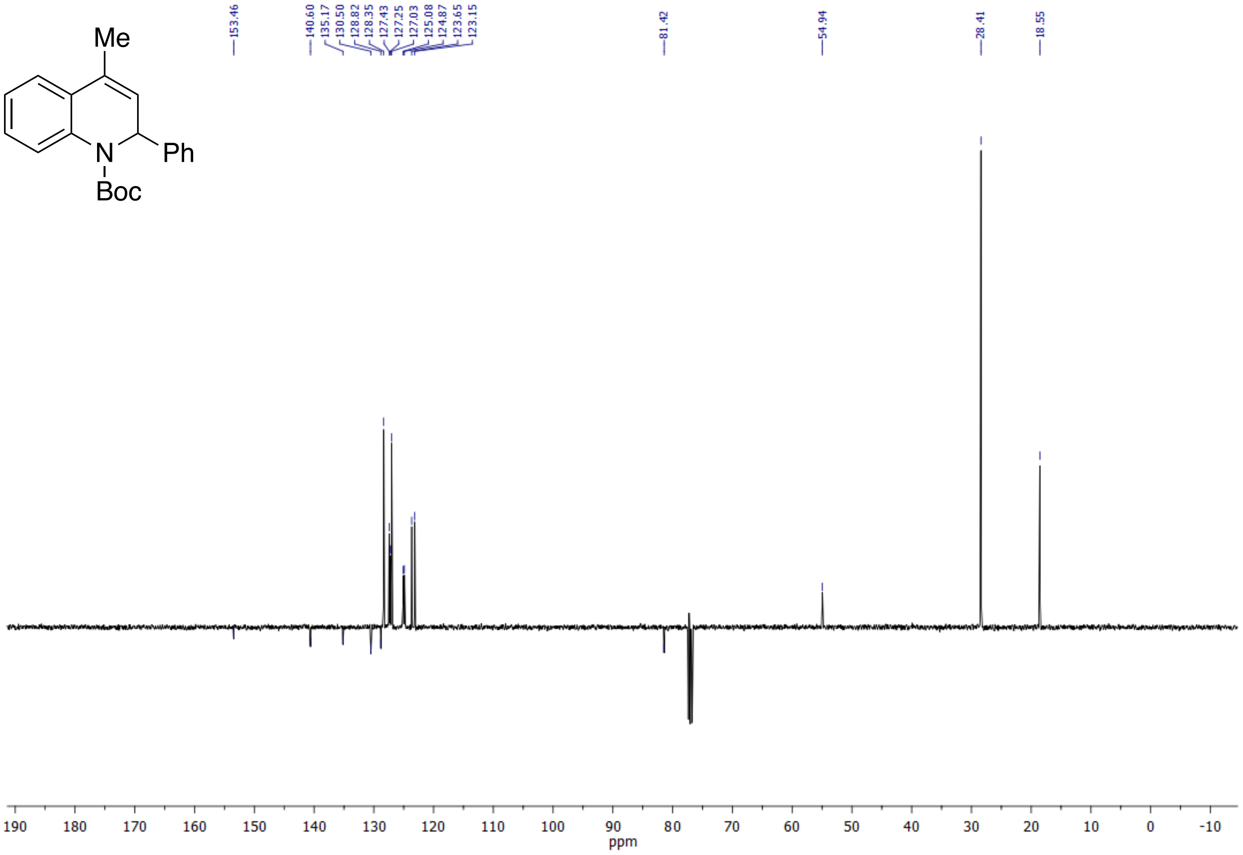

^{1}H NMR spectrum ($CDCl_3$, 400 MHz):

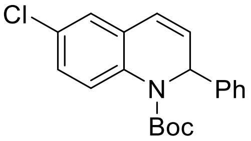

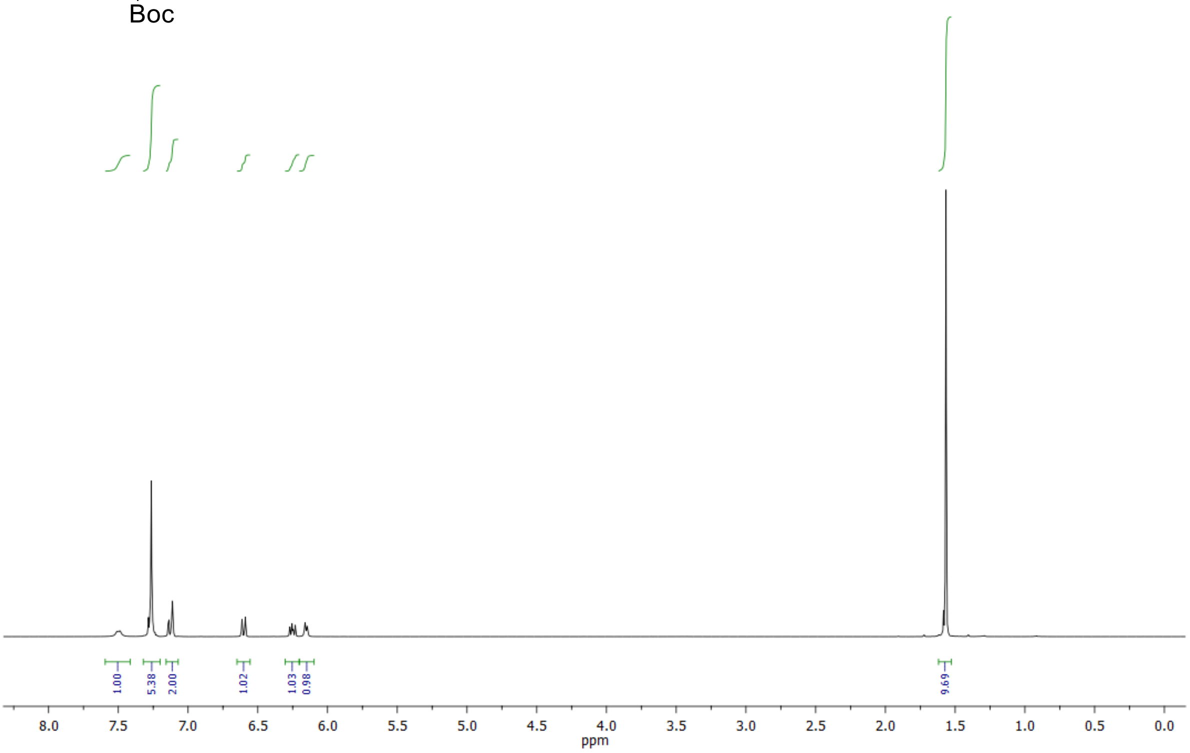

$^{13}C\{1H\}$ NMR spectrum ($CDCl_3$, 100 MHz):

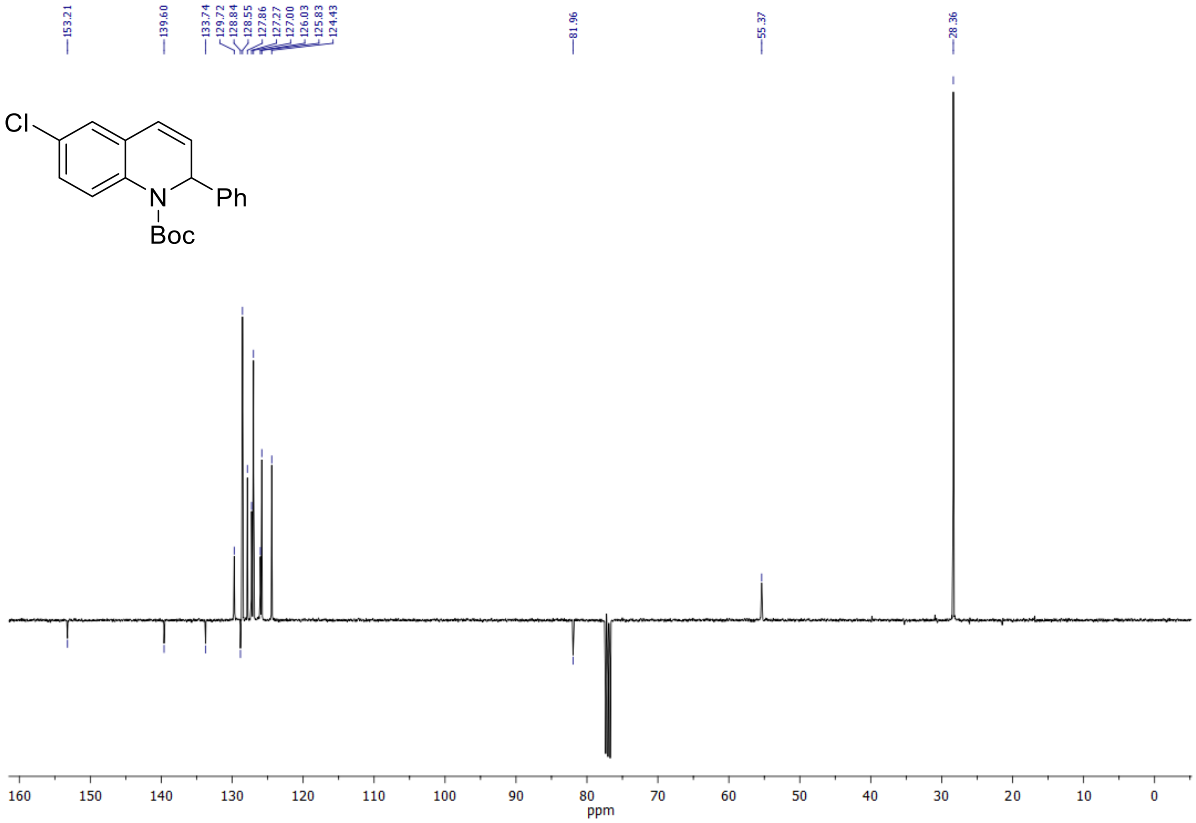

^{1}H NMR spectrum ($CDCl_3$, 400 MHz):

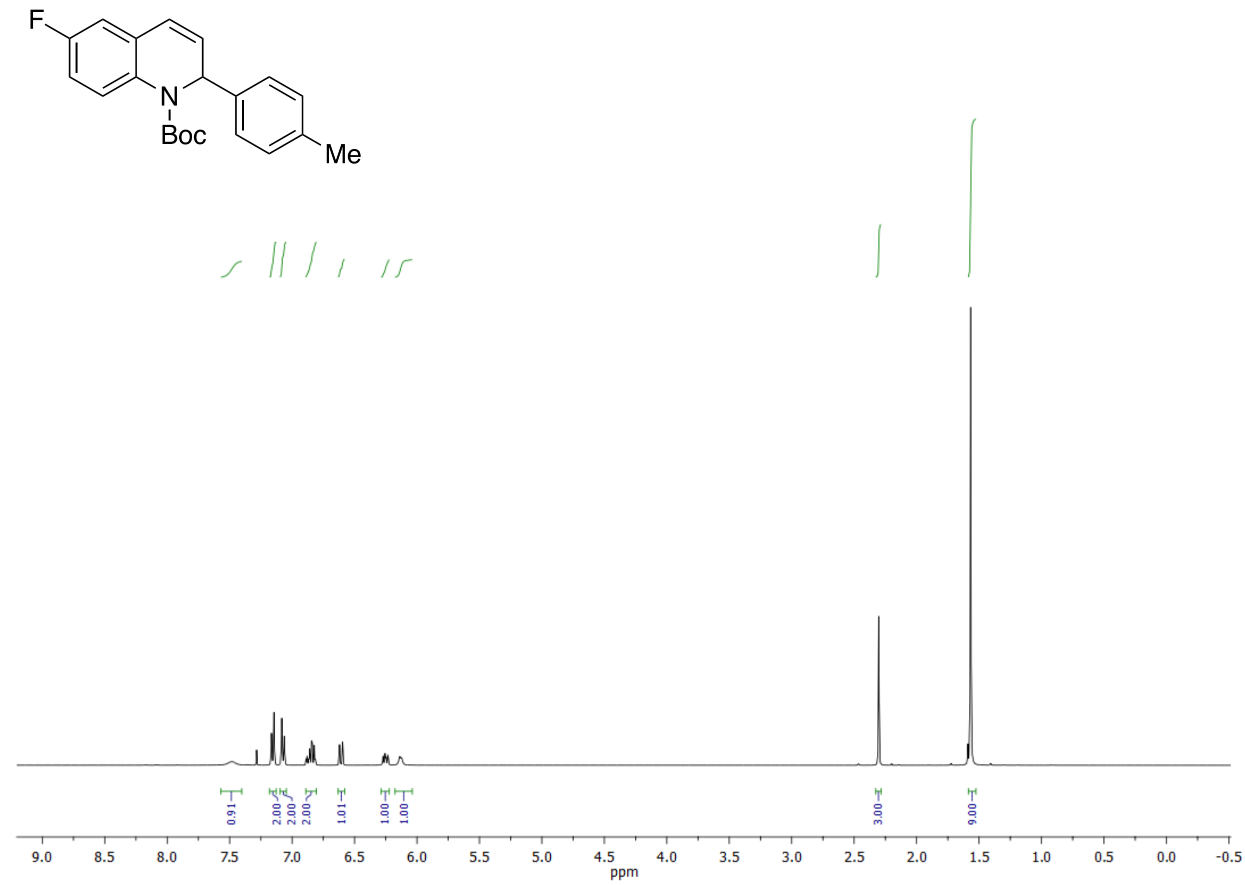

$^{13}C\{1H\}$ NMR spectrum ($CDCl_3$, 100 MHz):

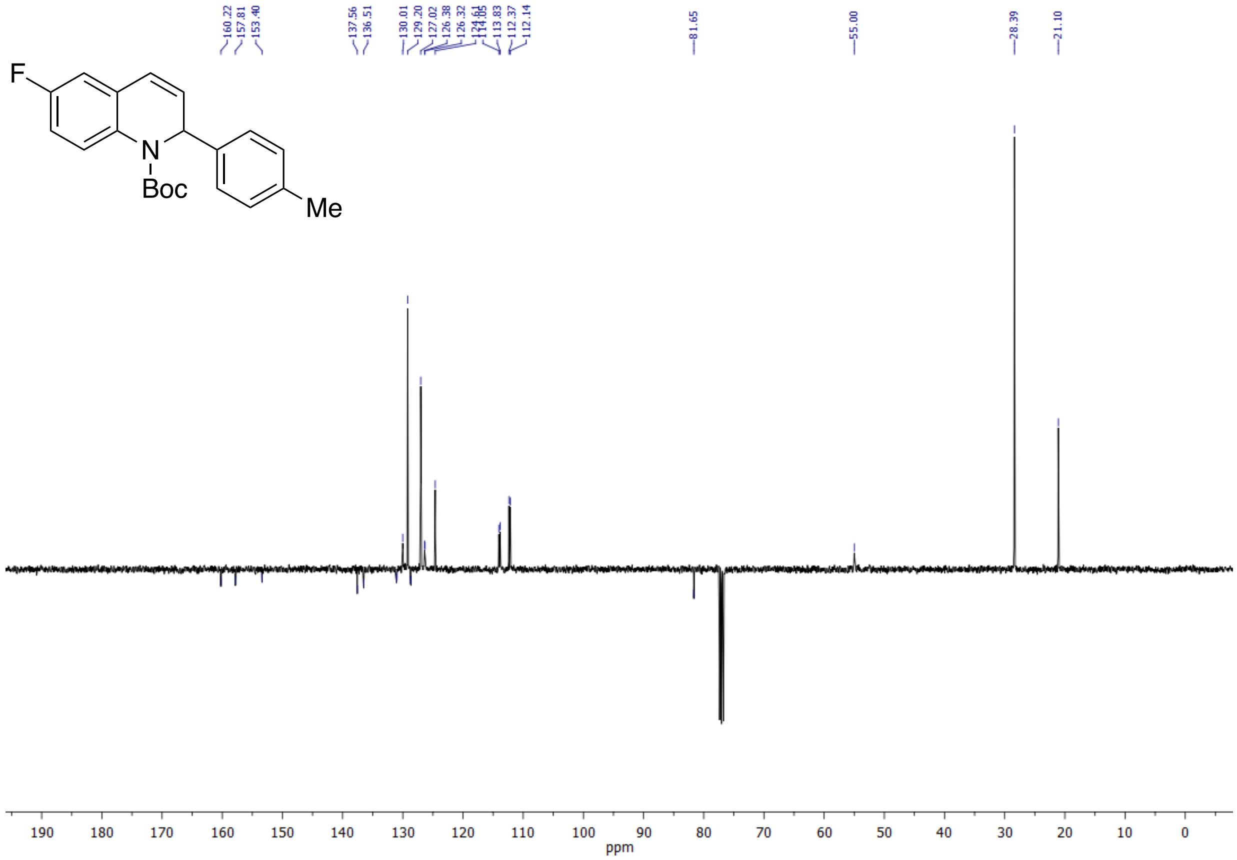

^{19}F NMR spectrum ($CDCl_3$, 377 MHz):

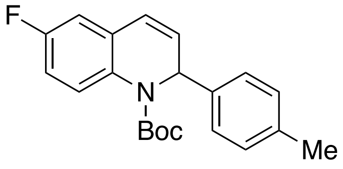

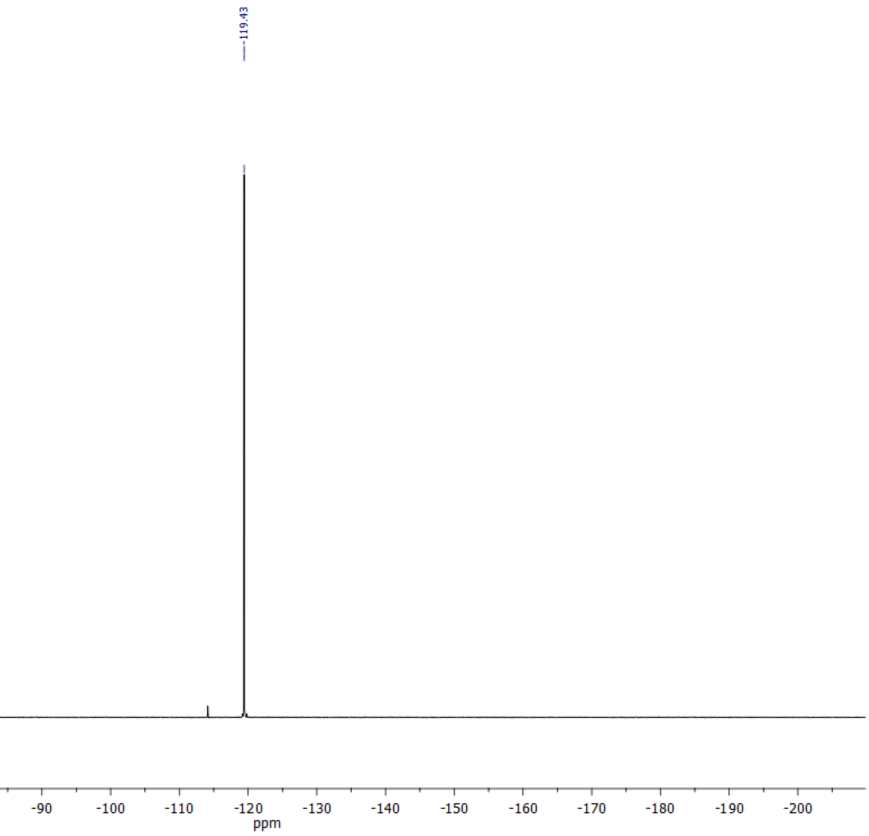

^{1}H NMR spectrum ($CDCl_3$, 400 MHz):

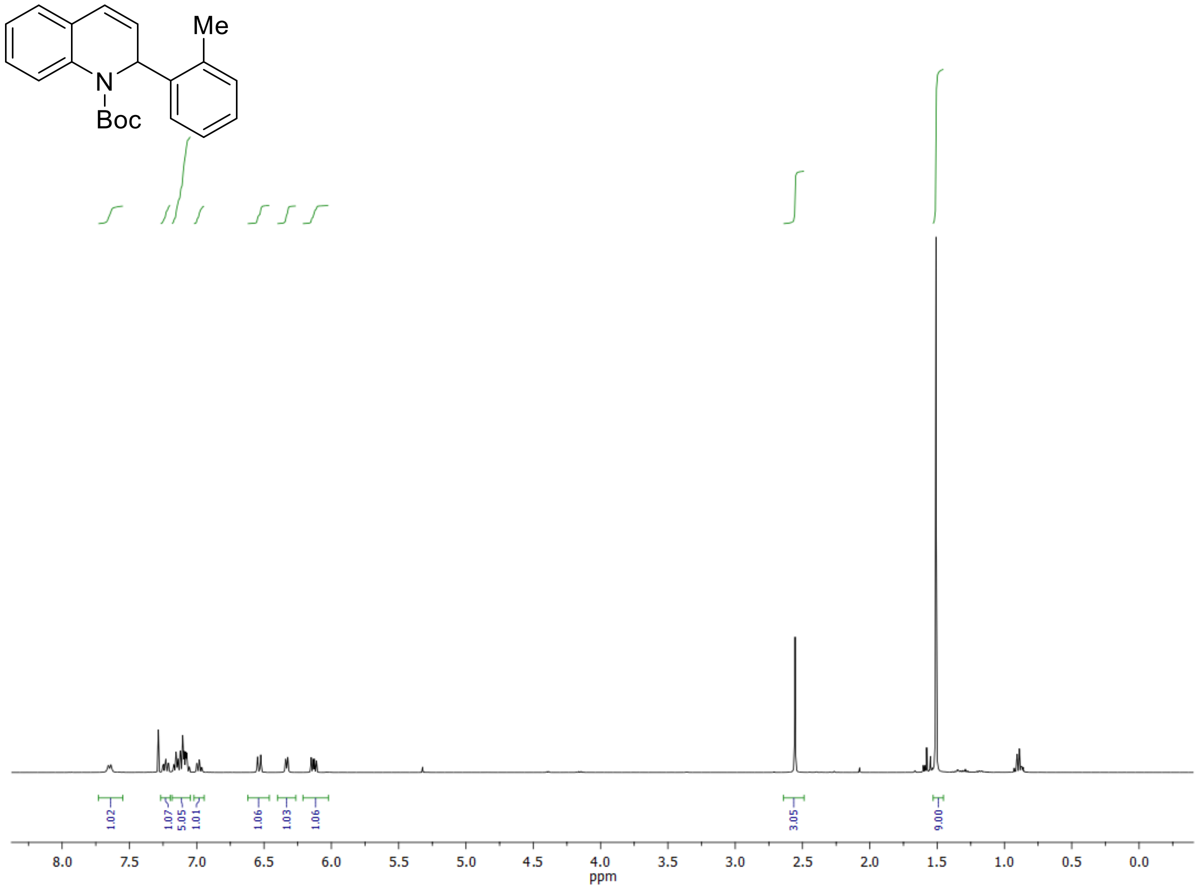

^{13}C{1H} NMR spectrum ($CDCl_3$, 100 MHz):

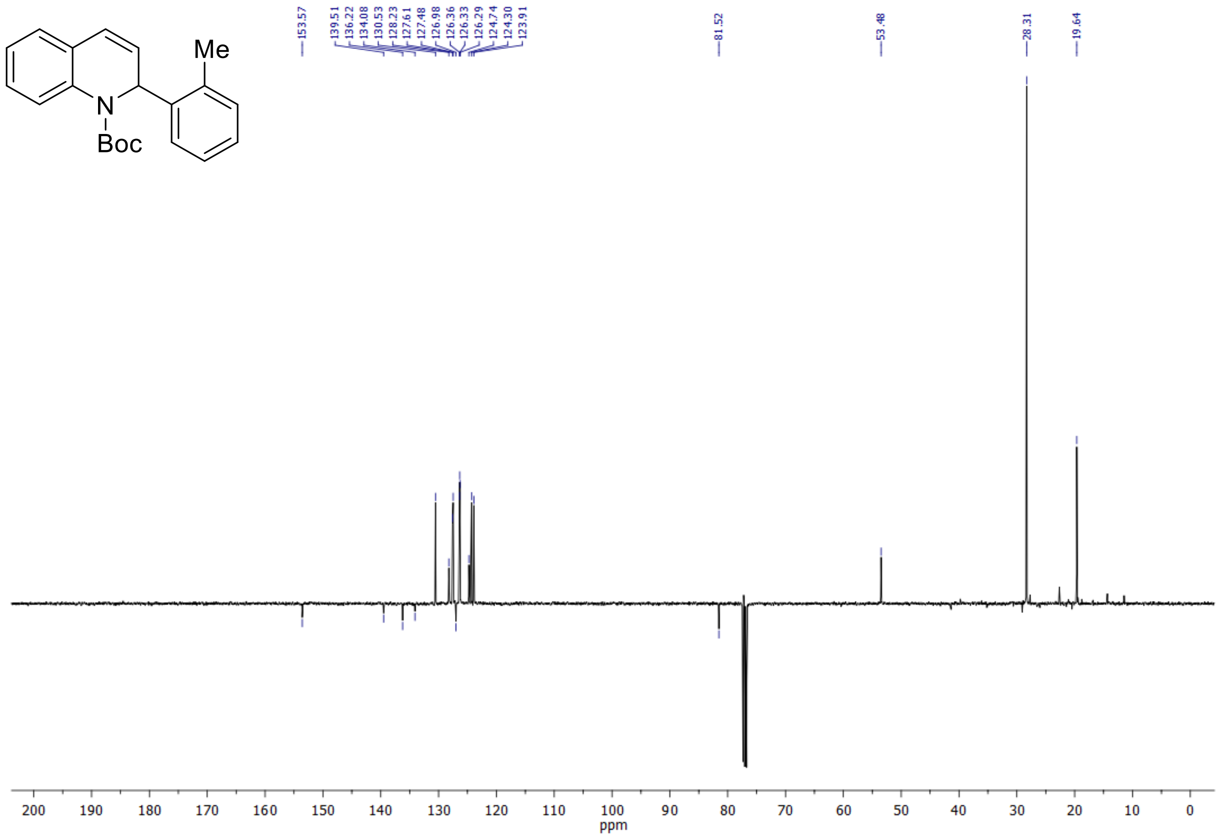

^{1}H NMR spectrum ($CDCl_3$, 400 MHz):

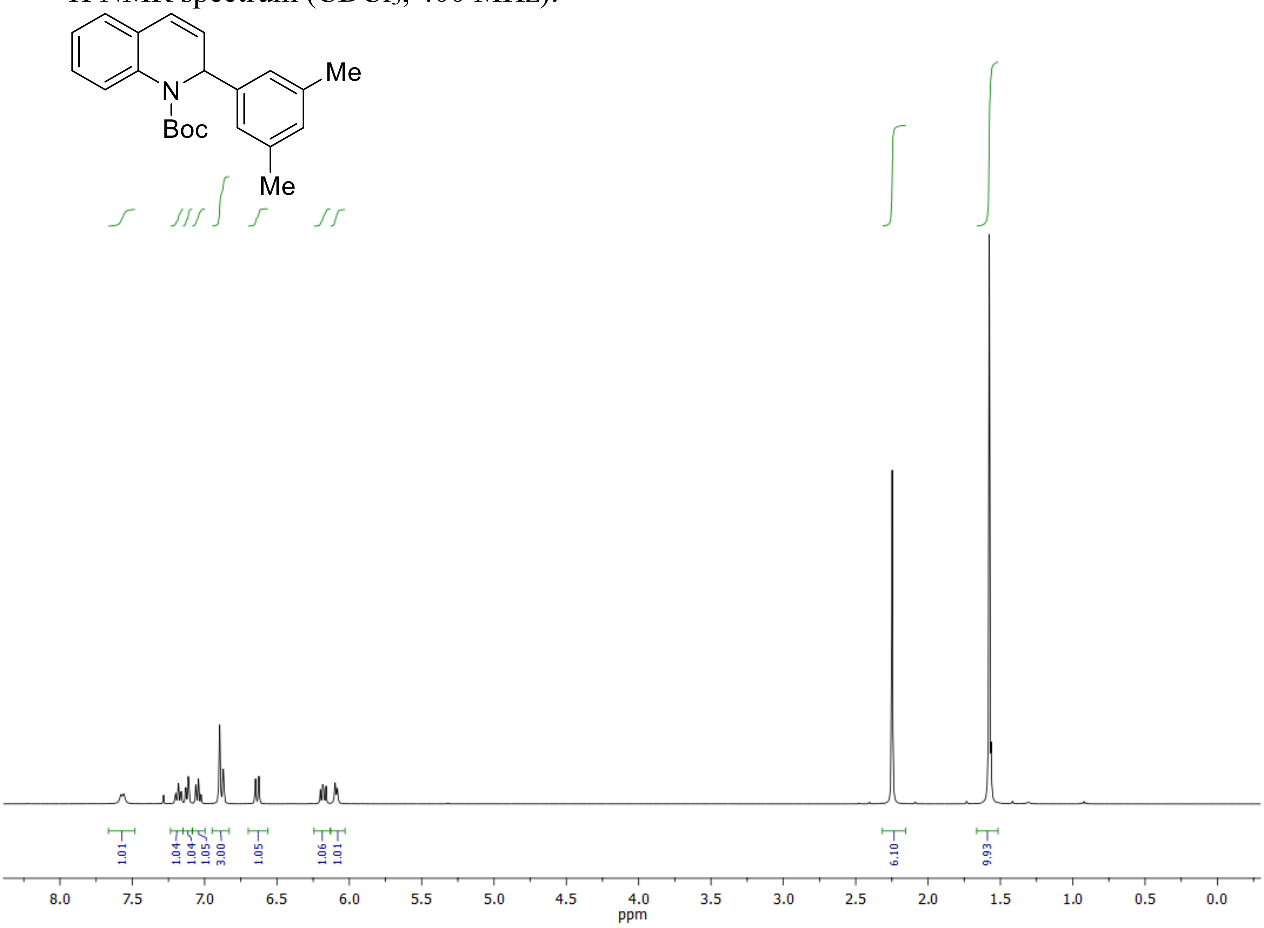

$^{13}C\{1H\}$ NMR spectrum ($CDCl_3$, 100 MHz):

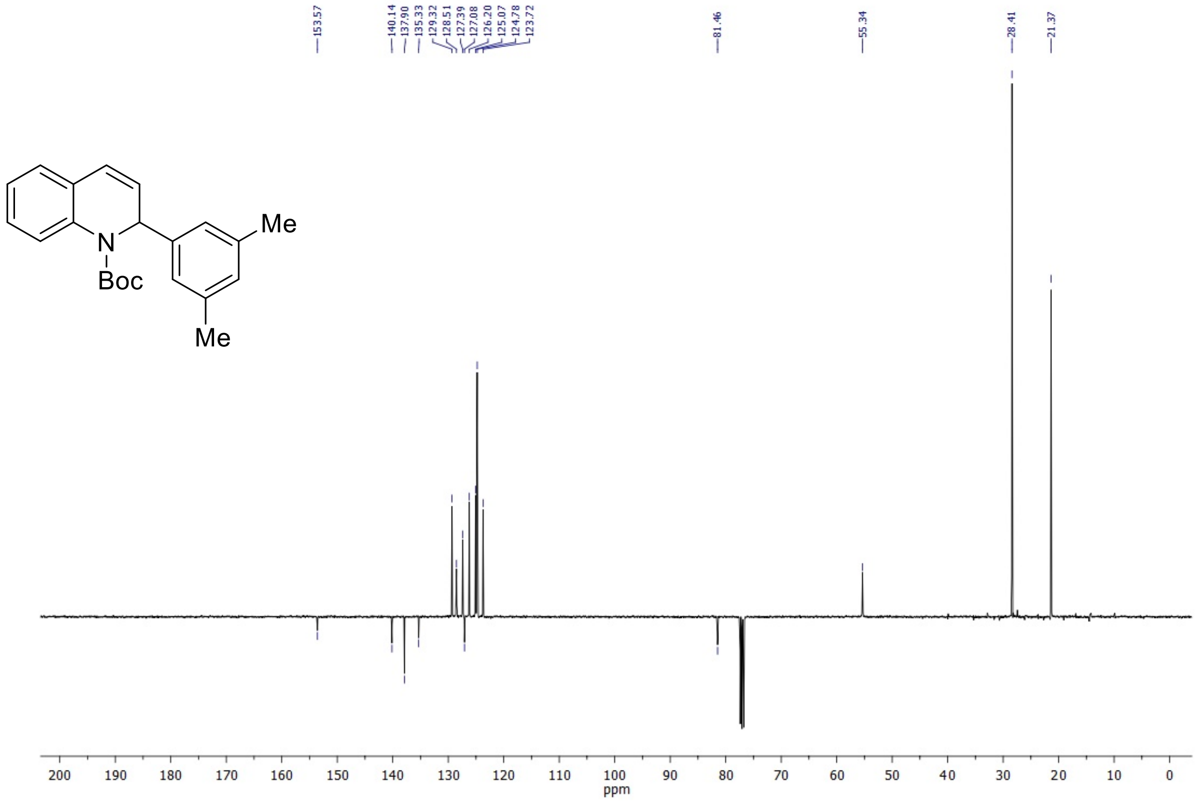

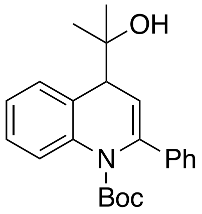

^{1}H NMR spectrum ($CDCl_3$, 400 MHz):

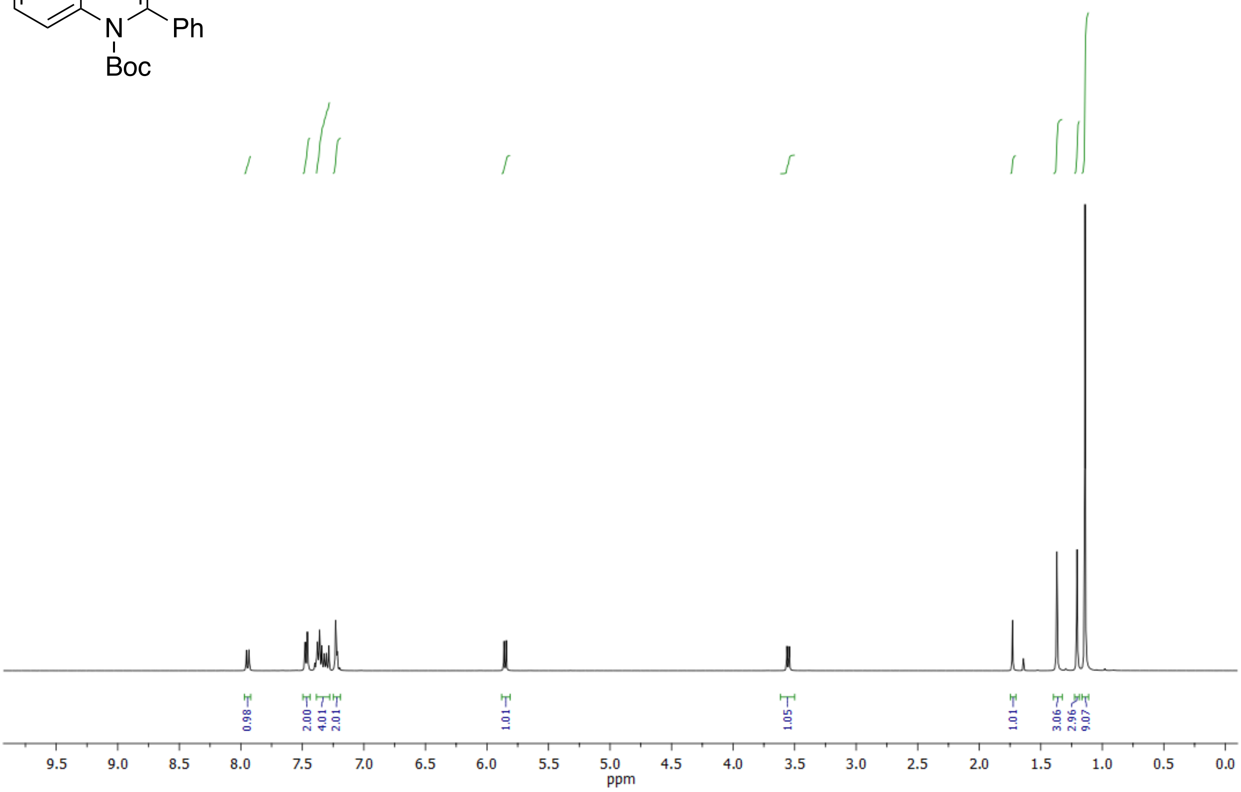

$^{13}C\{1H\}$ NMR spectrum ($CDCl_3$, 100 MHz):

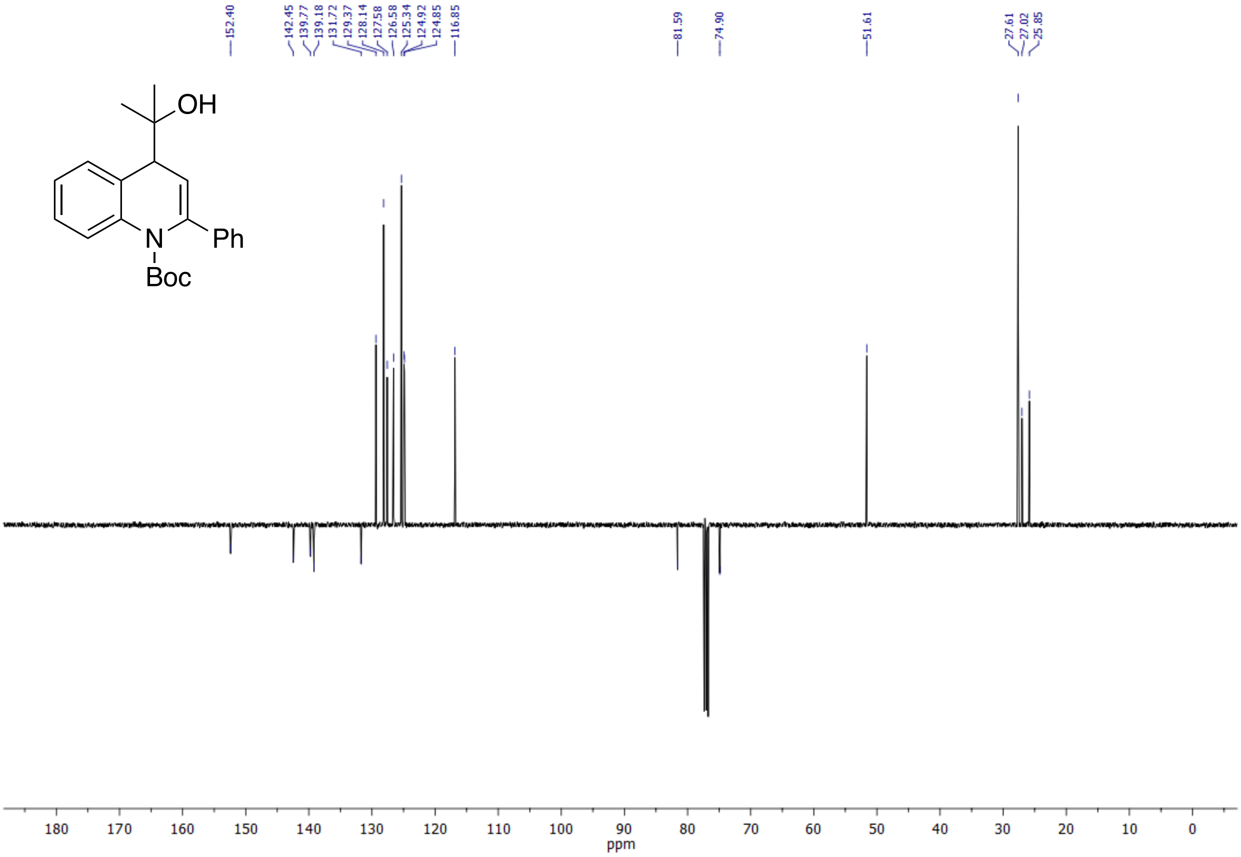

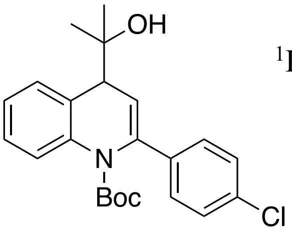

^{1}H NMR spectrum ($CDCl_3$, 400 MHz):

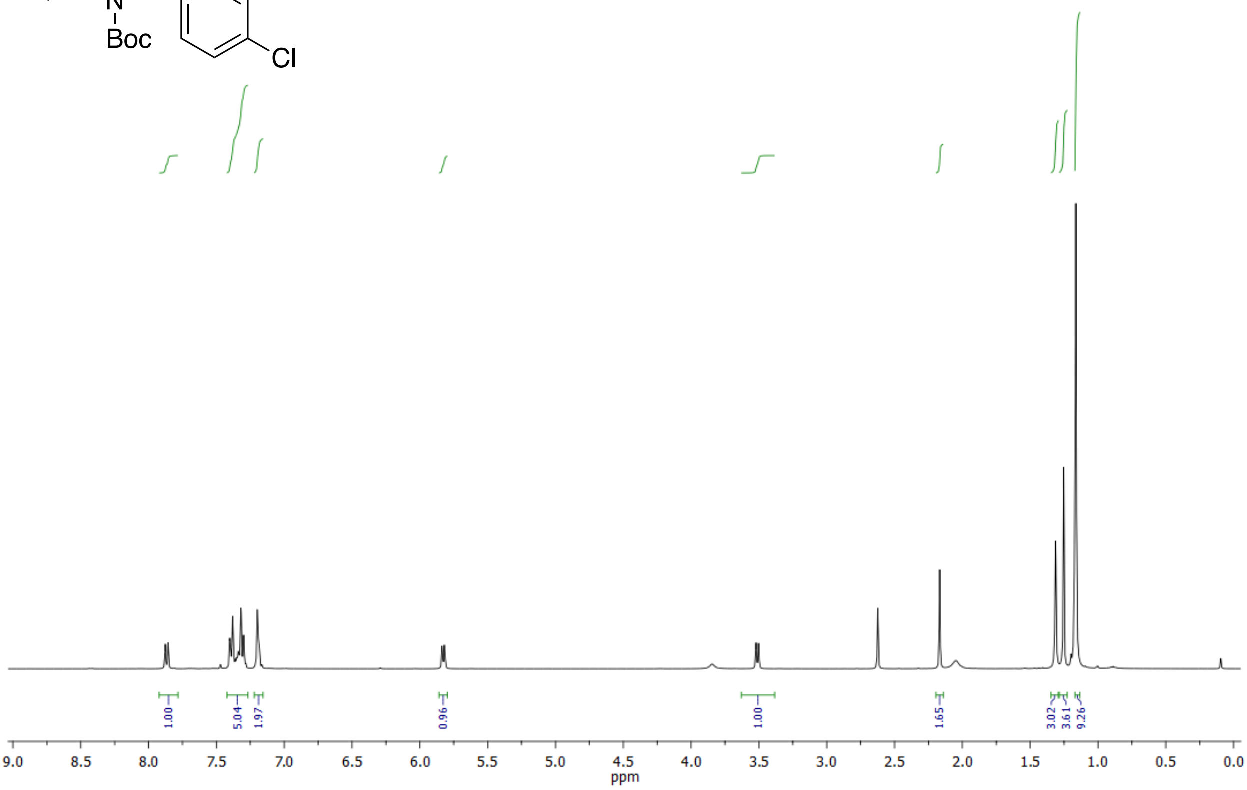

^{13}C{1H} NMR spectrum ($CDCl_3$, 100 MHz):

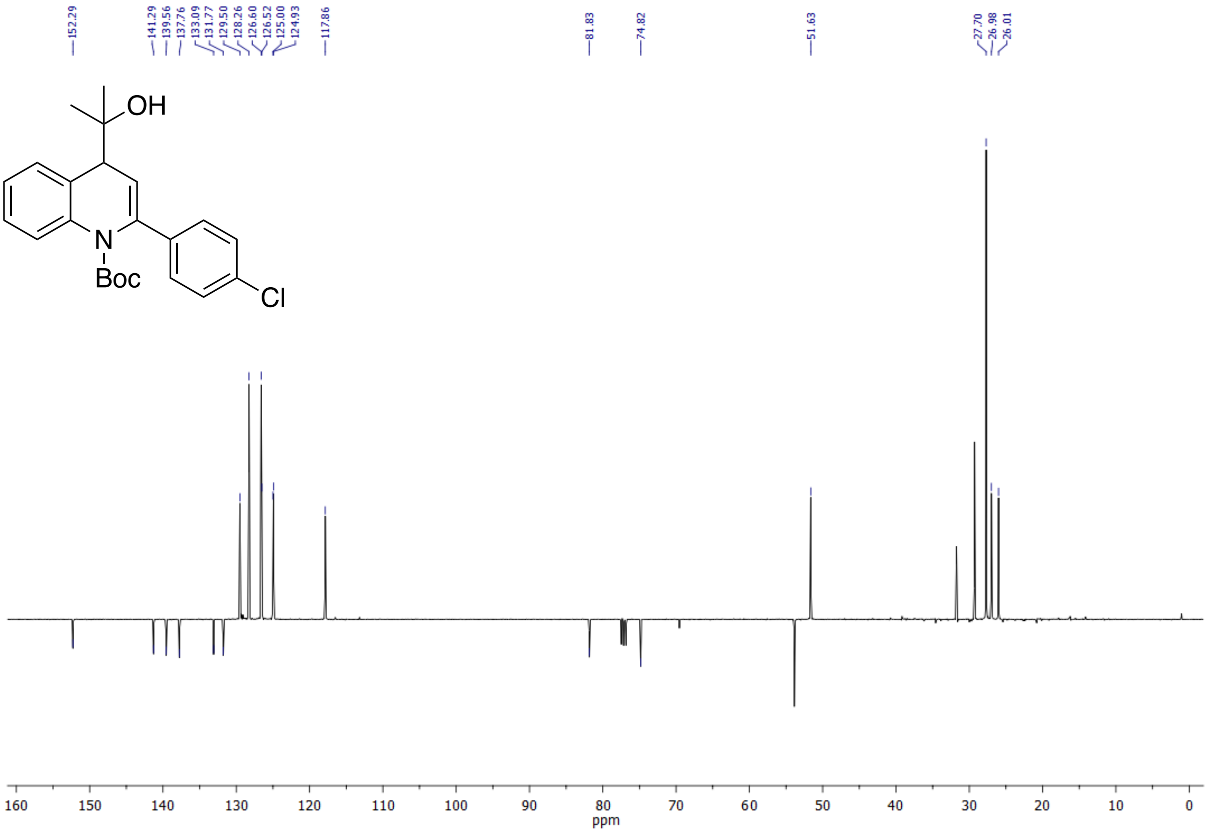

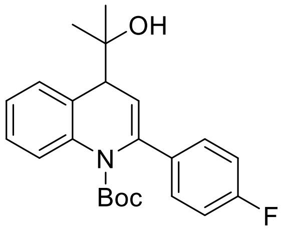

^{1}H NMR spectrum ($CDCl_3$, 400 MHz):

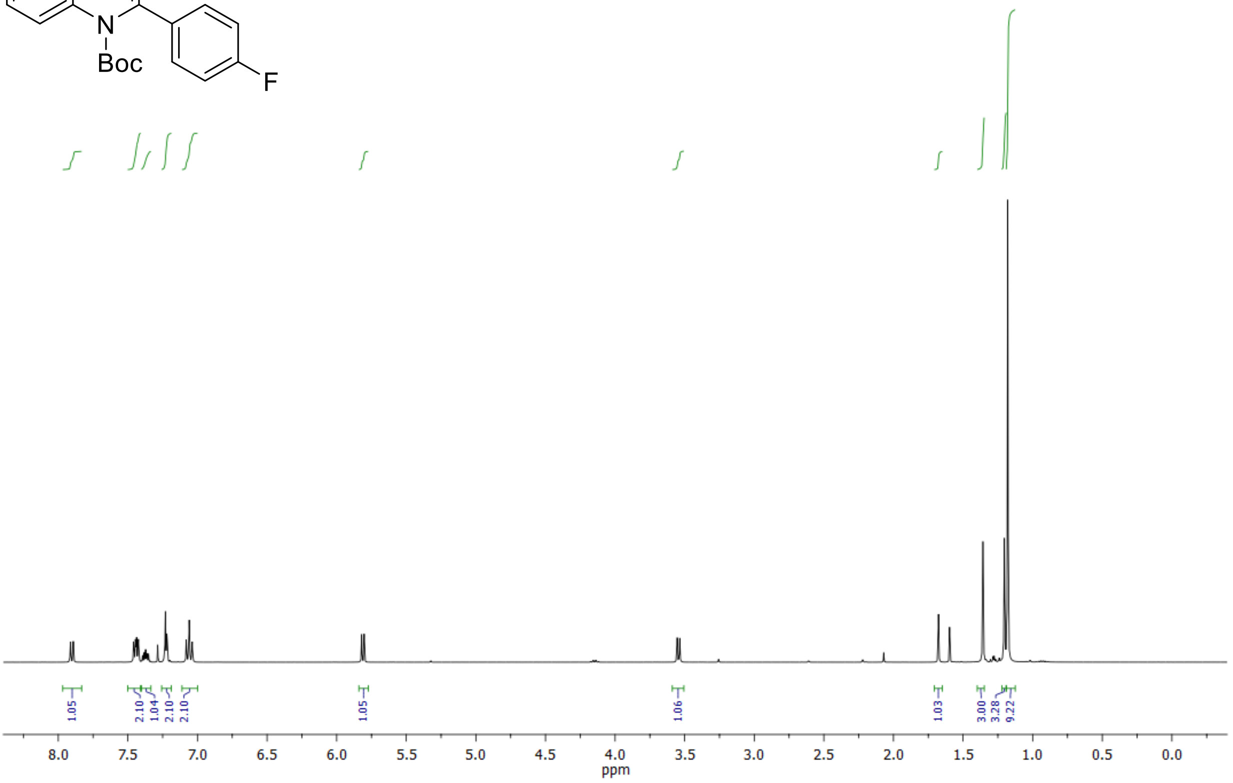

^{13}C{1H} NMR spectrum ($CDCl_3$, 100 MHz):

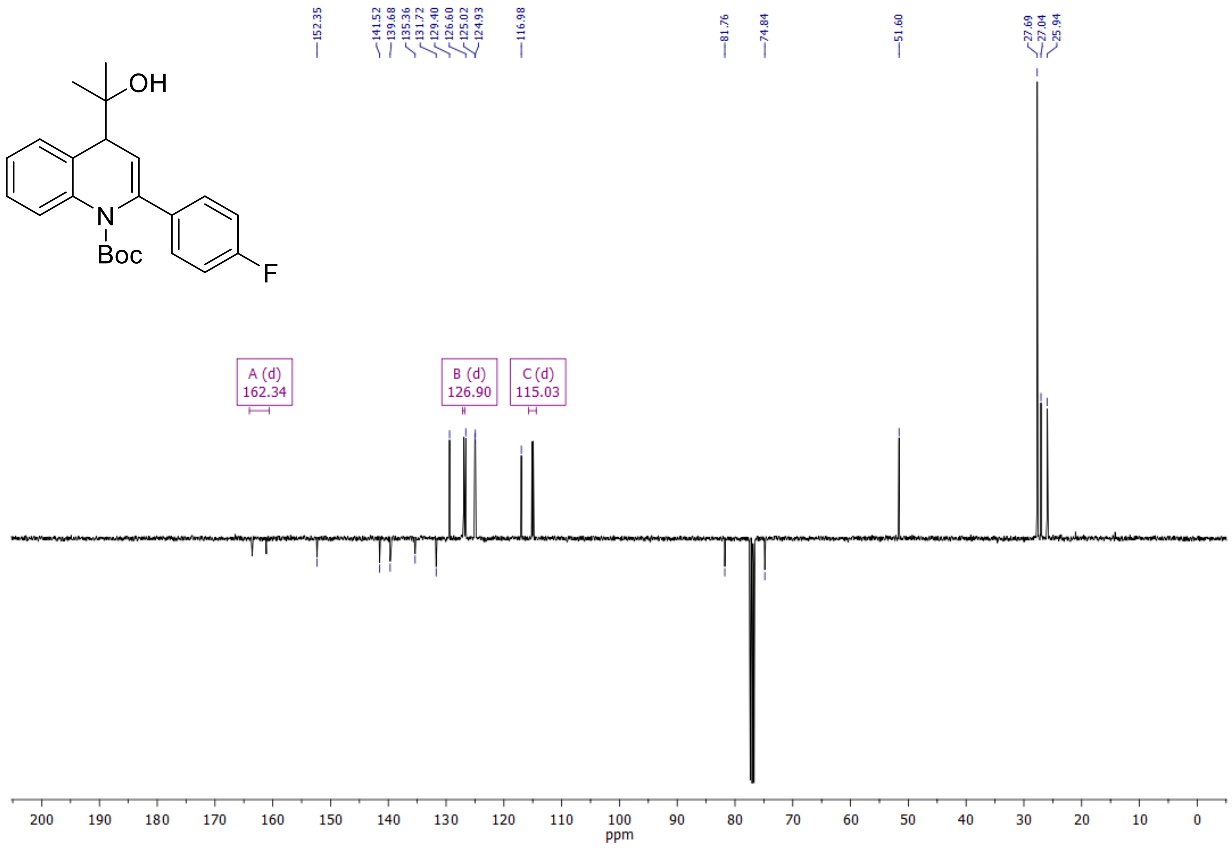

^{19}F NMR spectrum ($CDCl_3$, 377 MHz):

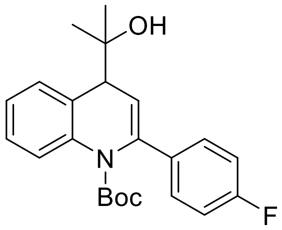

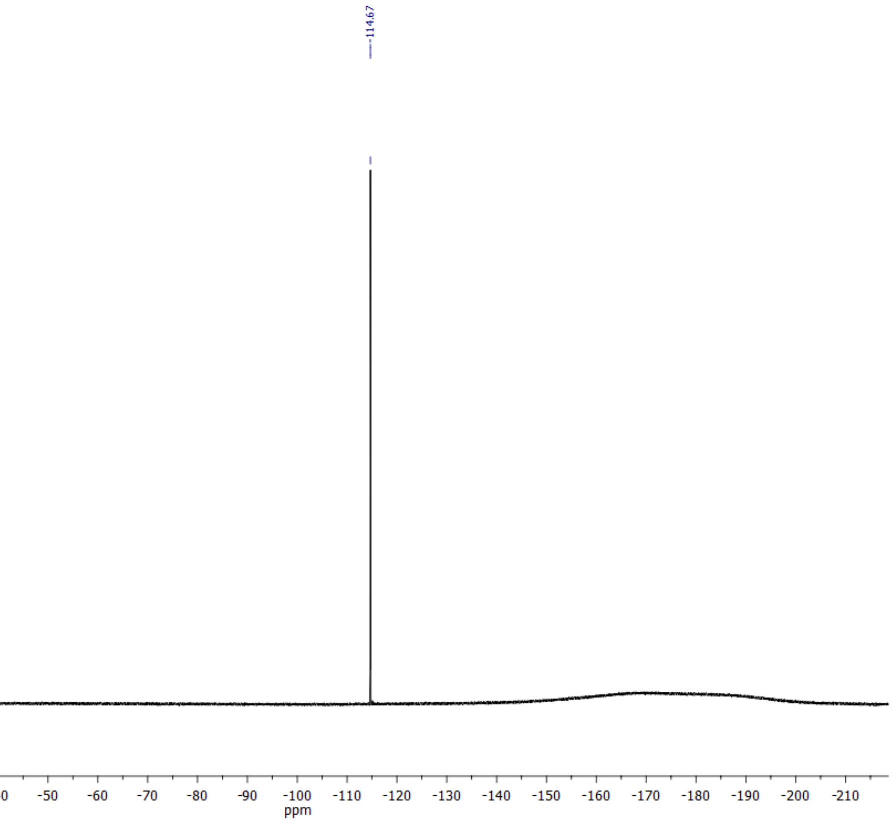

^{1}H NMR spectrum ($CDCl_3$, 400 MHz):

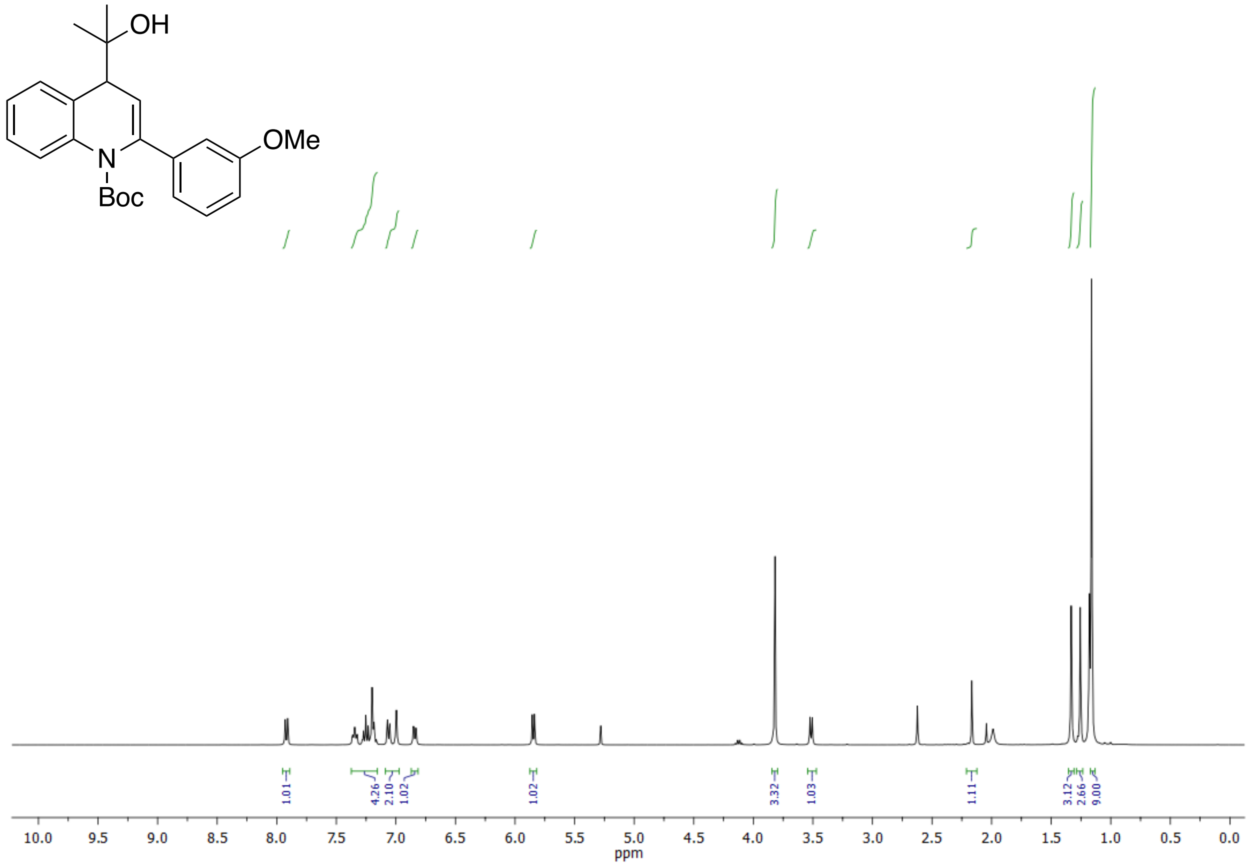

$^{13}C\{1H\}$ NMR spectrum ($CDCl_3$, 100 MHz):

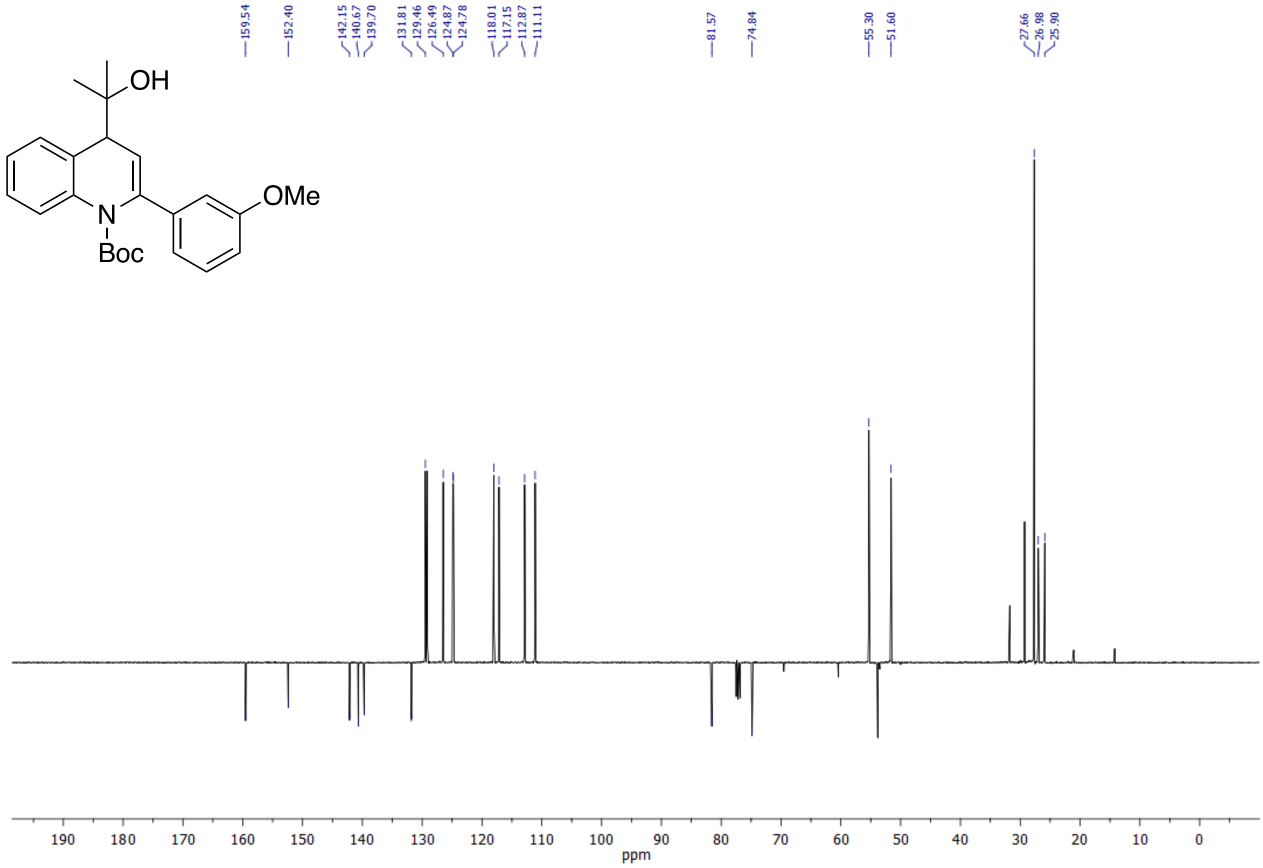

^{1}H NMR spectrum ($CDCl_3$, 400 MHz):

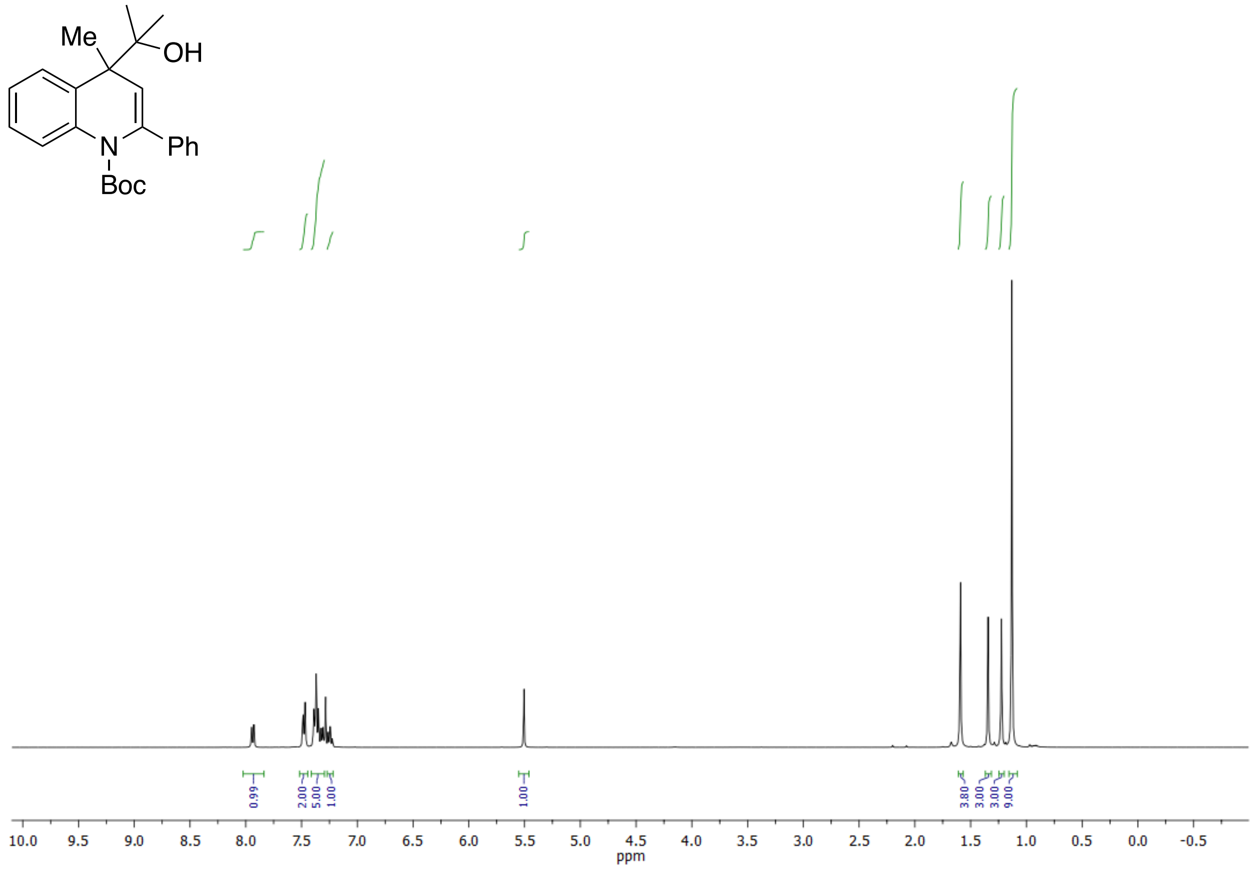

$^{13}C\{1H\}$ NMR spectrum ($CDCl_3$, 100 MHz):

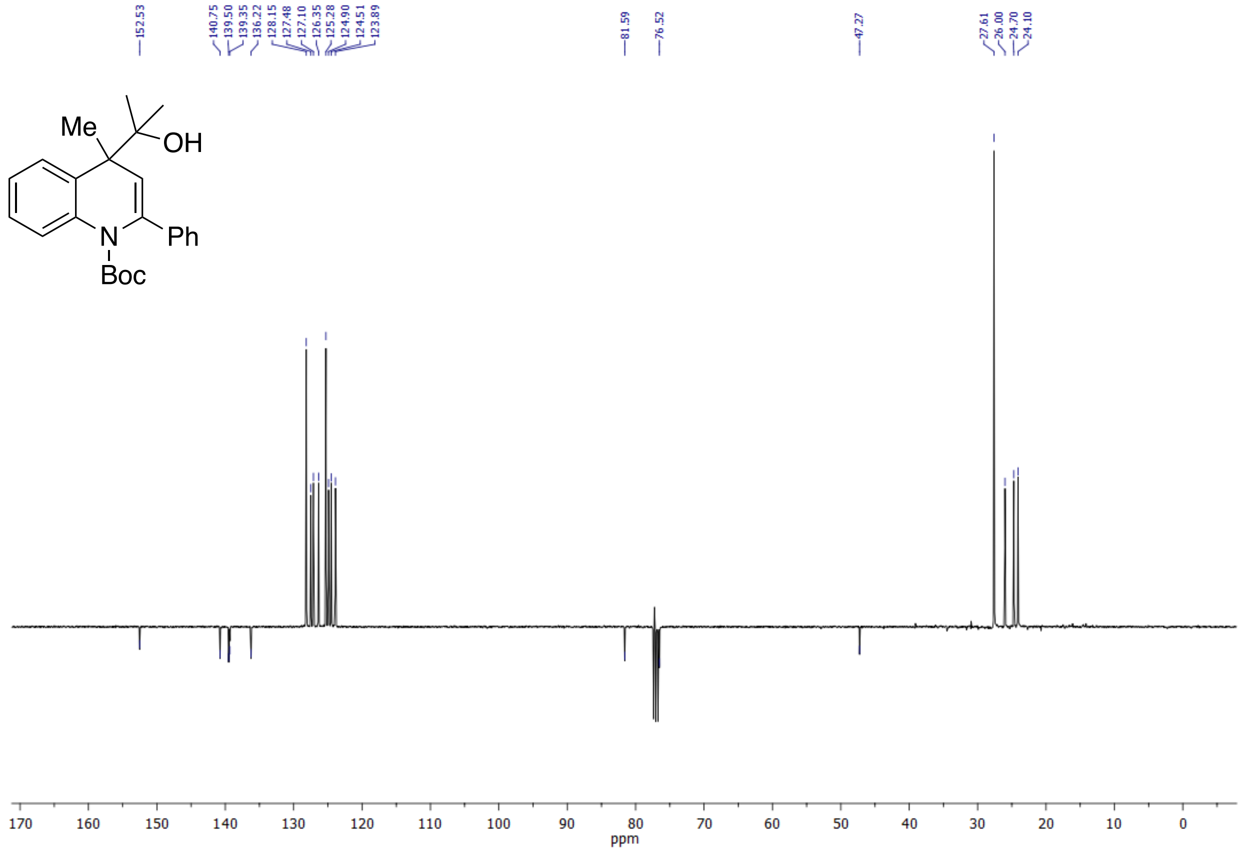

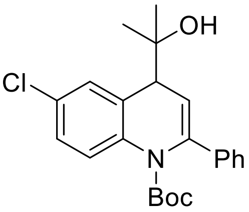

^{1}H NMR spectrum ($CDCl_3$, 400 MHz):

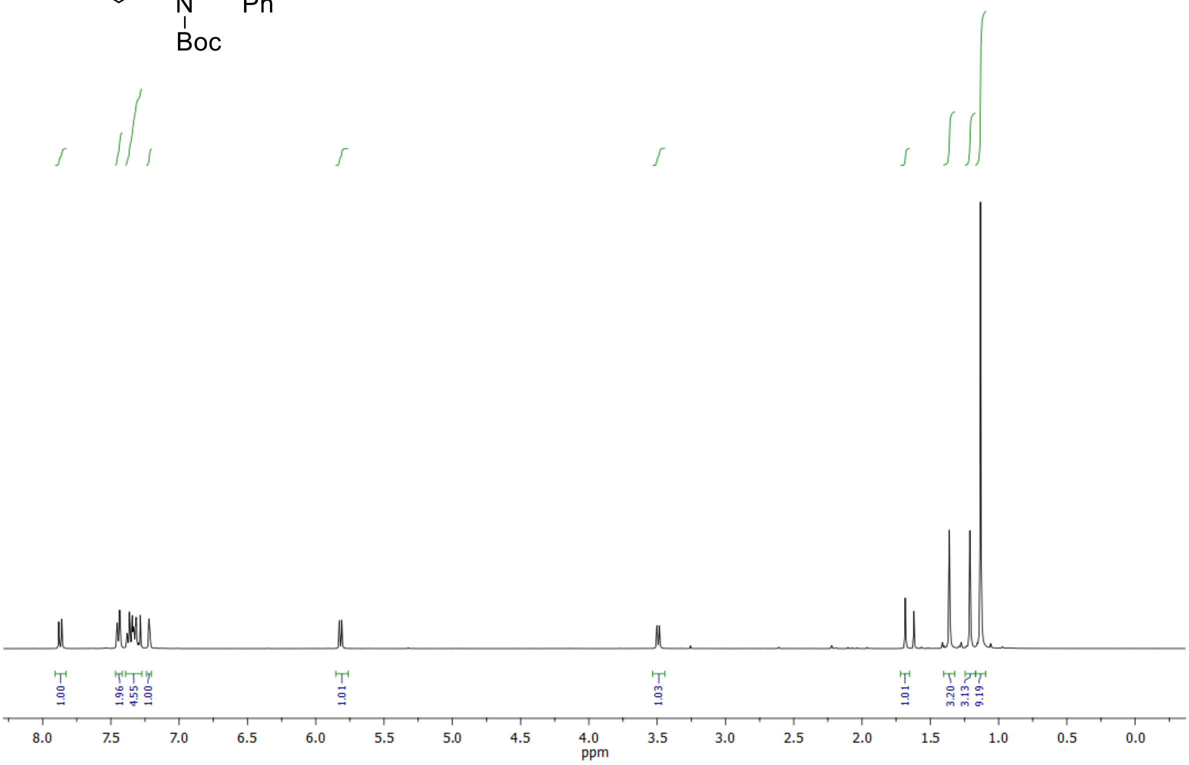

^{13}C{1H} NMR spectrum ($CDCl_3$, 100 MHz):

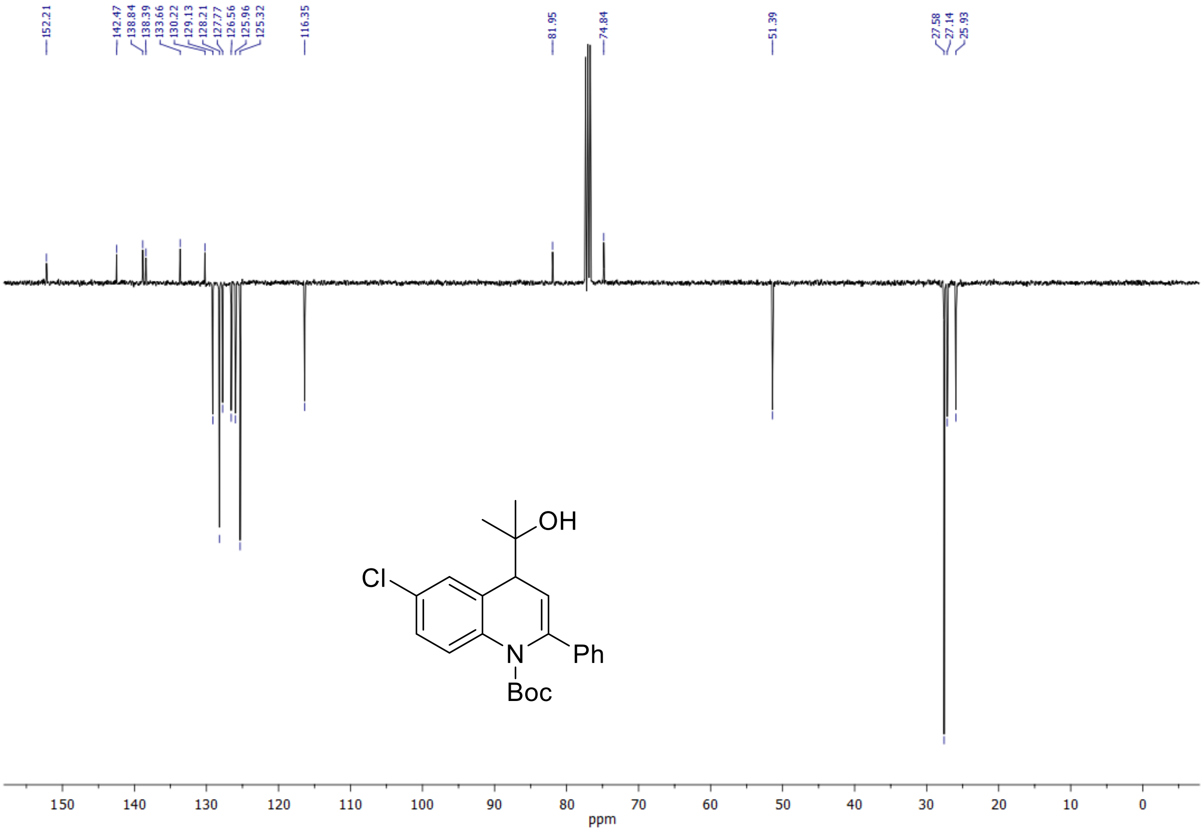

^{1}H NMR spectrum ($CDCl_3$, 400 MHz):

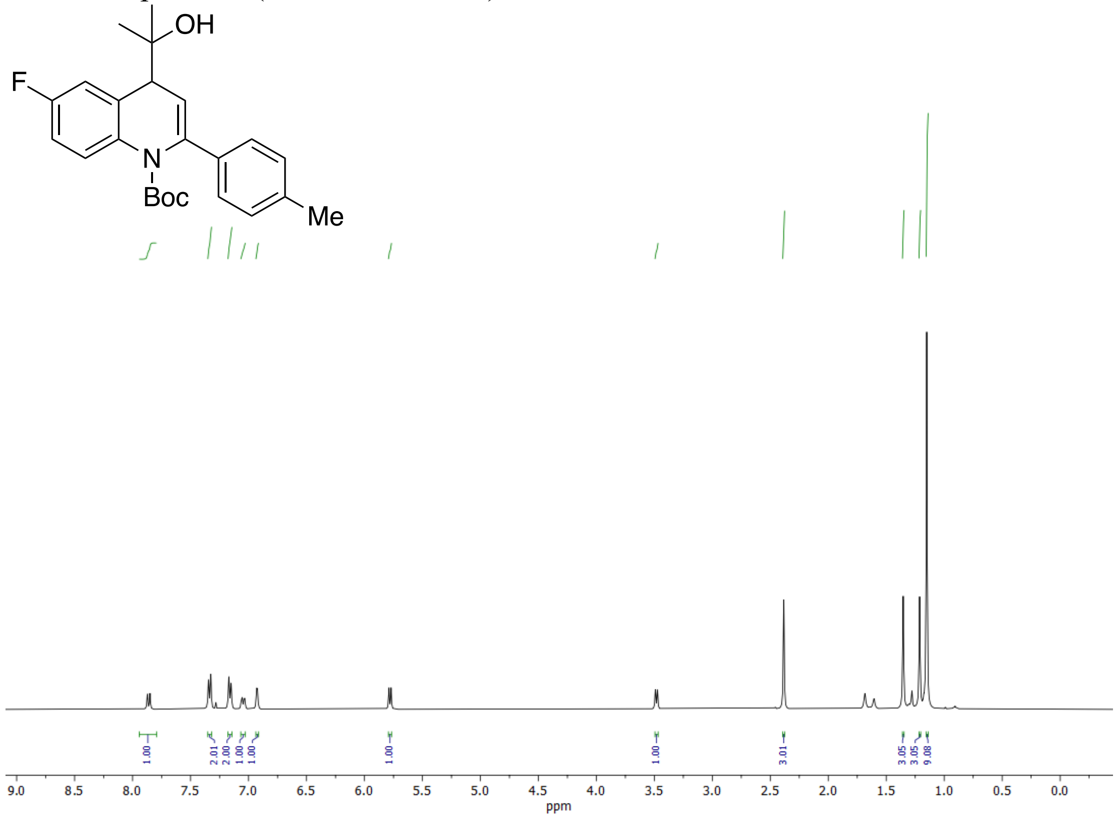

$^{13}C\{1H\}$ NMR spectrum ($CDCl_3$, 100 MHz):

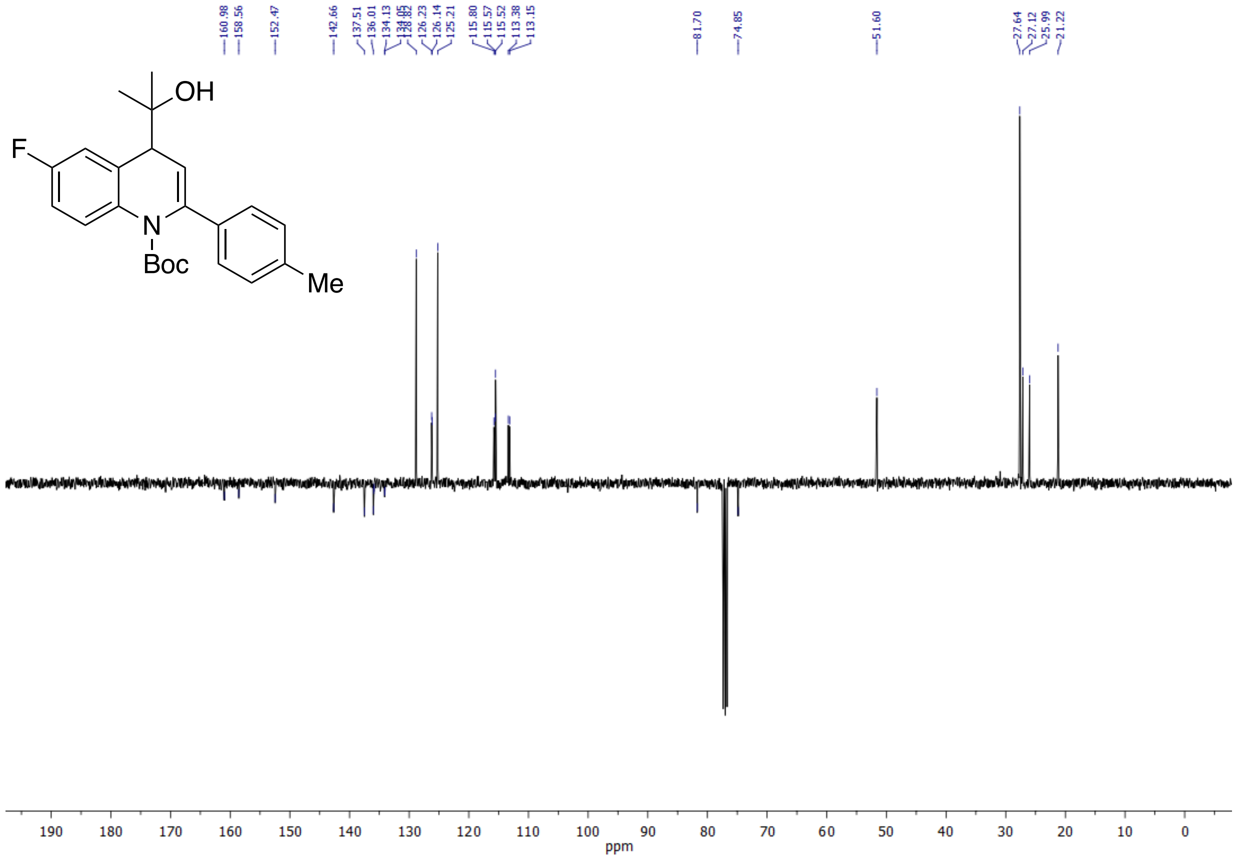

^{19}F NMR spectrum ($CDCl_3$, 377 MHz):

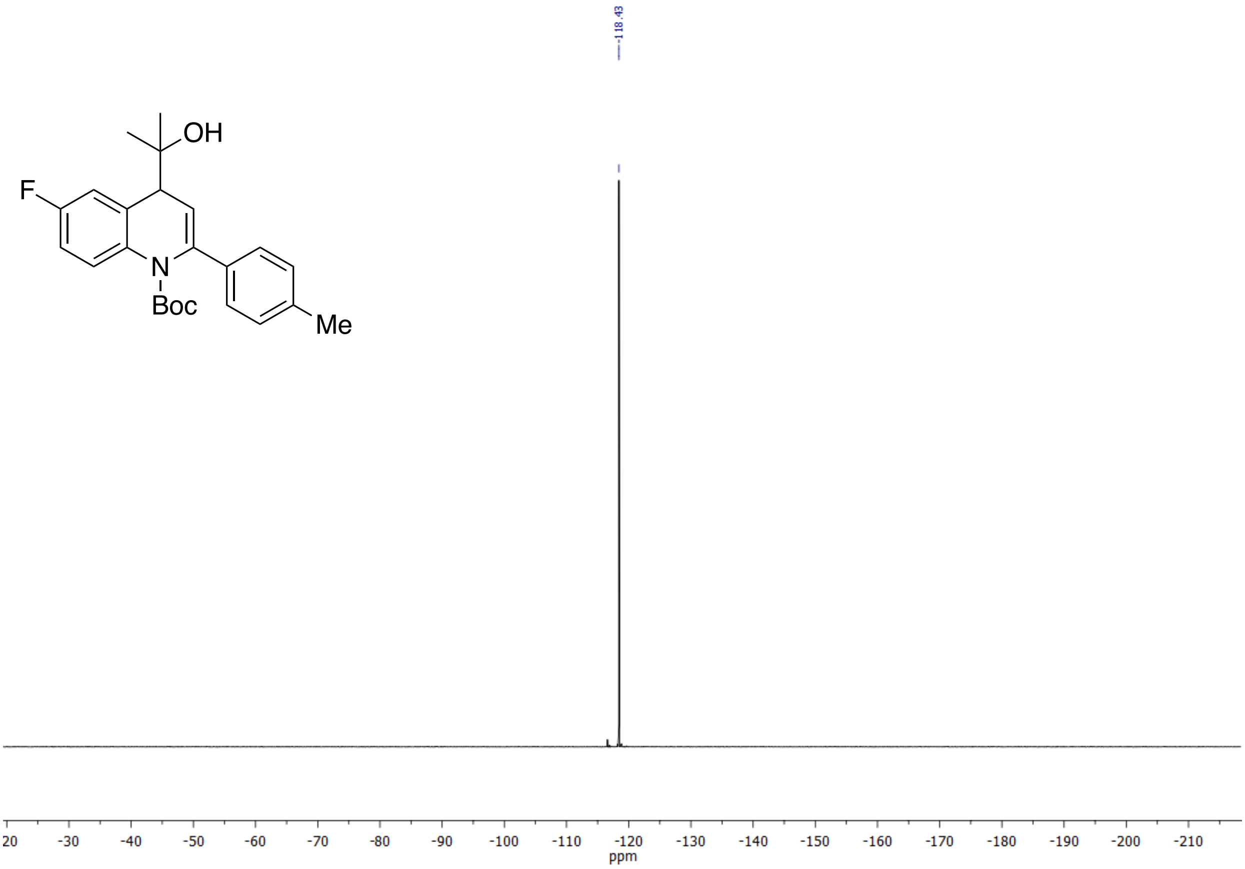

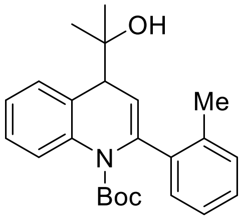

^{1}H NMR spectrum ($CDCl_3$, 400 MHz):

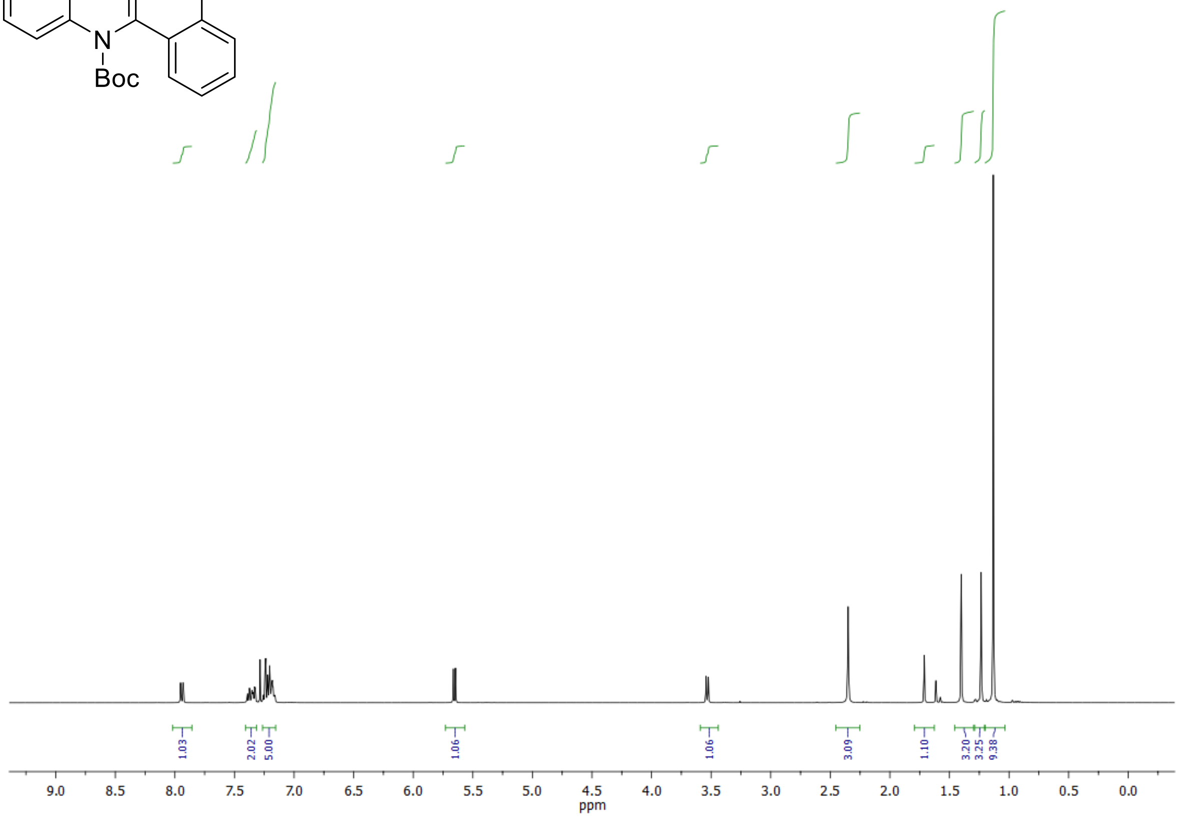

$^{13}C\{1H\}$ NMR spectrum ($CDCl_3$, 100 MHz):

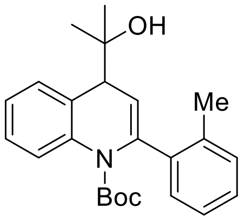

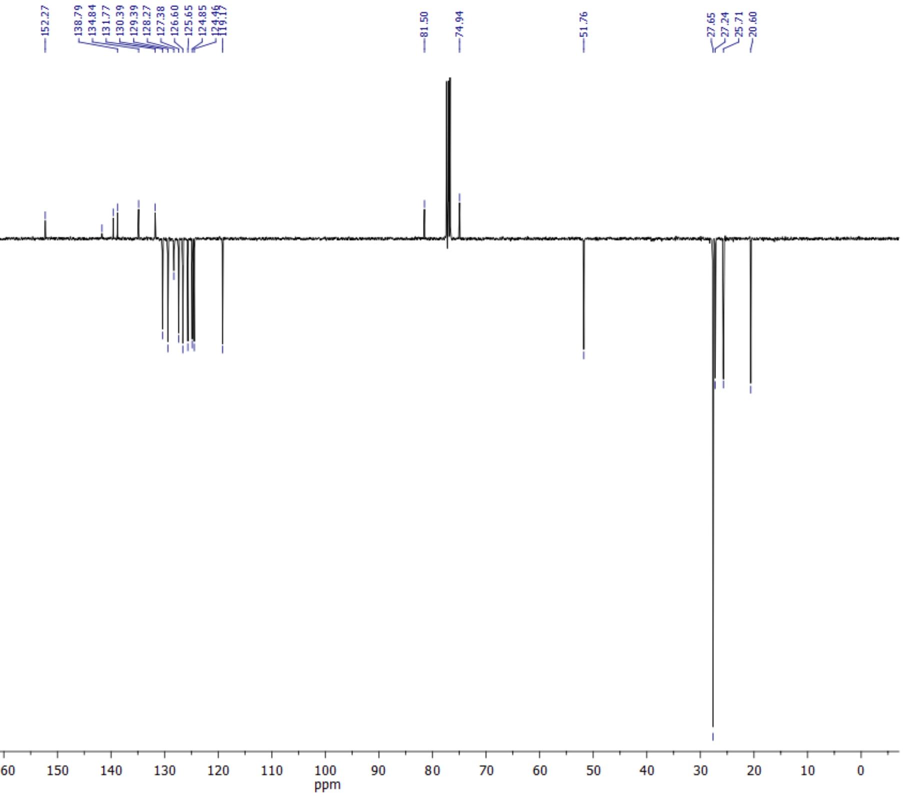

^{1}H NMR spectrum ($CDCl_3$, 400 MHz):

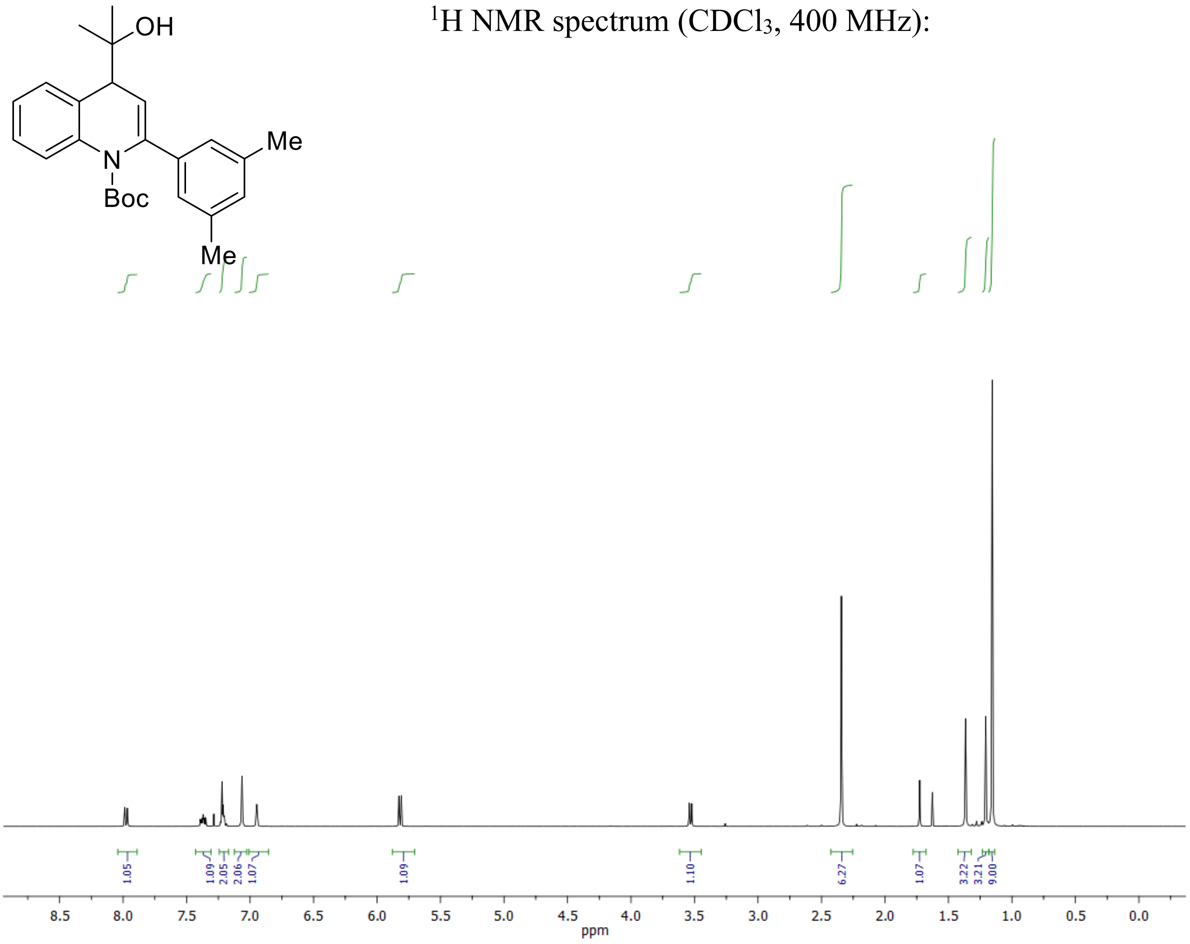

^{13}C{1H} NMR spectrum ($CDCl_3$, 100 MHz):

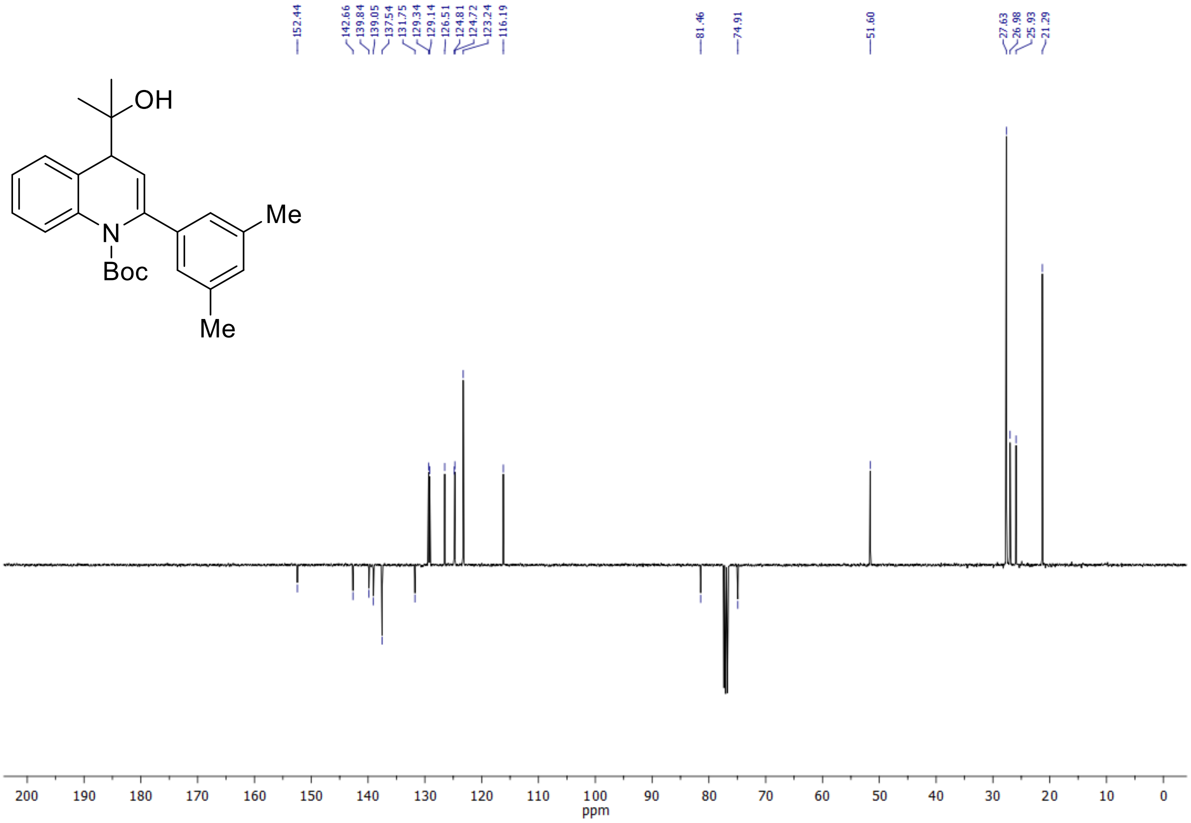

^{1}H NMR spectrum ($CDCl_3$, 400 MHz):

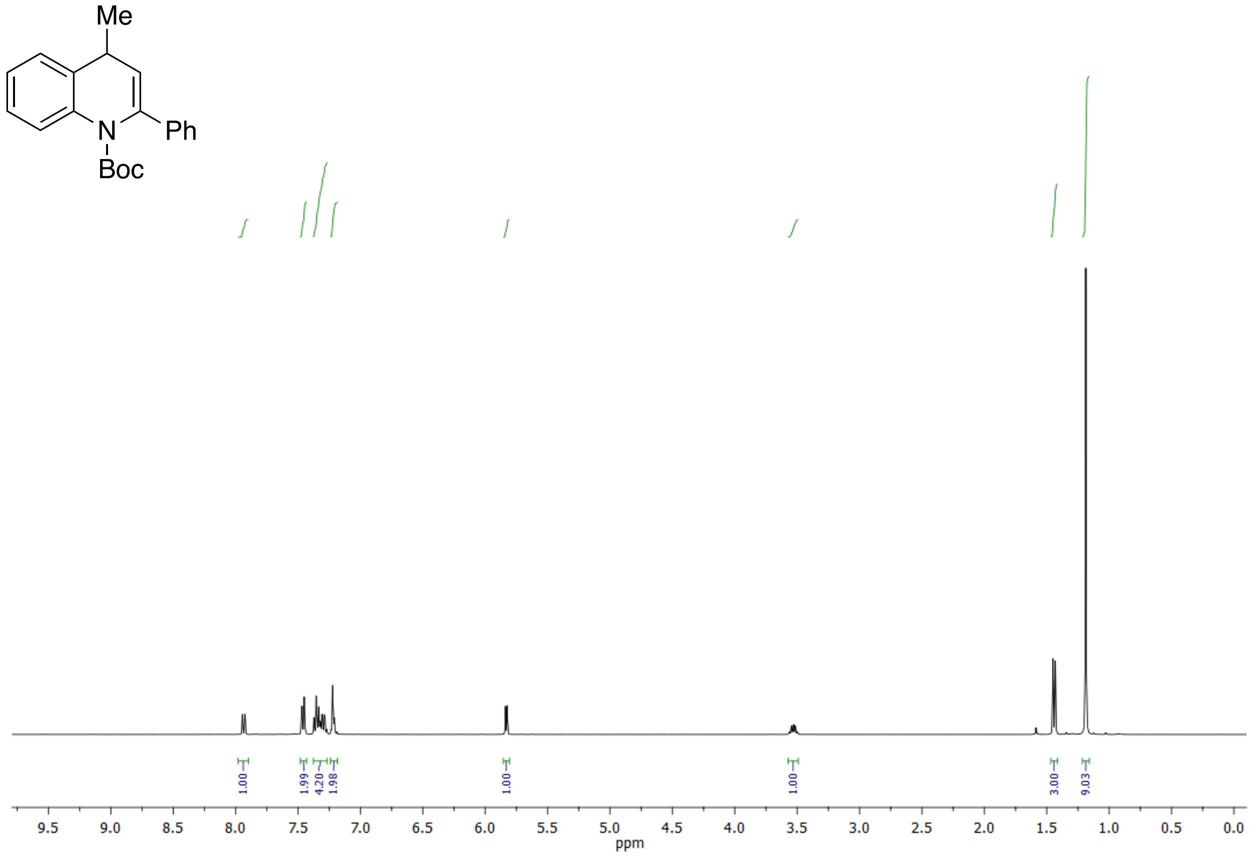

^{13}C{1H} NMR spectrum ($CDCl_3$, 100 MHz):

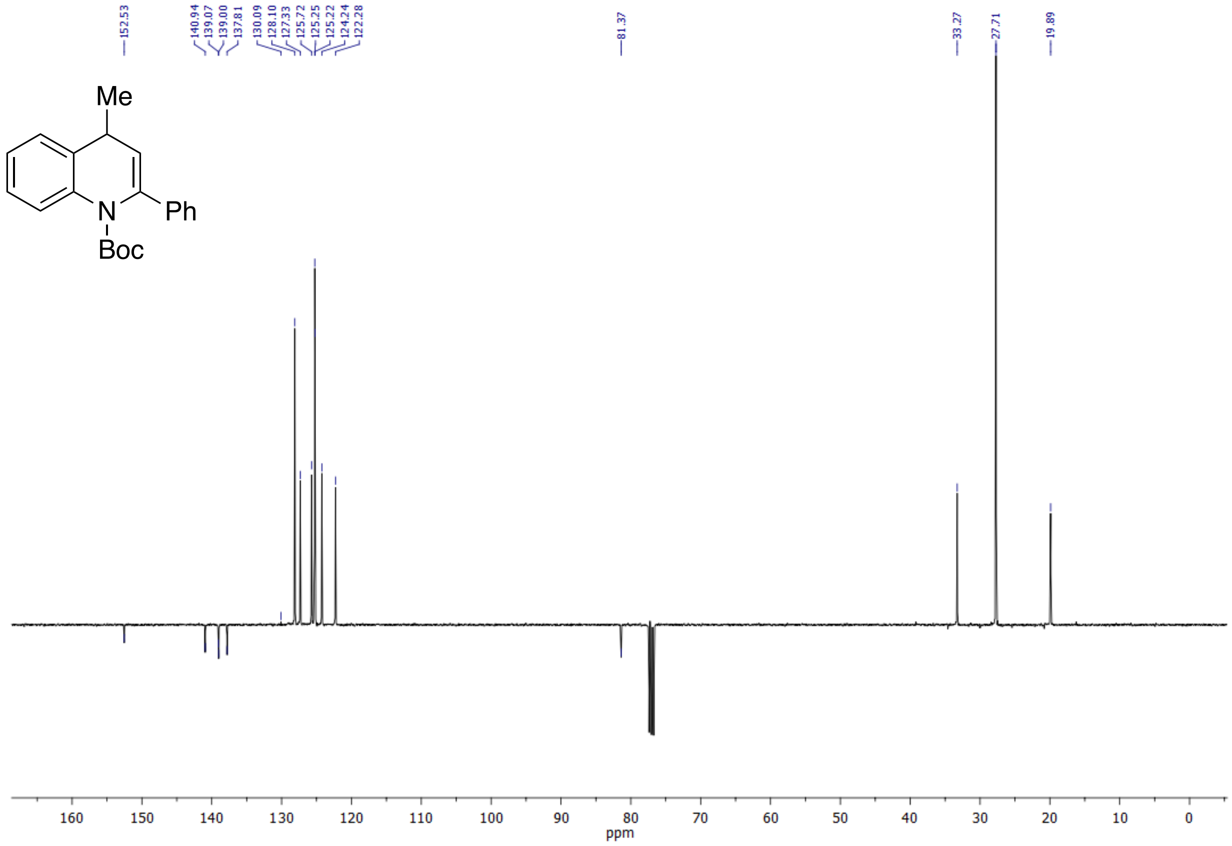

^{1}H NMR spectrum ($CDCl_3$, 400 MHz):

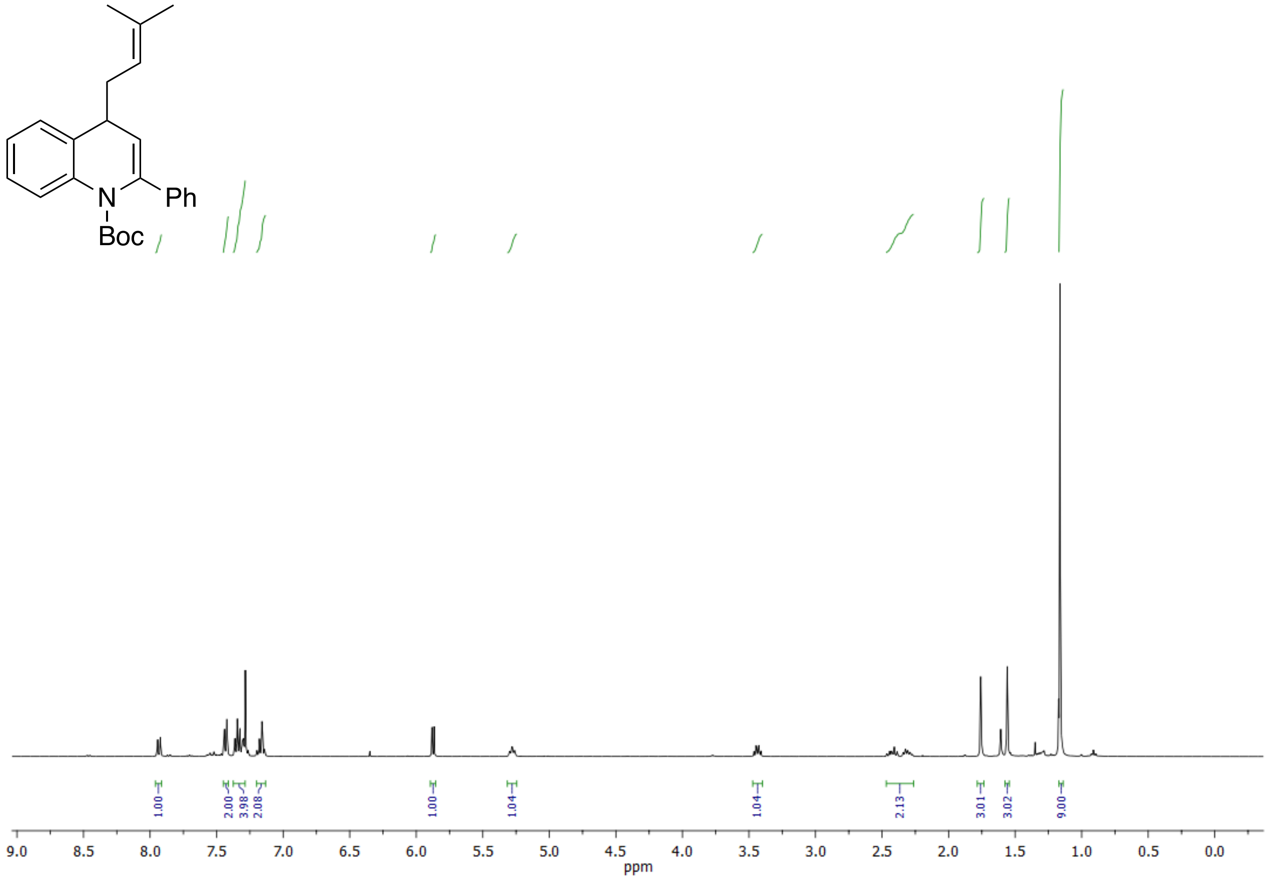

^{13}C{1H} NMR spectrum ($CDCl_3$, 100 MHz):

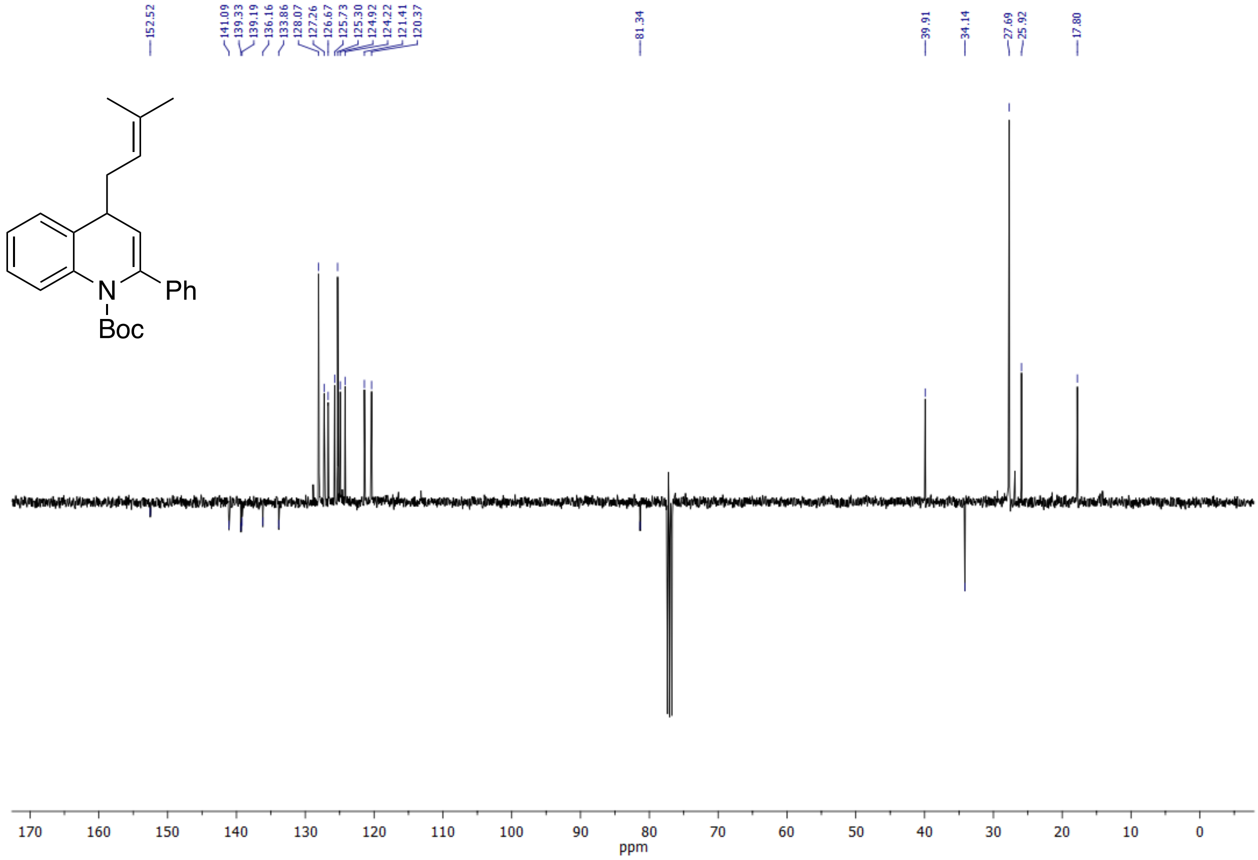

^{1}H NMR spectrum ($CDCl_3$, 400 MHz):

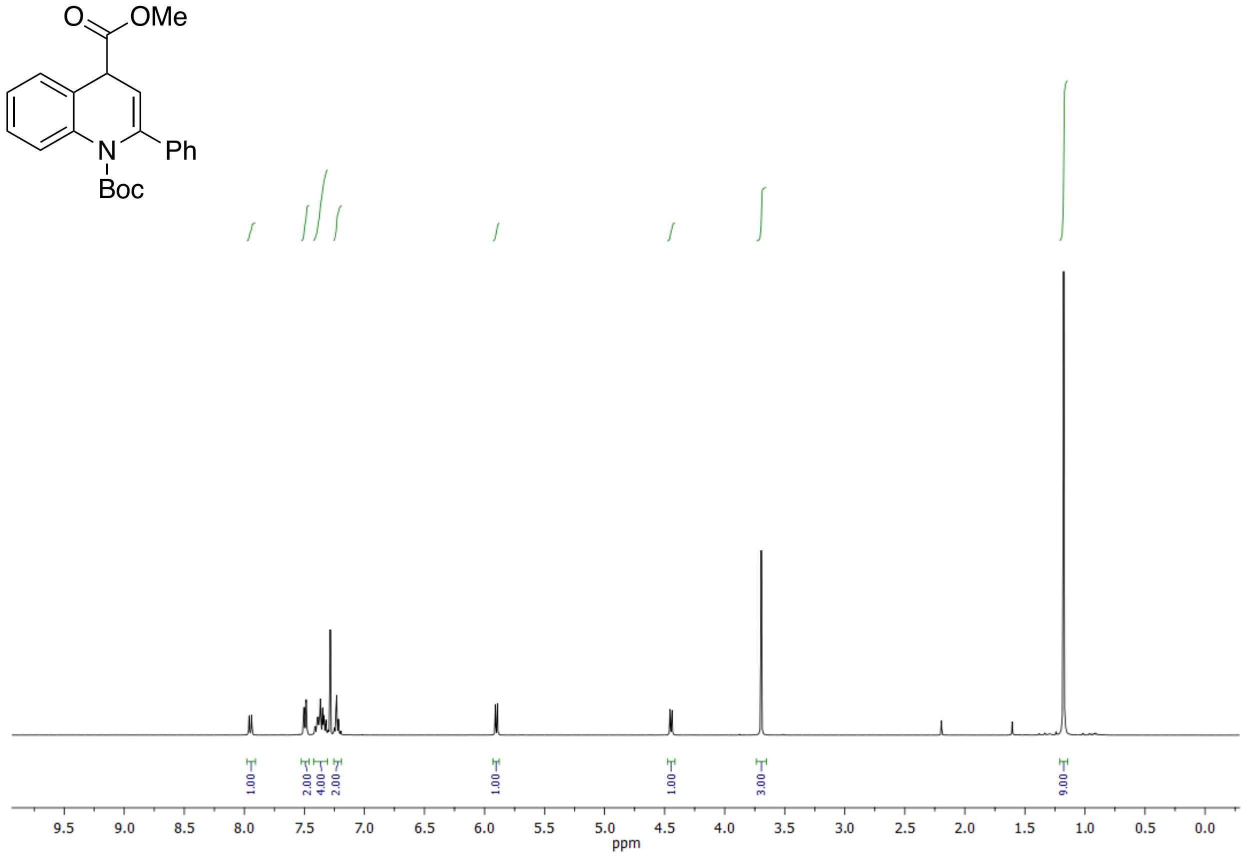

^{13}C{1H} NMR spectrum ($CDCl_3$, 100 MHz):

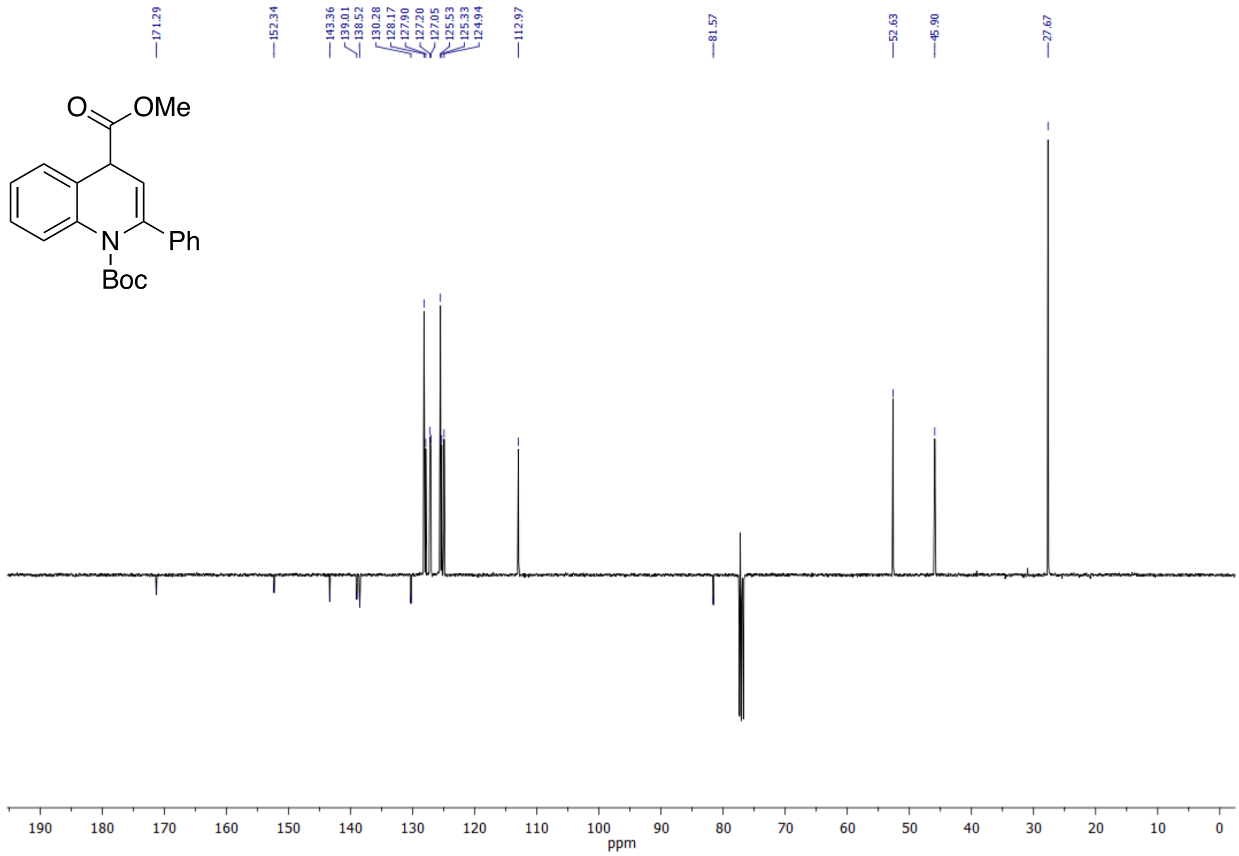

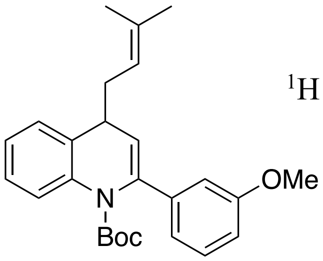

^{1}H NMR spectrum ($CDCl_3$, 400 MHz):

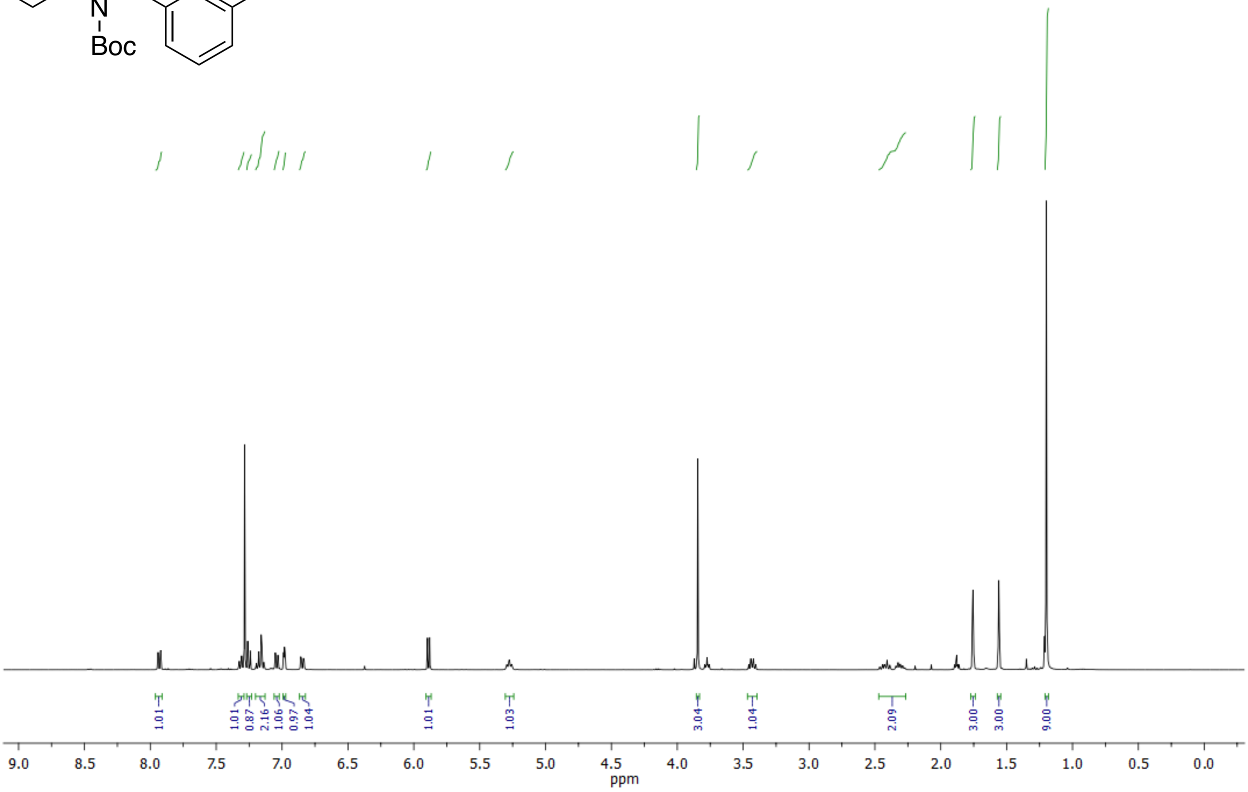

^{13}C{1H} NMR spectrum ($CDCl_3$, 100 MHz):

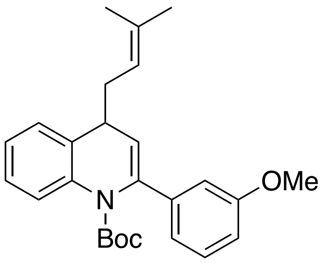

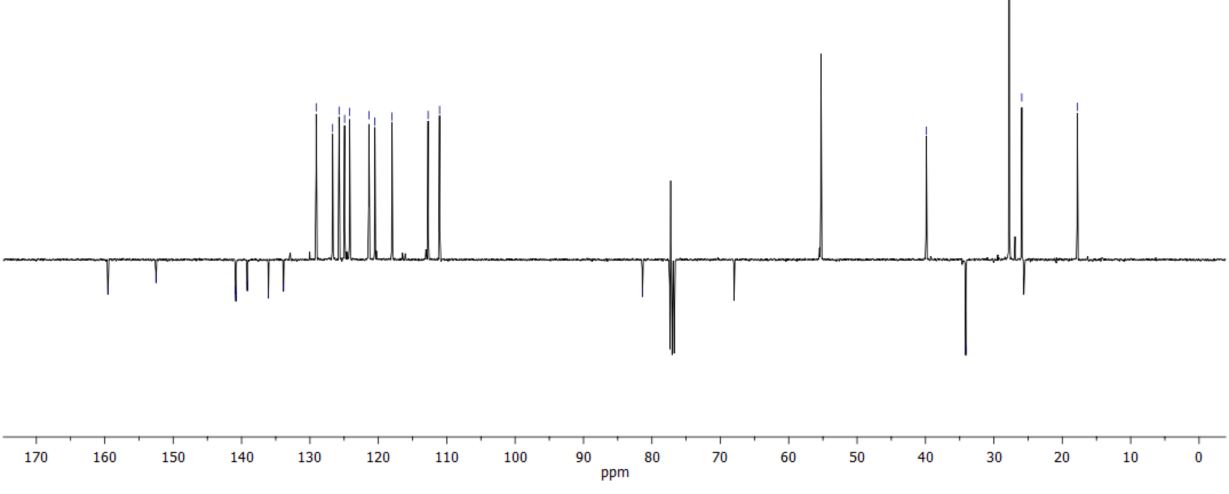

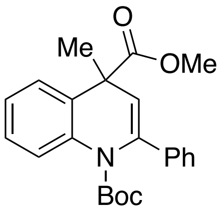

^{1}H NMR spectrum ($CDCl_3$, 400 MHz):

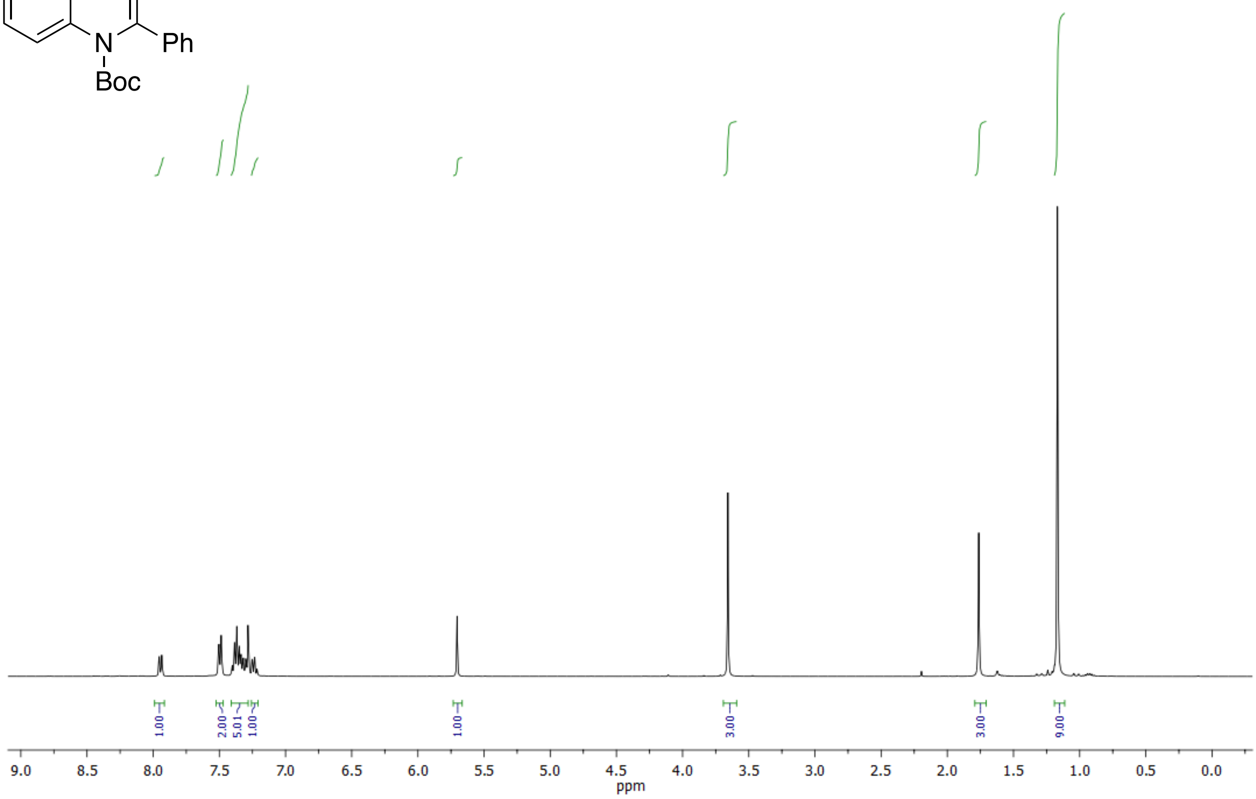

$^{13}C\{1H\}$ NMR spectrum ($CDCl_3$, 100 MHz):

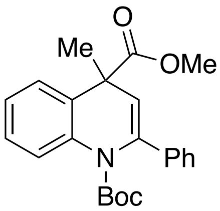

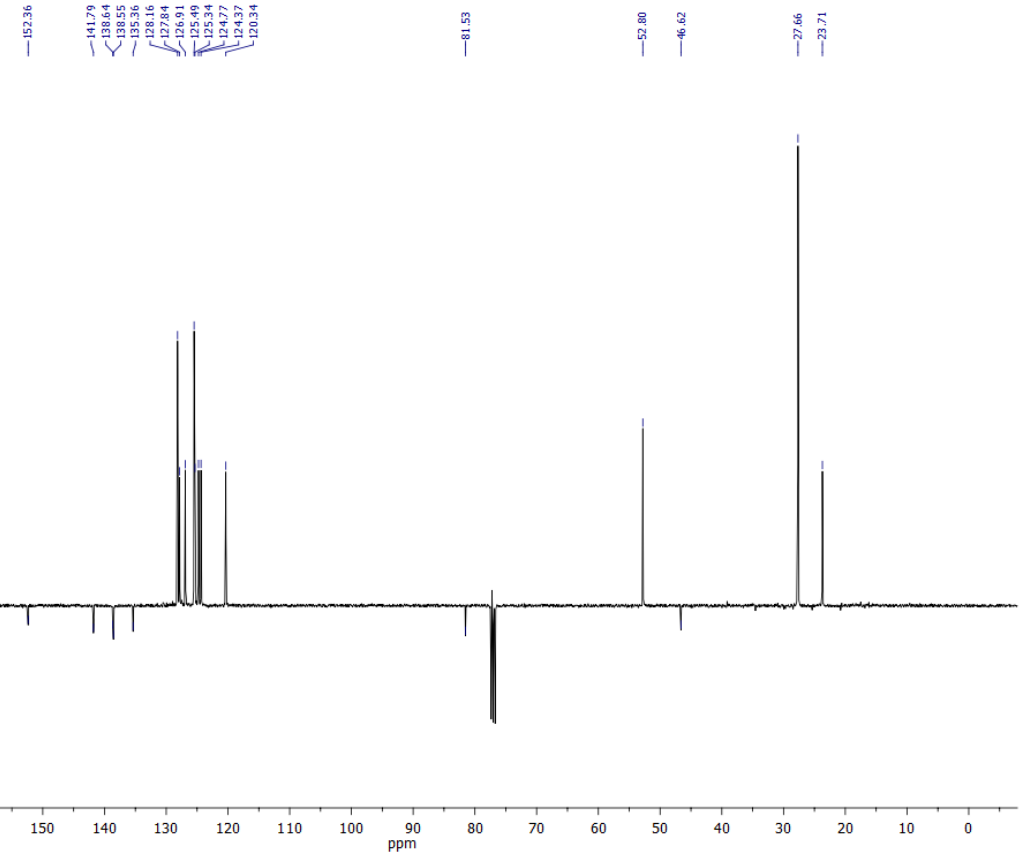

^{1}H NMR spectrum ($CDCl_3$, 400 MHz):

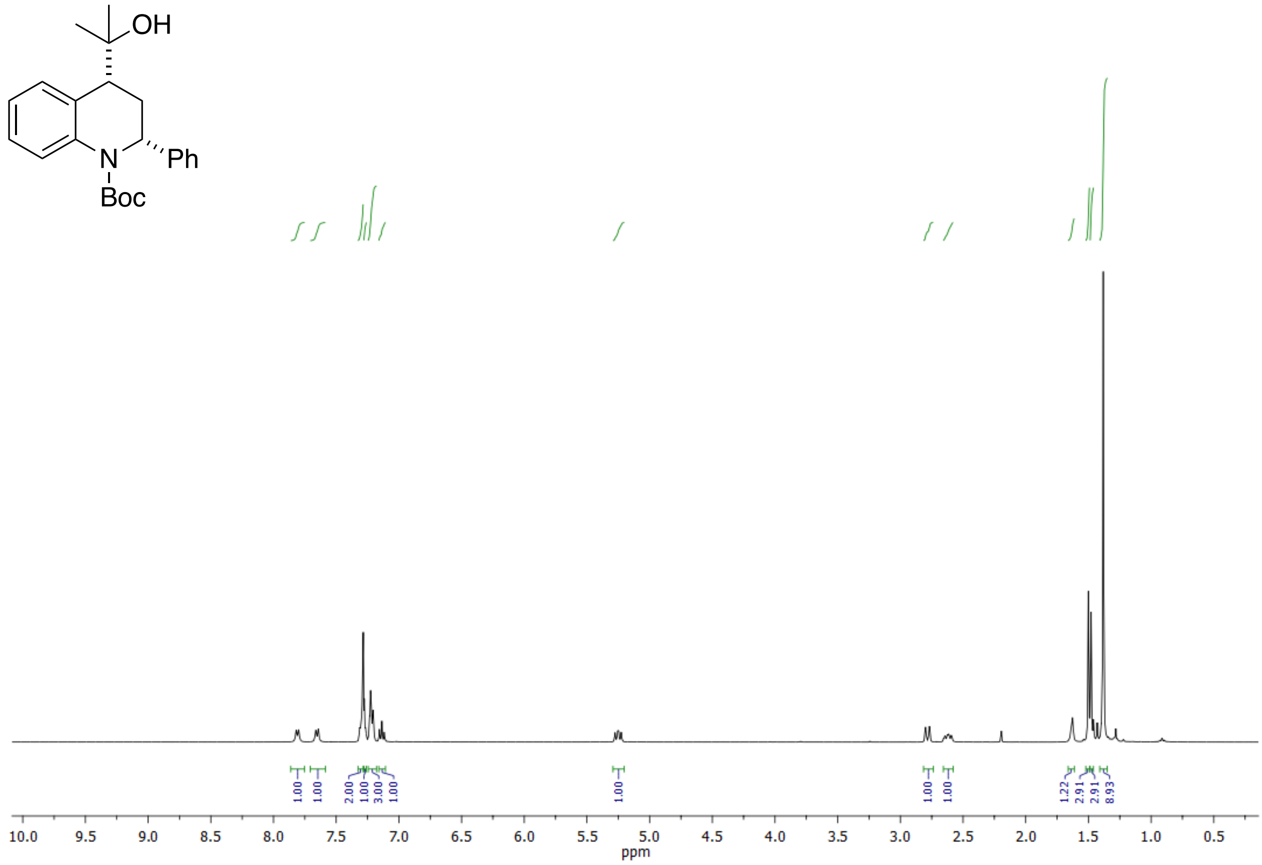

$^{13}C\{1H\}$ NMR spectrum ($CDCl_3$, 100 MHz):

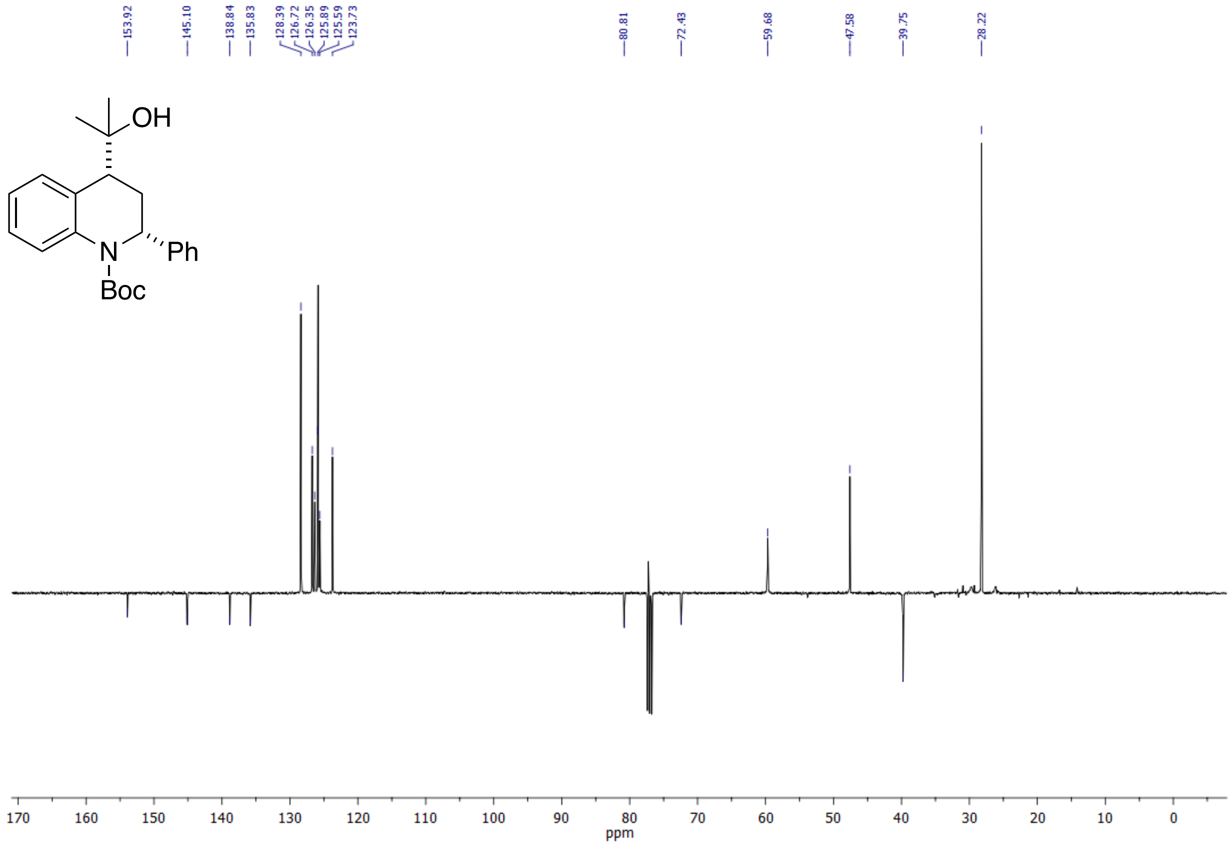

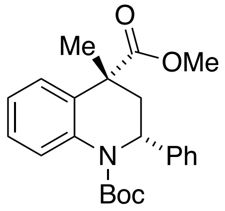

^{1}H NMR spectrum (C_6D_6, 400 MHz):

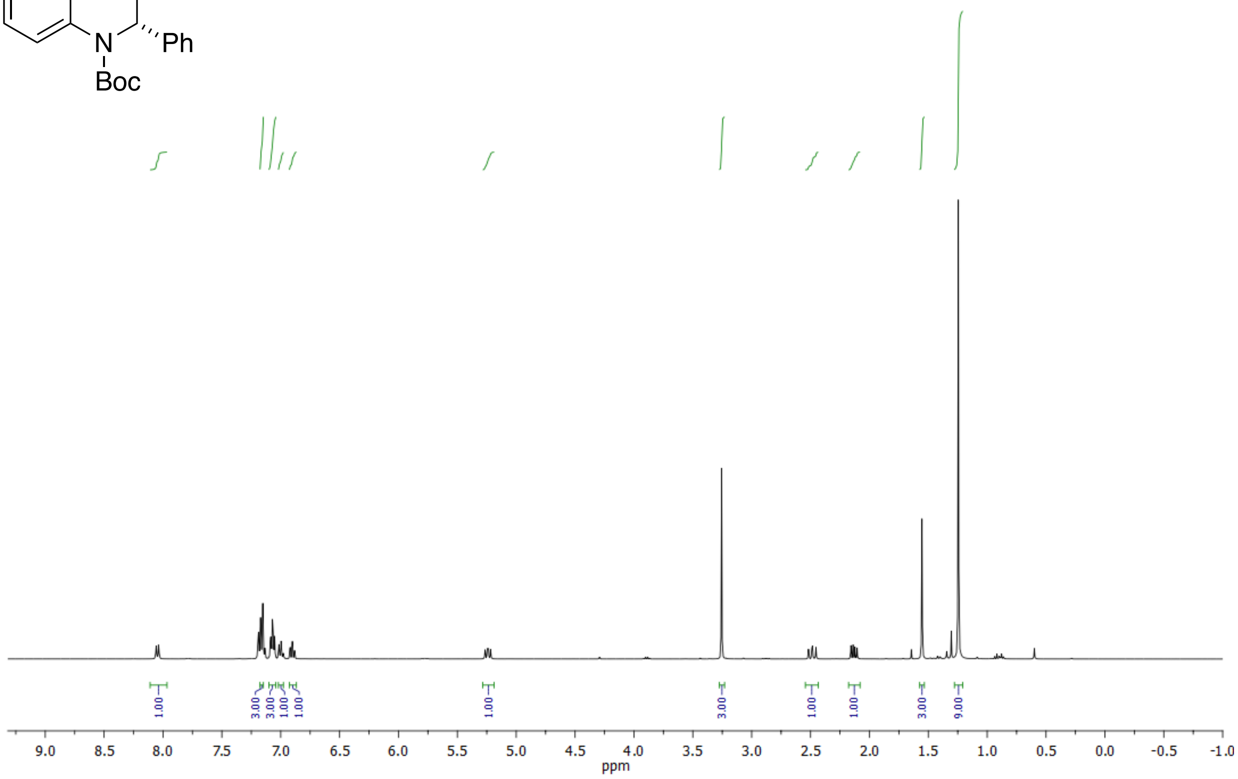

^{13}C{1H} NMR spectrum (C_6D_6, 100 MHz):

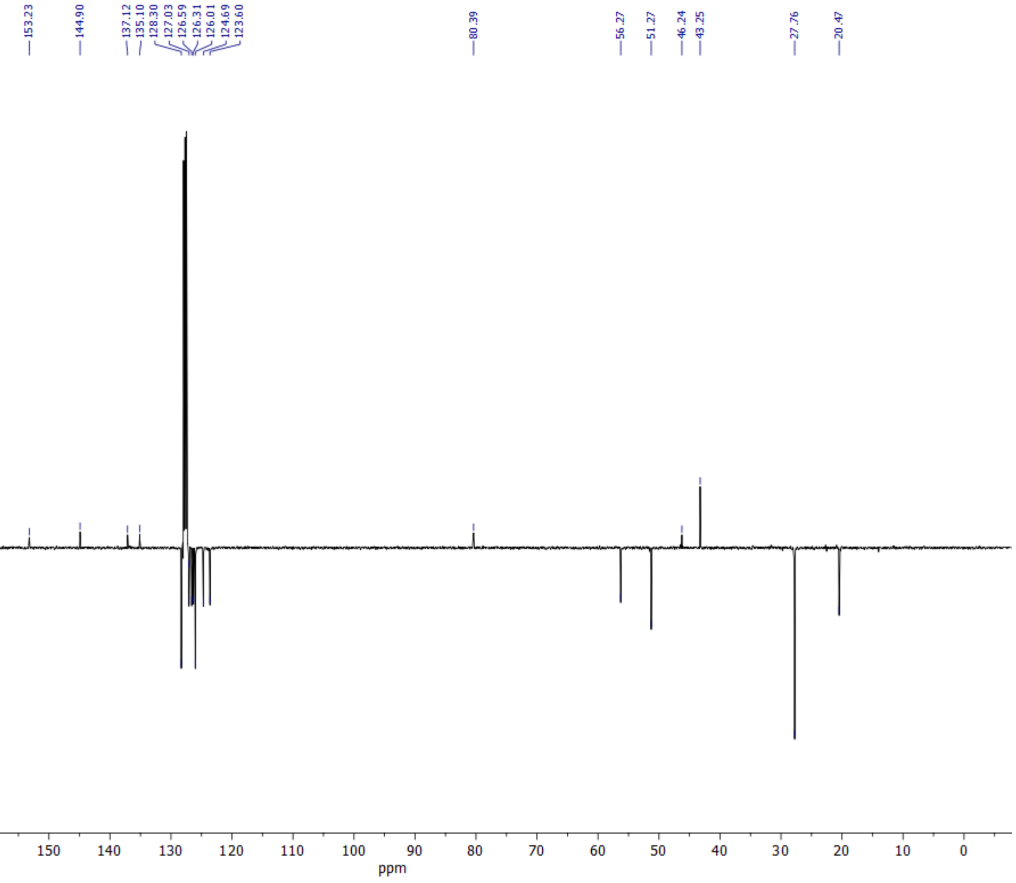

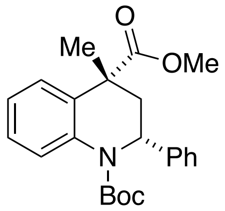

^{1}H NMR spectrum ($CDCl_3$, 400 MHz):

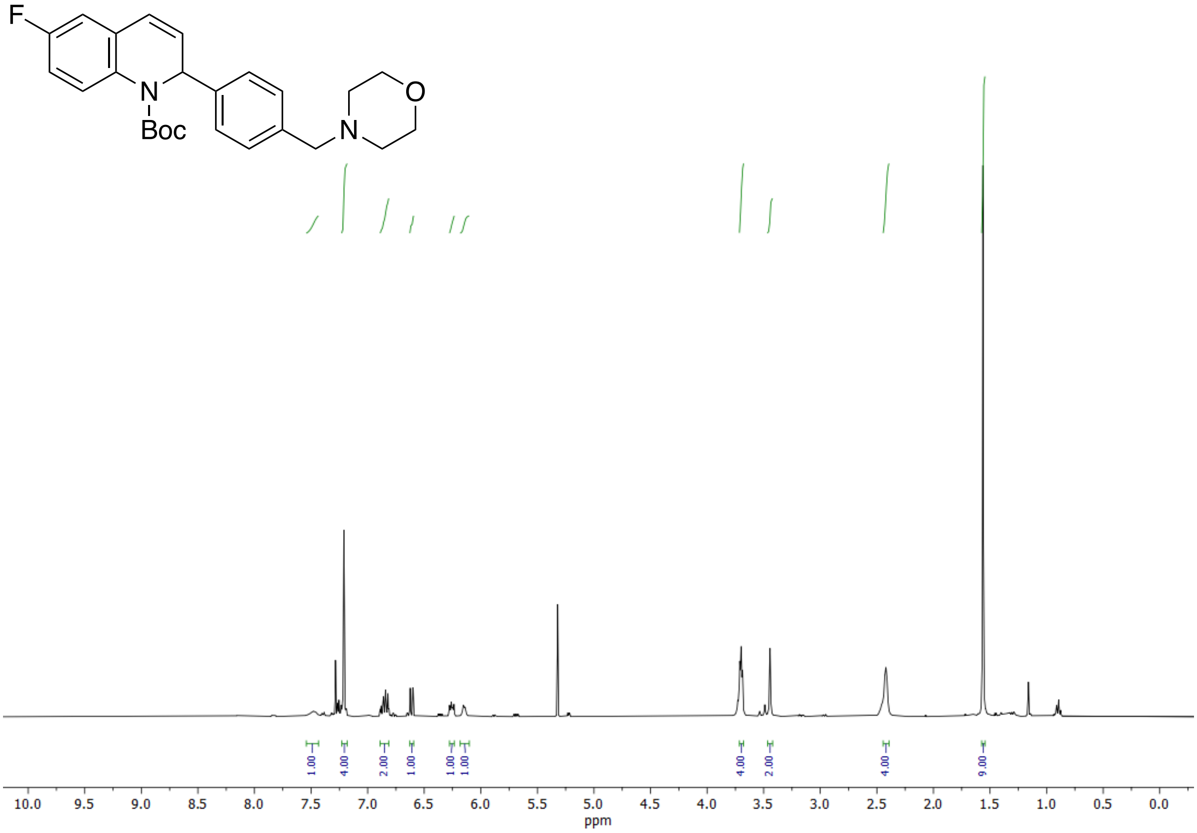

$^{13}C\{1H\}$ NMR spectrum ($CDCl_3$, 100 MHz):

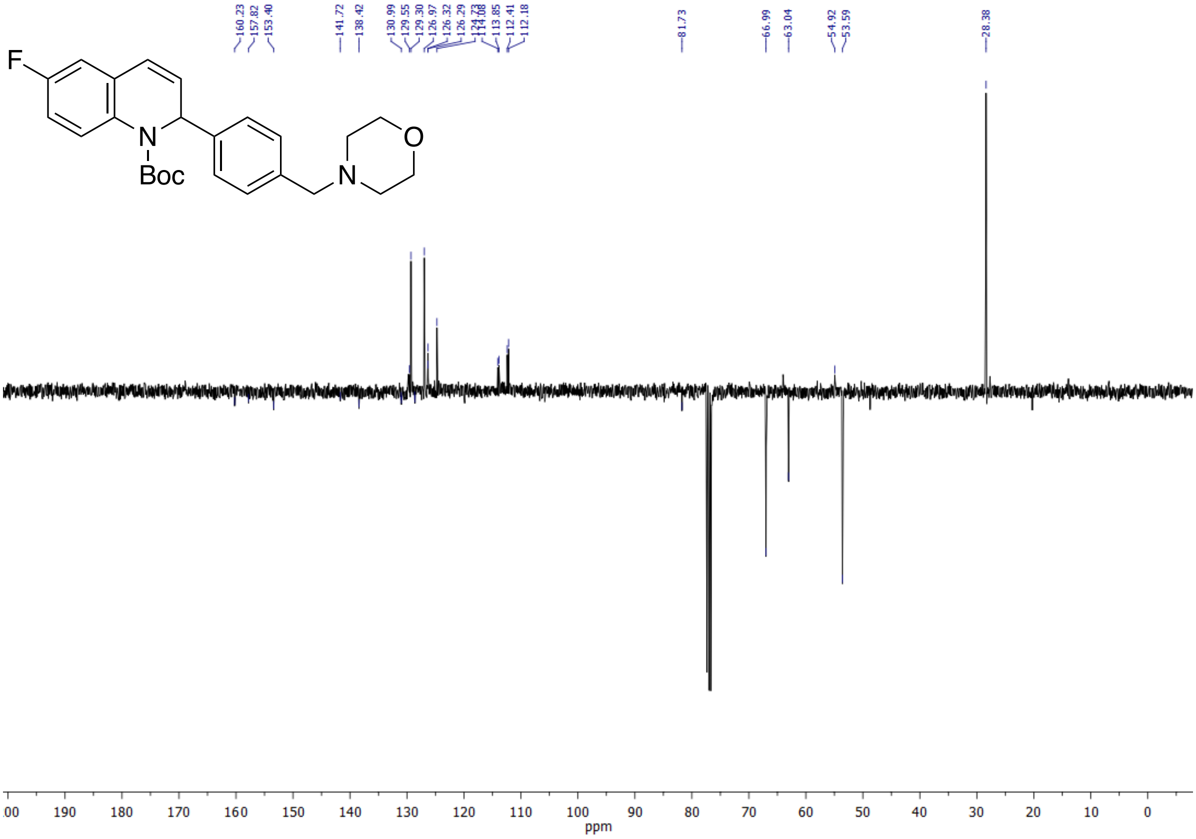

^{19}F NMR spectrum ($CDCl_3$, 377 MHz):

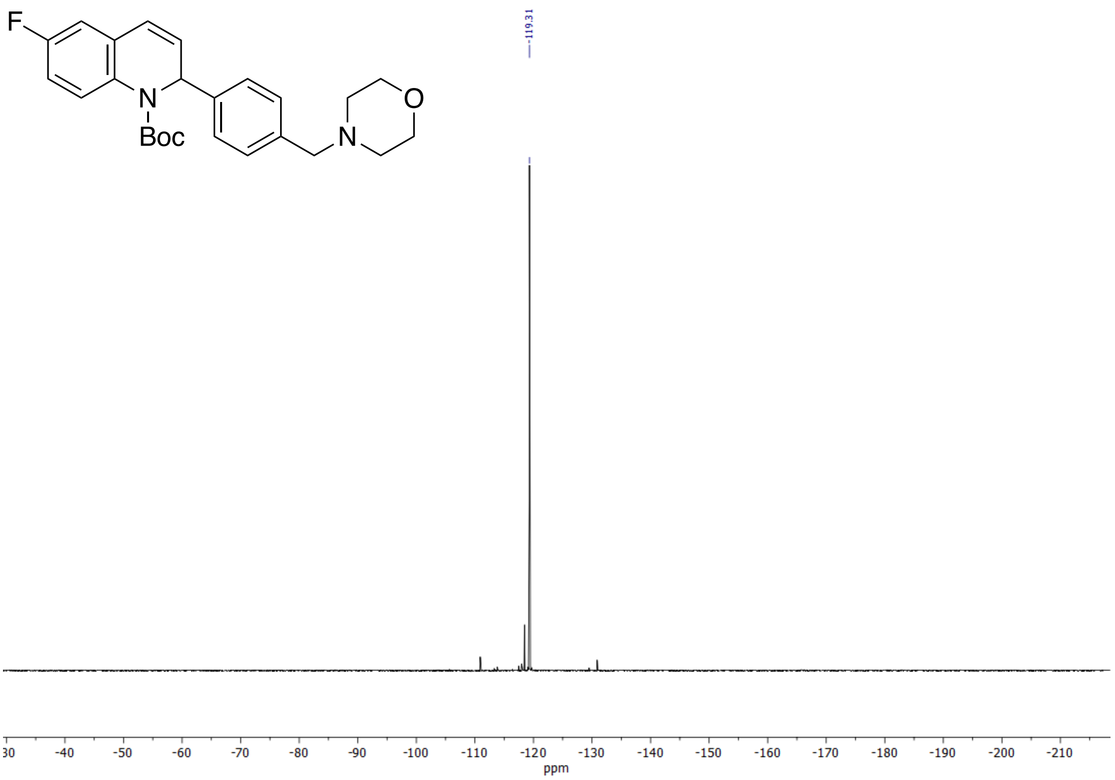

^{1}H NMR spectrum (DMSO-d_6, 400 MHz):

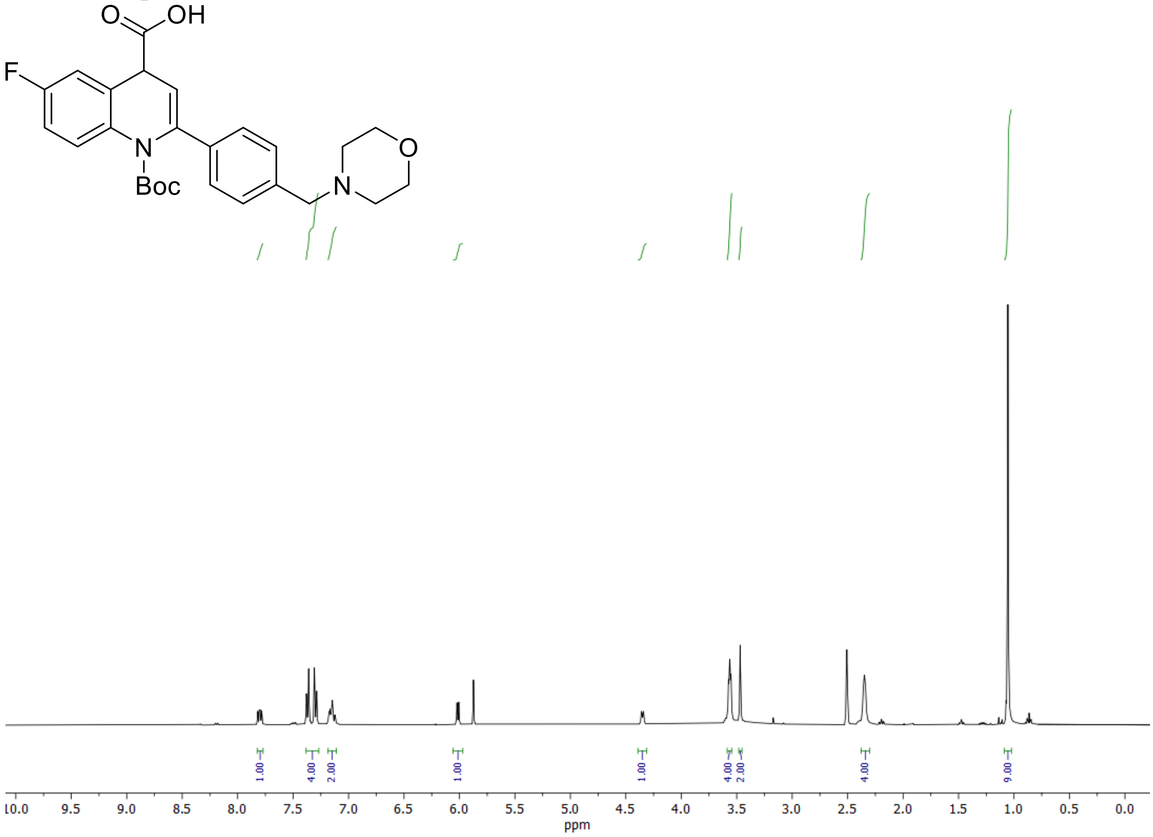

$^{13}C\{1H\}$ NMR spectrum (DMSO-d_6, 100 MHz):

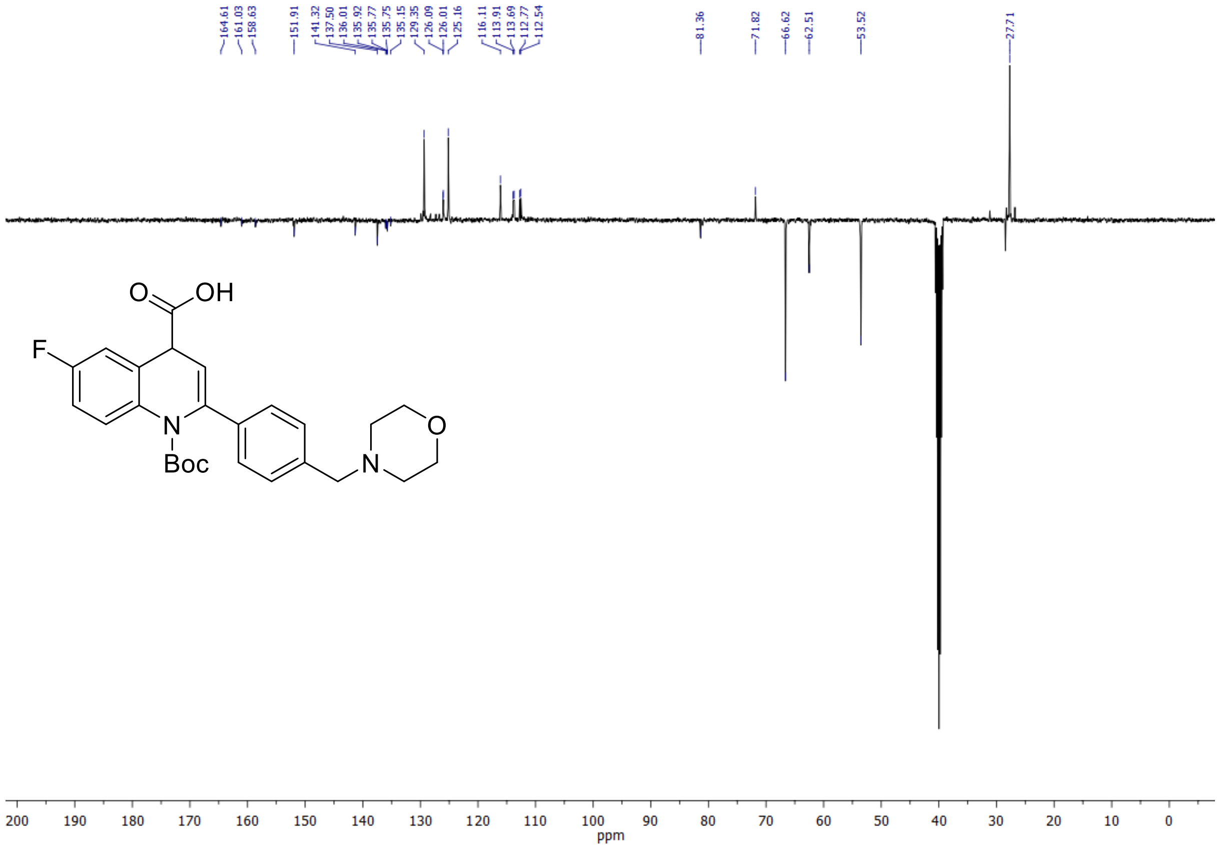

^{19}F NMR spectrum (DMSO-d_6, 377 MHz):

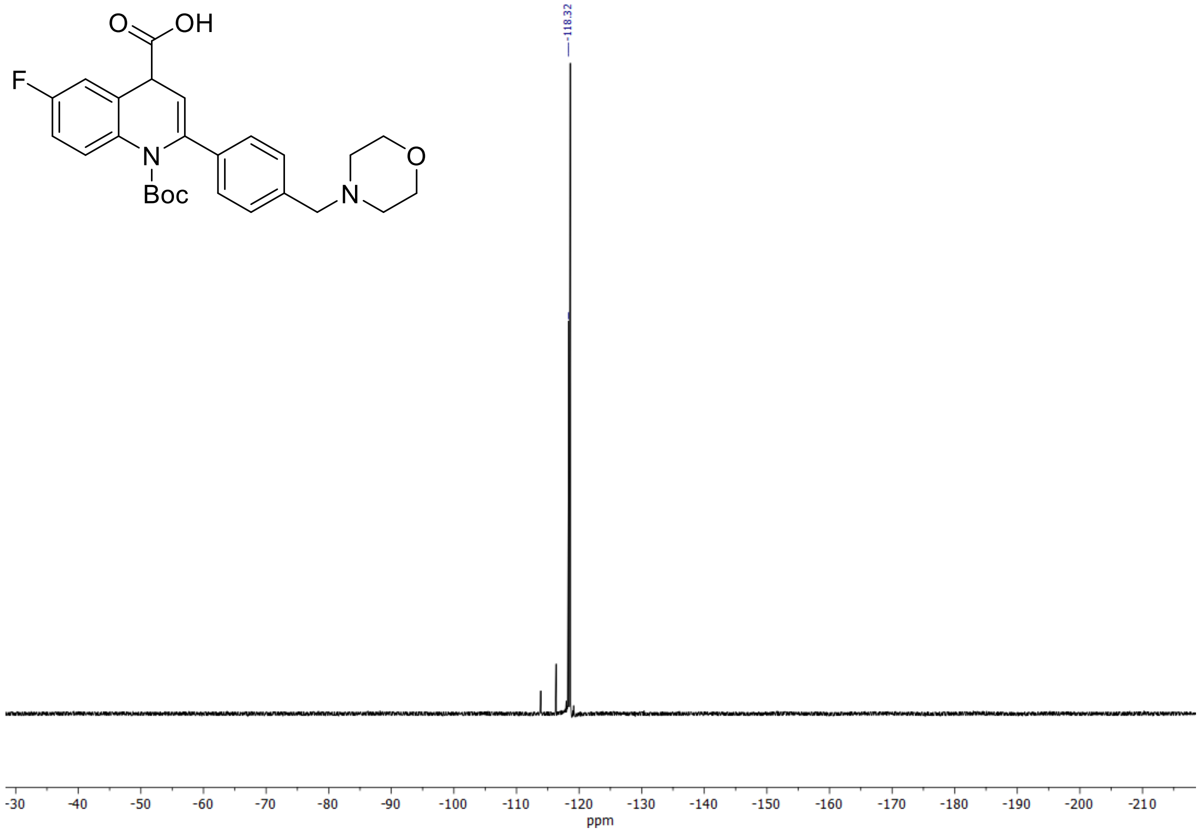

^{1}H NMR spectrum ($CDCl_3$, 400 MHz):

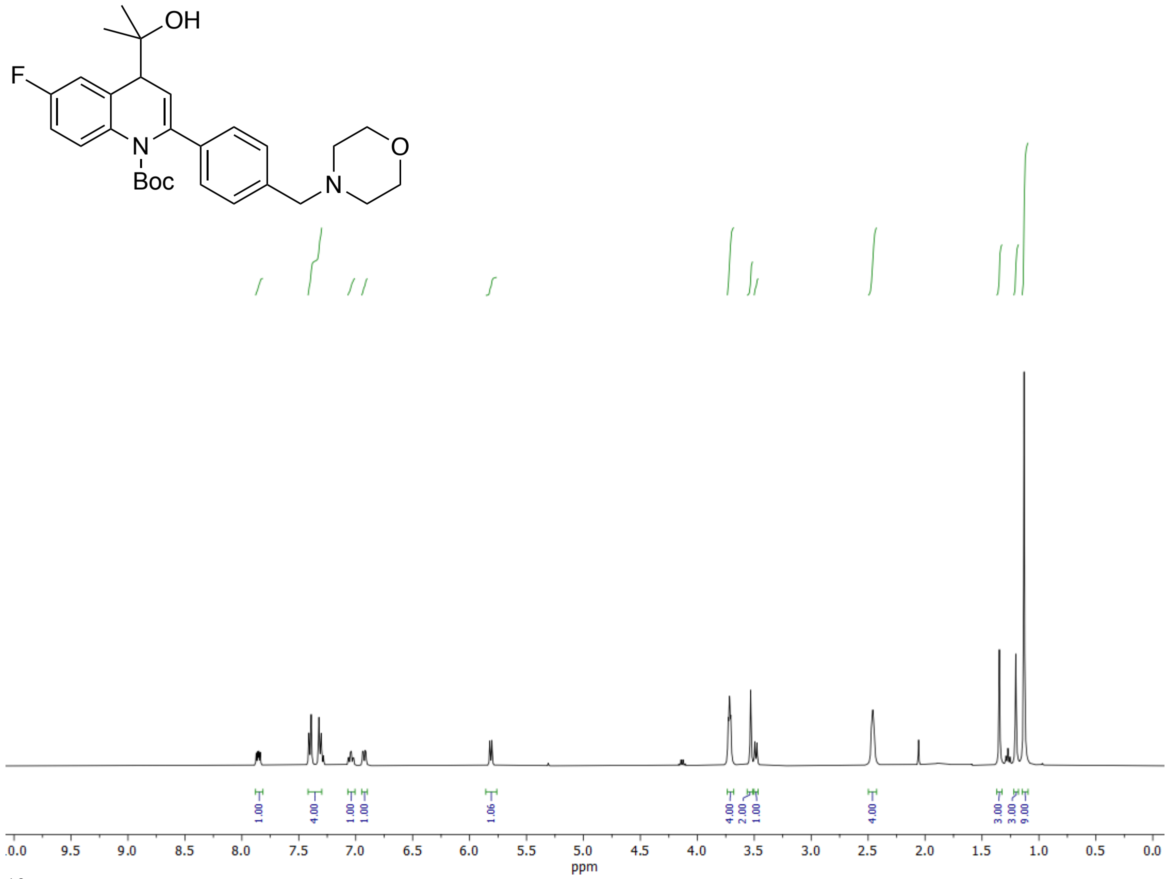

^{13}C{1H} NMR spectrum ($CDCl_3$, 100 MHz):

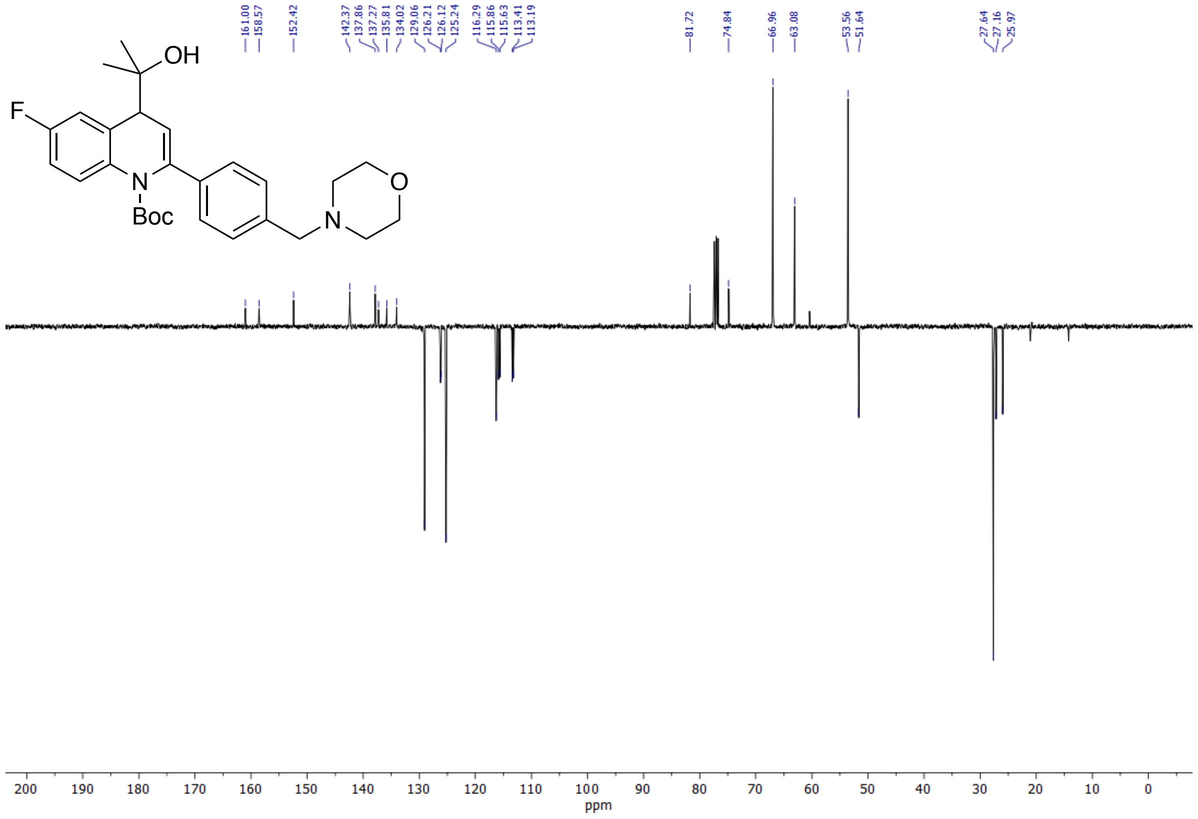

^{19}F NMR spectrum ($CDCl_3$, 377 MHz):

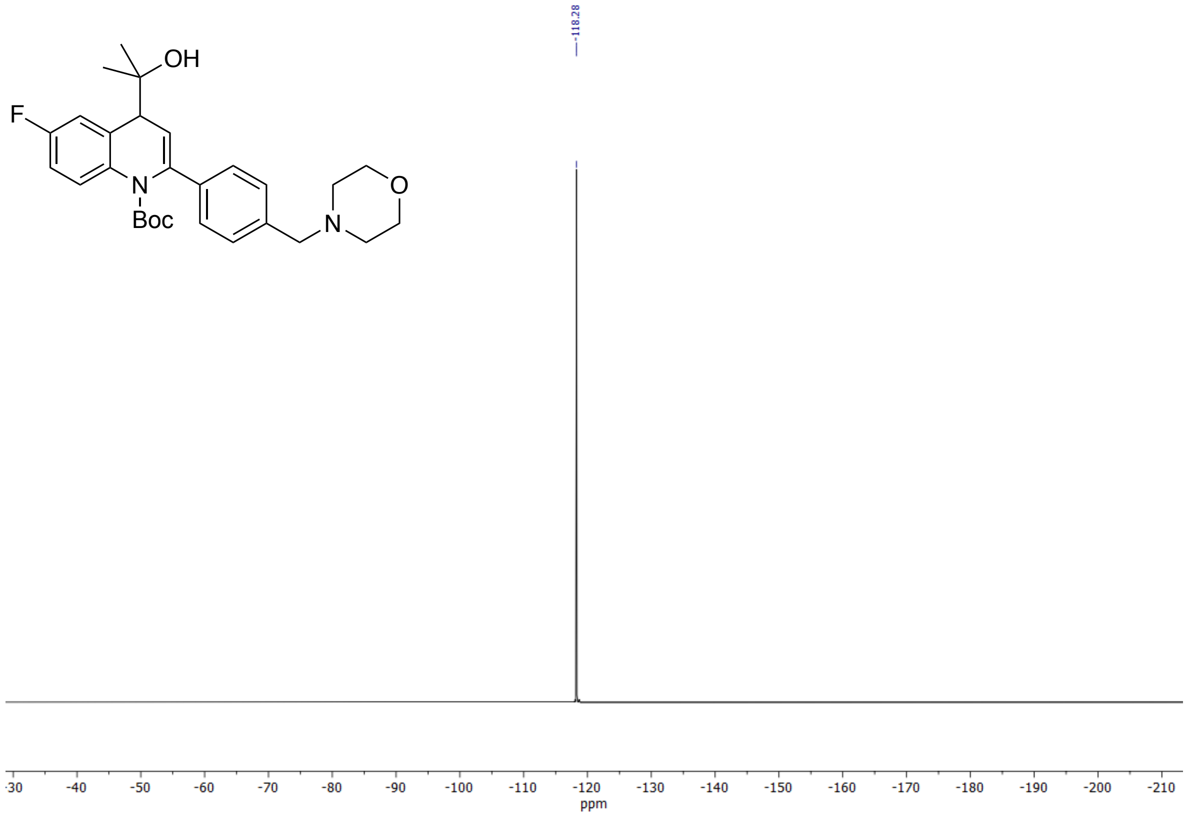

8. DFT data

8.1 Rotation calculations

DFT calculations were performed on the rotamers of **1a** (shown below as **1a-1** = 'left rotamer' and **1a-2** = 'right rotamer') were carried out using the B3LYP-D3BJ functional with the def2-TZVP basis set (B3LYP-D3BJ//def2-TZVP). Conformations of dihydroquinoline **1a** were considered where the phenyl group could be pseudo-axial or pseudo-equatorial. The minimal energy structures for the rotamers of dihydroquinoline **1a** had the phenyl group pseudo-axial as shown in structures a) and b) below. Of these structures the rotamer **1a–1**, in which the carbonyl group of the Boc group is pointing away from the C-2 position, was lower in Gibbs free energy by 1.2 kJ/mol at 195 K. This equates to a ratio of ~2:1 which matches that found by NMR spectroscopy.

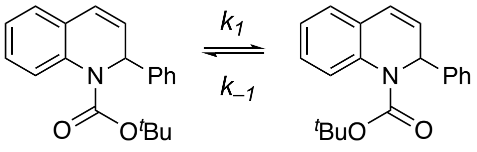

1a-1 **1a-2**

ratio 2 : 1
so $2k_1 = k_{-1}$

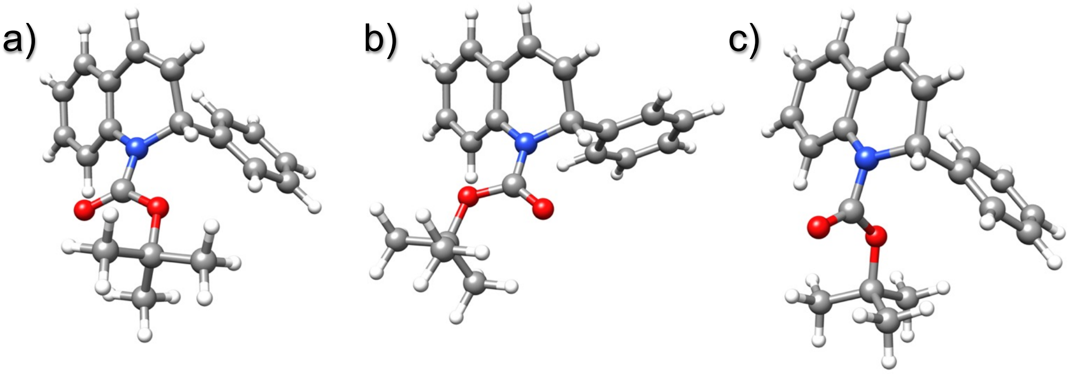

From structures a) and b), the transition state structures for rotation of the Boc group were investigated. The conformations of dihydroquinoline **1a** where the phenyl group could be pseudo-axial or pseudo-equatorial gave the same transition states for the clockwise rotation and anticlockwise rotation of the Boc group. The lowest energy transition state for rotation of the Boc group was obtained [structure c) above] where the Gibbs energy of activation was calculated to be ~ 43 kJ/mol at –78 °C with ΔH ≈ 38 kJ/mol and ΔS ≈ –25 J/K·mol.

Left Rotamer Axial

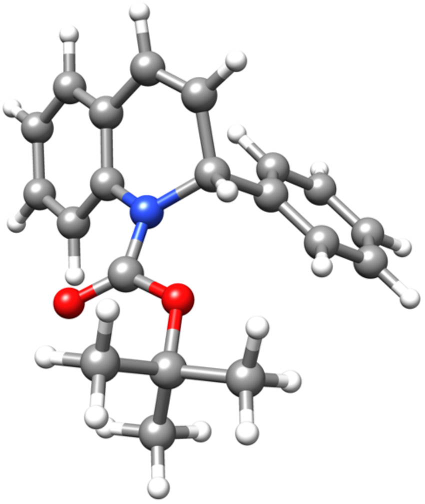

Route : # opt freq b3lyp scrf=(solvent=thf) geom=connectivity def2tzvp
empiricaldispersion=gd3bj int=ultrafine
pop=(regular,mk)

SMILES : CC(C)(C)OC(=O)N2c1ccccc1C=CC2c3ccccc3

Formula : $C_{20}H_{21}NO_2$

Charge : 0

Multiplicity : 1

Dipole : 3.3208 Debye

Energy : -980.468612027 a.u.

Gibbs Energy : -980.153580 a.u.

Number of imaginary frequencies : 0

Cartesian Coordinates (XYZ format)

44

C	1.24960600	-0.43895300	2.25185000
C	0.19446100	-0.57946400	1.19167900
H	-0.77511200	-0.57784600	1.67713000
H	1.10084100	-1.00525600	3.16184300
C	2.33050600	0.31695300	2.06483700
N	0.21082400	0.60775700	0.30203500
C	-0.96364100	1.20513800	-0.10745800
O	-1.01964400	2.25782600	-0.71149900
O	-2.02454400	0.46874600	0.25455700
C	-3.40563200	0.91524600	-0.00462300
C	-3.67858500	2.22296700	0.72839200
H	-3.09446600	3.03946400	0.31259400
H	-4.73768800	2.46848800	0.63860600
H	-3.44192300	2.11889100	1.78835400
C	-3.64526500	1.02244200	-1.50587800
H	-4.70650600	1.20017600	-1.68558900

H	-3.07431700	1.83776100	-1.94130500
H	-3.36747700	0.08974800	-1.99889200
C	-4.23231000	-0.21786000	0.58999600
H	-4.01586100	-0.33523500	1.65223500
H	-5.29387800	0.00079800	0.47442300
H	-4.01645000	-1.15825100	0.08221500
H	3.09710700	0.39355700	2.82619300
C	0.30984200	-1.87417800	0.40056400
C	1.53590500	-2.30771800	-0.10379900
C	-0.83147500	-2.63117100	0.14418500
C	1.61415600	-3.46750500	-0.86451000
H	2.43277900	-1.73940100	0.10159500
C	-0.75427100	-3.79824800	-0.60769200
H	-1.78520200	-2.29907800	0.53205000
C	0.46924900	-4.21668600	-1.11804700
H	2.57076500	-3.79080600	-1.25459300
H	-1.64853400	-4.37881700	-0.79509800
H	0.53225500	-5.12338000	-1.70580600
C	2.54074700	1.00898800	0.80374000
C	3.78989800	1.52008100	0.44722800
C	1.46837000	1.12090500	-0.10121100
C	3.99191300	2.11533700	-0.78857900
H	4.60685400	1.43484800	1.15324300
C	1.68016300	1.69751800	-1.35097300
C	2.93364300	2.19301000	-1.68805900
H	4.96526000	2.50704100	-1.05247500
H	0.86530200	1.77008700	-2.05238800
H	3.08030700	2.63952500	-2.66289300

Frequencies

Mode	IR frequency	IR intensity	Raman intensity
1	15.81590000	0.17580000	0.00000000
2	30.88860000	0.04350000	0.00000000
3	39.99600000	1.15550000	0.00000000
4	50.62310000	0.25150000	0.00000000
5	59.96980000	1.82690000	0.00000000
6	85.36100000	0.70230000	0.00000000
7	112.00730000	1.91980000	0.00000000
8	119.77810000	0.83520000	0.00000000
9	169.30140000	3.56980000	0.00000000
10	182.81810000	1.02280000	0.00000000
11	205.20450000	0.15920000	0.00000000
12	217.93060000	2.45740000	0.00000000
13	231.39730000	3.55970000	0.00000000
14	244.27300000	2.12600000	0.00000000
15	252.38630000	2.77260000	0.00000000
16	276.25470000	0.11560000	0.00000000
17	304.26320000	7.36510000	0.00000000

18	327.94660000	5.82010000	0.00000000
19	348.38510000	10.65890000	0.00000000
20	353.02990000	5.47760000	0.00000000
21	399.72050000	4.40060000	0.00000000
22	415.21150000	0.08560000	0.00000000
23	416.64700000	3.10730000	0.00000000
24	418.39090000	1.62530000	0.00000000
25	455.77190000	2.53990000	0.00000000
26	463.32540000	3.48170000	0.00000000
27	478.60640000	3.63280000	0.00000000
28	495.19490000	19.61090000	0.00000000
29	524.07390000	0.33580000	0.00000000
30	551.12420000	8.13010000	0.00000000
31	563.55880000	2.21720000	0.00000000
32	605.59330000	21.06200000	0.00000000
33	637.14830000	0.17940000	0.00000000
34	646.96510000	4.83110000	0.00000000
35	671.88750000	5.96110000	0.00000000
36	713.37600000	59.65040000	0.00000000
37	725.93120000	21.10630000	0.00000000
38	748.67090000	13.29820000	0.00000000
39	766.31450000	25.64630000	0.00000000
40	774.85850000	78.88250000	0.00000000
41	782.91110000	58.52920000	0.00000000
42	795.21760000	9.45690000	0.00000000
43	801.33930000	5.51260000	0.00000000
44	824.24000000	27.55060000	0.00000000
45	851.27500000	47.46950000	0.00000000
46	860.41750000	0.33500000	0.00000000
47	871.65990000	30.99260000	0.00000000
48	885.20940000	13.15350000	0.00000000
49	908.80950000	31.25150000	0.00000000
50	931.24360000	0.09810000	0.00000000
51	931.63570000	2.42320000	0.00000000
52	938.88650000	4.15890000	0.00000000
53	955.32960000	3.68610000	0.00000000
54	973.64860000	0.08190000	0.00000000
55	978.63540000	0.13440000	0.00000000
56	981.61830000	0.53460000	0.00000000
57	994.86870000	0.83300000	0.00000000
58	996.21030000	1.02960000	0.00000000
59	1013.46820000	47.72330000	0.00000000
60	1026.49660000	11.30010000	0.00000000

61	1043.24350000	89.66890000	0.00000000
62	1053.34450000	0.84840000	0.00000000
63	1055.22890000	8.01320000	0.00000000
64	1060.90720000	12.02500000	0.00000000
65	1070.68010000	13.21180000	0.00000000
66	1108.16360000	17.79900000	0.00000000
67	1137.33610000	58.03720000	0.00000000
68	1148.40110000	296.75720000	0.00000000
69	1181.35580000	2.96800000	0.00000000
70	1184.29500000	30.26550000	0.00000000
71	1187.76670000	442.57780000	0.00000000
72	1194.88640000	31.46250000	0.00000000
73	1206.10630000	6.05960000	0.00000000
74	1213.06440000	21.66790000	0.00000000
75	1238.21560000	14.33870000	0.00000000
76	1256.49750000	35.58810000	0.00000000
77	1270.68880000	22.50460000	0.00000000
78	1278.03020000	130.66410000	0.00000000
79	1285.05070000	2.55520000	0.00000000
80	1313.31090000	370.17580000	0.00000000
81	1330.43960000	363.65970000	0.00000000
82	1343.13280000	23.36770000	0.00000000
83	1350.14460000	50.11320000	0.00000000
84	1378.88590000	37.86530000	0.00000000
85	1398.42700000	26.18780000	0.00000000
86	1400.64930000	36.77840000	0.00000000
87	1411.34060000	99.24670000	0.00000000
88	1423.94000000	27.10240000	0.00000000
89	1425.73950000	41.40400000	0.00000000
90	1467.24280000	0.15290000	0.00000000
91	1484.45680000	1.88170000	0.00000000
92	1486.02350000	0.74120000	0.00000000
93	1487.26820000	21.46800000	0.00000000
94	1491.49460000	27.70420000	0.00000000
95	1494.19860000	1.33910000	0.00000000
96	1497.78620000	2.95510000	0.00000000
97	1517.14300000	24.06170000	0.00000000
98	1524.28110000	80.77510000	0.00000000
99	1532.05600000	13.45260000	0.00000000
100	1610.76380000	3.97770000	0.00000000
101	1628.08770000	0.17040000	0.00000000
102	1641.58620000	13.48590000	0.00000000
103	1644.27190000	2.47810000	0.00000000

104	1700.37700000	5.48670000	0.00000000
105	1720.12690000	573.20900000	0.00000000
106	3042.68760000	16.26800000	0.00000000
107	3045.05570000	35.88750000	0.00000000
108	3051.45260000	20.72180000	0.00000000
109	3104.41540000	6.88360000	0.00000000
110	3105.90010000	26.17040000	0.00000000
111	3112.51920000	44.16410000	0.00000000
112	3119.22180000	65.01870000	0.00000000
113	3137.53890000	3.31870000	0.00000000
114	3145.96470000	0.99810000	0.00000000
115	3148.97260000	24.89170000	0.00000000
116	3168.74080000	1.04280000	0.00000000
117	3169.90750000	0.34580000	0.00000000
118	3170.64030000	10.58010000	0.00000000
119	3178.23140000	9.22560000	0.00000000
120	3180.62540000	16.66750000	0.00000000
121	3188.88540000	25.73350000	0.00000000
122	3191.60440000	21.55800000	0.00000000
123	3196.12040000	37.30600000	0.00000000
124	3196.36220000	22.50680000	0.00000000
125	3200.58700000	7.61080000	0.00000000
126	3236.73070000	2.26170000	0.00000000

Right Rotamer Axial

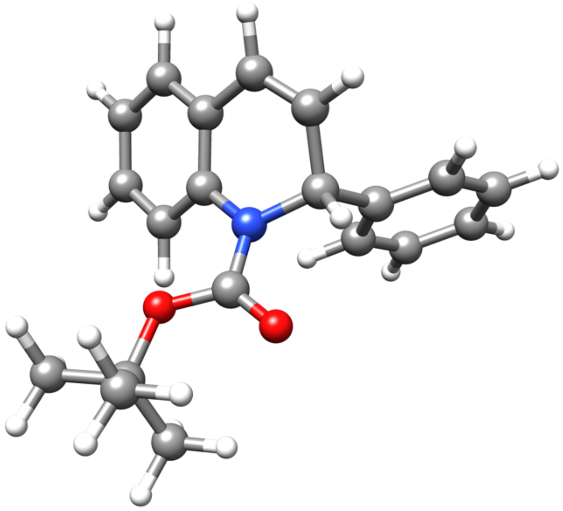

Route : # opt freq b3lyp scrf=(solvent=thf) geom=connectivity def2tzvp
empiricaldispersion=gd3bj int=ultrafine pop=(regular,mk)

SMILES : CC(C)(C)OC(=O)N2c1ccccc1C=CC2c3ccccc3

Formula : $C_{20}H_{21}NO_2$

Charge : 0

Multiplicity : 1

Dipole : 2.1556 Debye

Energy : -980.467667697 a.u.

Gibbs Energy : -980.153326 a.u.

Number of imaginary frequencies : 0

Cartesian Coordinates (XYZ format)

44

C	1.60657500	0.57921900	2.14601000
C	1.15125000	-0.48491800	1.17752100
H	0.86646200	-1.37285300	1.73785600
H	2.19702100	0.26134600	2.99423700
C	1.31927100	1.86291000	1.93604900
N	-0.07073700	-0.04467700	0.49465400
C	-1.12502600	-0.93724900	0.47705000
O	-1.00207900	-2.10239600	0.80623200
O	-2.26161800	-0.35633400	0.09081800
C	-3.49998600	-1.12923900	-0.12404700
C	-3.27186900	-2.18760100	-1.19631600
H	-2.58329300	-2.95653900	-0.85599800
H	-4.22502700	-2.65667200	-1.44366100
H	-2.87411400	-1.72835100	-2.10258600

C	-3.97810200	-1.72373100	1.19493400
H	-4.96560000	-2.16481500	1.05130700
H	-3.29835200	-2.49292100	1.55127500
H	-4.06096400	-0.94280800	1.95216400
C	-4.46786900	-0.06151700	-0.61704700
H	-4.10483200	0.38999000	-1.54057800
H	-5.44318200	-0.50899500	-0.80888900
H	-4.58569000	0.72302100	0.13071200
H	1.67089200	2.63211800	2.61264900
C	2.26991900	-0.88569800	0.22163600
C	2.14465600	-0.77014200	-1.15901500
C	3.45895400	-1.39329500	0.74898400
C	3.19086900	-1.14474500	-1.99780200
H	1.22846500	-0.39206100	-1.58782200
C	4.50134000	-1.77095800	-0.08581900
H	3.57004200	-1.50071100	1.82100000
C	4.37235200	-1.64387800	-1.46605500
H	3.07686400	-1.04636200	-3.06987500
H	5.41409300	-2.16747400	0.34021000
H	5.18459400	-1.93637700	-2.11870400
C	0.56365700	2.27046100	0.76283500
C	0.52585000	3.59935000	0.33398400
C	-0.12559200	1.29238500	0.02368800
C	-0.15860600	3.95898400	-0.81607600
H	1.05050700	4.34823000	0.91449100
C	-0.78836800	1.65241800	-1.14706000
C	-0.80817800	2.97769600	-1.55947100
H	-0.17795500	4.99154000	-1.13829100
H	-1.29202300	0.89935700	-1.73210400
H	-1.32976200	3.24120200	-2.47027000

Frequencies

Mode	IR frequency	IR intensity	Raman intensity
1	13.85610000	0.08790000	0.00000000
2	23.88460000	0.34840000	0.00000000
3	35.35450000	0.13800000	0.00000000
4	45.21180000	1.67430000	0.00000000
5	59.25610000	1.54950000	0.00000000
6	91.95180000	0.54840000	0.00000000
7	104.69510000	1.64040000	0.00000000
8	122.20010000	0.29460000	0.00000000
9	155.16640000	2.87570000	0.00000000
10	193.83020000	1.45420000	0.00000000
11	201.28370000	0.27440000	0.00000000
12	216.23860000	1.60780000	0.00000000
13	232.56700000	6.49830000	0.00000000
14	244.21700000	1.46570000	0.00000000
15	255.01030000	0.17210000	0.00000000

16	267.94750000	0.17370000	0.00000000
17	296.35590000	4.73410000	0.00000000
18	330.42760000	14.51040000	0.00000000
19	349.81520000	1.81060000	0.00000000
20	378.24320000	4.86460000	0.00000000
21	394.10300000	9.67870000	0.00000000
22	414.22720000	0.45630000	0.00000000
23	422.99430000	1.70760000	0.00000000
24	431.95500000	2.30970000	0.00000000
25	455.75010000	1.93800000	0.00000000
26	464.29020000	4.37830000	0.00000000
27	472.21080000	11.95380000	0.00000000
28	493.22440000	14.20370000	0.00000000
29	518.87400000	5.18540000	0.00000000
30	549.71770000	8.23510000	0.00000000
31	563.60280000	3.87180000	0.00000000
32	612.18640000	3.87950000	0.00000000
33	637.72040000	0.24740000	0.00000000
34	648.42040000	9.94910000	0.00000000
35	680.37610000	8.83330000	0.00000000
36	712.57980000	40.11220000	0.00000000
37	727.00360000	31.26670000	0.00000000
38	748.85170000	21.12420000	0.00000000
39	756.36080000	20.42380000	0.00000000
40	770.04870000	68.11810000	0.00000000
41	777.47690000	55.64690000	0.00000000
42	796.32160000	18.05800000	0.00000000
43	801.06760000	9.37910000	0.00000000
44	826.54780000	7.70100000	0.00000000
45	854.08790000	36.05130000	0.00000000
46	857.82670000	3.91220000	0.00000000
47	880.94240000	41.46930000	0.00000000
48	890.13670000	51.48780000	0.00000000
49	898.62250000	7.44360000	0.00000000
50	930.98850000	0.32670000	0.00000000
51	931.16760000	0.21180000	0.00000000
52	937.91450000	5.50770000	0.00000000
53	958.89920000	5.15070000	0.00000000
54	974.07990000	0.09490000	0.00000000
55	977.13580000	0.11060000	0.00000000
56	980.71640000	0.51290000	0.00000000
57	991.79900000	4.25350000	0.00000000
58	992.87770000	2.30980000	0.00000000

59	1000.33150000	41.10080000	0.00000000
60	1024.34530000	0.69680000	0.00000000
61	1043.44950000	70.92610000	0.00000000
62	1053.19970000	1.12400000	0.00000000
63	1055.29740000	9.23960000	0.00000000
64	1062.34250000	4.95810000	0.00000000
65	1073.37210000	30.51600000	0.00000000
66	1109.60240000	13.55330000	0.00000000
67	1145.78210000	7.18510000	0.00000000
68	1152.50610000	244.19420000	0.00000000
69	1181.24070000	0.10360000	0.00000000
70	1184.92140000	22.34270000	0.00000000
71	1187.51580000	381.46570000	0.00000000
72	1194.16930000	49.54300000	0.00000000
73	1208.11790000	3.03840000	0.00000000
74	1215.30970000	10.64130000	0.00000000
75	1240.09660000	13.78000000	0.00000000
76	1270.63230000	23.99260000	0.00000000
77	1274.30580000	56.86070000	0.00000000
78	1276.41520000	168.27370000	0.00000000
79	1296.28850000	179.36090000	0.00000000
80	1305.39630000	107.88940000	0.00000000
81	1331.64370000	23.65470000	0.00000000
82	1338.73940000	67.99160000	0.00000000
83	1360.24780000	375.64840000	0.00000000
84	1364.29430000	85.17900000	0.00000000
85	1398.61350000	27.70300000	0.00000000
86	1401.89280000	41.57990000	0.00000000
87	1416.84090000	58.68030000	0.00000000
88	1423.15440000	36.21990000	0.00000000
89	1425.37110000	12.98030000	0.00000000
90	1467.24630000	0.26520000	0.00000000
91	1483.10080000	0.40040000	0.00000000
92	1484.34410000	2.62610000	0.00000000
93	1485.33680000	8.12820000	0.00000000
94	1488.44790000	29.50760000	0.00000000
95	1494.58620000	23.40300000	0.00000000
96	1496.71570000	3.23090000	0.00000000
97	1516.01720000	25.10140000	0.00000000
98	1524.02660000	124.38790000	0.00000000
99	1533.12120000	19.08710000	0.00000000
100	1608.69390000	28.20640000	0.00000000
101	1626.82800000	4.86810000	0.00000000

102	1644.71970000	29.21610000	0.00000000
103	1645.65360000	8.77960000	0.00000000
104	1696.21140000	35.86050000	0.00000000
105	1712.21500000	647.93460000	0.00000000
106	3043.30830000	14.56770000	0.00000000
107	3044.54300000	35.68310000	0.00000000
108	3051.63090000	18.82490000	0.00000000
109	3099.46740000	8.52140000	0.00000000
110	3104.03960000	7.95430000	0.00000000
111	3105.88520000	27.13090000	0.00000000
112	3114.14240000	42.66190000	0.00000000
113	3119.35740000	61.47220000	0.00000000
114	3143.49410000	4.15650000	0.00000000
115	3147.61770000	21.71740000	0.00000000
116	3166.68090000	5.54510000	0.00000000
117	3169.59720000	0.35270000	0.00000000
118	3171.81140000	12.83970000	0.00000000
119	3173.89450000	3.06840000	0.00000000
120	3181.22740000	16.17470000	0.00000000
121	3182.92850000	28.55280000	0.00000000
122	3194.68620000	29.23180000	0.00000000
123	3196.77860000	32.30160000	0.00000000
124	3198.97730000	17.50550000	0.00000000
125	3213.85030000	7.06950000	0.00000000
126	3228.00220000	4.19500000	0.00000000

Left Rotamer Equatorial

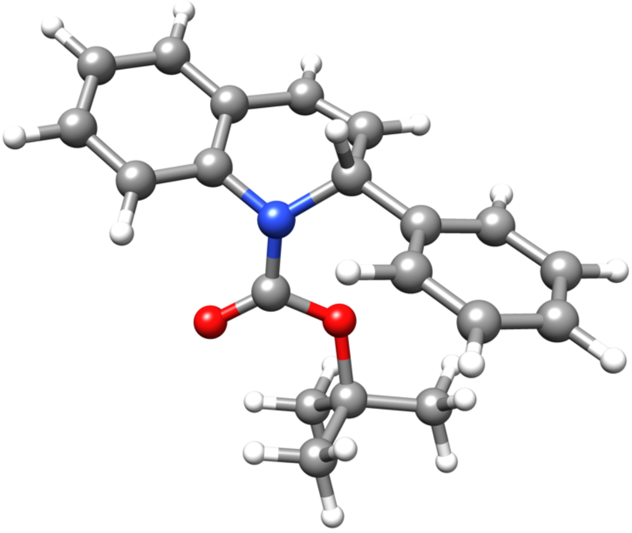

Route : # opt freq b3lyp scrf=(solvent=thf) geom=connectivity def2tzvp
empiricaldispersion=gd3bj int=ultrafine
pop=(regular,mk)

SMILES : CC(C)(C)OC(=O)N2c1ccccc1C=CC2c3ccccc3

Formula : $C_{20}H_{21}NO_2$

Charge : 0

Multiplicity : 1

Dipole : 2.9221 Debye

Energy : -980.456213568 a.u.

Gibbs Energy : -980.140744 a.u.

Number of imaginary frequencies : 0

Cartesian Coordinates (XYZ format)

44

C	0.30639400	-1.38207900	-0.11847100
C	0.82411700	-1.64085200	-1.51160200
H	0.82301800	-2.10209900	0.53097200
H	0.20339200	-2.09538600	-2.26863200
C	2.10752900	-1.34840300	-1.74438000
N	0.84857100	-0.08278000	0.34875600
C	0.23116100	1.14604500	0.16619200
O	0.74052400	2.19575400	0.50522400
O	-0.96307300	1.01353200	-0.41033800
C	-1.93832500	2.12042700	-0.44455600
C	-2.22919100	2.59562600	0.97373500
H	-1.35993100	3.07212700	1.41982100
H	-3.04717400	3.31681600	0.94781500
H	-2.53510600	1.75353500	1.59584200
C	-1.42409800	3.23636700	-1.34529800

H	-2.20905200	3.98332600	-1.47370600
H	-0.54742400	3.71625300	-0.91894200
H	-1.16829800	2.83857700	-2.32845400
C	-3.16890700	1.45915100	-1.04990800
H	-3.51381800	0.64021500	-0.42062100
H	-3.96918000	2.19357700	-1.14493200
H	-2.94195500	1.06219800	-2.03963500
H	2.56958900	-1.55761100	-2.70120100
C	-1.16399200	-1.59367100	0.17079600
C	-2.06901400	-2.03490200	-0.78685700
C	-1.61298500	-1.40556800	1.47935300
C	-3.39932200	-2.27075500	-0.44999900
H	-1.75868000	-2.18382700	-1.81045400
C	-2.93927500	-1.62699800	1.81570300
H	-0.91516600	-1.06604900	2.23458100
C	-3.84176400	-2.06290300	0.84848600
H	-4.09039300	-2.60767500	-1.21195100
H	-3.27014600	-1.46485900	2.83354800
H	-4.87748000	-2.23909800	1.10810300
C	2.92427700	-0.74248400	-0.69802300
C	4.32132000	-0.75036600	-0.72967300
C	2.27722800	-0.09435700	0.37182800
C	5.05534100	-0.13560900	0.27376900
H	4.82614700	-1.24062100	-1.55300900
C	3.01050300	0.53013200	1.37011000
C	4.39937500	0.50459000	1.32219100
H	6.13675600	-0.15229400	0.24027500
H	2.49335500	1.03373800	2.17292900
H	4.96970000	0.98398400	2.10690200

Frequencies

Mode	IR frequency	IR intensity	Raman intensity
1	26.81800000	1.03590000	0.00000000
2	35.09700000	1.11530000	0.00000000
3	40.75900000	0.68260000	0.00000000
4	59.25380000	0.92130000	0.00000000
5	77.41220000	0.30020000	0.00000000
6	84.62910000	1.74560000	0.00000000
7	103.20580000	0.95030000	0.00000000
8	119.77060000	0.26610000	0.00000000
9	150.42880000	5.10600000	0.00000000
10	176.61860000	0.66860000	0.00000000
11	208.69600000	0.37930000	0.00000000
12	216.01120000	7.35010000	0.00000000
13	240.22950000	1.91820000	0.00000000
14	255.57820000	5.17200000	0.00000000
15	268.19290000	0.78930000	0.00000000
16	279.98690000	0.44050000	0.00000000

17	301.03180000	1.21690000	0.00000000
18	310.80060000	4.42170000	0.00000000
19	334.46920000	19.15560000	0.00000000
20	354.16120000	2.09960000	0.00000000
21	400.05720000	4.69640000	0.00000000
22	412.85010000	0.23030000	0.00000000
23	423.85140000	1.76010000	0.00000000
24	437.05610000	0.01270000	0.00000000
25	459.78760000	3.72460000	0.00000000
26	470.27170000	21.00860000	0.00000000
27	480.94060000	3.55590000	0.00000000
28	489.41650000	0.13040000	0.00000000
29	512.77880000	4.90250000	0.00000000
30	532.05060000	1.99850000	0.00000000
31	574.75580000	3.55690000	0.00000000
32	603.85620000	4.32690000	0.00000000
33	636.85190000	0.21080000	0.00000000
34	657.02170000	8.55780000	0.00000000
35	664.31680000	9.01060000	0.00000000
36	703.58560000	4.85810000	0.00000000
37	712.30580000	55.15690000	0.00000000
38	744.28320000	42.64380000	0.00000000
39	749.22990000	46.68590000	0.00000000
40	767.88220000	14.37240000	0.00000000
41	777.20800000	11.65080000	0.00000000
42	785.48920000	133.87470000	0.00000000
43	794.22380000	18.88940000	0.00000000
44	843.43950000	34.45850000	0.00000000
45	850.55270000	21.41530000	0.00000000
46	856.72610000	0.70050000	0.00000000
47	874.73920000	3.67730000	0.00000000
48	885.47370000	3.16330000	0.00000000
49	919.41720000	24.14410000	0.00000000
50	931.74740000	1.03190000	0.00000000
51	932.44270000	0.04660000	0.00000000
52	940.98650000	10.11070000	0.00000000
53	959.48930000	3.25160000	0.00000000
54	974.54710000	0.14790000	0.00000000
55	976.84810000	1.03400000	0.00000000
56	982.64190000	0.82540000	0.00000000
57	984.77630000	0.19100000	0.00000000
58	1002.26790000	0.15400000	0.00000000
59	1013.71690000	55.45410000	0.00000000

60	1024.22800000	2.45450000	0.00000000
61	1034.06770000	5.05340000	0.00000000
62	1054.76780000	1.34040000	0.00000000
63	1057.87900000	15.25070000	0.00000000
64	1059.47960000	1.19770000	0.00000000
65	1064.94350000	1.04550000	0.00000000
66	1107.55570000	9.06010000	0.00000000
67	1137.55480000	12.78590000	0.00000000
68	1154.49540000	133.80330000	0.00000000
69	1180.04540000	188.18290000	0.00000000
70	1181.84020000	0.64120000	0.00000000
71	1183.08130000	408.34450000	0.00000000
72	1193.35410000	30.57960000	0.00000000
73	1215.12470000	0.95280000	0.00000000
74	1235.06880000	0.68600000	0.00000000
75	1244.20550000	4.75390000	0.00000000
76	1264.03590000	64.45970000	0.00000000
77	1271.06230000	47.40550000	0.00000000
78	1273.65430000	235.45830000	0.00000000
79	1291.39920000	180.49380000	0.00000000
80	1312.42060000	3.97950000	0.00000000
81	1316.32220000	17.99590000	0.00000000
82	1337.91790000	16.08020000	0.00000000
83	1352.67020000	6.89590000	0.00000000
84	1367.17500000	2.41340000	0.00000000
85	1398.20110000	26.35450000	0.00000000
86	1401.88240000	29.15060000	0.00000000
87	1408.50040000	289.19630000	0.00000000
88	1425.58970000	22.40140000	0.00000000
89	1427.71210000	117.64320000	0.00000000
90	1467.48420000	0.32420000	0.00000000
91	1484.31570000	1.50840000	0.00000000
92	1485.77090000	2.85600000	0.00000000
93	1487.35960000	28.00090000	0.00000000
94	1488.88410000	6.45120000	0.00000000
95	1493.73100000	2.15530000	0.00000000
96	1498.06510000	2.60410000	0.00000000
97	1514.31090000	58.72360000	0.00000000
98	1517.19750000	26.76280000	0.00000000
99	1538.23600000	17.90210000	0.00000000
100	1602.62840000	5.61930000	0.00000000
101	1628.88180000	4.85160000	0.00000000
102	1644.59030000	11.39230000	0.00000000

103	1649.74320000	20.07700000	0.00000000
104	1672.07090000	21.60450000	0.00000000
105	1716.11920000	546.63400000	0.00000000
106	2963.86890000	16.77750000	0.00000000
107	3044.02720000	18.90630000	0.00000000
108	3045.39420000	25.68540000	0.00000000
109	3052.27630000	28.34030000	0.00000000
110	3103.09400000	14.44860000	0.00000000
111	3107.65180000	17.83580000	0.00000000
112	3115.22800000	62.96780000	0.00000000
113	3128.90660000	23.36020000	0.00000000
114	3141.34420000	8.41030000	0.00000000
115	3147.73710000	18.55900000	0.00000000
116	3168.13840000	3.30830000	0.00000000
117	3170.60430000	2.58820000	0.00000000
118	3174.71170000	1.83890000	0.00000000
119	3175.57150000	13.78020000	0.00000000
120	3181.85090000	16.57310000	0.00000000
121	3183.83960000	31.22950000	0.00000000
122	3194.65990000	34.25630000	0.00000000
123	3195.62860000	29.08350000	0.00000000
124	3205.77650000	2.24520000	0.00000000
125	3218.95870000	5.86940000	0.00000000
126	3221.84060000	17.67610000	0.00000000

Right Rotamer Equatorial

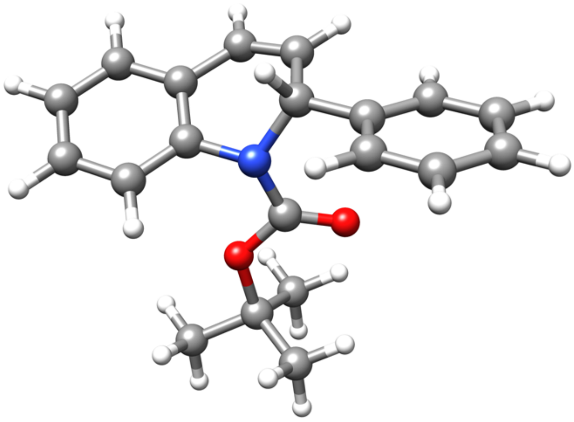

Route : # opt freq b3lyp scrf=(solvent=thf) geom=connectivity def2tzvp
empiricaldispersion=gd3bj int=ultrafine pop=(regular,mk)

SMILES : CC(C)(C)OC(=O)N2c1ccccc1C=CC2c3ccccc3

Formula : $C_{20}H_{21}NO_2$

Charge : 0

Multiplicity : 1

Dipole : 2.5199 Debye

Energy : -980.454619636 a.u.

Gibbs Energy : -980.140584 a.u.

Number of imaginary frequencies : 0

Cartesian Coordinates (XYZ format)

44

C	0.99422900	-1.41921300	0.05237100
C	0.65074600	-2.07903700	1.36596400
H	0.90353100	-2.20046400	-0.71348100
H	1.42005400	-2.31047100	2.08764900
C	-0.62131200	-2.43999200	1.55915400
N	-0.09666600	-0.47977600	-0.30241000
C	-0.05126800	0.83539300	0.13618500
O	0.92375700	1.35062300	0.63960500
O	-1.21663500	1.44946300	-0.10390400
C	-1.43892900	2.86176700	0.24747600
C	-1.30953200	3.05268300	1.75418300
H	-0.28556100	2.90367900	2.08591300
H	-1.61908100	4.06590000	2.01453900
H	-1.95975600	2.35181700	2.27974800
C	-0.48415700	3.75206000	-0.53915300
H	-0.75293800	4.79696000	-0.37731400

H	0.54529400	3.60245200	-0.22509900
H	-0.56478000	3.54118200	-1.60659300
C	-2.87752700	3.08571200	-0.20098400
H	-3.55224500	2.41254800	0.32833700
H	-3.17453400	4.11344200	0.00881100
H	-2.97711800	2.90671300	-1.27192600
H	-0.92836200	-2.97140900	2.45144100
C	2.38555900	-0.85261100	-0.12420600
C	3.28914400	-0.70732200	0.92252900
C	2.79260300	-0.51127600	-1.41387000
C	4.56982400	-0.21798400	0.68850800
H	2.99623600	-0.94778900	1.93436400
C	4.06621100	-0.01426200	-1.65081900
H	2.09765900	-0.62850400	-2.23670700
C	4.96282700	0.13352300	-0.59670800
H	5.25852600	-0.10575000	1.51614500
H	4.36234200	0.25093000	-2.65773100
H	5.95891100	0.51638300	-0.77738700
C	-1.62749600	-2.15143400	0.54213000
C	-2.85505400	-2.81681100	0.48442900
C	-1.35177700	-1.15889000	-0.41640600
C	-3.77846400	-2.51388400	-0.50508600
H	-3.07725200	-3.57567000	1.22460600
C	-2.27403200	-0.85775200	-1.40785700
C	-3.48545400	-1.53627600	-1.45297500
H	-4.72377800	-3.03910400	-0.54184100
H	-2.04367000	-0.08896700	-2.13077000
H	-4.20063600	-1.30249900	-2.23062400

Frequencies

Mode	IR frequency	IR intensity	Raman intensity
1	22.18490000	0.14340000	0.00000000
2	29.86450000	0.99540000	0.00000000
3	33.27730000	0.31040000	0.00000000
4	52.27110000	0.92780000	0.00000000
5	63.53310000	0.24410000	0.00000000
6	72.38880000	1.27790000	0.00000000
7	97.47200000	0.45580000	0.00000000
8	107.54230000	0.81780000	0.00000000
9	150.14240000	4.23620000	0.00000000
10	174.30570000	3.42140000	0.00000000
11	206.69520000	0.14580000	0.00000000
12	219.85540000	2.25020000	0.00000000
13	236.47200000	11.12710000	0.00000000
14	246.75850000	0.01320000	0.00000000
15	262.52200000	2.01650000	0.00000000
16	272.71430000	0.05130000	0.00000000
17	304.17050000	1.36520000	0.00000000

18	333.09100000	19.54180000	0.00000000
19	341.97280000	4.59570000	0.00000000
20	351.10350000	1.79560000	0.00000000
21	394.89700000	8.39230000	0.00000000
22	414.00860000	0.32060000	0.00000000
23	421.02170000	0.76040000	0.00000000
24	430.51180000	2.23260000	0.00000000
25	459.06990000	2.87720000	0.00000000
26	471.04010000	3.53850000	0.00000000
27	479.74820000	21.38460000	0.00000000
28	486.81330000	3.13120000	0.00000000
29	516.38730000	6.96320000	0.00000000
30	532.81830000	0.80640000	0.00000000
31	574.46090000	3.51380000	0.00000000
32	602.30000000	12.07170000	0.00000000
33	637.26420000	0.07210000	0.00000000
34	646.24600000	9.32940000	0.00000000
35	682.81740000	9.46410000	0.00000000
36	701.30620000	3.81040000	0.00000000
37	709.80520000	53.54280000	0.00000000
38	734.53030000	13.48430000	0.00000000
39	757.73570000	53.97860000	0.00000000
40	765.22760000	40.99770000	0.00000000
41	784.14210000	66.24090000	0.00000000
42	790.37990000	54.60410000	0.00000000
43	794.82880000	15.15590000	0.00000000
44	844.88080000	9.35910000	0.00000000
45	849.48390000	10.64420000	0.00000000
46	854.51330000	0.35720000	0.00000000
47	885.51710000	3.87920000	0.00000000
48	891.04210000	53.17080000	0.00000000
49	916.19750000	42.55440000	0.00000000
50	930.95990000	0.14550000	0.00000000
51	931.11340000	1.40330000	0.00000000
52	939.22870000	2.21500000	0.00000000
53	961.05450000	3.02480000	0.00000000
54	972.66590000	0.93920000	0.00000000
55	973.65140000	0.08960000	0.00000000
56	981.66740000	0.91270000	0.00000000
57	986.11660000	0.45120000	0.00000000
58	989.38930000	0.05000000	0.00000000
59	1014.63560000	19.41310000	0.00000000
60	1023.47720000	13.54320000	0.00000000

61	1031.28120000	8.36620000	0.00000000
62	1054.45470000	1.16060000	0.00000000
63	1057.04680000	14.81930000	0.00000000
64	1059.29860000	0.93580000	0.00000000
65	1064.09810000	14.23770000	0.00000000
66	1107.77450000	5.54270000	0.00000000
67	1136.77330000	30.75500000	0.00000000
68	1140.79540000	205.12320000	0.00000000
69	1178.33090000	307.49260000	0.00000000
70	1181.22230000	88.48960000	0.00000000
71	1181.33590000	61.50430000	0.00000000
72	1190.55330000	116.80460000	0.00000000
73	1210.27000000	0.53770000	0.00000000
74	1233.77620000	11.29150000	0.00000000
75	1242.07480000	6.59650000	0.00000000
76	1265.89880000	126.76170000	0.00000000
77	1269.87850000	78.86830000	0.00000000
78	1270.53540000	159.25570000	0.00000000
79	1289.23330000	141.49370000	0.00000000
80	1314.91990000	17.40960000	0.00000000
81	1318.86860000	83.02850000	0.00000000
82	1338.24960000	21.74540000	0.00000000
83	1352.55100000	20.53300000	0.00000000
84	1368.68460000	15.47020000	0.00000000
85	1394.66560000	139.23710000	0.00000000
86	1398.52340000	25.85230000	0.00000000
87	1401.27390000	48.73110000	0.00000000
88	1423.20100000	20.74060000	0.00000000
89	1424.31610000	26.01860000	0.00000000
90	1467.47320000	0.24370000	0.00000000
91	1483.99310000	1.85960000	0.00000000
92	1484.46470000	0.95100000	0.00000000
93	1488.30410000	29.24820000	0.00000000
94	1489.01240000	13.55000000	0.00000000
95	1493.67970000	16.01970000	0.00000000
96	1497.92350000	2.22870000	0.00000000
97	1514.34430000	64.99650000	0.00000000
98	1517.20140000	27.55920000	0.00000000
99	1537.41100000	15.45460000	0.00000000
100	1602.53170000	4.30570000	0.00000000
101	1629.37390000	2.91430000	0.00000000
102	1645.65230000	31.53090000	0.00000000
103	1649.55240000	16.03320000	0.00000000

104	1673.43130000	4.36900000	0.00000000
105	1733.80820000	690.58230000	0.00000000
106	2974.95680000	16.17280000	0.00000000
107	3042.40860000	14.71390000	0.00000000
108	3044.10740000	36.22060000	0.00000000
109	3050.84700000	20.58450000	0.00000000
110	3103.29500000	8.90790000	0.00000000
111	3105.13490000	25.29350000	0.00000000
112	3112.84700000	41.74250000	0.00000000
113	3118.08420000	62.43320000	0.00000000
114	3144.71080000	0.37610000	0.00000000
115	3147.59160000	25.56800000	0.00000000
116	3164.84720000	6.53880000	0.00000000
117	3170.80380000	2.37080000	0.00000000
118	3172.46900000	0.31750000	0.00000000
119	3174.43750000	14.31100000	0.00000000
120	3181.58670000	11.93880000	0.00000000
121	3181.83280000	36.14540000	0.00000000
122	3192.96770000	35.68460000	0.00000000
123	3195.27040000	30.22880000	0.00000000
124	3201.46250000	4.11600000	0.00000000
125	3212.45640000	9.35590000	0.00000000
126	3214.39650000	15.95490000	0.00000000

Clockwise Transition State

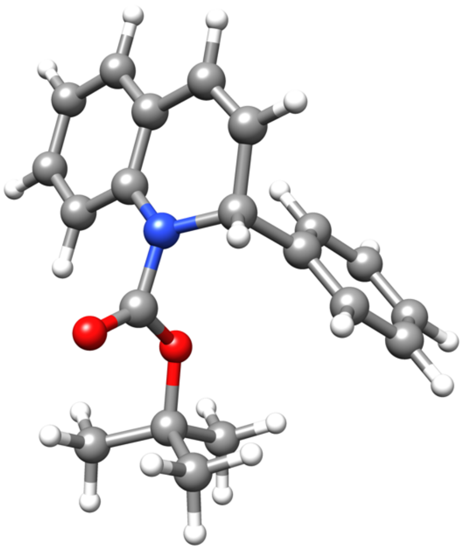

Route : # opt freq b3lyp scrf=(solvent=thf) geom=connectivity def2tzvp
empiricaldispersion=gd3bj int=ultrafine pop=(regular,mk)

SMILES : CC(C)(C)OC(=O)N2c1ccccc1C=CC2c3ccccc3

Formula : $C_{20}H_{21}NO_2$

Charge : 0

Multiplicity : 1

Dipole : 3.9691 Debye

Energy : -980.452812470 a.u.

Gibbs Energy : -980.136244 a.u.

Number of imaginary frequencies : 1

Cartesian Coordinates (XYZ format)

44

C	1.54572300	2.02163100	-1.54742100
C	0.34300000	1.14148000	-1.35585200
H	-0.16128200	1.02055000	-2.31860300
H	1.38615900	2.92464700	-2.12146100
C	2.72458300	1.75201100	-0.98599700
N	0.78112100	-0.21039600	-0.94569400
C	-0.23027300	-1.21799800	-0.98349100
O	-0.30769700	-2.02922700	-1.87091400
O	-1.03866400	-1.09986300	0.05667000
C	-2.25669100	-1.93020800	0.23364600
C	-3.18960100	-1.72650100	-0.95232100
H	-2.76861000	-2.13377200	-1.86813100

H	-4.13383200	-2.23317300	-0.74929300
H	-3.39470600	-0.66496500	-1.09244400
C	-1.85178600	-3.38479200	0.42417900
H	-2.73739100	-3.97216700	0.66982900
H	-1.40409300	-3.79383000	-0.47843100
H	-1.14386700	-3.47752600	1.24884000
C	-2.85776100	-1.34876900	1.50483400
H	-3.08227000	-0.29080600	1.36981100
H	-3.77978400	-1.87606200	1.75011500
H	-2.16388800	-1.45381600	2.33908000
H	3.55691100	2.43515200	-1.10662800
C	-0.66093500	1.77181700	-0.39802400
C	-0.29870100	2.07397900	0.91396700
C	-1.96179100	2.02719600	-0.81724000
C	-1.23027600	2.60358600	1.79518500
H	0.71238600	1.88282700	1.24901100
C	-2.89685300	2.56733900	0.06028400
H	-2.25015500	1.78977300	-1.83449500
C	-2.53365500	2.85154200	1.37064200
H	-0.94195600	2.82520600	2.81475200
H	-3.90739300	2.75786500	-0.27772300
H	-3.25952600	3.26483900	2.05889100
C	2.93772100	0.55284200	-0.19569800
C	4.11008100	0.32788400	0.52256400
C	1.91999600	-0.42554200	-0.18033900
C	4.29824900	-0.83966400	1.25165600
H	4.88330500	1.08660400	0.49981500
C	2.11329500	-1.60050600	0.54987200
C	3.29400300	-1.80145200	1.25501500
H	5.21395700	-0.99880300	1.80455900
H	1.34672600	-2.36295900	0.56932500
H	3.42239500	-2.71933000	1.81421600

Frequencies

Mode	IR frequency	IR intensity	Raman intensity
1	-36.04260000	1.67780000	0.00000000
2	31.19690000	0.13960000	0.00000000
3	49.01600000	0.91020000	0.00000000
4	49.64620000	0.15510000	0.00000000
5	56.62420000	0.31920000	0.00000000
6	79.24070000	0.92180000	0.00000000
7	99.15010000	0.52760000	0.00000000
8	127.99350000	3.77190000	0.00000000
9	163.57790000	1.69650000	0.00000000
10	174.72350000	0.03370000	0.00000000
11	194.44550000	4.71890000	0.00000000
12	207.50020000	0.04880000	0.00000000
13	220.14910000	4.24780000	0.00000000
14	247.67410000	0.73180000	0.00000000

15	250.93140000	0.35140000	0.00000000
16	270.81950000	0.66210000	0.00000000
17	276.87380000	3.25640000	0.00000000
18	321.10880000	8.67630000	0.00000000
19	334.61400000	4.52880000	0.00000000
20	354.69040000	1.97930000	0.00000000
21	387.14500000	3.58540000	0.00000000
22	405.54430000	2.38450000	0.00000000
23	414.96710000	0.14630000	0.00000000
24	415.82570000	0.12570000	0.00000000
25	453.51120000	5.49050000	0.00000000
26	473.19700000	7.02290000	0.00000000
27	476.11000000	12.38280000	0.00000000
28	521.57960000	0.05680000	0.00000000
29	533.01280000	2.51580000	0.00000000
30	541.69370000	5.58580000	0.00000000
31	559.64150000	6.35130000	0.00000000
32	585.78090000	23.72310000	0.00000000
33	636.41170000	0.23100000	0.00000000
34	644.19650000	4.81990000	0.00000000
35	672.56570000	5.34810000	0.00000000
36	714.71760000	63.23490000	0.00000000
37	724.78720000	17.91640000	0.00000000
38	750.51460000	47.69360000	0.00000000
39	758.33250000	55.91530000	0.00000000
40	775.70350000	8.14600000	0.00000000
41	783.04790000	50.71860000	0.00000000
42	796.26580000	10.77540000	0.00000000
43	801.39360000	17.86970000	0.00000000
44	830.62990000	21.45270000	0.00000000
45	848.06580000	58.79140000	0.00000000
46	856.73800000	35.72210000	0.00000000
47	860.61480000	0.80190000	0.00000000
48	863.12270000	13.19660000	0.00000000
49	894.11310000	9.79380000	0.00000000
50	933.56930000	0.46340000	0.00000000
51	934.82680000	5.19180000	0.00000000
52	935.08560000	0.07490000	0.00000000
53	939.15420000	5.17250000	0.00000000
54	958.02760000	0.20910000	0.00000000
55	976.59850000	0.07250000	0.00000000
56	982.43620000	0.34140000	0.00000000
57	995.39560000	1.67160000	0.00000000

58	999.98410000	0.62400000	0.00000000
59	1010.13570000	2.58030000	0.00000000
60	1026.26250000	0.90030000	0.00000000
61	1036.33510000	20.27340000	0.00000000
62	1053.93550000	8.53390000	0.00000000
63	1055.08640000	1.98430000	0.00000000
64	1062.82830000	0.13850000	0.00000000
65	1072.32060000	17.45660000	0.00000000
66	1106.38260000	27.21400000	0.00000000
67	1115.27680000	75.92360000	0.00000000
68	1149.45360000	177.65320000	0.00000000
69	1171.69810000	492.33060000	0.00000000
70	1180.75410000	0.48210000	0.00000000
71	1187.50660000	38.03060000	0.00000000
72	1195.81440000	7.06220000	0.00000000
73	1204.09960000	5.29450000	0.00000000
74	1210.55720000	29.08810000	0.00000000
75	1242.74850000	20.38920000	0.00000000
76	1264.21600000	296.09010000	0.00000000
77	1274.62300000	29.86460000	0.00000000
78	1284.74440000	59.09980000	0.00000000
79	1289.92660000	43.62360000	0.00000000
80	1291.43180000	239.40100000	0.00000000
81	1331.01560000	27.39660000	0.00000000
82	1347.34270000	21.08690000	0.00000000
83	1351.60180000	18.30980000	0.00000000
84	1378.37610000	42.69010000	0.00000000
85	1400.98160000	27.16380000	0.00000000
86	1404.02050000	31.86610000	0.00000000
87	1415.12310000	34.59250000	0.00000000
88	1424.96570000	28.72010000	0.00000000
89	1427.26670000	19.75100000	0.00000000
90	1469.16020000	0.30300000	0.00000000
91	1485.18150000	0.59400000	0.00000000
92	1486.16120000	1.91320000	0.00000000
93	1490.32400000	72.78120000	0.00000000
94	1492.10290000	15.05910000	0.00000000
95	1494.93680000	3.26960000	0.00000000
96	1497.52170000	4.12390000	0.00000000
97	1517.38700000	17.91600000	0.00000000
98	1527.00300000	120.90130000	0.00000000
99	1531.63830000	13.09160000	0.00000000
100	1608.05760000	21.87550000	0.00000000

101	1630.27990000	1.52200000	0.00000000
102	1640.02280000	85.26990000	0.00000000
103	1644.70900000	2.01950000	0.00000000
104	1699.86550000	22.91460000	0.00000000
105	1761.72230000	594.75040000	0.00000000
106	3026.57780000	43.04480000	0.00000000
107	3044.78510000	12.03930000	0.00000000
108	3047.88930000	30.20920000	0.00000000
109	3054.36470000	18.02670000	0.00000000
110	3107.34980000	12.36890000	0.00000000
111	3112.70750000	10.86040000	0.00000000
112	3117.45920000	42.34080000	0.00000000
113	3122.12880000	40.04630000	0.00000000
114	3139.49930000	5.23730000	0.00000000
115	3143.54200000	21.00220000	0.00000000
116	3162.40610000	8.36430000	0.00000000
117	3164.87770000	0.55470000	0.00000000
118	3167.55660000	22.02650000	0.00000000
119	3171.72240000	0.37160000	0.00000000
120	3176.52690000	9.66940000	0.00000000
121	3180.49730000	19.65960000	0.00000000
122	3189.17250000	32.57620000	0.00000000
123	3192.60720000	13.43730000	0.00000000
124	3192.83310000	32.23620000	0.00000000
125	3196.91260000	17.98390000	0.00000000
126	3200.61920000	18.36620000	0.00000000

Anticlockwise Transition State

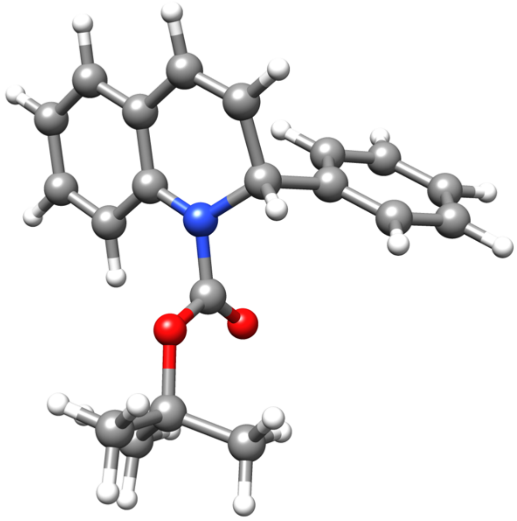

Route : # opt freq b3lyp scrf=(solvent=thf) geom=connectivity def2tzvp
empiricaldispersion=gd3bj int=ultrafine pop=(regular,mk)

SMILES : CC(C)(C)OC(=O)N2c1ccccc1C=CC2c3ccccc3

Formula : $C_{20}H_{21}NO_2$

Charge : 0

Multiplicity : 1

Dipole : 2.9115 Debye

Energy : -980.450611469 a.u.

Gibbs Energy : -980.135492 a.u.

Number of imaginary frequencies : 1

Cartesian Coordinates (XYZ format)

44

C	-0.88908400	0.41514500	1.23062000
C	-1.74253500	-0.49023600	2.07387500
H	-0.26815200	1.02758200	1.89132000
H	-2.25565400	-0.02097800	2.90270900
C	-1.91959900	-1.78001500	1.78540200
N	0.03640900	-0.39404400	0.41551900
C	1.09144300	0.33353900	-0.20953600
O	0.95573100	0.98175300	-1.21540500
O	2.20189600	0.19665100	0.50126300
C	3.47525200	0.85607300	0.10511800
C	3.91806800	0.33219500	-1.25431500

H	3.24699400	0.65528700	-2.04593500
H	4.91778700	0.71064300	-1.47074800
H	3.96238900	-0.75767200	-1.24464900
C	3.29161800	2.36738600	0.12624800
H	4.26012000	2.84454100	-0.02814600
H	2.61334700	2.69867100	-0.65565100
H	2.90517200	2.68857800	1.09449500
C	4.43001500	0.40564800	1.20065300
H	4.51477000	-0.68113800	1.21290500
H	5.41843600	0.82933900	1.02271700
H	4.07943100	0.74021800	2.17723900
H	-2.58021000	-2.38939900	2.39064700
C	-1.74316000	1.36710900	0.40315100
C	-2.64472700	0.87188400	-0.53855400
C	-1.63251900	2.74199500	0.57836700
C	-3.41389800	1.74123100	-1.29842800
H	-2.74058500	-0.19686500	-0.67967400
C	-2.40930000	3.61718000	-0.17497600
H	-0.92858800	3.13242300	1.30402900
C	-3.29966900	3.11778600	-1.11695300
H	-4.10604700	1.34757700	-2.03174300
H	-2.31378700	4.68558700	-0.02959800
H	-3.90211000	3.79499300	-1.70847900
C	-1.24804200	-2.41278400	0.66469500
C	-1.52356200	-3.71898000	0.26609500
C	-0.25985200	-1.67511300	-0.02336000
C	-0.84398200	-4.31070200	-0.79134400
H	-2.28288900	-4.27380800	0.80433400
C	0.42429500	-2.27282300	-1.08503100
C	0.13295500	-3.57945400	-1.45840200
H	-1.07049000	-5.32541400	-1.08898600
H	1.17808100	-1.71878700	-1.62753500
H	0.67380100	-4.02268700	-2.28470000

Frequencies

Mode	IR frequency	IR intensity	Raman intensity
1	-40.75020000	1.60440000	0.00000000
2	21.09080000	0.36170000	0.00000000
3	29.85360000	0.09910000	0.00000000
4	43.06280000	0.29330000	0.00000000
5	46.34480000	1.10140000	0.00000000
6	76.59300000	0.40020000	0.00000000
7	106.24310000	0.63570000	0.00000000
8	131.39390000	4.10290000	0.00000000
9	159.33740000	2.23270000	0.00000000
10	175.68580000	0.58190000	0.00000000
11	195.25120000	1.58270000	0.00000000
12	204.57300000	1.14840000	0.00000000
13	206.66060000	0.65620000	0.00000000

14	246.55340000	3.14920000	0.00000000
15	248.77690000	0.04270000	0.00000000
16	270.04270000	0.05750000	0.00000000
17	287.94200000	3.73030000	0.00000000
18	320.41670000	5.21270000	0.00000000
19	324.59320000	5.78600000	0.00000000
20	355.08130000	2.43320000	0.00000000
21	390.61990000	3.49820000	0.00000000
22	402.66780000	2.30800000	0.00000000
23	413.09840000	0.48450000	0.00000000
24	414.49080000	1.73920000	0.00000000
25	449.43560000	1.20310000	0.00000000
26	473.10150000	8.84790000	0.00000000
27	476.05030000	16.10920000	0.00000000
28	513.73670000	10.38190000	0.00000000
29	538.80810000	8.79400000	0.00000000
30	548.49710000	17.81870000	0.00000000
31	557.27770000	5.00600000	0.00000000
32	584.80520000	6.11480000	0.00000000
33	636.11450000	0.24830000	0.00000000
34	647.60270000	7.10860000	0.00000000
35	669.35830000	12.36340000	0.00000000
36	711.90210000	57.70310000	0.00000000
37	725.55770000	17.06200000	0.00000000
38	751.29030000	24.27300000	0.00000000
39	755.81200000	80.67140000	0.00000000
40	774.68490000	18.69230000	0.00000000
41	782.09110000	39.12600000	0.00000000
42	795.45980000	18.32730000	0.00000000
43	804.50090000	9.21110000	0.00000000
44	832.75350000	6.70690000	0.00000000
45	845.57280000	79.18300000	0.00000000
46	856.85550000	22.64180000	0.00000000
47	857.84390000	7.38110000	0.00000000
48	864.83090000	11.75560000	0.00000000
49	892.91140000	6.67760000	0.00000000
50	931.37280000	4.98930000	0.00000000
51	933.79640000	0.01610000	0.00000000
52	934.41390000	0.09950000	0.00000000
53	940.11140000	4.99060000	0.00000000
54	960.54010000	0.03320000	0.00000000
55	976.24190000	0.04220000	0.00000000
56	976.38460000	0.12330000	0.00000000

57	993.56280000	0.24110000	0.00000000
58	995.36960000	2.24680000	0.00000000
59	1009.95800000	1.61800000	0.00000000
60	1025.51070000	1.44500000	0.00000000
61	1036.42260000	47.47250000	0.00000000
62	1053.50170000	8.67110000	0.00000000
63	1054.75500000	1.52880000	0.00000000
64	1062.38350000	2.77880000	0.00000000
65	1072.07490000	15.61710000	0.00000000
66	1106.54340000	20.95200000	0.00000000
67	1121.35230000	66.26930000	0.00000000
68	1149.17830000	240.13810000	0.00000000
69	1172.14210000	569.68700000	0.00000000
70	1180.93380000	0.11480000	0.00000000
71	1187.18050000	44.02280000	0.00000000
72	1196.40700000	8.81110000	0.00000000
73	1203.55640000	3.39510000	0.00000000
74	1212.30170000	14.98670000	0.00000000
75	1242.95200000	23.44830000	0.00000000
76	1267.03760000	478.85240000	0.00000000
77	1273.51670000	29.03050000	0.00000000
78	1285.35710000	14.53240000	0.00000000
79	1287.38700000	5.59480000	0.00000000
80	1294.57210000	265.41620000	0.00000000
81	1331.03160000	64.84500000	0.00000000
82	1347.51060000	20.32910000	0.00000000
83	1350.65690000	15.67700000	0.00000000
84	1376.90020000	41.59110000	0.00000000
85	1401.21140000	26.10840000	0.00000000
86	1403.91030000	28.11760000	0.00000000
87	1416.75560000	26.09280000	0.00000000
88	1426.36710000	27.47880000	0.00000000
89	1427.25400000	22.66470000	0.00000000
90	1468.94710000	0.44490000	0.00000000
91	1483.77920000	0.24270000	0.00000000
92	1485.31610000	2.25120000	0.00000000
93	1490.38380000	69.90230000	0.00000000
94	1491.44030000	20.19470000	0.00000000
95	1495.30400000	9.91040000	0.00000000
96	1498.02270000	2.73060000	0.00000000
97	1517.45220000	24.14190000	0.00000000
98	1527.83530000	114.07930000	0.00000000
99	1531.48850000	15.77930000	0.00000000

100	1607.35800000	21.86880000	0.00000000
101	1630.10230000	1.70430000	0.00000000
102	1640.15310000	96.06890000	0.00000000
103	1644.74430000	0.52270000	0.00000000
104	1699.22820000	26.26060000	0.00000000
105	1759.85220000	435.03340000	0.00000000
106	3017.84990000	48.26540000	0.00000000
107	3044.41500000	14.18990000	0.00000000
108	3046.93070000	32.74440000	0.00000000
109	3053.24720000	14.87150000	0.00000000
110	3106.86360000	8.52350000	0.00000000
111	3109.34780000	16.95030000	0.00000000
112	3115.55380000	39.35580000	0.00000000
113	3120.60780000	53.19370000	0.00000000
114	3143.05500000	0.53600000	0.00000000
115	3145.92620000	25.82880000	0.00000000
116	3162.28610000	9.48840000	0.00000000
117	3164.90140000	0.84910000	0.00000000
118	3167.14940000	22.00500000	0.00000000
119	3171.51180000	0.46190000	0.00000000
120	3176.50390000	10.28620000	0.00000000
121	3180.16450000	18.46670000	0.00000000
122	3188.43550000	35.07660000	0.00000000
123	3192.43260000	14.60830000	0.00000000
124	3193.00600000	29.07340000	0.00000000
125	3196.63020000	20.02300000	0.00000000
126	3200.57090000	18.01150000	0.00000000

Thermochemistry Values

Left Rotamer Axial:

	Temperature	
	298 K	195 K
ε_0	–980.468612027 a.u.	–980.468612027 a.u.
ε_{ZPE}	0.365354 a.u.	0.365354 a.u.
E_{tot}	0.385767 a.u.	0.374725 a.u.
H_{corr}	0.386711 a.u.	0.375343 a.u.
G_{corr}	0.315032 a.u.	0.337461 a.u.
S_{tot}	150.861 calmol^{-1}K^{-1}	121.903 calmol^{-1}K^{-1}
$\varepsilon_0 + \varepsilon_{ZPE}$	–980.103258 a.u.	–980.103258 a.u.
$\varepsilon_0 + E_{tot}$	–980.082845 a.u.	–980.093887 a.u.
$\varepsilon_0 + H_{corr}$	–980.081901 a.u.	–980.093269 a.u.
$\varepsilon_0 + G_{corr}$	–980.153580 a.u.	–980.131151 a.u.

Right Rotamer Axial:

	Temperature	
	298 K	195 K
ε_0	–980.467667697 a.u.	–980.467667697 a.u.
ε_{ZPE}	0.365248 a.u.	0.365248 a.u.
E_{tot}	0.385694 a.u.	0.374659 a.u.
H_{corr}	0.386639 a.u.	0.375276 a.u.
G_{corr}	0.314342 a.u.	0.336986 a.u.
S_{tot}	152.161 calmol^{-1}K^{-1}	123.219 calmol^{-1}K^{-1}
$\varepsilon_0 + \varepsilon_{ZPE}$	–980.102420 a.u.	–980.102420 a.u.
$\varepsilon_0 + E_{tot}$	–980.081973 a.u.	–980.093009 a.u.
$\varepsilon_0 + H_{corr}$	–980.081029 a.u.	–980.092392 a.u.
$\varepsilon_0 + G_{corr}$	–980.153326 a.u.	–980.130682 a.u.

Left Rotamer Equatorial:

	Temperature	
	298 K	195 K
ε_0	–980.456213568 a.u.	–980.456213568 a.u.
ε_{ZPE}	0.364894 a.u.	0.364894 a.u.
E_{tot}	0.385264 a.u.	0.374211 a.u.
H_{corr}	0.386208 a.u.	0.374829 a.u.
G_{corr}	0.315469 a.u.	0.337570 a.u.
S_{tot}	148.883 calmol^{-1}K^{-1}	119.897 calmol^{-1}K^{-1}
$\varepsilon_0 + \varepsilon_{ZPE}$	–980.091320 a.u.	–980.091320 a.u.
$\varepsilon_0 + E_{tot}$	–980.070949 a.u.	–980.082003 a.u.
$\varepsilon_0 + H_{corr}$	–980.070005 a.u.	–980.081385 a.u.
$\varepsilon_0 + G_{corr}$	–980.140744 a.u.	–980.118644 a.u.

Right Rotamer Equatorial:

	Temperature	
	298 K	195 K
ε_0	–980.454619636 a.u.	–980.454619636 a.u.
ε_{ZPE}	0.364566 a.u.	0.364566 a.u.
E_{tot}	0.385089 a.u.	0.374023 a.u.
H_{corr}	0.386033 a.u.	0.374640 a.u.
G_{corr}	0.314036 a.u.	0.336570 a.u.
S_{tot}	151.531 calmol^{-1}K^{-1}	122.510 calmol^{-1}K^{-1}
$\varepsilon_0 + \varepsilon_{ZPE}$	–980.090054 a.u.	–980.090054 a.u.
$\varepsilon_0 + E_{tot}$	–980.069531 a.u.	–980.080597 a.u.
$\varepsilon_0 + H_{corr}$	–980.068587 a.u.	–980.079980 a.u.
$\varepsilon_0 + G_{corr}$	–980.140584 a.u.	–980.118050 a.u.

Clockwise Transition State:

	Temperature	
	298 K	195 K
ε_0	–980.452812470 a.u.	–980.452812470 a.u.
ε_{ZPE}	0.364505 a.u.	0.364505 a.u.
E_{tot}	0.384190 a.u.	0.373413 a.u.
H_{corr}	0.385134 a.u.	0.374030 a.u.
G_{corr}	0.316569 a.u.	0.337973 a.u.
S_{tot}	144.309 calmol^{-1}K^{-1}	116.033 calmol^{-1}K^{-1}
$\varepsilon_0 + \varepsilon_{ZPE}$	–980.088307 a.u.	–980.088307 a.u.
$\varepsilon_0 + E_{tot}$	–980.068622 a.u.	–980.079399 a.u.
$\varepsilon_0 + H_{corr}$	–980.067678 a.u.	–980.078782 a.u.
$\varepsilon_0 + G_{corr}$	–980.136244 a.u.	–980.114839 a.u.

Anticlockwise Transition State:

	Temperature	
	298 K	195 K
ε_0	–980.450611469 a.u.	–980.450611469 a.u.
ε_{ZPE}	0.364289 a.u.	0.364289 a.u.
E_{tot}	0.384099 a.u.	0.373307 a.u.
H_{corr}	0.385044 a.u.	0.373925 a.u.
G_{corr}	0.315120 a.u.	0.336991 a.u.
S_{tot}	147.167 calmol^{-1}K^{-1}	118.853 calmol^{-1}K^{-1}
$\varepsilon_0 + \varepsilon_{ZPE}$	–980.086322 a.u.	–980.086322 a.u.
$\varepsilon_0 + E_{tot}$	–980.066512 a.u.	–980.077304 a.u.
$\varepsilon_0 + H_{corr}$	–980.065568 a.u.	–980.076686 a.u.
$\varepsilon_0 + G_{corr}$	–980.135492 a.u.	–980.113620 a.u.

8.2 Lithiation pathway calculations

DFT calculations were performed to investigate the lithiation pathway. Unlike the individual rotamer calculations, these calculations were performed using the B3LYP-D3BJ functional with the 6-311G(d,p) basis set (B3LYP-D3BJ//6-311G(d,p)). The relative changes in Gibbs energy are shown in the energy profile in Figure 6 of the manuscript (copied below).

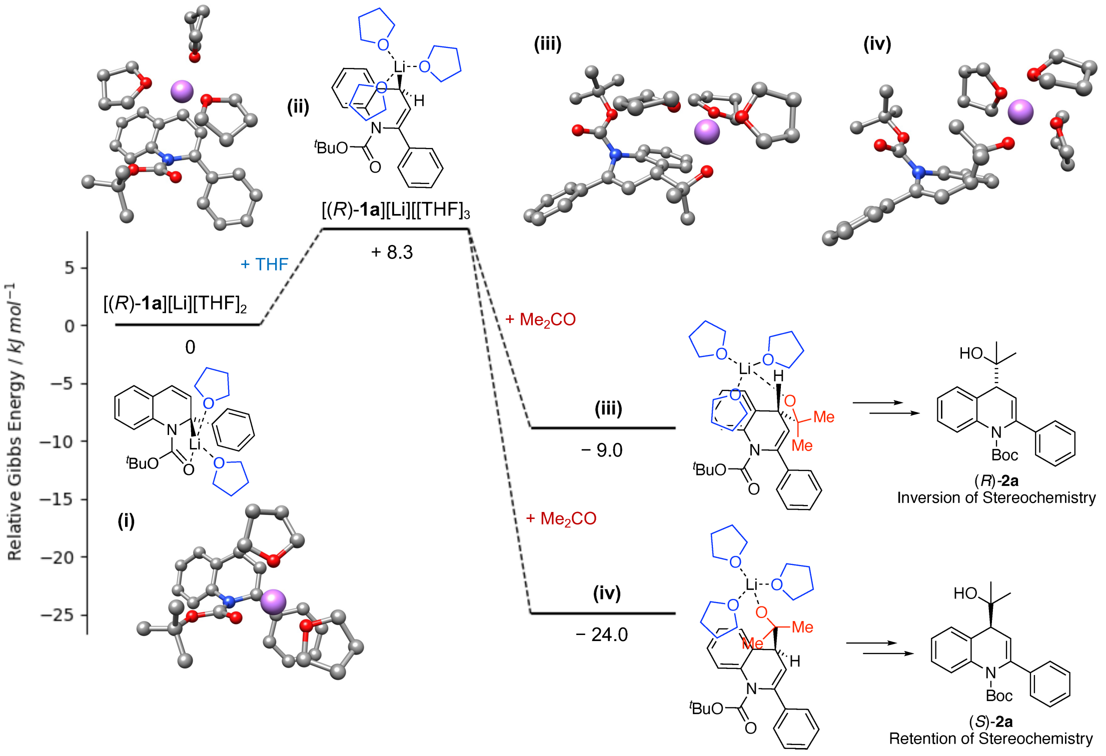

The first calculation performed was an optimisation of lithiated **1a** which had been stabilised with two THF molecules, which gave the structure shown in Figure 6(i). By introducing another molecule of THF and reoptimizing the structure the lithium atom was found to migrate from the 2-position to the 4-position of the dihydroquinoline as shown in Figure 6(ii). With these optimised structures we investigated the quenching with a molecule of acetone. Acetone could approach the 4-position of the dihydroquinoline from above or below the C-4 position, therefore both scenarios were investigated. The more energetically favourable process was found to be when the molecule of acetone approached C-4 from the same side as the lithium atom to give the structure shown in Figure 6(iv). In comparison when acetone approached from opposite the lithium atom, the structure shown in Figure 6(iii) was obtained.

Overall, the results obtained from these DFT studies indicate that there is a preference for the retention of stereochemistry during the lithiation process, rather than inversion. This is also supported by the experimental results.

Tetrahydrofuran

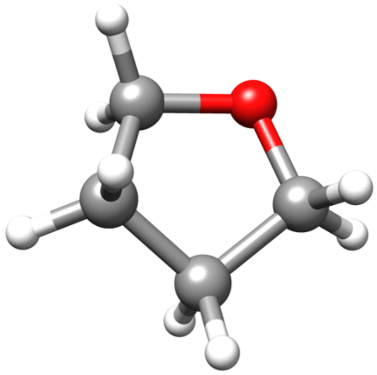

Route : # opt freq b3lyp/6-311g(d,p) scrf=(solvent=thf)
geom=connectivity empiricaldispersion=gd3bj
int=ultrafine
pop=(regular,mk)

SMILES : C1CCOC1

Formula : C_4H_8O

Charge : 0

Multiplicity : 1

Dipole : 2.1836 Debye

Energy : -232.532329517 a.u.

Gibbs Energy : -232.444268 a.u.

Number of imaginary frequencies : 0

Cartesian Coordinates (XYZ format)

13

C	-0.73112400	0.99314700	-0.23199500
C	0.73107900	0.99316600	0.23202100
C	-1.16882300	-0.42560100	0.13389800
H	-0.78449800	1.14298000	-1.31410300
H	-1.34018100	1.75827700	0.25176100
C	1.16882400	-0.42553900	-0.13396100
H	0.78443700	1.14291800	1.31414200
H	1.34010700	1.75835600	-0.25167300
O	0.00004800	-1.25447600	0.00004900
H	-1.95152600	-0.81786300	-0.51990200
H	-1.52649200	-0.47114300	1.17031300
H	1.95165700	-0.81775200	0.51971400
H	1.52637300	-0.47100700	-1.17042800

Frequencies

Mode	IR frequency	IR intensity	Raman intensity
1	68.96700000	7.73630000	0.00000000
2	261.63880000	0.00340000	0.00000000
3	580.21850000	1.28330000	0.00000000
4	672.51130000	5.13810000	0.00000000
5	845.42890000	12.07610000	0.00000000
6	882.86320000	12.92160000	0.00000000
7	899.99890000	31.64710000	0.00000000
8	909.98520000	9.14020000	0.00000000
9	927.36130000	9.19780000	0.00000000
10	969.08250000	0.29810000	0.00000000
11	1032.69870000	4.98050000	0.00000000
12	1074.94040000	164.44860000	0.00000000
13	1168.57970000	0.03520000	0.00000000
14	1186.53430000	7.38750000	0.00000000
15	1187.67940000	10.67360000	0.00000000
16	1258.32760000	8.41480000	0.00000000
17	1259.21310000	0.04650000	0.00000000
18	1319.89710000	2.46700000	0.00000000
19	1342.29010000	0.86220000	0.00000000
20	1364.90310000	0.29820000	0.00000000
21	1399.62170000	3.70440000	0.00000000
22	1485.13550000	5.77070000	0.00000000
23	1494.21160000	7.80890000	0.00000000
24	1519.02330000	1.14390000	0.00000000
25	1530.64640000	0.05760000	0.00000000
26	2990.81460000	202.69030000	0.00000000
27	2997.50150000	7.08610000	0.00000000
28	3039.13930000	26.70670000	0.00000000
29	3043.54000000	20.43120000	0.00000000
30	3064.18560000	159.70530000	0.00000000
31	3067.15180000	5.35930000	0.00000000
32	3097.85070000	26.07800000	0.00000000
33	3104.95030000	78.18220000	0.00000000

Lithiated Dihydroquinoline with Two THF Molecules (Figure 6i)

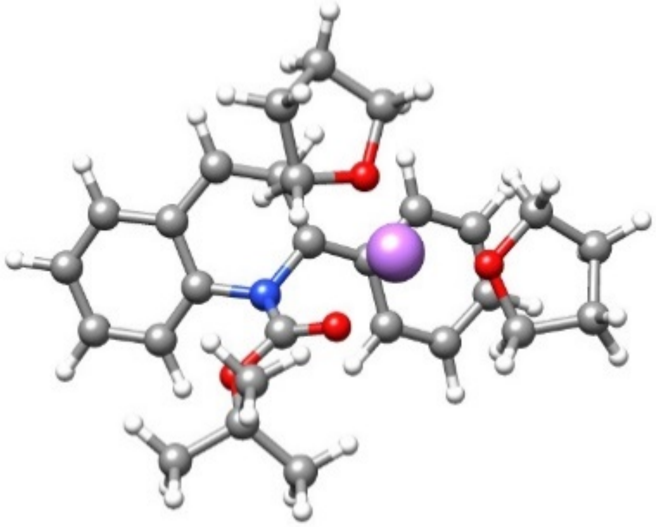

Route : # opt freq b3lyp/6-311g(d,p) scrf=(solvent=thf)
geom=connectivity empiricaldispersion=gd3bj
int=ultrafine
pop=(regular,mk)

SMILES :
[Li].CC(C)(C)OC(=O)N2c1ccccc1C=CC2c3ccccc3.C1CCOC1.C1CCOC1

Formula : $C_{28}H_{36}LiNO_4$

Charge : 0

Multiplicity : 1

Dipole : 10.9274 Debye

Energy : -1452.45684804 a.u.

Gibbs Energy : -1451.938242 a.u.

Number of imaginary frequencies : 0

Cartesian Coordinates (XYZ format)

70

H	2.50855200	-0.73853800	-3.21705000
H	2.00197600	-2.18494200	-4.10429100
C	1.84117300	-1.60307400	-3.19362800
H	0.81037500	-1.25570200	-3.17515100
H	4.29087000	-2.15400500	-2.10393800
H	3.72107400	-3.62766400	-2.91338400
C	3.59041200	-2.98861100	-2.03781400
C	2.15430400	-2.47522200	-1.97891400
H	2.10704600	3.44405000	1.02393600
C	1.88108700	2.38508800	1.05352100
O	2.16944700	-1.65441600	-0.75168000
H	-0.19464100	2.67584200	1.45287000

C	0.58176900	1.94366000	1.25578400
C	1.09644500	-0.98045600	-0.33457000
O	-0.00160200	-1.01469500	-0.90880300
H	4.56543900	2.84769100	1.47248600
H	3.82486000	-3.56821100	-1.14294800
C	2.95012500	1.44209400	1.22866700
C	4.28833900	1.79868200	1.50436200
H	1.26081700	-4.30744000	-2.66857000
C	1.16866000	-3.63068000	-1.81569400
N	1.31068300	-0.28767700	0.81699700
Li	-1.39858500	0.23289500	-0.85856900
C	2.63741100	0.05721400	1.25810200
C	5.23337100	0.84039100	1.84272800
C	0.25449200	0.58469400	1.31208600
H	0.14444000	-3.26711400	-1.76181200
H	6.25350300	1.14509800	2.05130400
C	3.56922600	-0.89421900	1.64651700
C	4.87856900	-0.51042800	1.93642700
H	1.39577100	-4.19263500	-0.90670500
H	3.27955800	-1.93701100	1.67284700
H	5.61516700	-1.25555800	2.21202000
C	-0.91802600	-0.00442100	1.90325400
H	-1.97829800	1.86143700	2.24373200
H	-0.23507600	-2.05165100	1.76855500
C	-2.02404300	0.78243500	2.33002800
C	-1.05315700	-1.40860900	2.06611900
C	-3.16342400	0.20590300	2.86942700
C	-2.19323900	-1.97234000	2.62318400
C	-3.26904500	-1.17969900	3.02719200
H	-3.98204000	0.84799400	3.17870600
H	-2.24316600	-3.05029500	2.74063400
H	-4.15888800	-1.62395200	3.45636300
C	-5.11922400	-1.68441800	-0.76665900
C	-5.40881700	-0.37010300	-0.02843000
C	-3.59623000	-1.69782000	-0.80656100
H	-5.52962900	-1.65526200	-1.77962700
H	-5.52375600	-2.55846800	-0.25491400
C	-4.32327800	0.55843200	-0.56685000
H	-5.28063800	-0.50840700	1.04646200
H	-6.41085600	0.01685500	-0.21694000
O	-3.20990000	-0.30194800	-0.91855400
H	-3.97593400	1.28081500	0.17295400
H	-4.64290200	1.08541100	-1.47040400
H	-3.18024100	-2.22727200	-1.66434600
H	-3.16861000	-2.09900800	0.11445900
O	-1.00860300	1.86533000	-1.75967600
C	-1.59160400	3.14630200	-1.41599400
C	0.32267000	2.05257600	-2.31527600
C	0.40948400	3.53607300	-2.65258300
H	0.41856500	1.39499200	-3.17919900
H	1.05380900	1.77603800	-1.55276300
C	-0.46496700	4.16569900	-1.55933300
H	-1.98969300	3.08069300	-0.40170000
H	-2.41381600	3.34609900	-2.10948000
H	0.09919800	4.23886200	-0.62791100
H	-0.83799400	5.15596300	-1.82296200
H	-0.01146500	3.73307800	-3.64223900
H	1.43796600	3.89824000	-2.63511600

Frequencies

Mode	IR frequency	IR intensity	Raman intensity
1	15.48420000	0.86580000	0.00000000
2	26.23720000	0.38430000	0.00000000
3	27.74920000	2.25930000	0.00000000
4	34.53600000	0.87210000	0.00000000
5	39.91950000	0.94730000	0.00000000
6	42.80780000	0.42970000	0.00000000
7	44.08290000	1.10540000	0.00000000
8	49.41210000	0.84300000	0.00000000
9	58.70020000	2.33220000	0.00000000
10	67.96320000	0.53620000	0.00000000
11	75.20070000	0.46330000	0.00000000
12	81.79900000	1.41470000	0.00000000
13	86.51720000	2.04090000	0.00000000
14	87.72300000	0.73260000	0.00000000
15	88.59750000	1.32310000	0.00000000
16	98.22900000	1.83690000	0.00000000
17	105.80740000	5.57150000	0.00000000
18	108.69210000	0.40770000	0.00000000
19	116.33500000	9.20210000	0.00000000
20	134.99080000	5.99310000	0.00000000
21	148.61260000	3.09150000	0.00000000
22	169.05910000	5.30040000	0.00000000
23	174.86400000	2.50910000	0.00000000
24	184.63410000	3.50140000	0.00000000
25	210.48630000	0.52860000	0.00000000
26	211.14190000	1.85430000	0.00000000
27	242.30470000	6.77670000	0.00000000
28	250.78070000	9.74050000	0.00000000
29	256.21720000	3.16700000	0.00000000
30	258.39980000	11.04960000	0.00000000
31	262.63310000	79.95290000	0.00000000
32	271.05550000	3.84570000	0.00000000
33	273.28900000	0.55800000	0.00000000
34	315.30260000	6.94900000	0.00000000
35	332.24620000	26.97010000	0.00000000
36	349.07760000	4.64210000	0.00000000
37	353.04300000	3.09260000	0.00000000
38	395.54870000	27.90150000	0.00000000

39	421.40100000	0.88390000	0.00000000
40	425.90020000	3.08770000	0.00000000
41	446.34210000	6.15730000	0.00000000
42	456.96590000	7.42260000	0.00000000
43	471.61920000	40.34890000	0.00000000
44	479.33230000	0.71680000	0.00000000
45	492.62670000	80.43900000	0.00000000
46	506.20840000	16.10720000	0.00000000
47	513.96570000	39.04920000	0.00000000
48	544.37610000	55.32760000	0.00000000
49	558.79110000	53.91990000	0.00000000
50	569.60610000	106.35930000	0.00000000
51	578.94950000	1.60970000	0.00000000
52	581.12310000	2.11560000	0.00000000
53	597.28920000	23.09860000	0.00000000
54	605.59740000	6.65680000	0.00000000
55	635.91430000	0.31650000	0.00000000
56	684.67330000	11.08950000	0.00000000
57	691.66230000	62.31640000	0.00000000
58	693.88980000	18.13770000	0.00000000
59	702.24690000	63.06460000	0.00000000
60	704.91180000	44.21470000	0.00000000
61	714.13960000	18.48350000	0.00000000
62	743.79080000	17.71470000	0.00000000
63	748.86980000	162.24050000	0.00000000
64	753.98140000	36.47750000	0.00000000
65	769.46550000	2.35560000	0.00000000
66	779.11470000	34.29850000	0.00000000
67	806.36550000	2.11370000	0.00000000
68	826.57730000	0.55780000	0.00000000
69	849.94480000	21.15770000	0.00000000
70	854.97870000	23.64460000	0.00000000
71	855.36670000	5.39530000	0.00000000
72	856.04060000	12.90060000	0.00000000
73	867.33670000	9.24180000	0.00000000
74	873.35520000	32.60360000	0.00000000
75	876.10600000	24.33930000	0.00000000
76	886.82200000	25.97150000	0.00000000
77	888.59100000	97.75880000	0.00000000
78	894.35250000	41.12200000	0.00000000
79	911.57490000	86.14090000	0.00000000
80	918.21680000	5.59350000	0.00000000
81	920.69930000	11.31030000	0.00000000

82	926.74410000	94.82110000	0.00000000
83	928.47590000	7.10150000	0.00000000
84	929.07330000	3.39970000	0.00000000
85	929.49540000	6.38390000	0.00000000
86	931.69450000	11.98750000	0.00000000
87	935.08520000	36.09920000	0.00000000
88	965.69400000	2.07370000	0.00000000
89	966.73940000	0.95840000	0.00000000
90	971.67360000	0.28540000	0.00000000
91	973.11350000	0.40220000	0.00000000
92	975.33300000	1.02150000	0.00000000
93	978.12280000	4.97350000	0.00000000
94	997.59710000	145.85370000	0.00000000
95	1027.15070000	57.82540000	0.00000000
96	1043.41650000	12.33130000	0.00000000
97	1045.39350000	5.65350000	0.00000000
98	1046.15780000	39.14460000	0.00000000
99	1050.50040000	24.84660000	0.00000000
100	1051.61610000	6.46830000	0.00000000
101	1052.46010000	26.04680000	0.00000000
102	1054.88780000	222.83290000	0.00000000
103	1061.33850000	8.49890000	0.00000000
104	1094.47140000	40.94820000	0.00000000
105	1105.71010000	54.36460000	0.00000000
106	1137.29300000	113.30780000	0.00000000
107	1143.31870000	103.39400000	0.00000000
108	1168.45560000	2.43820000	0.00000000
109	1169.30360000	3.37980000	0.00000000
110	1169.91590000	44.06030000	0.00000000
111	1171.35250000	5.46880000	0.00000000
112	1191.21040000	63.84670000	0.00000000
113	1191.56040000	138.09240000	0.00000000
114	1191.70260000	219.41070000	0.00000000
115	1196.91480000	4.81880000	0.00000000
116	1199.71510000	4.13680000	0.00000000
117	1202.92340000	140.85430000	0.00000000
118	1238.88040000	7.51740000	0.00000000
119	1257.20980000	288.65920000	0.00000000
120	1262.76510000	3.89640000	0.00000000
121	1265.90130000	0.43990000	0.00000000
122	1268.67710000	22.60790000	0.00000000
123	1269.14960000	6.89290000	0.00000000
124	1271.88700000	8.77110000	0.00000000

125	1275.93170000	192.70460000	0.00000000
126	1279.00690000	32.02280000	0.00000000
127	1296.69970000	397.34880000	0.00000000
128	1325.66030000	0.55350000	0.00000000
129	1326.52890000	117.20590000	0.00000000
130	1327.27110000	32.72640000	0.00000000
131	1341.59880000	20.20630000	0.00000000
132	1349.09370000	37.59750000	0.00000000
133	1349.89950000	215.50720000	0.00000000
134	1350.82550000	10.86620000	0.00000000
135	1362.49120000	98.98390000	0.00000000
136	1374.93800000	1.64940000	0.00000000
137	1376.38920000	2.39180000	0.00000000
138	1390.76970000	634.14910000	0.00000000
139	1394.92410000	30.13450000	0.00000000
140	1400.22980000	224.09330000	0.00000000
141	1405.24680000	5.75370000	0.00000000
142	1406.89230000	3.82780000	0.00000000
143	1421.66280000	29.05880000	0.00000000
144	1445.54350000	245.55170000	0.00000000
145	1466.92080000	0.18840000	0.00000000
146	1472.80260000	761.90340000	0.00000000
147	1480.70350000	812.75170000	0.00000000
148	1483.79930000	11.97600000	0.00000000
149	1484.77970000	2.34620000	0.00000000
150	1485.25110000	19.03150000	0.00000000
151	1486.01150000	2.94960000	0.00000000
152	1487.97370000	23.36620000	0.00000000
153	1492.22570000	17.64040000	0.00000000
154	1493.55700000	9.25660000	0.00000000
155	1499.66930000	0.59280000	0.00000000
156	1501.43040000	48.53130000	0.00000000
157	1512.61640000	6.96360000	0.00000000
158	1513.79570000	1.91200000	0.00000000
159	1520.22210000	33.86730000	0.00000000
160	1523.81140000	6.64260000	0.00000000
161	1525.00520000	19.03020000	0.00000000
162	1526.94720000	4.24330000	0.00000000
163	1546.79180000	771.68800000	0.00000000
164	1580.81870000	28.98340000	0.00000000
165	1589.99580000	59.34520000	0.00000000
166	1630.89370000	94.91270000	0.00000000
167	1634.83220000	187.87280000	0.00000000

168	1662.17620000	613.16210000	0.00000000
169	3032.71600000	55.43490000	0.00000000
170	3035.03090000	114.94960000	0.00000000
171	3038.52220000	17.70580000	0.00000000
172	3039.50960000	30.50250000	0.00000000
173	3046.46300000	18.63250000	0.00000000
174	3046.58030000	41.73970000	0.00000000
175	3047.02810000	44.65380000	0.00000000
176	3054.60790000	38.59330000	0.00000000
177	3058.99970000	44.64170000	0.00000000
178	3064.83950000	18.68330000	0.00000000
179	3065.94680000	23.13460000	0.00000000
180	3089.44650000	23.78900000	0.00000000
181	3094.75210000	40.14010000	0.00000000
182	3101.18840000	11.53230000	0.00000000
183	3101.53740000	16.61150000	0.00000000
184	3103.07110000	31.29920000	0.00000000
185	3103.66310000	17.34210000	0.00000000
186	3111.27210000	20.56610000	0.00000000
187	3112.63590000	21.16130000	0.00000000
188	3113.54760000	41.85110000	0.00000000
189	3116.80820000	62.25090000	0.00000000
190	3119.78500000	11.53590000	0.00000000
191	3120.03330000	133.59220000	0.00000000
192	3142.15380000	2.88680000	0.00000000
193	3144.61220000	30.12390000	0.00000000
194	3145.61930000	27.51420000	0.00000000
195	3147.44590000	13.36790000	0.00000000
196	3148.11400000	23.46280000	0.00000000
197	3152.50320000	18.57390000	0.00000000
198	3163.39290000	49.06460000	0.00000000
199	3172.69020000	4.96470000	0.00000000
200	3176.33640000	78.11650000	0.00000000
201	3177.53160000	56.30800000	0.00000000
202	3183.19880000	52.63880000	0.00000000
203	3191.00190000	11.12010000	0.00000000
204	3194.36910000	28.30140000	0.00000000

Lithiated Dihydroquinoline with Three THF Molecules (Figure 6ii)

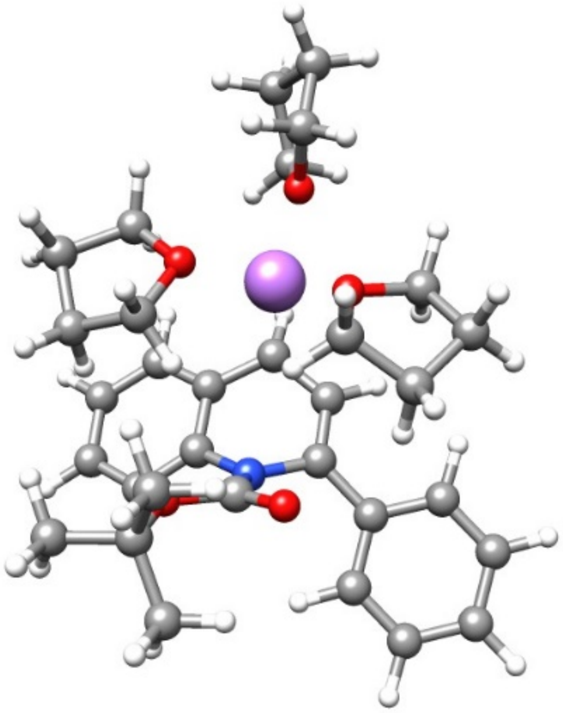

Route : # opt freq b3lyp/6-311g(d,p) scrf=(solvent=thf)
geom=connectivity empiricaldispersion=gd3bj
int=ultrafine
pop=(regular,mk)

SMILES :
CC(C)(C)OC(=O)N3C(c1ccccc1)=CC([Li])c2ccccc23.C1CCOC1.C1CCOC1.
C1CCOC1

Formula : $C_{32}H_{44}LiNO_5$

Charge : 0

Multiplicity : 1

Dipole : 13.5655 Debye

Energy : -1685.00264580 a.u.

Gibbs Energy : -1684.371815 a.u.

Number of imaginary frequencies : 0

Cartesian Coordinates (XYZ format)

83

C	0.79640700	3.17473300	-3.03340700
C	-0.50257800	3.52614200	-2.65501400
C	1.28742600	1.90432100	-2.76447000
C	0.51283700	0.94310600	-2.07861500

C	-0.77798100	1.34784100	-1.64414900
C	-1.29028600	2.59537000	-1.97526800
C	0.90631300	-0.43085100	-1.89957000
C	-0.14077500	-1.36069600	-1.69030700
C	-1.38416500	-0.99223100	-1.24206900
N	-1.50663200	0.39915800	-0.84152500
Li	1.89844300	-0.61876600	0.15061200
C	-2.56577300	-1.83439600	-1.23956000
H	1.79766100	-0.76763200	-2.41897500
H	0.01434400	-2.39298500	-1.98516600
H	1.42100700	3.89129700	-3.55640400
H	2.27902500	1.62397000	-3.10558600
H	-0.89535800	4.51149000	-2.87528400
H	-2.28916900	2.85653300	-1.65361700
C	-1.99211700	0.67940700	0.41079400
O	-1.94714600	2.00463900	0.68701000
O	-2.43515500	-0.16435200	1.17458700
C	-2.67623000	2.56699500	1.83278900
C	-2.42985600	4.06557900	1.67529200
H	-1.36083200	4.28206300	1.71572300
H	-2.92834200	4.61237700	2.47851800
H	-2.81727100	4.41797300	0.71748000
C	-2.09250100	2.06223900	3.15298300
H	-1.02449500	2.28306500	3.20787700
H	-2.24082700	0.98998500	3.25781900
H	-2.58611200	2.57356200	3.98334200
C	-4.16529600	2.24661700	1.70362700
H	-4.53864400	2.58225500	0.73301300
H	-4.72048800	2.77128200	2.48483800
H	-4.34300400	1.17733000	1.80135200
C	-2.46574600	-3.24390100	-1.20867700
C	-3.86789100	-1.28988000	-1.25774500
C	-4.99385200	-2.10300900	-1.26823200
C	-4.87432500	-3.49352400	-1.24961500
C	-3.59458200	-4.05218500	-1.21797400
H	-3.98618400	-0.21360100	-1.27478600
H	-1.48809600	-3.70853000	-1.15904300
H	-3.47500900	-5.13038200	-1.18848000
H	-5.97769400	-1.64550900	-1.28977100
H	-5.75446700	-4.12564600	-1.25048200
C	5.86626700	-1.95873200	0.58008400
C	5.95344700	-1.21931400	-0.76199100
C	4.49165700	-1.53566700	1.08630200
H	6.66333200	-1.68848600	1.27346500
H	5.89862900	-3.03989900	0.42134100
C	4.52251900	-1.32971600	-1.28103900
H	6.67748900	-1.65629700	-1.45025800
H	6.22024700	-0.17135600	-0.60107700
O	3.68305600	-1.35258800	-0.10236600
H	4.36348500	-2.25797500	-1.83794200
H	4.21709300	-0.48784700	-1.90323900
H	4.53096000	-0.58456500	1.62592000
H	3.99953600	-2.27572100	1.71679300
O	1.21796800	-1.89831000	1.43502200
C	1.16419800	-3.28588200	1.01107500
C	0.20582200	-1.65340000	2.44210400
C	0.06147800	-3.94529700	1.84342600
H	2.14777400	-3.73439600	1.16395400

H	0.93244900	-3.29588200	-0.05527700
C	-0.82196000	-2.75610100	2.24289900
H	0.48575400	-4.42328600	2.73035300
H	-0.47893000	-4.70215800	1.27363200
H	-0.20183000	-0.65872900	2.27593400
H	0.67570900	-1.69995600	3.43106200
H	-1.40794800	-2.94323600	3.14402000
H	-1.49967600	-2.47379600	1.43711800
C	1.34420400	1.98069000	1.47607000
C	1.45727700	3.29004600	0.70975500
O	2.34564500	1.11053200	0.89814100
H	0.37543100	1.50229800	1.36254100
H	1.56301800	2.10879100	2.54205600
C	2.96429900	3.36348600	0.42869000
H	1.09072900	4.13974600	1.28707200
H	0.89278700	3.22840100	-0.22036700
C	3.33567800	1.89942700	0.18786400
H	3.27433300	1.63995300	-0.87023400
H	4.32455600	1.63654500	0.56750500
H	3.49733800	3.75603700	1.29870700
H	3.20434800	3.98844400	-0.43207700

Frequencies

Mode	IR frequency	IR intensity	Raman intensity
1	20.67010000	0.47750000	0.00000000
2	25.06480000	0.63180000	0.00000000
3	30.91840000	0.85890000	0.00000000
4	35.11510000	0.39290000	0.00000000
5	38.24020000	1.95820000	0.00000000
6	42.96810000	2.15170000	0.00000000
7	46.41640000	0.14680000	0.00000000
8	52.15420000	0.53200000	0.00000000
9	55.22460000	0.82120000	0.00000000
10	58.85000000	0.14880000	0.00000000
11	63.49420000	1.83450000	0.00000000
12	65.25280000	3.37300000	0.00000000
13	68.38120000	0.16500000	0.00000000
14	73.58250000	0.55090000	0.00000000
15	77.44350000	1.46690000	0.00000000
16	80.82730000	1.78580000	0.00000000
17	82.89430000	0.32340000	0.00000000
18	85.49840000	1.11080000	0.00000000
19	95.15090000	1.52960000	0.00000000
20	102.97200000	0.60260000	0.00000000
21	106.52840000	1.13080000	0.00000000
22	112.34940000	1.09640000	0.00000000

23	120.42140000	0.18790000	0.00000000
24	131.32320000	4.87820000	0.00000000
25	142.62500000	4.43460000	0.00000000
26	145.18740000	10.55300000	0.00000000
27	153.38520000	0.44270000	0.00000000
28	156.12260000	11.58210000	0.00000000
29	164.20920000	8.60860000	0.00000000
30	181.97050000	1.84580000	0.00000000
31	210.01050000	13.98790000	0.00000000
32	213.48750000	2.04940000	0.00000000
33	218.05620000	1.00600000	0.00000000
34	243.69510000	11.94920000	0.00000000
35	257.86610000	1.65510000	0.00000000
36	258.37090000	0.65390000	0.00000000
37	260.86570000	0.25580000	0.00000000
38	265.26190000	0.82920000	0.00000000
39	274.23000000	0.44840000	0.00000000
40	278.91610000	6.07130000	0.00000000
41	312.92740000	8.79680000	0.00000000
42	329.47750000	15.47620000	0.00000000
43	347.51270000	4.06700000	0.00000000
44	355.56470000	5.21800000	0.00000000
45	387.57650000	8.19440000	0.00000000
46	414.61440000	117.21790000	0.00000000
47	415.47250000	153.44410000	0.00000000
48	418.97540000	48.86000000	0.00000000
49	424.88800000	5.47660000	0.00000000
50	444.34360000	14.31530000	0.00000000
51	458.15840000	14.11780000	0.00000000
52	464.26270000	10.32160000	0.00000000
53	476.88700000	62.51560000	0.00000000
54	480.74480000	18.90750000	0.00000000
55	510.41140000	59.22600000	0.00000000
56	515.15420000	3.63150000	0.00000000
57	558.58410000	13.16890000	0.00000000
58	561.55120000	16.47630000	0.00000000
59	580.00770000	1.41640000	0.00000000
60	584.82110000	5.19410000	0.00000000
61	585.00440000	27.81940000	0.00000000
62	585.72030000	59.22390000	0.00000000
63	604.33340000	4.35980000	0.00000000
64	636.88090000	0.22490000	0.00000000
65	680.66010000	5.77560000	0.00000000

66	688.18510000	64.79630000	0.00000000
67	688.93810000	3.68310000	0.00000000
68	692.59960000	72.27070000	0.00000000
69	695.13380000	44.68200000	0.00000000
70	705.83920000	53.10480000	0.00000000
71	722.94240000	63.48160000	0.00000000
72	744.32850000	150.15670000	0.00000000
73	746.17580000	25.52160000	0.00000000
74	757.55620000	57.64900000	0.00000000
75	766.88500000	15.47750000	0.00000000
76	772.81870000	15.19100000	0.00000000
77	800.61030000	7.63560000	0.00000000
78	839.33170000	0.72600000	0.00000000
79	842.92620000	19.73670000	0.00000000
80	846.40850000	13.93620000	0.00000000
81	851.04820000	19.15800000	0.00000000
82	851.59470000	26.22020000	0.00000000
83	854.47350000	10.40940000	0.00000000
84	877.41840000	8.29600000	0.00000000
85	877.65080000	15.24390000	0.00000000
86	878.18850000	46.73000000	0.00000000
87	884.85850000	53.42150000	0.00000000
88	890.63050000	9.01780000	0.00000000
89	891.04550000	101.90060000	0.00000000
90	892.16760000	39.23820000	0.00000000
91	898.70220000	33.84650000	0.00000000
92	916.90230000	11.45900000	0.00000000
93	917.93130000	4.01400000	0.00000000
94	921.29190000	9.96310000	0.00000000
95	923.34520000	8.10320000	0.00000000
96	925.62180000	14.28230000	0.00000000
97	926.63500000	14.40930000	0.00000000
98	928.78300000	13.97090000	0.00000000
99	929.25170000	13.11400000	0.00000000
100	930.44430000	9.29660000	0.00000000
101	930.96890000	12.68710000	0.00000000
102	935.81420000	7.12130000	0.00000000
103	962.32280000	1.32290000	0.00000000
104	968.44540000	0.30440000	0.00000000
105	969.15720000	0.14640000	0.00000000
106	970.97970000	1.87890000	0.00000000
107	972.20300000	0.63060000	0.00000000
108	978.47400000	1.73250000	0.00000000

109	981.33190000	0.55610000	0.00000000
110	1005.41740000	83.43610000	0.00000000
111	1026.46770000	70.04460000	0.00000000
112	1041.48860000	19.91660000	0.00000000
113	1045.78620000	14.93960000	0.00000000
114	1046.92970000	30.79250000	0.00000000
115	1048.55250000	11.75260000	0.00000000
116	1050.26350000	1.51610000	0.00000000
117	1053.87250000	90.69050000	0.00000000
118	1053.94550000	14.83260000	0.00000000
119	1060.14500000	154.98950000	0.00000000
120	1060.80900000	46.78180000	0.00000000
121	1061.81400000	117.19860000	0.00000000
122	1088.43600000	188.78970000	0.00000000
123	1101.10430000	15.76060000	0.00000000
124	1121.13730000	189.19410000	0.00000000
125	1134.18660000	88.53350000	0.00000000
126	1166.22170000	0.36320000	0.00000000
127	1167.97720000	1.22470000	0.00000000
128	1169.10620000	1.46220000	0.00000000
129	1171.05880000	25.19190000	0.00000000
130	1171.87280000	1.79760000	0.00000000
131	1189.84280000	15.46780000	0.00000000
132	1190.64270000	35.17410000	0.00000000
133	1192.20920000	11.19010000	0.00000000
134	1192.62350000	428.86500000	0.00000000
135	1194.57430000	0.86990000	0.00000000
136	1199.70540000	67.38930000	0.00000000
137	1207.57650000	21.48400000	0.00000000
138	1208.73270000	9.70390000	0.00000000
139	1234.99180000	8.38060000	0.00000000
140	1247.86370000	199.97340000	0.00000000
141	1257.96970000	2.77820000	0.00000000
142	1261.16980000	3.44130000	0.00000000
143	1263.96570000	9.35480000	0.00000000
144	1264.86020000	9.34940000	0.00000000
145	1266.56380000	16.57370000	0.00000000
146	1268.44410000	4.71720000	0.00000000
147	1270.85460000	5.92510000	0.00000000
148	1277.06050000	38.08760000	0.00000000
149	1287.08410000	25.49930000	0.00000000
150	1300.20160000	392.85030000	0.00000000
151	1323.43500000	0.78880000	0.00000000

152	1324.44200000	0.11270000	0.00000000
153	1325.54860000	9.19790000	0.00000000
154	1333.73880000	149.71220000	0.00000000
155	1336.91710000	88.05680000	0.00000000
156	1340.60110000	52.67480000	0.00000000
157	1344.55520000	24.06410000	0.00000000
158	1346.31550000	0.65540000	0.00000000
159	1348.25590000	11.94240000	0.00000000
160	1353.55420000	377.61100000	0.00000000
161	1359.29110000	80.55880000	0.00000000
162	1369.25000000	0.25330000	0.00000000
163	1370.89590000	7.14380000	0.00000000
164	1374.17250000	20.17060000	0.00000000
165	1392.46320000	30.38620000	0.00000000
166	1396.47900000	47.34800000	0.00000000
167	1397.93880000	1.80020000	0.00000000
168	1399.68620000	4.45600000	0.00000000
169	1402.97210000	0.85190000	0.00000000
170	1419.22750000	521.45410000	0.00000000
171	1420.74630000	30.41830000	0.00000000
172	1467.78090000	0.56910000	0.00000000
173	1473.44400000	146.00800000	0.00000000
174	1481.85870000	458.90110000	0.00000000
175	1485.40020000	16.55280000	0.00000000
176	1486.21860000	15.03630000	0.00000000
177	1486.97990000	2.84810000	0.00000000
178	1488.22540000	37.88820000	0.00000000
179	1490.15420000	25.10000000	0.00000000
180	1490.24610000	5.50180000	0.00000000
181	1495.87240000	12.32140000	0.00000000
182	1497.60170000	5.29130000	0.00000000
183	1500.18510000	7.19080000	0.00000000
184	1500.73100000	12.02820000	0.00000000
185	1501.25170000	115.75660000	0.00000000
186	1513.73990000	5.25870000	0.00000000
187	1516.94080000	2.20060000	0.00000000
188	1520.76400000	21.01760000	0.00000000
189	1522.09120000	69.62970000	0.00000000
190	1523.65720000	6.56730000	0.00000000
191	1526.24030000	6.04310000	0.00000000
192	1533.39820000	3.45410000	0.00000000
193	1533.71150000	0.15620000	0.00000000
194	1573.47100000	721.55930000	0.00000000

195	1589.00150000	79.30770000	0.00000000
196	1598.32870000	183.82130000	0.00000000
197	1634.69050000	173.44060000	0.00000000
198	1639.09120000	12.83690000	0.00000000
199	1712.68900000	648.30120000	0.00000000
200	3013.14100000	62.86710000	0.00000000
201	3015.69210000	125.02840000	0.00000000
202	3031.34060000	109.05520000	0.00000000
203	3036.85940000	22.49590000	0.00000000
204	3036.95680000	6.91700000	0.00000000
205	3039.40440000	27.67650000	0.00000000
206	3044.16130000	37.65150000	0.00000000
207	3045.39510000	39.54170000	0.00000000
208	3046.62160000	37.80750000	0.00000000
209	3046.71360000	15.68780000	0.00000000
210	3051.83390000	35.83230000	0.00000000
211	3053.64880000	73.75480000	0.00000000
212	3055.24580000	119.50140000	0.00000000
213	3072.42980000	26.92870000	0.00000000
214	3076.18690000	33.63020000	0.00000000
215	3089.14830000	31.02430000	0.00000000
216	3093.70980000	30.71940000	0.00000000
217	3096.57090000	46.17010000	0.00000000
218	3099.16310000	18.47780000	0.00000000
219	3102.40040000	28.67480000	0.00000000
220	3103.80250000	24.55490000	0.00000000
221	3105.78700000	45.99420000	0.00000000
222	3108.04070000	31.57970000	0.00000000
223	3108.65290000	46.21420000	0.00000000
224	3112.67250000	59.98980000	0.00000000
225	3115.10870000	81.12620000	0.00000000
226	3115.16030000	57.56630000	0.00000000
227	3126.85040000	20.33640000	0.00000000
228	3130.68820000	19.18810000	0.00000000
229	3137.04360000	23.46270000	0.00000000
230	3140.19620000	16.44860000	0.00000000
231	3140.65550000	10.16520000	0.00000000
232	3145.06200000	15.68390000	0.00000000
233	3148.89120000	28.93750000	0.00000000
234	3150.05480000	20.56470000	0.00000000
235	3152.40480000	7.89360000	0.00000000
236	3154.12560000	33.83190000	0.00000000
237	3159.74210000	14.15280000	0.00000000

238	3162.12890000	59.84940000	0.00000000
239	3174.16570000	51.17340000	0.00000000
240	3178.60300000	53.04110000	0.00000000
241	3180.01800000	61.58880000	0.00000000
242	3186.53270000	19.44370000	0.00000000
243	3203.63390000	19.74820000	0.00000000

Acetone

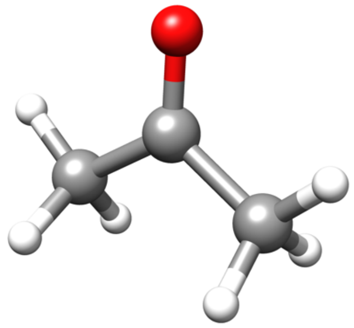

Route : # opt freq b3lyp/6-311g(d,p) scrf=(solvent=thf)
geom=connectivity empiricaldispersion=gd3bj
int=ultrafine
pop=(regular,mk)

SMILES : CC(C)=O

Formula : C_3H_6O

Charge : 0

Multiplicity : 1

Dipole : 3.4921 Debye

Energy : -193.227857154 a.u.

Gibbs Energy : -193.172559 a.u.

Number of imaginary frequencies : 0

Cartesian Coordinates (XYZ format)

10

C	-1.28627700	-0.61331900	-0.00190100
C	-0.00000100	0.18423600	0.00000300
H	-2.14041200	0.05409300	-0.10667300
H	-1.37177200	-1.17219500	0.93538100
H	-1.28197100	-1.34854300	-0.81135800
C	1.28626800	-0.61333600	0.00190200
H	1.28195700	-1.34856400	0.81135200
H	2.14040900	0.05406900	0.10667000
H	1.37175200	-1.17219900	-0.93539100
O	0.00001200	1.39848200	0.00000000

Frequencies

Mode	IR frequency	IR intensity	Raman intensity
1	86.87850000	0.02370000	0.00000000
2	136.57450000	0.11360000	0.00000000
3	381.75920000	1.70610000	0.00000000
4	492.88880000	0.54730000	0.00000000
5	535.56160000	22.68770000	0.00000000
6	788.39980000	2.05430000	0.00000000
7	883.85910000	0.08740000	0.00000000
8	892.59610000	11.19360000	0.00000000
9	1083.64400000	0.06990000	0.00000000
10	1117.28440000	6.82260000	0.00000000
11	1234.27270000	97.71540000	0.00000000
12	1378.36720000	34.43410000	0.00000000
13	1384.22850000	91.72940000	0.00000000
14	1454.82910000	2.23890000	0.00000000
15	1455.02810000	10.38500000	0.00000000
16	1460.43890000	36.31010000	0.00000000
17	1477.15980000	27.51840000	0.00000000
18	1776.40730000	279.49360000	0.00000000
19	3028.80630000	2.51590000	0.00000000
20	3035.28860000	3.56870000	0.00000000
21	3086.51530000	0.54290000	0.00000000
22	3093.06240000	22.97700000	0.00000000
23	3138.12800000	23.23620000	0.00000000
24	3139.44340000	15.85360000	0.00000000

Acetone Quenched Dihydroquinoline Above C-4 Position (Figure 6iv)

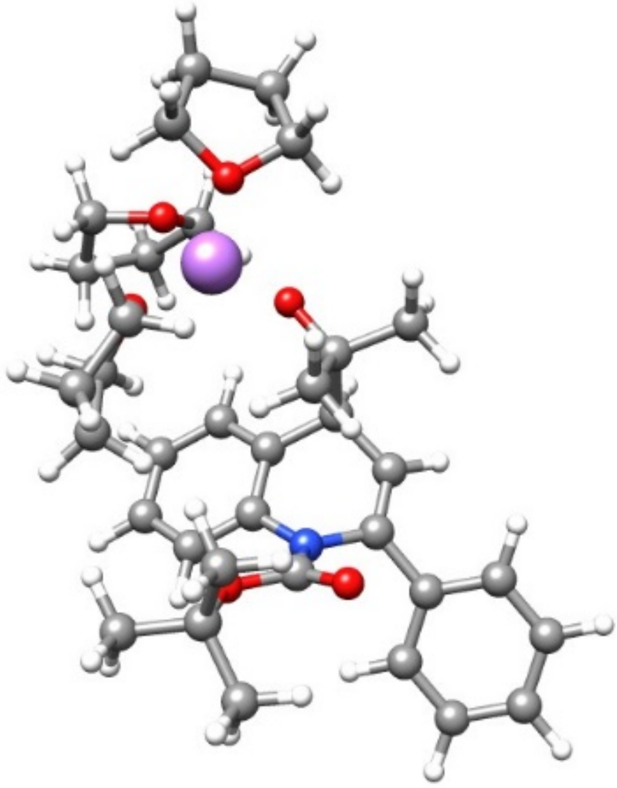

Route : # opt freq b3lyp/6-311g(d,p) scrf=(solvent=thf) geom=connectivity empiricaldispersion=gd3bj int=ultrafine pop=(regular,mk)

SMILES : CC(C)(C)OC(=O)N3C(c1ccccc1)=CC(C(C)(C)O[Li])c2ccccc23.C1CCOC1.C1CCOC1.C1CCOC1

Formula : $C_{35}H_{50}LiNO_6$

Charge : 0

Multiplicity : 1

Dipole : 10.5924 Debye

Energy : -1878.26450462 a.u.

Gibbs Energy : -1877.548551 a.u.

Number of imaginary frequencies : 0

Cartesian Coordinates (XYZ format)

93

C	0.47317500	-0.36981200	3.42403800
C	-0.55846000	-1.30302200	3.32894500
C	0.50977900	0.70749000	2.54435000
C	-0.46664600	0.87122000	1.55961000

C	-1.50324100	-0.07125500	1.48230500
C	-1.55345100	-1.14884300	2.36875800
C	-0.45632900	2.05709200	0.62970400
C	-1.87224600	2.45031900	0.34016000
C	-2.85737000	1.54847000	0.26048700
N	-2.54102700	0.17139900	0.52069600
Li	2.92369500	0.29762400	-0.51717200
C	-4.28100100	1.89110700	0.07246500
H	0.04578000	2.88374800	1.13854400
H	-2.11970100	3.50073800	0.25437500
H	1.24080800	-0.48007500	4.18132000
H	1.30196600	1.44146400	2.60489300
H	-0.60306200	-2.14177600	4.01381800
H	-2.36689000	-1.85542800	2.30300100
C	-2.98705600	-0.75574500	-0.41172700
O	-2.68206700	-2.01371100	-0.05860100
O	-3.59553700	-0.43634300	-1.41327700
C	-3.14076200	-3.15520300	-0.88336600
C	-2.64557900	-4.36224900	-0.09224200
H	-1.55908200	-4.35810400	-0.01348400
H	-2.95403400	-5.28060600	-0.59576700
H	-3.06879500	-4.35900200	0.91424500
C	-2.48323200	-3.09513200	-2.26054800
H	-1.39963900	-3.00877200	-2.15834100
H	-2.85137600	-2.24693700	-2.83354300
H	-2.70387900	-4.01546000	-2.80653100
C	-4.66694900	-3.16179600	-0.96027200
H	-5.09359400	-3.14116600	0.04553100
H	-4.99563700	-4.08047900	-1.45180600
H	-5.03835500	-2.30761600	-1.52115000
C	-4.65833300	2.94788500	-0.76527200
C	-5.28123300	1.19204600	0.76144400
C	-6.61811500	1.55050100	0.62831300
C	-6.98299800	2.61195600	-0.19923800
C	-5.99594100	3.30815000	-0.89533700
H	-5.00278500	0.36889800	1.40843700
H	-3.89883700	3.47226000	-1.33249500
H	-6.26963400	4.12548000	-1.55287800
H	-7.37769400	1.00097100	1.17278700
H	-8.02544800	2.88805600	-0.30652700
C	6.20498500	0.95228400	-1.81458700
C	5.35735200	2.23814000	-1.99203200
C	5.25093000	-0.18467400	-2.23162600
H	6.51459200	0.83808700	-0.77678300
H	7.09799500	0.95958000	-2.44142900
C	4.08629600	1.74485900	-2.68847600
H	5.86960400	3.00488200	-2.57485400
H	5.10278000	2.66075300	-1.01979900
O	3.93233600	0.38577300	-2.24299800
H	4.19295500	1.75673500	-3.78036800
H	3.18359000	2.27442200	-2.39189300
H	5.23357400	-1.02494300	-1.53884800
H	5.48798400	-0.55475600	-3.23583900
O	2.61846800	-1.64691700	-0.47396600
C	2.25559100	-2.40746100	-1.65190800
C	1.96086500	-2.19493200	0.69382200
C	1.49568800	-3.62971900	-1.13826700
H	3.16826400	-2.65705200	-2.19461300

H	1.62737300	-1.77742300	-2.28638700
C	0.86305600	-3.10013900	0.15529400
H	2.18835300	-4.44548800	-0.91416500
H	0.76491800	-3.99319400	-1.86185500
H	1.58723900	-1.36736100	1.29124600
H	2.69559100	-2.76084900	1.27726200
H	0.59926900	-3.88793600	0.86166400
H	-0.03506000	-2.51724800	-0.05532300
C	5.00496500	-0.54709200	1.56222300
C	4.40894500	-0.43655800	2.96120800
O	4.36270200	0.50007400	0.82653400
H	4.79193000	-1.48374800	1.04921400
H	6.09026600	-0.38041400	1.57516500
C	4.27832600	1.08964000	3.14642300
H	5.03210100	-0.90542500	3.72410400
H	3.42615000	-0.91038500	2.97845200
C	4.21686200	1.64111600	1.70455200
H	3.26734200	2.10014700	1.43156700
H	5.03525800	2.34160300	1.50985700
H	5.14764000	1.48925100	3.67191900
H	3.39231700	1.35501000	3.72394100
C	0.46889800	1.83061800	-0.66687100
C	0.34593800	3.11104500	-1.52403800
C	-0.06146900	0.63548300	-1.48913600
O	1.75628600	1.63198200	-0.26525700
H	0.69062600	3.97566100	-0.94857100
H	-0.67674000	3.30043300	-1.86629200
H	0.98931200	3.01186300	-2.40278400
H	-1.09506400	0.76259700	-1.81968100
H	0.01118300	-0.28203300	-0.90211900
H	0.56971900	0.51678200	-2.37499300

Frequencies

Mode	IR frequency	IR intensity	Raman intensity
1	18.41870000	0.62410000	0.00000000
2	25.96950000	1.89490000	0.00000000
3	30.91940000	0.86680000	0.00000000
4	35.52530000	0.64810000	0.00000000
5	41.76810000	2.71200000	0.00000000
6	45.03070000	1.44810000	0.00000000
7	51.25530000	0.01210000	0.00000000
8	54.75510000	0.57340000	0.00000000
9	56.00850000	1.28300000	0.00000000
10	57.35290000	0.89090000	0.00000000
11	58.31760000	1.19570000	0.00000000
12	64.33460000	0.17990000	0.00000000
13	67.72530000	0.60820000	0.00000000
14	71.20270000	1.22280000	0.00000000
15	73.50810000	1.54760000	0.00000000

16	79.07430000	1.96840000	0.00000000
17	82.86030000	1.88610000	0.00000000
18	85.04700000	0.23670000	0.00000000
19	95.40330000	0.83510000	0.00000000
20	99.11270000	2.89430000	0.00000000
21	101.38550000	0.87710000	0.00000000
22	102.67500000	3.99570000	0.00000000
23	107.84000000	4.52450000	0.00000000
24	111.28990000	1.89440000	0.00000000
25	114.60310000	2.36660000	0.00000000
26	127.02630000	0.23250000	0.00000000
27	130.93710000	1.69540000	0.00000000
28	133.25810000	4.80450000	0.00000000
29	137.01590000	2.55070000	0.00000000
30	150.07400000	8.49500000	0.00000000
31	157.20650000	2.02760000	0.00000000
32	171.44400000	17.37540000	0.00000000
33	181.42250000	1.15060000	0.00000000
34	204.94860000	0.56900000	0.00000000
35	210.38600000	1.14050000	0.00000000
36	226.30560000	1.74940000	0.00000000
37	241.51240000	1.34720000	0.00000000
38	248.10620000	2.39100000	0.00000000
39	260.63090000	5.15510000	0.00000000
40	265.74910000	1.22790000	0.00000000
41	269.55060000	0.94900000	0.00000000
42	275.45690000	5.09980000	0.00000000
43	287.30420000	5.31020000	0.00000000
44	287.59350000	3.01310000	0.00000000
45	290.41390000	5.11160000	0.00000000
46	303.77900000	2.71740000	0.00000000
47	317.03230000	0.70270000	0.00000000
48	330.51510000	9.53420000	0.00000000
49	333.83570000	5.32350000	0.00000000
50	356.40530000	3.35390000	0.00000000
51	364.74080000	1.82670000	0.00000000
52	368.04890000	9.49150000	0.00000000
53	404.26960000	5.63100000	0.00000000
54	411.42160000	116.83700000	0.00000000
55	418.43330000	0.34100000	0.00000000
56	422.54680000	101.72000000	0.00000000
57	427.03970000	19.64890000	0.00000000
58	446.04320000	13.99730000	0.00000000

59	450.68820000	5.89160000	0.00000000
60	458.91410000	2.62000000	0.00000000
61	477.22450000	29.78790000	0.00000000
62	480.76680000	6.23020000	0.00000000
63	492.90080000	7.47470000	0.00000000
64	531.88920000	7.60720000	0.00000000
65	549.30320000	21.56700000	0.00000000
66	571.22090000	10.67240000	0.00000000
67	580.27560000	26.39160000	0.00000000
68	585.28740000	2.59950000	0.00000000
69	604.29490000	5.41770000	0.00000000
70	625.32640000	7.39460000	0.00000000
71	628.16800000	8.71690000	0.00000000
72	629.29230000	14.57100000	0.00000000
73	633.79530000	122.06990000	0.00000000
74	639.92180000	5.80040000	0.00000000
75	671.96770000	13.87820000	0.00000000
76	689.81800000	8.71680000	0.00000000
77	694.97340000	17.02410000	0.00000000
78	699.14290000	11.09410000	0.00000000
79	710.65470000	41.10040000	0.00000000
80	712.80100000	22.50130000	0.00000000
81	728.39360000	17.52600000	0.00000000
82	758.70600000	18.87940000	0.00000000
83	765.24810000	55.30630000	0.00000000
84	772.00020000	80.80600000	0.00000000
85	782.54660000	70.02160000	0.00000000
86	792.49120000	14.30660000	0.00000000
87	797.97610000	11.03310000	0.00000000
88	813.89050000	2.13800000	0.00000000
89	815.49490000	8.81260000	0.00000000
90	842.47230000	158.96640000	0.00000000
91	851.10280000	34.65920000	0.00000000
92	852.34660000	15.76860000	0.00000000
93	857.55990000	1.25760000	0.00000000
94	865.71670000	11.96370000	0.00000000
95	872.63620000	26.16620000	0.00000000
96	876.86610000	2.94530000	0.00000000
97	880.60500000	14.36350000	0.00000000
98	881.40550000	11.63020000	0.00000000
99	892.93810000	29.11140000	0.00000000
100	896.65000000	72.08880000	0.00000000
101	907.93020000	13.96460000	0.00000000

102	909.92510000	11.63820000	0.00000000
103	912.24970000	91.22060000	0.00000000
104	918.20150000	57.32390000	0.00000000
105	919.84010000	35.29730000	0.00000000
106	926.87790000	55.85200000	0.00000000
107	928.18710000	0.66270000	0.00000000
108	929.26280000	24.29530000	0.00000000
109	930.72540000	10.10200000	0.00000000
110	932.18420000	3.86210000	0.00000000
111	935.77410000	18.96680000	0.00000000
112	936.67800000	2.64810000	0.00000000
113	945.37590000	4.48150000	0.00000000
114	955.95500000	3.84580000	0.00000000
115	964.99370000	4.16060000	0.00000000
116	971.10200000	0.25490000	0.00000000
117	976.59630000	2.61370000	0.00000000
118	978.79410000	2.16490000	0.00000000
119	985.63160000	0.13200000	0.00000000
120	987.88760000	0.25110000	0.00000000
121	996.01940000	1.20420000	0.00000000
122	1002.79420000	0.19870000	0.00000000
123	1016.71190000	11.43910000	0.00000000
124	1021.06950000	100.56940000	0.00000000
125	1037.50870000	25.45200000	0.00000000
126	1043.83180000	6.46730000	0.00000000
127	1045.89280000	20.77620000	0.00000000
128	1048.76140000	54.96140000	0.00000000
129	1052.80950000	1.88430000	0.00000000
130	1053.91640000	24.04020000	0.00000000
131	1056.33020000	12.68280000	0.00000000
132	1062.17180000	112.52600000	0.00000000
133	1063.02370000	55.27970000	0.00000000
134	1068.87370000	41.21100000	0.00000000
135	1070.83220000	172.50750000	0.00000000
136	1072.62310000	22.81660000	0.00000000
137	1103.51440000	5.44960000	0.00000000
138	1123.49380000	16.51150000	0.00000000
139	1137.37600000	161.07280000	0.00000000
140	1146.07500000	8.67380000	0.00000000
141	1147.32210000	150.98890000	0.00000000
142	1158.16550000	1.41140000	0.00000000
143	1169.61360000	110.18150000	0.00000000
144	1170.71950000	83.25410000	0.00000000

145	1176.79440000	1.01860000	0.00000000
146	1178.49380000	2.65140000	0.00000000
147	1187.20530000	382.15320000	0.00000000
148	1192.97810000	43.99920000	0.00000000
149	1198.37260000	6.57630000	0.00000000
150	1202.42120000	11.50070000	0.00000000
151	1204.54410000	9.11620000	0.00000000
152	1214.35300000	100.23820000	0.00000000
153	1217.24750000	10.16440000	0.00000000
154	1230.77640000	127.40260000	0.00000000
155	1235.90880000	3.10210000	0.00000000
156	1238.22900000	14.23510000	0.00000000
157	1254.69040000	2.46870000	0.00000000
158	1257.62580000	67.27440000	0.00000000
159	1257.88610000	6.63650000	0.00000000
160	1261.80690000	2.74060000	0.00000000
161	1269.23200000	30.26520000	0.00000000
162	1275.03810000	61.78440000	0.00000000
163	1276.40680000	53.96850000	0.00000000
164	1279.36780000	3.16090000	0.00000000
165	1280.21400000	41.13460000	0.00000000
166	1286.02640000	2.94840000	0.00000000
167	1288.43530000	125.79460000	0.00000000
168	1294.70750000	138.39240000	0.00000000
169	1313.07220000	2.52430000	0.00000000
170	1317.31090000	43.64660000	0.00000000
171	1317.93370000	6.62600000	0.00000000
172	1326.40810000	3.74390000	0.00000000
173	1327.46410000	53.77000000	0.00000000
174	1328.20480000	3.17340000	0.00000000
175	1334.22960000	0.53800000	0.00000000
176	1346.63000000	349.56070000	0.00000000
177	1350.13110000	62.65160000	0.00000000
178	1352.02930000	10.55890000	0.00000000
179	1357.43080000	57.39190000	0.00000000
180	1362.91350000	0.31490000	0.00000000
181	1369.04850000	36.00230000	0.00000000
182	1369.83900000	0.35130000	0.00000000
183	1374.29230000	5.11540000	0.00000000
184	1379.43440000	4.52930000	0.00000000
185	1386.24260000	17.63480000	0.00000000
186	1396.24300000	25.60020000	0.00000000
187	1396.60160000	5.48290000	0.00000000

188	1399.43840000	38.80530000	0.00000000
189	1405.08330000	9.94750000	0.00000000
190	1406.30250000	1.34850000	0.00000000
191	1423.35720000	27.05820000	0.00000000
192	1467.04540000	0.05490000	0.00000000
193	1477.26300000	4.85130000	0.00000000
194	1477.41350000	4.06030000	0.00000000
195	1482.15040000	3.11630000	0.00000000
196	1482.51520000	1.83560000	0.00000000
197	1485.29180000	1.65370000	0.00000000
198	1486.58990000	1.73150000	0.00000000
199	1488.28320000	41.57400000	0.00000000
200	1488.79100000	4.94820000	0.00000000
201	1490.08240000	9.49790000	0.00000000
202	1491.05840000	7.94000000	0.00000000
203	1493.86010000	5.26500000	0.00000000
204	1495.75870000	7.23070000	0.00000000
205	1500.38060000	5.23580000	0.00000000
206	1501.47660000	8.36480000	0.00000000
207	1502.57400000	11.38900000	0.00000000
208	1502.66320000	18.93550000	0.00000000
209	1503.45690000	4.72390000	0.00000000
210	1513.39770000	38.67140000	0.00000000
211	1515.16720000	55.23460000	0.00000000
212	1518.35050000	4.01000000	0.00000000
213	1520.77540000	24.70670000	0.00000000
214	1521.83020000	2.66580000	0.00000000
215	1526.14910000	38.47570000	0.00000000
216	1531.00840000	7.87410000	0.00000000
217	1533.36580000	1.97330000	0.00000000
218	1615.11690000	17.33400000	0.00000000
219	1617.55290000	14.44510000	0.00000000
220	1640.74390000	6.06330000	0.00000000
221	1643.54430000	35.39750000	0.00000000
222	1702.56970000	44.49340000	0.00000000
223	1735.51270000	587.04500000	0.00000000
224	2992.03320000	101.99530000	0.00000000
225	3000.44170000	80.79320000	0.00000000
226	3008.15170000	65.48370000	0.00000000
227	3010.04050000	108.97590000	0.00000000
228	3013.99100000	96.07670000	0.00000000
229	3020.27800000	100.33940000	0.00000000
230	3023.08100000	41.35840000	0.00000000

231	3040.06010000	26.33360000	0.00000000
232	3043.64960000	17.61570000	0.00000000
233	3044.95850000	35.92480000	0.00000000
234	3047.36920000	58.27410000	0.00000000
235	3047.91040000	51.31590000	0.00000000
236	3050.44680000	30.51730000	0.00000000
237	3059.75980000	55.22860000	0.00000000
238	3063.76460000	42.92290000	0.00000000
239	3066.10270000	32.42470000	0.00000000
240	3068.11030000	17.33250000	0.00000000
241	3072.50770000	57.76690000	0.00000000
242	3074.02500000	64.45260000	0.00000000
243	3080.91970000	57.46020000	0.00000000
244	3085.72040000	122.09850000	0.00000000
245	3092.46010000	36.03900000	0.00000000
246	3095.68590000	13.16050000	0.00000000
247	3100.58160000	17.71410000	0.00000000
248	3101.86710000	16.88420000	0.00000000
249	3104.50420000	54.31240000	0.00000000
250	3106.77510000	17.54110000	0.00000000
251	3107.06110000	58.33700000	0.00000000
252	3110.45870000	41.45930000	0.00000000
253	3112.47390000	11.61900000	0.00000000
254	3113.46940000	69.90300000	0.00000000
255	3116.58540000	30.69290000	0.00000000
256	3117.37310000	32.83170000	0.00000000
257	3121.60400000	46.37860000	0.00000000
258	3125.01460000	85.38940000	0.00000000
259	3137.04600000	26.44610000	0.00000000
260	3137.24300000	7.60670000	0.00000000
261	3146.01030000	3.61900000	0.00000000
262	3146.99230000	7.65530000	0.00000000
263	3152.75860000	15.46000000	0.00000000
264	3161.62020000	4.86480000	0.00000000
265	3165.64380000	7.27400000	0.00000000
266	3168.62370000	8.01790000	0.00000000
267	3179.19930000	35.44520000	0.00000000
268	3181.61260000	36.03310000	0.00000000
269	3184.16710000	22.96920000	0.00000000
270	3186.93150000	28.00280000	0.00000000
271	3191.90290000	25.57260000	0.00000000
272	3204.11720000	16.47070000	0.00000000
273	3226.94880000	6.07230000	0.00000000

Acetone Quenched Dihydroquinoline Below C-4 Position (Figure 6iii)

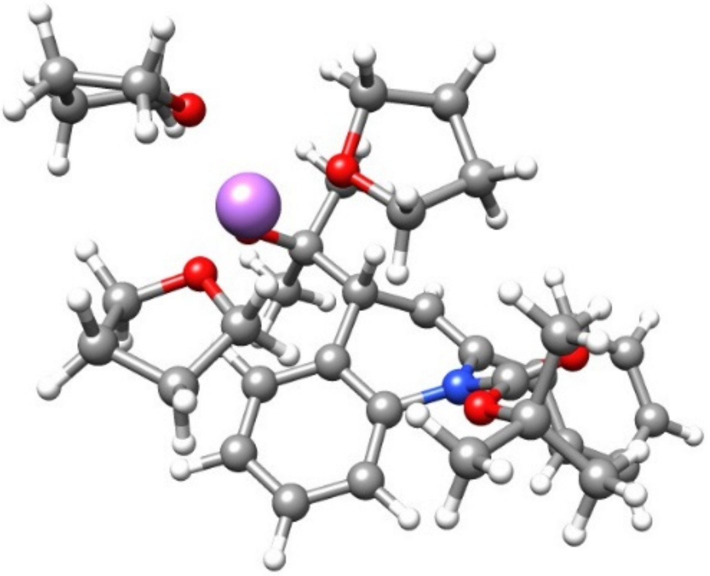

Route : # opt freq b3lyp/6-311g(d,p) scrf=(solvent=thf)
geom=connectivity empiricaldispersion=gd3bj
int=ultrafine
pop=(regular,mk)

SMILES :
CC(C)(C)OC(=O)N3C(c1ccccc1)=CC(C(C)(C)O[Li])c2ccccc23.C1CCOC1.
C1CCOC1.C1CCOC1

Formula : $C_{35}H_{50}LiNO_6$

Charge : 0

Multiplicity : 1

Dipole : 10.3747 Debye

Energy : -1878.25709953 a.u.

Gibbs Energy : -1877.542996 a.u.

Number of imaginary frequencies : 0

Cartesian Coordinates (XYZ format)

93

C	-0.68616000	1.01155600	3.36291400
C	0.42331500	1.85047700	3.26102600
C	-0.81492600	-0.09094600	2.52377600
C	0.16422300	-0.38111200	1.56843100
C	1.26277300	0.48214500	1.47002200
C	1.40543400	1.58231200	2.31508800
C	0.14368700	-1.57214800	0.61286600
C	1.55651700	-2.10074200	0.51717900

C	2.58823700	-1.25523600	0.41385000
N	2.27617700	0.14365500	0.51053200
Li	-2.65360300	-0.60343400	-0.24717900
C	4.00962300	-1.64582700	0.36978500
H	1.74339000	-3.16345800	0.58839500
H	-1.45708900	1.22019200	4.09612500
H	-1.68826500	-0.72526600	2.55484100
H	0.52779200	2.70649800	3.91753700
H	2.27394200	2.21904500	2.22133300
C	2.72097900	1.00238700	-0.47938500
O	2.17465300	2.22088700	-0.33643600
O	3.48852800	0.66770200	-1.35943600
C	2.61515800	3.36907700	-1.15441000
C	1.77641900	4.50950600	-0.58471300
H	0.71235600	4.29106600	-0.69631300
H	2.00070300	5.43687900	-1.11550100
H	1.99199300	4.65156400	0.47592500
C	2.28249900	3.13367500	-2.62604400
H	1.21328000	2.95210200	-2.74936800
H	2.83733000	2.28622700	-3.02206200
H	2.54222700	4.02719800	-3.19869100
C	4.10506800	3.61605100	-0.92714600
H	4.31053400	3.72798700	0.14009600
H	4.39966800	4.53931600	-1.43124800
H	4.70299100	2.79575400	-1.32028900
C	4.40661800	-2.82225800	-0.27801200
C	4.98472000	-0.86934100	1.00925200
C	6.31662500	-1.26787500	1.01526000
C	6.70083000	-2.44730400	0.37789700
C	5.73937200	-3.22187600	-0.26918900
H	4.68987300	0.04554200	1.50838200
H	3.66824200	-3.41243000	-0.80742700
H	6.02967300	-4.13296200	-0.77979300
H	7.05734300	-0.65729800	1.51896000
H	7.73994500	-2.75491300	0.37857900
C	-6.67312200	-0.47969800	-0.69540900
C	-6.22515000	-1.65004300	0.20404000
C	-5.38978100	-0.08827300	-1.46353900
H	-7.07727900	0.35485700	-0.12081200
H	-7.44698000	-0.80839400	-1.39180600
C	-5.03732900	-2.21887600	-0.56189800
H	-7.01564100	-2.38454300	0.36478500
H	-5.89136800	-1.28826100	1.17961500
O	-4.38365500	-1.05208800	-1.09708800
H	-5.36527300	-2.86550700	-1.38626100
H	-4.28876200	-2.72453700	0.04703100
H	-5.00149100	0.89305900	-1.18969700
H	-5.54199600	-0.11888100	-2.54679800
O	-1.63642600	-0.10696800	-1.91761100
C	-1.84771600	-0.90025800	-3.09734300
C	-0.47901800	0.69967300	-2.18270600
C	-0.44387000	-1.31600500	-3.54901900
H	-2.34571600	-0.28463700	-3.85682800
H	-2.50531400	-1.72168600	-2.82201700
C	0.47168200	-0.18848800	-3.00616500
H	-0.38543500	-1.41844900	-4.63325200
H	-0.17007400	-2.27397500	-3.10575100
H	-0.07099200	1.02260500	-1.22821700

H	-0.78729000	1.58556700	-2.75070400
H	0.94193300	0.38334400	-3.80609000
H	1.26796500	-0.59580900	-2.38446500
C	-2.13910900	2.35598700	0.39255800
C	-2.66415000	3.38825400	1.39918800
O	-3.01367000	1.20487100	0.49416000
H	-1.11778500	2.04536200	0.61480400
H	-2.19163600	2.71317900	-0.63810600
C	-4.09880900	2.91297100	1.67617000
H	-2.62090100	4.40399500	1.00460800
H	-2.07167000	3.35098000	2.31466000
C	-3.94871500	1.40157400	1.56927600
H	-3.54500900	0.97069700	2.49105900
H	-4.86744800	0.87779300	1.30750800
H	-4.78401400	3.27718200	0.90582500
H	-4.47347900	3.22920200	2.65062400
H	-0.10363100	-1.15863600	-0.37291600
C	-0.97967600	-2.64010500	0.85232300
C	-0.88156900	-3.68938100	-0.28522100
C	-0.75640300	-3.37716900	2.19466600
O	-2.21218900	-2.02432800	0.80314200
H	0.06155600	-4.24577900	-0.30905600
H	-1.01563200	-3.19340800	-1.24977600
H	-1.69620700	-4.40736100	-0.16034400
H	-0.79610900	-2.68589900	3.03774700
H	0.20379900	-3.90309900	2.23296300
H	-1.55406800	-4.11399400	2.32392100

Frequencies

Mode	IR frequency	IR intensity	Raman intensity
1	14.85980000	0.15500000	0.00000000
2	21.64360000	1.43220000	0.00000000
3	26.80620000	0.27680000	0.00000000
4	30.85770000	0.66720000	0.00000000
5	36.77610000	1.22540000	0.00000000
6	46.03060000	0.30480000	0.00000000
7	48.99160000	0.19530000	0.00000000
8	52.34270000	4.51260000	0.00000000
9	53.27550000	2.05040000	0.00000000
10	55.37830000	0.11940000	0.00000000
11	58.07050000	0.55370000	0.00000000
12	60.90030000	1.57340000	0.00000000
13	67.50130000	1.22470000	0.00000000
14	72.93320000	1.28390000	0.00000000
15	74.36710000	0.94420000	0.00000000
16	77.65460000	0.66390000	0.00000000
17	83.52580000	1.07240000	0.00000000
18	84.67810000	1.09630000	0.00000000

19	88.29440000	1.68510000	0.00000000
20	98.01410000	0.49520000	0.00000000
21	99.14880000	2.79860000	0.00000000
22	105.68520000	2.15150000	0.00000000
23	110.61220000	2.12780000	0.00000000
24	117.61480000	2.50640000	0.00000000
25	121.48870000	1.17550000	0.00000000
26	122.88840000	1.95060000	0.00000000
27	128.48790000	0.73250000	0.00000000
28	134.95290000	3.52620000	0.00000000
29	146.81010000	5.56730000	0.00000000
30	148.97850000	2.48100000	0.00000000
31	160.99310000	10.17570000	0.00000000
32	170.39620000	0.77050000	0.00000000
33	172.21240000	6.69420000	0.00000000
34	197.43060000	1.65060000	0.00000000
35	205.54130000	0.49080000	0.00000000
36	208.41860000	1.61860000	0.00000000
37	218.31450000	1.38510000	0.00000000
38	234.76830000	7.92800000	0.00000000
39	255.68120000	0.92160000	0.00000000
40	256.39140000	4.73780000	0.00000000
41	265.79200000	0.96810000	0.00000000
42	268.27420000	0.32380000	0.00000000
43	271.38140000	4.32470000	0.00000000
44	280.57460000	2.39790000	0.00000000
45	286.97170000	16.98380000	0.00000000
46	292.24110000	9.09270000	0.00000000
47	297.20630000	3.90060000	0.00000000
48	325.59960000	9.27650000	0.00000000
49	333.50880000	8.47210000	0.00000000
50	344.00780000	5.13500000	0.00000000
51	353.26240000	5.87220000	0.00000000
52	364.36360000	3.21040000	0.00000000
53	385.89330000	88.89780000	0.00000000
54	400.83060000	26.03590000	0.00000000
55	415.89720000	142.76320000	0.00000000
56	417.83100000	1.84330000	0.00000000
57	427.26020000	17.81650000	0.00000000
58	445.88700000	2.29440000	0.00000000
59	455.67210000	12.78060000	0.00000000
60	458.90080000	6.07780000	0.00000000
61	467.33880000	15.58860000	0.00000000

62	482.41700000	23.12620000	0.00000000
63	486.39920000	0.58450000	0.00000000
64	523.08570000	13.88190000	0.00000000
65	545.54950000	6.41670000	0.00000000
66	564.08750000	52.86610000	0.00000000
67	587.47030000	10.20200000	0.00000000
68	590.71210000	27.44250000	0.00000000
69	605.47430000	12.37530000	0.00000000
70	610.18970000	45.82590000	0.00000000
71	631.20420000	2.73890000	0.00000000
72	640.56360000	6.43100000	0.00000000
73	642.30830000	8.53520000	0.00000000
74	652.86740000	38.63370000	0.00000000
75	666.38600000	5.25980000	0.00000000
76	668.83650000	10.33580000	0.00000000
77	699.63340000	23.87680000	0.00000000
78	700.04060000	28.33220000	0.00000000
79	700.80340000	6.81410000	0.00000000
80	710.92830000	43.89100000	0.00000000
81	745.71130000	21.64540000	0.00000000
82	758.95950000	18.34580000	0.00000000
83	769.95300000	99.60650000	0.00000000
84	773.29800000	20.95450000	0.00000000
85	777.43100000	42.06330000	0.00000000
86	791.61990000	31.97860000	0.00000000
87	805.71030000	13.55860000	0.00000000
88	814.96570000	0.73890000	0.00000000
89	817.90340000	6.16200000	0.00000000
90	840.09220000	8.71670000	0.00000000
91	840.39560000	8.26240000	0.00000000
92	850.92220000	23.08340000	0.00000000
93	857.55960000	0.49570000	0.00000000
94	866.67490000	12.19260000	0.00000000
95	877.15070000	16.26810000	0.00000000
96	878.52010000	26.14690000	0.00000000
97	881.16420000	14.41040000	0.00000000
98	887.80010000	1.93490000	0.00000000
99	896.99270000	15.14800000	0.00000000
100	901.59930000	54.48710000	0.00000000
101	910.32170000	6.52680000	0.00000000
102	911.61490000	43.57110000	0.00000000
103	912.12960000	9.53110000	0.00000000
104	919.56550000	98.42990000	0.00000000

105	920.09580000	68.10630000	0.00000000
106	928.42600000	0.84560000	0.00000000
107	929.30220000	5.05180000	0.00000000
108	931.56080000	43.23150000	0.00000000
109	931.84800000	8.83020000	0.00000000
110	932.93190000	11.49000000	0.00000000
111	934.99590000	2.72560000	0.00000000
112	946.72970000	4.19260000	0.00000000
113	965.59280000	0.91360000	0.00000000
114	966.81330000	38.65010000	0.00000000
115	971.79460000	0.14380000	0.00000000
116	973.63610000	9.14560000	0.00000000
117	975.70520000	1.09790000	0.00000000
118	979.45920000	1.29990000	0.00000000
119	986.04970000	0.04560000	0.00000000
120	996.12950000	3.60900000	0.00000000
121	1003.81750000	0.28070000	0.00000000
122	1005.08400000	17.38400000	0.00000000
123	1017.96620000	2.42000000	0.00000000
124	1018.66150000	70.82320000	0.00000000
125	1037.69520000	20.16540000	0.00000000
126	1042.13480000	14.76800000	0.00000000
127	1045.31380000	9.06990000	0.00000000
128	1052.78110000	11.87410000	0.00000000
129	1053.40330000	4.47490000	0.00000000
130	1057.32610000	12.67110000	0.00000000
131	1058.58200000	17.27040000	0.00000000
132	1067.16620000	107.19670000	0.00000000
133	1067.47930000	79.56470000	0.00000000
134	1068.64920000	99.69530000	0.00000000
135	1072.79090000	168.75290000	0.00000000
136	1085.91790000	32.51290000	0.00000000
137	1104.25160000	6.80260000	0.00000000
138	1125.37960000	28.25090000	0.00000000
139	1136.07070000	299.89880000	0.00000000
140	1147.28440000	36.13150000	0.00000000
141	1148.19510000	19.75940000	0.00000000
142	1162.86520000	1.59970000	0.00000000
143	1164.46950000	1.54570000	0.00000000
144	1174.75400000	33.38030000	0.00000000
145	1177.48380000	0.54210000	0.00000000
146	1185.80170000	320.68560000	0.00000000
147	1190.01760000	3.86180000	0.00000000

148	1194.66270000	166.56850000	0.00000000
149	1198.81180000	5.34540000	0.00000000
150	1200.41350000	11.03880000	0.00000000
151	1204.35900000	67.04230000	0.00000000
152	1211.63660000	5.47600000	0.00000000
153	1216.95050000	36.27650000	0.00000000
154	1219.91160000	6.60620000	0.00000000
155	1236.18520000	2.30520000	0.00000000
156	1237.66690000	4.69820000	0.00000000
157	1246.48080000	4.55020000	0.00000000
158	1254.52960000	0.13470000	0.00000000
159	1266.66880000	14.07510000	0.00000000
160	1269.45320000	10.78550000	0.00000000
161	1269.76990000	22.15960000	0.00000000
162	1274.59630000	188.16630000	0.00000000
163	1275.08420000	5.74860000	0.00000000
164	1279.36430000	38.43530000	0.00000000
165	1281.11880000	19.57270000	0.00000000
166	1285.05850000	11.02650000	0.00000000
167	1287.13310000	6.03610000	0.00000000
168	1300.87830000	59.79830000	0.00000000
169	1312.35570000	103.19940000	0.00000000
170	1318.66440000	2.70090000	0.00000000
171	1319.10420000	3.42550000	0.00000000
172	1322.19160000	27.97990000	0.00000000
173	1324.20910000	1.72310000	0.00000000
174	1331.23530000	9.86830000	0.00000000
175	1332.15800000	4.87700000	0.00000000
176	1337.70320000	20.31430000	0.00000000
177	1341.35790000	10.66040000	0.00000000
178	1346.04010000	266.00130000	0.00000000
179	1351.34110000	161.29450000	0.00000000
180	1359.29940000	160.32720000	0.00000000
181	1365.10790000	0.87390000	0.00000000
182	1366.39960000	1.51410000	0.00000000
183	1369.12220000	8.52330000	0.00000000
184	1373.38950000	17.30290000	0.00000000
185	1391.97920000	20.66130000	0.00000000
186	1395.88800000	23.09380000	0.00000000
187	1397.84150000	10.34170000	0.00000000
188	1398.89910000	41.35610000	0.00000000
189	1400.16050000	2.52740000	0.00000000
190	1402.23970000	0.39870000	0.00000000

191	1422.53230000	23.84790000	0.00000000
192	1467.41920000	0.53550000	0.00000000
193	1473.64880000	0.26380000	0.00000000
194	1476.53040000	0.76080000	0.00000000
195	1477.94600000	9.64470000	0.00000000
196	1484.48180000	8.99870000	0.00000000
197	1485.10720000	13.12270000	0.00000000
198	1485.48820000	3.49410000	0.00000000
199	1485.78720000	4.63400000	0.00000000
200	1486.03120000	0.30040000	0.00000000
201	1488.99070000	6.01080000	0.00000000
202	1490.68020000	11.93600000	0.00000000
203	1491.24840000	22.35000000	0.00000000
204	1496.81660000	9.69870000	0.00000000
205	1497.18280000	5.47140000	0.00000000
206	1499.94810000	1.46620000	0.00000000
207	1502.35100000	7.71400000	0.00000000
208	1507.39910000	71.79740000	0.00000000
209	1507.56620000	22.96150000	0.00000000
210	1513.62750000	1.41910000	0.00000000
211	1515.75680000	1.58260000	0.00000000
212	1520.56470000	30.88880000	0.00000000
213	1522.34160000	1.91310000	0.00000000
214	1526.32250000	25.41710000	0.00000000
215	1526.58420000	6.41750000	0.00000000
216	1531.20390000	2.60460000	0.00000000
217	1533.66230000	5.86630000	0.00000000
218	1612.61900000	19.67670000	0.00000000
219	1617.39610000	4.09330000	0.00000000
220	1640.47440000	4.09220000	0.00000000
221	1644.42870000	16.57750000	0.00000000
222	1691.96150000	23.04690000	0.00000000
223	1739.12280000	684.39180000	0.00000000
224	2990.95890000	105.11520000	0.00000000
225	3001.71030000	67.25620000	0.00000000
226	3005.99030000	103.24510000	0.00000000
227	3011.00710000	43.32910000	0.00000000
228	3014.10080000	29.53890000	0.00000000
229	3018.46710000	63.37340000	0.00000000
230	3026.73960000	86.41570000	0.00000000
231	3032.65200000	60.24570000	0.00000000
232	3038.03560000	16.90640000	0.00000000
233	3041.31120000	28.83630000	0.00000000

234	3046.40520000	43.73390000	0.00000000
235	3049.17860000	24.91700000	0.00000000
236	3051.16300000	51.09960000	0.00000000
237	3052.10380000	48.42600000	0.00000000
238	3058.80710000	45.75690000	0.00000000
239	3065.39670000	30.58970000	0.00000000
240	3066.60420000	36.51110000	0.00000000
241	3072.00850000	19.78590000	0.00000000
242	3079.59700000	163.48180000	0.00000000
243	3080.86140000	18.55580000	0.00000000
244	3087.59640000	15.04620000	0.00000000
245	3096.75310000	78.20830000	0.00000000
246	3098.66410000	12.00080000	0.00000000
247	3101.36170000	18.63770000	0.00000000
248	3102.94140000	17.79230000	0.00000000
249	3103.30300000	25.60370000	0.00000000
250	3103.61760000	14.29120000	0.00000000
251	3104.95330000	59.28470000	0.00000000
252	3106.88720000	9.29590000	0.00000000
253	3109.91400000	111.97060000	0.00000000
254	3111.66470000	51.72570000	0.00000000
255	3113.63770000	18.93330000	0.00000000
256	3115.63210000	17.24840000	0.00000000
257	3116.27650000	75.75910000	0.00000000
258	3118.95890000	84.94600000	0.00000000
259	3123.98430000	29.25950000	0.00000000
260	3136.18130000	23.72660000	0.00000000
261	3139.69660000	10.69210000	0.00000000
262	3143.09100000	31.84150000	0.00000000
263	3151.40820000	13.04920000	0.00000000
264	3162.45940000	10.85870000	0.00000000
265	3162.98490000	3.71920000	0.00000000
266	3169.97180000	6.66090000	0.00000000
267	3179.43560000	38.45950000	0.00000000
268	3180.09970000	30.15050000	0.00000000
269	3186.33150000	40.71030000	0.00000000
270	3192.85450000	18.06810000	0.00000000
271	3200.87310000	18.57060000	0.00000000
272	3208.83760000	6.86800000	0.00000000
273	3211.28540000	25.31140000	0.00000000

Lithiated Dihydroquinoline with Three THF Molecules Above C2 Carbon of Dihydroquinoline

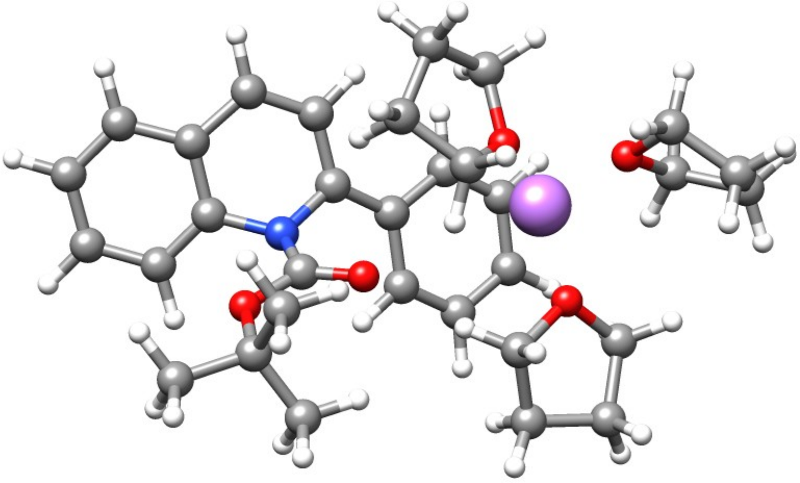

Route : # opt freq b3lyp/6-311g(d,p) scrf=(solvent=thf)
geom=connectivity empiricaldispersion=gd3bj
int=ultrafine
pop=(regular,mk)

SMILES :
CC(C)(C)OC(=O)N2c1ccccc1C=CC2([Li])c3ccccc3.C1CCOC1.C1CCOC1.
C1CCOC1

Formula : $C_{32}H_{44}LiNO_5$

Charge : 0

Multiplicity : 1

Dipole : 17.7760 Debye

Energy : -1684.99681103 a.u.

Gibbs Energy : -1684.366906 a.u.

Number of imaginary frequencies : 0

Cartesian Coordinates (XYZ format)

83

C	6.30007400	1.78684700	0.96736600
C	5.98501600	0.57760700	1.59799100
C	5.31373500	2.53432200	0.33857400
C	3.97264300	2.09312700	0.30039600
C	3.69459800	0.82805900	0.88372600
C	4.67154900	0.10827800	1.55564200
C	2.87105900	2.88401000	-0.16916800
C	1.59583500	2.54455200	0.26072600
C	1.31199200	1.31631000	0.86501000
N	2.36075200	0.32508400	0.69185600

Li	-2.47310000	0.37819800	-0.11454600
C	0.15183600	0.98225300	1.62718200
H	3.06442600	3.84962300	-0.62096800
H	0.81024800	3.29019900	0.19294600
H	7.32175000	2.15209200	0.98128200
H	5.56224100	3.48829900	-0.11627100
H	6.75369200	0.00027100	2.09797000
H	4.41020300	-0.84415400	1.99991900
C	2.11367700	-0.74498400	-0.12935500
O	3.22965300	-1.46335600	-0.37112000
O	1.00213300	-1.03873800	-0.54790100
C	3.19280700	-2.67151300	-1.20340100
C	4.65557400	-3.11042400	-1.20491700
H	5.28607600	-2.32498200	-1.62569500
H	4.77508400	-4.01621200	-1.80321500
H	4.99245500	-3.31549600	-0.18679100
C	2.73473900	-2.32658300	-2.62111300
H	3.34140700	-1.51060100	-3.02080300
H	1.68915300	-2.02672700	-2.63264300
H	2.86317300	-3.19872200	-3.26720300
C	2.31204000	-3.73736300	-0.55007900
H	2.62900000	-3.90347900	0.48239900
H	2.41492600	-4.67901000	-1.09525200
H	1.26618200	-3.43819200	-0.55472000
C	-0.94549500	1.88161900	1.77915000
C	0.00719900	-0.27910900	2.27272900
C	-1.12392400	-0.60200300	3.00512200
C	-2.19286100	0.29262800	3.13145600
C	-2.07986700	1.53915400	2.50487200
H	0.81169100	-0.99768100	2.19671000
H	-0.89024600	2.87348200	1.34859400
H	-2.88058300	2.26554700	2.59671400
H	-1.17788300	-1.57269600	3.48758100
H	-3.07144500	0.03740400	3.71120100
C	-6.48234000	0.68143000	0.23767200
C	-6.09263600	0.44263800	-1.23882100
C	-5.21115400	1.28804000	0.87303300
H	-6.77958700	-0.24394300	0.73154500
H	-7.31574600	1.38089000	0.31484500
C	-4.88638100	1.35569300	-1.42241800
H	-6.90144000	0.67587000	-1.93195400
H	-5.79395200	-0.59532400	-1.39935500
O	-4.19977900	1.28152300	-0.15778100
H	-5.18948700	2.39295300	-1.60813100
H	-4.18289800	1.04076800	-2.19046900
H	-4.81994600	0.71507100	1.71131600
H	-5.37671500	2.31928600	1.19774500
O	-2.83519400	-1.50476900	0.00122700
C	-1.82165600	-2.51501800	-0.26025400
C	-3.87372900	-2.05311800	0.85297600
C	-2.02898000	-3.57034200	0.81589700
H	-0.84991900	-2.02874200	-0.22492300
H	-1.99586300	-2.92602800	-1.26083800
C	-3.55137200	-3.53914000	1.00610600
H	-1.51994600	-3.27125000	1.73455500
H	-1.65483400	-4.54915700	0.51316500
H	-4.84044700	-1.86951600	0.38275600
H	-3.83061800	-1.52935000	1.81036800

H	-3.87366400	-3.92848800	1.97254000
H	-4.04229400	-4.12066500	0.22132900
C	-0.86264000	0.28751700	-2.67848800
C	0.32493900	1.19419300	-2.95496500
O	-1.67721600	1.03203900	-1.73992200
H	-0.58406300	-0.64746800	-2.20125200
H	-1.45239700	0.09638500	-3.58270300
C	-0.32282000	2.58617200	-2.95353800
H	0.82041500	0.95648500	-3.89755500
H	1.04556100	1.10858900	-2.14148200
C	-1.43745700	2.45506400	-1.90850400
H	-1.14320200	2.85034600	-0.93668600
H	-2.36953600	2.93235200	-2.21884400
H	-0.74410400	2.81517900	-3.93551300
H	0.38438800	3.37350300	-2.69121700

Frequencies

Mode	IR frequency	IR intensity	Raman intensity
1	19.57600000	0.73980000	0.00000000
2	21.98940000	0.77480000	0.00000000
3	23.10150000	0.72340000	0.00000000
4	31.75570000	0.05110000	0.00000000
5	38.89100000	3.52200000	0.00000000
6	42.27820000	0.71450000	0.00000000
7	47.34550000	0.50110000	0.00000000
8	47.85290000	0.49000000	0.00000000
9	52.66230000	2.14670000	0.00000000
10	58.24620000	0.74310000	0.00000000
11	61.20860000	0.48970000	0.00000000
12	64.01670000	0.12280000	0.00000000
13	72.88670000	0.16830000	0.00000000
14	74.08270000	1.75560000	0.00000000
15	77.97440000	1.58830000	0.00000000
16	84.91330000	1.00890000	0.00000000
17	91.21660000	2.60290000	0.00000000
18	93.16960000	2.21060000	0.00000000
19	96.16370000	0.83050000	0.00000000
20	99.90780000	3.86550000	0.00000000
21	107.89570000	1.98140000	0.00000000
22	114.50980000	1.01710000	0.00000000
23	118.36790000	6.67430000	0.00000000
24	127.64230000	0.73900000	0.00000000
25	133.68450000	0.46850000	0.00000000
26	149.02110000	6.14880000	0.00000000
27	156.49430000	7.51010000	0.00000000
28	159.63490000	5.04940000	0.00000000

29	163.05280000	7.65020000	0.00000000
30	172.95510000	11.34250000	0.00000000
31	184.28740000	3.56900000	0.00000000
32	205.62560000	1.52570000	0.00000000
33	213.72450000	0.42200000	0.00000000
34	238.18930000	0.02440000	0.00000000
35	248.68070000	14.17270000	0.00000000
36	267.19460000	0.87020000	0.00000000
37	269.93940000	0.15360000	0.00000000
38	274.36840000	3.10810000	0.00000000
39	278.30610000	5.57510000	0.00000000
40	294.08680000	5.93020000	0.00000000
41	315.00260000	7.96490000	0.00000000
42	329.62280000	14.09500000	0.00000000
43	345.84310000	7.54300000	0.00000000
44	351.22290000	2.14460000	0.00000000
45	392.06410000	63.88790000	0.00000000
46	395.33760000	137.69820000	0.00000000
47	429.23970000	2.84110000	0.00000000
48	433.00330000	10.93580000	0.00000000
49	444.81720000	18.75320000	0.00000000
50	455.59990000	112.72510000	0.00000000
51	458.24150000	5.99090000	0.00000000
52	471.04890000	30.24690000	0.00000000
53	476.40130000	9.19530000	0.00000000
54	500.66740000	71.66350000	0.00000000
55	506.99690000	16.72250000	0.00000000
56	517.31090000	37.82090000	0.00000000
57	551.43770000	53.34440000	0.00000000
58	559.58900000	15.42320000	0.00000000
59	583.20240000	2.71590000	0.00000000
60	589.11330000	6.25540000	0.00000000
61	595.49100000	49.47030000	0.00000000
62	605.20130000	1.76820000	0.00000000
63	612.16780000	7.70440000	0.00000000
64	637.41060000	0.71230000	0.00000000
65	683.32690000	15.64950000	0.00000000
66	692.19590000	55.63520000	0.00000000
67	694.66720000	6.01310000	0.00000000
68	699.03010000	21.94910000	0.00000000
69	703.19480000	33.30200000	0.00000000
70	708.20550000	57.14400000	0.00000000
71	716.07000000	22.82190000	0.00000000

72	741.97940000	24.06830000	0.00000000
73	746.68980000	186.78070000	0.00000000
74	753.25850000	26.57610000	0.00000000
75	769.28530000	4.09260000	0.00000000
76	778.38620000	25.62030000	0.00000000
77	802.35950000	2.26470000	0.00000000
78	802.71330000	2.84580000	0.00000000
79	828.30740000	4.07740000	0.00000000
80	836.05450000	11.18930000	0.00000000
81	851.56930000	17.92850000	0.00000000
82	852.90490000	18.43530000	0.00000000
83	857.75490000	16.14660000	0.00000000
84	858.65960000	12.05580000	0.00000000
85	874.97900000	24.71000000	0.00000000
86	875.98730000	23.41830000	0.00000000
87	879.06390000	29.47030000	0.00000000
88	888.59000000	28.02180000	0.00000000
89	891.30880000	96.48270000	0.00000000
90	895.10910000	7.11020000	0.00000000
91	910.96460000	7.40360000	0.00000000
92	911.36380000	81.72690000	0.00000000
93	918.67140000	146.68250000	0.00000000
94	921.74940000	15.16650000	0.00000000
95	922.91670000	31.32300000	0.00000000
96	925.89380000	56.76940000	0.00000000
97	926.67210000	3.35230000	0.00000000
98	928.99490000	6.12450000	0.00000000
99	931.11510000	14.28440000	0.00000000
100	932.36270000	18.81740000	0.00000000
101	933.12780000	46.09100000	0.00000000
102	940.77430000	4.37220000	0.00000000
103	961.01400000	2.39000000	0.00000000
104	968.33710000	0.20480000	0.00000000
105	970.10290000	3.30640000	0.00000000
106	973.84160000	2.73880000	0.00000000
107	974.32110000	1.20300000	0.00000000
108	979.31020000	6.08980000	0.00000000
109	983.91360000	5.08990000	0.00000000
110	997.56290000	196.70410000	0.00000000
111	1025.28310000	72.43720000	0.00000000
112	1043.29610000	37.20910000	0.00000000
113	1046.11400000	24.14070000	0.00000000
114	1047.19830000	10.81450000	0.00000000

115	1048.84980000	42.00170000	0.00000000
116	1049.13100000	2.43870000	0.00000000
117	1049.77720000	59.59860000	0.00000000
118	1050.77900000	68.60920000	0.00000000
119	1054.19700000	128.38900000	0.00000000
120	1060.31850000	10.45030000	0.00000000
121	1060.94870000	106.43660000	0.00000000
122	1089.56480000	70.36350000	0.00000000
123	1100.87580000	38.81810000	0.00000000
124	1129.47200000	171.98410000	0.00000000
125	1141.37500000	65.66100000	0.00000000
126	1153.35100000	0.74420000	0.00000000
127	1164.99530000	1.30900000	0.00000000
128	1168.24450000	51.81980000	0.00000000
129	1170.46090000	8.30840000	0.00000000
130	1170.55960000	3.18240000	0.00000000
131	1189.22240000	13.34550000	0.00000000
132	1191.65090000	31.00390000	0.00000000
133	1193.77210000	446.08290000	0.00000000
134	1202.41140000	207.85440000	0.00000000
135	1207.99000000	11.69480000	0.00000000
136	1210.85670000	8.81320000	0.00000000
137	1217.48870000	5.27120000	0.00000000
138	1236.95540000	7.30110000	0.00000000
139	1240.49070000	4.03230000	0.00000000
140	1248.84790000	5.60090000	0.00000000
141	1254.74100000	273.37390000	0.00000000
142	1259.75710000	1.37490000	0.00000000
143	1265.67710000	9.46620000	0.00000000
144	1266.10430000	21.03560000	0.00000000
145	1274.58270000	187.50900000	0.00000000
146	1275.99030000	34.89410000	0.00000000
147	1276.94060000	52.33410000	0.00000000
148	1283.40710000	6.02850000	0.00000000
149	1288.54850000	4.48770000	0.00000000
150	1296.07150000	558.22450000	0.00000000
151	1314.67080000	8.43810000	0.00000000
152	1326.14950000	3.71610000	0.00000000
153	1327.59100000	194.10910000	0.00000000
154	1328.53490000	40.63180000	0.00000000
155	1329.99610000	4.54460000	0.00000000
156	1340.04560000	66.73520000	0.00000000
157	1341.55800000	21.10230000	0.00000000

158	1347.57860000	9.34600000	0.00000000
159	1350.79980000	647.93420000	0.00000000
160	1358.64810000	27.82930000	0.00000000
161	1366.41950000	1.90180000	0.00000000
162	1370.66830000	182.31830000	0.00000000
163	1372.55310000	76.38960000	0.00000000
164	1380.92140000	0.92160000	0.00000000
165	1390.98110000	27.31080000	0.00000000
166	1396.36640000	41.51840000	0.00000000
167	1396.74170000	0.45010000	0.00000000
168	1402.89700000	4.35360000	0.00000000
169	1417.64980000	0.99270000	0.00000000
170	1419.56760000	25.13130000	0.00000000
171	1445.75280000	263.21620000	0.00000000
172	1466.20340000	1.44440000	0.00000000
173	1471.02890000	1035.47220000	0.00000000
174	1477.97200000	832.21310000	0.00000000
175	1484.37180000	3.90930000	0.00000000
176	1485.59300000	5.25320000	0.00000000
177	1486.19830000	19.63550000	0.00000000
178	1486.30720000	8.36630000	0.00000000
179	1487.24050000	16.42340000	0.00000000
180	1489.40850000	5.11440000	0.00000000
181	1494.65200000	18.15360000	0.00000000
182	1495.74650000	10.58030000	0.00000000
183	1498.64680000	5.46750000	0.00000000
184	1500.27770000	21.93090000	0.00000000
185	1503.86410000	10.41590000	0.00000000
186	1506.70750000	12.31690000	0.00000000
187	1516.34920000	4.96900000	0.00000000
188	1518.58230000	24.85010000	0.00000000
189	1522.22400000	3.32770000	0.00000000
190	1525.13520000	7.17420000	0.00000000
191	1527.65190000	1.15990000	0.00000000
192	1531.49910000	0.81070000	0.00000000
193	1536.30800000	10.76050000	0.00000000
194	1546.51790000	851.76720000	0.00000000
195	1570.14920000	47.27800000	0.00000000
196	1588.67750000	73.66870000	0.00000000
197	1629.36830000	220.99040000	0.00000000
198	1635.44100000	77.80750000	0.00000000
199	1706.01120000	731.12890000	0.00000000
200	3008.69950000	98.43520000	0.00000000

201	3012.26590000	66.30440000	0.00000000
202	3014.53990000	88.51130000	0.00000000
203	3035.84100000	18.44200000	0.00000000
204	3036.78770000	29.68950000	0.00000000
205	3036.95600000	65.50420000	0.00000000
206	3044.04100000	32.99890000	0.00000000
207	3046.09630000	27.82940000	0.00000000
208	3047.54840000	36.46990000	0.00000000
209	3052.83030000	66.89210000	0.00000000
210	3057.61080000	107.34440000	0.00000000
211	3058.86740000	32.84690000	0.00000000
212	3062.03950000	25.84030000	0.00000000
213	3068.59510000	45.36240000	0.00000000
214	3070.67340000	24.32110000	0.00000000
215	3097.09920000	15.06200000	0.00000000
216	3097.54250000	29.92880000	0.00000000
217	3098.81840000	7.35950000	0.00000000
218	3098.94400000	16.28370000	0.00000000
219	3099.55340000	28.32220000	0.00000000
220	3106.31650000	28.56520000	0.00000000
221	3110.99050000	46.80720000	0.00000000
222	3113.43620000	62.53010000	0.00000000
223	3113.60140000	36.82060000	0.00000000
224	3114.87920000	41.44180000	0.00000000
225	3114.96250000	70.90530000	0.00000000
226	3124.98350000	44.10730000	0.00000000
227	3127.78020000	51.17760000	0.00000000
228	3133.99490000	25.37720000	0.00000000
229	3143.62160000	31.81200000	0.00000000
230	3144.19260000	0.44160000	0.00000000
231	3144.28490000	5.22840000	0.00000000
232	3146.92960000	32.41420000	0.00000000
233	3148.73650000	31.13150000	0.00000000
234	3151.78340000	29.55290000	0.00000000
235	3154.37860000	21.03690000	0.00000000
236	3158.88920000	7.15540000	0.00000000
237	3160.17470000	52.56550000	0.00000000
238	3170.46410000	32.96750000	0.00000000
239	3173.89910000	51.47200000	0.00000000
240	3178.03680000	68.67360000	0.00000000
241	3184.81510000	48.19310000	0.00000000
242	3190.11660000	31.93590000	0.00000000
243	3199.40590000	12.80030000	0.00000000

Lithiated Dihydroquinoline with Two THF & One Acetone Molecules Above C2 Carbon of Dihydroquinoline

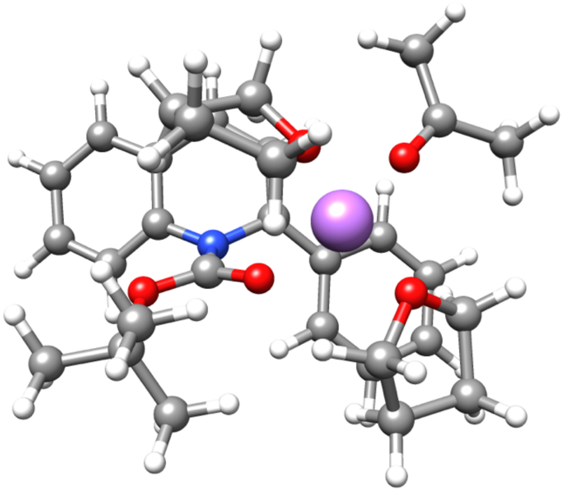

Route : # opt freq b3lyp/6-311g(d,p) scrf=(solvent=thf)
geom=connectivity empiricaldispersion=gd3bj
int=ultrafine
pop=(regular,mk)

SMILES :
CC(C)(C)OC(=O)N2c1ccccc1C=CC2([Li])c3ccccc3.C1CCOC1.C1CCOC1.
CC(C)=O

Formula : $C_{31}H_{42}LiNO_5$

Charge : 0

Multiplicity : 1

Dipole : 11.0852 Debye

Energy : -1645.70220632 a.u.

Gibbs Energy : -1645.103349 a.u.

Number of imaginary frequencies : 0

Cartesian Coordinates (XYZ format)

80

C	5.08073700	2.66099400	0.67214400
C	4.84413300	1.71012600	1.67247600
C	4.07112000	3.03294600	-0.20385600
C	2.78436400	2.45512700	-0.12743600
C	2.59721500	1.43652500	0.84530800
C	3.59093300	1.10370100	1.75470100
C	1.64320400	2.91653100	-0.86524400
C	0.38278000	2.60756200	-0.37157800
C	0.18202800	1.62284300	0.59958200

N	1.33174100	0.75314200	0.78694900
Li	-1.31934000	-0.53791100	-0.99142600
C	-0.94086800	1.49596700	1.48821000
H	1.77121900	3.68645000	-1.61653500
H	-0.46580600	3.19797200	-0.69796900
H	6.05815500	3.12484500	0.59036000
H	4.25419500	3.80027600	-0.94955400
H	5.62988700	1.43097900	2.36425400
H	3.39542700	0.33730500	2.49376600
C	1.23511000	-0.55828500	0.44619400
O	2.39877700	-1.21710100	0.55982900
O	0.17680500	-1.09511500	0.10018200
C	2.45323600	-2.67507400	0.77318300
C	3.92548500	-2.90230300	1.10425000
H	4.55584200	-2.56373800	0.27976500
H	4.10961800	-3.96548700	1.27255900
H	4.20487800	-2.35003400	2.00317500
C	2.06582500	-3.41572800	-0.50333400
H	2.70228900	-3.09864800	-1.33138100
H	1.02764900	-3.22359300	-0.76101900
H	2.20326500	-4.48988900	-0.35763000
C	1.56432100	-3.05490400	1.95645300
H	1.83857300	-2.47039700	2.83781500
H	1.70395500	-4.11268800	2.19012200
H	0.51339400	-2.88322500	1.73132400
C	-2.06505300	2.36176700	1.40757800
C	-0.99807200	0.49977900	2.49786100
C	-2.09472200	0.37366700	3.33996100
C	-3.20101700	1.21612600	3.21846400
C	-3.16399400	2.21383300	2.23876700
H	-0.16163600	-0.17408700	2.62133200
H	-2.06294500	3.17183800	0.68939100
H	-4.00093400	2.89660800	2.13166900
H	-2.08806300	-0.40232600	4.09884800
H	-4.05847100	1.10858100	3.87179200
C	1.20341400	-2.04475400	-3.87415600
C	1.71885800	-0.74093000	-3.24514200
C	-0.22096600	-2.17364700	-3.30901900
H	1.81993300	-2.90998600	-3.62921900
H	1.17324300	-1.94985000	-4.96208600
C	0.44421000	0.08443600	-3.15011900
H	2.48746600	-0.25313600	-3.84586800
H	2.12325500	-0.91854600	-2.24702500
O	-0.58134900	-0.87078400	-2.79563300
H	0.19179000	0.54333900	-4.11372200
H	0.47041100	0.85680700	-2.38264100
H	-0.27755600	-2.88169800	-2.48054400
H	-0.94737500	-2.46877700	-4.07010400
O	-2.69401900	-1.80328600	-0.39890900
C	-2.26910700	-3.06682400	0.15759200
C	-3.71873700	-1.21715900	0.45634900
C	-2.60054600	-2.97332900	1.63972000
H	-1.20597000	-3.17526200	-0.04853900
H	-2.82241200	-3.87886000	-0.32800400
C	-3.92561700	-2.20007900	1.61107000
H	-1.83427200	-2.38864500	2.15260300
H	-2.68081600	-3.95191200	2.11542700
H	-4.61888700	-1.06294400	-0.14168900

H	-3.34999800	-0.25404700	0.80782600
H	-4.13399600	-1.67799000	2.54460500
H	-4.75509600	-2.88009600	1.39940900
O	-2.39513200	0.98904500	-1.51697900
C	-3.03222800	1.98091500	-1.82033800
C	-2.47964500	3.00209000	-2.77999300
C	-4.41272900	2.22695700	-1.27097800
H	-2.39967400	3.97208600	-2.28036200
H	-3.17183100	3.13588700	-3.61663800
H	-1.50265200	2.69326900	-3.14581000
H	-4.52662900	3.26739700	-0.95913300
H	-4.61406700	1.56018000	-0.43647300
H	-5.14221600	2.04846000	-2.06840700

Frequencies

Mode	IR frequency	IR intensity	Raman intensity
1	24.54490000	0.31300000	0.00000000
2	30.26800000	0.29870000	0.00000000
3	34.33600000	0.29540000	0.00000000
4	37.36490000	4.89020000	0.00000000
5	45.23480000	1.55790000	0.00000000
6	45.52480000	0.84150000	0.00000000
7	48.03680000	0.88770000	0.00000000
8	51.35080000	1.06800000	0.00000000
9	55.27910000	1.04590000	0.00000000
10	56.40780000	9.31680000	0.00000000
11	61.74370000	1.18090000	0.00000000
12	69.61780000	1.77290000	0.00000000
13	73.50030000	0.05800000	0.00000000
14	76.98440000	0.55880000	0.00000000
15	82.20920000	0.61270000	0.00000000
16	85.38620000	1.07320000	0.00000000
17	85.92360000	0.03360000	0.00000000
18	95.78730000	3.06870000	0.00000000
19	97.10810000	2.52000000	0.00000000
20	103.36290000	2.47720000	0.00000000
21	106.10970000	0.41450000	0.00000000
22	112.22590000	3.30690000	0.00000000
23	116.55260000	2.50840000	0.00000000
24	121.41050000	0.67520000	0.00000000
25	131.57680000	1.02630000	0.00000000
26	136.86950000	5.03700000	0.00000000
27	147.35680000	5.29820000	0.00000000
28	153.42980000	3.68860000	0.00000000
29	156.88910000	4.72950000	0.00000000

30	178.72700000	1.44390000	0.00000000
31	192.96830000	1.39350000	0.00000000
32	197.31990000	1.32430000	0.00000000
33	209.71680000	0.55790000	0.00000000
34	222.38820000	3.98060000	0.00000000
35	248.12160000	9.37060000	0.00000000
36	258.57260000	0.87900000	0.00000000
37	267.93480000	3.60170000	0.00000000
38	271.62180000	0.72860000	0.00000000
39	282.59700000	3.39780000	0.00000000
40	284.76980000	2.15030000	0.00000000
41	316.98830000	6.82010000	0.00000000
42	327.32270000	23.09980000	0.00000000
43	344.78650000	1.12090000	0.00000000
44	354.21320000	8.56690000	0.00000000
45	366.52730000	93.15370000	0.00000000
46	393.69130000	21.76100000	0.00000000
47	395.63280000	22.10930000	0.00000000
48	423.97150000	0.35700000	0.00000000
49	427.61490000	19.19480000	0.00000000
50	444.37390000	0.54050000	0.00000000
51	455.94650000	49.34130000	0.00000000
52	458.81820000	54.51070000	0.00000000
53	466.47690000	78.19060000	0.00000000
54	475.41870000	47.08610000	0.00000000
55	493.42530000	49.32990000	0.00000000
56	500.35280000	119.39040000	0.00000000
57	506.01170000	11.88090000	0.00000000
58	517.24750000	17.51810000	0.00000000
59	530.99140000	13.13410000	0.00000000
60	549.11200000	37.12050000	0.00000000
61	560.73000000	9.88600000	0.00000000
62	581.91020000	6.32850000	0.00000000
63	590.17760000	2.82360000	0.00000000
64	597.86840000	30.44240000	0.00000000
65	604.47740000	1.83250000	0.00000000
66	635.89000000	0.56800000	0.00000000
67	680.92140000	13.40730000	0.00000000
68	682.46830000	10.70040000	0.00000000
69	687.95690000	69.53340000	0.00000000
70	692.33270000	25.48810000	0.00000000
71	701.46250000	62.26720000	0.00000000
72	715.01720000	11.97940000	0.00000000

73	741.79240000	11.73860000	0.00000000
74	747.09150000	183.01980000	0.00000000
75	753.55620000	24.68670000	0.00000000
76	766.45320000	2.65350000	0.00000000
77	779.57940000	29.05400000	0.00000000
78	800.06820000	8.57900000	0.00000000
79	808.29570000	0.73140000	0.00000000
80	825.55510000	0.50850000	0.00000000
81	831.44080000	6.14340000	0.00000000
82	842.37920000	20.85900000	0.00000000
83	849.08680000	19.59550000	0.00000000
84	853.67790000	9.44960000	0.00000000
85	865.32270000	7.70120000	0.00000000
86	874.58470000	24.31270000	0.00000000
87	880.40520000	13.45340000	0.00000000
88	882.51900000	3.61330000	0.00000000
89	885.82360000	33.90540000	0.00000000
90	887.60830000	50.01540000	0.00000000
91	900.98850000	27.19350000	0.00000000
92	907.51830000	87.77290000	0.00000000
93	911.63590000	23.31850000	0.00000000
94	918.25730000	12.48360000	0.00000000
95	923.24920000	15.88640000	0.00000000
96	926.62240000	51.82830000	0.00000000
97	927.68630000	5.09060000	0.00000000
98	929.85730000	16.33970000	0.00000000
99	930.87180000	4.79630000	0.00000000
100	932.60340000	29.96300000	0.00000000
101	936.68070000	11.96040000	0.00000000
102	963.70380000	2.11950000	0.00000000
103	967.68300000	0.16150000	0.00000000
104	971.53340000	0.58470000	0.00000000
105	974.98180000	0.21390000	0.00000000
106	976.31030000	2.00530000	0.00000000
107	977.31350000	0.87640000	0.00000000
108	998.82090000	145.77970000	0.00000000
109	1026.34600000	66.47540000	0.00000000
110	1043.38830000	8.84400000	0.00000000
111	1044.65900000	5.55450000	0.00000000
112	1047.17020000	40.05200000	0.00000000
113	1050.15390000	28.91530000	0.00000000
114	1052.02320000	2.90160000	0.00000000
115	1054.05420000	113.56290000	0.00000000

116	1060.62140000	103.68810000	0.00000000
117	1061.62270000	44.23840000	0.00000000
118	1093.13490000	1.95830000	0.00000000
119	1093.71460000	75.49130000	0.00000000
120	1105.69690000	55.45350000	0.00000000
121	1117.95390000	32.72050000	0.00000000
122	1133.35230000	175.52790000	0.00000000
123	1141.03820000	47.32220000	0.00000000
124	1162.58810000	1.49510000	0.00000000
125	1166.77140000	0.72880000	0.00000000
126	1169.47490000	46.89260000	0.00000000
127	1171.73150000	3.55630000	0.00000000
128	1189.43260000	29.23110000	0.00000000
129	1190.92560000	232.64870000	0.00000000
130	1191.21450000	113.88810000	0.00000000
131	1204.60520000	160.43430000	0.00000000
132	1212.19250000	6.46200000	0.00000000
133	1219.89810000	11.58220000	0.00000000
134	1238.78950000	7.87330000	0.00000000
135	1250.63260000	56.19020000	0.00000000
136	1252.83750000	1.03630000	0.00000000
137	1255.29540000	276.75780000	0.00000000
138	1263.97530000	2.44660000	0.00000000
139	1269.09700000	27.82990000	0.00000000
140	1274.63270000	20.53860000	0.00000000
141	1275.47310000	15.77850000	0.00000000
142	1277.63890000	186.45130000	0.00000000
143	1279.73810000	30.66410000	0.00000000
144	1296.78240000	436.34590000	0.00000000
145	1325.46020000	3.67710000	0.00000000
146	1325.71590000	1.02840000	0.00000000
147	1329.96150000	151.50840000	0.00000000
148	1341.94000000	6.10010000	0.00000000
149	1344.84110000	4.57080000	0.00000000
150	1346.34710000	0.31430000	0.00000000
151	1351.82510000	202.05800000	0.00000000
152	1363.31160000	108.03660000	0.00000000
153	1369.07330000	0.96830000	0.00000000
154	1374.34400000	1.41500000	0.00000000
155	1384.33030000	420.38830000	0.00000000
156	1385.18980000	242.90050000	0.00000000
157	1391.14780000	95.54410000	0.00000000
158	1394.69820000	37.89550000	0.00000000

159	1399.45870000	73.06990000	0.00000000
160	1400.66670000	11.62870000	0.00000000
161	1403.66860000	6.65510000	0.00000000
162	1421.04950000	25.79130000	0.00000000
163	1444.40900000	297.20680000	0.00000000
164	1450.53400000	12.52520000	0.00000000
165	1458.77640000	11.21130000	0.00000000
166	1462.08930000	31.51300000	0.00000000
167	1467.78410000	2.80710000	0.00000000
168	1472.33780000	781.12520000	0.00000000
169	1479.09420000	201.68950000	0.00000000
170	1479.97730000	521.45830000	0.00000000
171	1482.02990000	56.89660000	0.00000000
172	1484.96320000	3.08210000	0.00000000
173	1485.56780000	2.05070000	0.00000000
174	1488.85090000	6.43230000	0.00000000
175	1489.96980000	16.06980000	0.00000000
176	1492.79690000	16.92640000	0.00000000
177	1497.72270000	12.41630000	0.00000000
178	1501.82450000	32.24790000	0.00000000
179	1503.18310000	16.10630000	0.00000000
180	1509.63780000	40.74370000	0.00000000
181	1519.29010000	11.88610000	0.00000000
182	1525.55690000	10.02020000	0.00000000
183	1527.41800000	11.11910000	0.00000000
184	1533.19140000	5.92410000	0.00000000
185	1536.39450000	14.21220000	0.00000000
186	1547.57240000	737.34500000	0.00000000
187	1582.19430000	27.99190000	0.00000000
188	1588.16070000	57.80470000	0.00000000
189	1632.21260000	102.34500000	0.00000000
190	1634.64180000	147.03160000	0.00000000
191	1675.67890000	695.07570000	0.00000000
192	1776.60560000	421.05510000	0.00000000
193	3003.53210000	103.02780000	0.00000000
194	3012.22020000	97.48630000	0.00000000
195	3032.06930000	0.70590000	0.00000000
196	3038.19990000	5.31390000	0.00000000
197	3039.08340000	13.71330000	0.00000000
198	3042.20780000	30.52940000	0.00000000
199	3042.60650000	62.90770000	0.00000000
200	3044.54360000	37.87130000	0.00000000
201	3049.56050000	25.53140000	0.00000000

202	3055.15220000	66.17730000	0.00000000
203	3058.71900000	115.23430000	0.00000000
204	3061.33160000	6.60140000	0.00000000
205	3068.70510000	16.81720000	0.00000000
206	3083.35340000	39.29270000	0.00000000
207	3088.18170000	6.75850000	0.00000000
208	3097.82020000	15.19060000	0.00000000
209	3103.74920000	14.42060000	0.00000000
210	3106.02720000	36.65240000	0.00000000
211	3106.86000000	9.71990000	0.00000000
212	3107.30480000	21.78250000	0.00000000
213	3112.38620000	30.75660000	0.00000000
214	3114.17600000	85.21680000	0.00000000
215	3114.96740000	21.81960000	0.00000000
216	3115.64350000	58.00970000	0.00000000
217	3122.46950000	19.53890000	0.00000000
218	3124.18370000	26.20260000	0.00000000
219	3128.07280000	47.19760000	0.00000000
220	3142.83820000	10.90690000	0.00000000
221	3146.11350000	13.44430000	0.00000000
222	3146.96990000	35.72930000	0.00000000
223	3151.61160000	30.58140000	0.00000000
224	3152.03190000	15.31260000	0.00000000
225	3158.60600000	2.83980000	0.00000000
226	3159.26130000	17.10600000	0.00000000
227	3162.28020000	58.22850000	0.00000000
228	3164.09270000	7.30990000	0.00000000
229	3174.22930000	13.76260000	0.00000000
230	3176.62270000	44.08970000	0.00000000
231	3180.21830000	75.92730000	0.00000000
232	3186.73730000	78.87510000	0.00000000
233	3194.40780000	25.20390000	0.00000000
234	3201.66760000	13.12880000	0.00000000

Acetone Quenched Dihydroquinoline Above C-2 Position

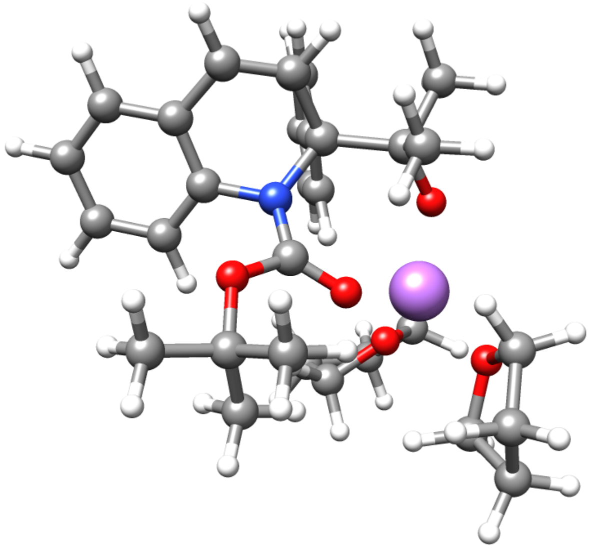

Route : # opt freq b3lyp/6-311g(d,p) scrf=(solvent=thf)
geom=connectivity empiricaldispersion=gd3bj
int=ultrafine
pop=(regular,mk)

SMILES :
CC(C)(C)OC3=O[Li]OC(C)(C)C4(c1ccccc1)C=Cc2ccccc2N34.C1CCOC1.
C1CCOC1.

Formula : $C_{31}H_{42}LiNO_5$

Charge : 0

Multiplicity : 1

Dipole : 9.7443 Debye

Energy : -1645.68981787 a.u.

Gibbs Energy : -1645.083789 a.u.

Number of imaginary frequencies : 0

Cartesian Coordinates (XYZ format)

80

C	-4.19430900	-2.52012200	-1.80415700
C	-3.07830500	-2.02623800	-2.48356100
C	-4.26481400	-2.40871900	-0.42097200
C	-3.22416000	-1.81177300	0.30121400
C	-2.09616900	-1.34480000	-0.38926900
C	-2.03543500	-1.43644900	-1.77751600
C	-3.25325500	-1.61288700	1.74156900
C	-2.46486700	-0.68628400	2.29544600
C	-1.52725100	0.17643900	1.45649000

N	-1.02734800	-0.75264000	0.36048600
Li	1.71402700	1.16246200	0.54807600
C	-2.28131300	1.29758400	0.73756600
H	-3.94098300	-2.20003600	2.34074600
H	-2.50774500	-0.50913900	3.35965100
H	-5.00718100	-2.97865900	-2.35453100
H	-5.13321600	-2.77651800	0.11448400
H	-3.02504000	-2.09216800	-3.56380900
H	-1.17168300	-1.04630500	-2.30118000
C	0.23282300	-1.27695100	0.17302900
O	0.16091400	-2.54581000	-0.25888900
O	1.29873800	-0.70206300	0.34086000
C	1.35124700	-3.28112800	-0.73564700
C	0.74126200	-4.58971500	-1.22961700
H	0.21107700	-5.09193600	-0.41838000
H	1.52842200	-5.25153000	-1.59637800
H	0.03552100	-4.39955400	-2.04044500
C	2.30072300	-3.52686600	0.43506500
H	1.76940000	-4.01681500	1.25400000
H	2.72295300	-2.59189600	0.79663200
H	3.11303300	-4.18234400	0.11170100
C	2.02425100	-2.53247200	-1.88449100
H	1.29594400	-2.30100300	-2.66522100
H	2.79607500	-3.17038200	-2.32100800
H	2.48785200	-1.61041600	-1.54127700
C	-3.54704400	1.72330300	1.14021000
C	-1.66705300	1.95019700	-0.33516500
C	-2.30422700	3.00149900	-0.98636800
C	-3.56899000	3.42326100	-0.57546300
C	-4.18779400	2.77913600	0.49175800
H	-0.68585900	1.62658600	-0.64640700
H	-4.04306400	1.22633200	1.96388600
H	-5.17286700	3.09164000	0.81989200
H	-1.81621600	3.49631000	-1.81870900
H	-4.06613700	4.24062600	-1.08542700
C	5.79013700	0.25214000	0.22941300
C	5.24536800	-0.63465300	1.35720000
C	4.51152500	0.88316200	-0.30792900
H	6.33205400	-0.30647700	-0.53497200
H	6.45423200	1.02099600	0.63353400
C	4.12172200	0.22587300	1.93514400
H	5.99505500	-0.89166900	2.10636700
H	4.84208100	-1.56243000	0.94359400
O	3.66396600	1.06726100	0.84645400
H	4.47926300	0.87612400	2.73827400
H	3.27342400	-0.35503400	2.29809400
H	4.01118100	0.21580700	-1.01800600
H	4.65392300	1.85499000	-0.78066000
O	1.67080100	1.97311200	-1.25310400
C	1.56364200	1.32384400	-2.53101300
C	1.46720500	3.40016800	-1.40327100
C	0.67590900	2.24084300	-3.36209000
H	1.15001700	0.33099000	-2.36199700
H	2.56099100	1.22114800	-2.97574100
C	1.12498400	3.63035700	-2.88217800
H	-0.37483100	2.06436200	-3.12166100
H	0.81312300	2.09641300	-4.43439600
H	2.37750300	3.91858200	-1.09472400

H	0.65386300	3.68198600	-0.73288400
H	0.35739700	4.39427900	-3.00992000
H	2.01220800	3.94795200	-3.43491400
O	0.47642700	1.62740400	1.72259600
C	-0.39552700	0.85831100	2.41300900
C	0.31031300	-0.25344900	3.24147300
C	-1.15348300	1.76611600	3.42785100
H	-0.37558600	-0.83619800	3.86258900
H	1.02589600	0.24231600	3.90233800
H	0.86876300	-0.93845700	2.60706400
H	-1.90314700	1.26951300	4.05053700
H	-1.62721800	2.59446700	2.90141000
H	-0.39057100	2.18105500	4.09047400

Frequencies

Mode	IR frequency	IR intensity	Raman intensity
1	21.32280000	0.45680000	0.00000000
2	25.63410000	0.63820000	0.00000000
3	27.48770000	0.88950000	0.00000000
4	34.67050000	0.99060000	0.00000000
5	41.34340000	1.05600000	0.00000000
6	45.32070000	0.28340000	0.00000000
7	48.31380000	0.25080000	0.00000000
8	53.76150000	1.11930000	0.00000000
9	54.89220000	0.55920000	0.00000000
10	58.87970000	1.87480000	0.00000000
11	63.88190000	0.03490000	0.00000000
12	70.05560000	0.55890000	0.00000000
13	79.95380000	1.38840000	0.00000000
14	87.73370000	1.55060000	0.00000000
15	93.37770000	2.88680000	0.00000000
16	96.13030000	2.69750000	0.00000000
17	100.26450000	1.16710000	0.00000000
18	107.67610000	3.66150000	0.00000000
19	119.57070000	2.74470000	0.00000000
20	136.53930000	3.36800000	0.00000000
21	141.77020000	13.01000000	0.00000000
22	151.56710000	7.23840000	0.00000000
23	155.34190000	2.16070000	0.00000000
24	173.60610000	8.85970000	0.00000000
25	176.14740000	9.14330000	0.00000000
26	182.44440000	0.05680000	0.00000000
27	197.69650000	3.20740000	0.00000000
28	211.56800000	0.72360000	0.00000000
29	222.42000000	4.19430000	0.00000000

30	237.31170000	1.89550000	0.00000000
31	254.02700000	1.71410000	0.00000000
32	257.60800000	1.14080000	0.00000000
33	259.64020000	2.41150000	0.00000000
34	262.83770000	1.31230000	0.00000000
35	265.00640000	1.26450000	0.00000000
36	270.63140000	1.26450000	0.00000000
37	276.43260000	0.19430000	0.00000000
38	285.69900000	5.65970000	0.00000000
39	306.87440000	0.01850000	0.00000000
40	329.50730000	4.10840000	0.00000000
41	339.58710000	10.32110000	0.00000000
42	348.96680000	6.06460000	0.00000000
43	355.72150000	4.39440000	0.00000000
44	391.35900000	5.65830000	0.00000000
45	405.66110000	104.52820000	0.00000000
46	417.53870000	16.57700000	0.00000000
47	421.26620000	25.17340000	0.00000000
48	423.42260000	15.26160000	0.00000000
49	431.66330000	3.43790000	0.00000000
50	446.31230000	8.13070000	0.00000000
51	456.86170000	4.12310000	0.00000000
52	476.80140000	3.30320000	0.00000000
53	480.78950000	93.37160000	0.00000000
54	490.73990000	28.01630000	0.00000000
55	502.08410000	8.42410000	0.00000000
56	508.97620000	18.52770000	0.00000000
57	542.75560000	5.23290000	0.00000000
58	550.53410000	8.51020000	0.00000000
59	568.72210000	28.04880000	0.00000000
60	581.71070000	1.07340000	0.00000000
61	584.35440000	35.70580000	0.00000000
62	591.49150000	3.14390000	0.00000000
63	636.08030000	5.79560000	0.00000000
64	637.83580000	1.50210000	0.00000000
65	657.80140000	45.95070000	0.00000000
66	669.61210000	40.42310000	0.00000000
67	685.36710000	14.17430000	0.00000000
68	689.63720000	42.30390000	0.00000000
69	701.81530000	41.77670000	0.00000000
70	712.77030000	61.70830000	0.00000000
71	723.65630000	5.14930000	0.00000000
72	741.11210000	32.94620000	0.00000000

73	765.13910000	37.48120000	0.00000000
74	769.09780000	68.56260000	0.00000000
75	770.97290000	28.75370000	0.00000000
76	791.21490000	19.67560000	0.00000000
77	807.86340000	3.23640000	0.00000000
78	828.44820000	6.33660000	0.00000000
79	839.78880000	12.16830000	0.00000000
80	847.02560000	16.25640000	0.00000000
81	853.21340000	5.57590000	0.00000000
82	854.55950000	0.29290000	0.00000000
83	861.44250000	74.93200000	0.00000000
84	874.05160000	38.48910000	0.00000000
85	879.39290000	17.29240000	0.00000000
86	881.48310000	7.34870000	0.00000000
87	881.94650000	8.02700000	0.00000000
88	893.45640000	66.70390000	0.00000000
89	902.60690000	59.50360000	0.00000000
90	904.69120000	83.66000000	0.00000000
91	916.66610000	6.90170000	0.00000000
92	920.34440000	34.45660000	0.00000000
93	922.90710000	28.29600000	0.00000000
94	927.68990000	10.97910000	0.00000000
95	929.03330000	2.73000000	0.00000000
96	929.69040000	4.17740000	0.00000000
97	932.68920000	6.81140000	0.00000000
98	942.33490000	13.14990000	0.00000000
99	958.24430000	7.13050000	0.00000000
100	968.65680000	1.44120000	0.00000000
101	972.04570000	0.33840000	0.00000000
102	975.32150000	1.98820000	0.00000000
103	983.40430000	1.16730000	0.00000000
104	986.22420000	4.97800000	0.00000000
105	990.93300000	3.48030000	0.00000000
106	994.94610000	2.62010000	0.00000000
107	1004.23080000	8.73710000	0.00000000
108	1020.52340000	54.96170000	0.00000000
109	1023.64920000	12.31530000	0.00000000
110	1036.92380000	8.47990000	0.00000000
111	1039.06700000	8.97980000	0.00000000
112	1044.81230000	7.05460000	0.00000000
113	1047.61280000	61.56740000	0.00000000
114	1051.75870000	1.50780000	0.00000000
115	1057.78950000	3.88180000	0.00000000

116	1061.88830000	5.34990000	0.00000000
117	1063.21880000	111.94050000	0.00000000
118	1063.72890000	49.59520000	0.00000000
119	1069.38660000	122.82390000	0.00000000
120	1104.90730000	85.04420000	0.00000000
121	1118.48470000	67.10620000	0.00000000
122	1138.85620000	219.47610000	0.00000000
123	1152.15500000	69.30590000	0.00000000
124	1166.85520000	3.53550000	0.00000000
125	1167.52110000	7.11880000	0.00000000
126	1168.23320000	126.54410000	0.00000000
127	1177.05590000	0.72150000	0.00000000
128	1178.53240000	2.19280000	0.00000000
129	1186.33900000	380.40540000	0.00000000
130	1190.13510000	10.67360000	0.00000000
131	1190.85990000	14.45000000	0.00000000
132	1195.66590000	13.33820000	0.00000000
133	1197.96380000	21.00940000	0.00000000
134	1207.01320000	12.80670000	0.00000000
135	1212.63550000	6.93010000	0.00000000
136	1213.97650000	39.71380000	0.00000000
137	1241.67250000	45.96960000	0.00000000
138	1246.09400000	139.18030000	0.00000000
139	1260.39490000	0.06700000	0.00000000
140	1260.55950000	0.43100000	0.00000000
141	1264.48860000	7.58180000	0.00000000
142	1268.10450000	27.21550000	0.00000000
143	1268.88440000	20.88140000	0.00000000
144	1270.45960000	92.83180000	0.00000000
145	1278.79580000	74.81060000	0.00000000
146	1300.12450000	112.31010000	0.00000000
147	1322.06950000	2.16820000	0.00000000
148	1324.57480000	2.07510000	0.00000000
149	1329.67300000	208.67960000	0.00000000
150	1335.37180000	82.34970000	0.00000000
151	1343.75740000	1.41310000	0.00000000
152	1344.50080000	1.19870000	0.00000000
153	1350.04690000	246.92030000	0.00000000
154	1358.26380000	19.26810000	0.00000000
155	1369.40610000	0.20810000	0.00000000
156	1373.28810000	0.14450000	0.00000000
157	1378.12960000	14.59950000	0.00000000
158	1393.22010000	23.38660000	0.00000000

159	1394.28310000	32.34160000	0.00000000
160	1398.83720000	49.28870000	0.00000000
161	1399.98120000	1.56820000	0.00000000
162	1405.45470000	2.94180000	0.00000000
163	1417.99420000	7.35850000	0.00000000
164	1421.16010000	26.66020000	0.00000000
165	1467.55930000	0.38260000	0.00000000
166	1473.21970000	9.09590000	0.00000000
167	1477.29090000	12.16880000	0.00000000
168	1484.19870000	4.22380000	0.00000000
169	1485.15690000	15.65490000	0.00000000
170	1485.69720000	17.84260000	0.00000000
171	1486.70630000	0.66900000	0.00000000
172	1488.38920000	11.01430000	0.00000000
173	1489.69410000	3.52200000	0.00000000
174	1490.43440000	14.97070000	0.00000000
175	1490.76510000	15.37820000	0.00000000
176	1494.78180000	6.03140000	0.00000000
177	1499.72870000	14.54890000	0.00000000
178	1500.91100000	1.13580000	0.00000000
179	1509.72660000	1.81690000	0.00000000
180	1517.31610000	2.51000000	0.00000000
181	1518.82040000	84.96410000	0.00000000
182	1522.19900000	18.68310000	0.00000000
183	1525.51440000	5.85670000	0.00000000
184	1527.54540000	11.69760000	0.00000000
185	1527.97340000	17.71650000	0.00000000
186	1536.08970000	0.98570000	0.00000000
187	1606.87660000	38.10810000	0.00000000
188	1624.73680000	1.40360000	0.00000000
189	1642.66500000	6.85490000	0.00000000
190	1644.90400000	9.39550000	0.00000000
191	1680.95710000	0.68040000	0.00000000
192	1721.78110000	858.89560000	0.00000000
193	3005.37830000	90.25540000	0.00000000
194	3021.72070000	98.43430000	0.00000000
195	3025.28470000	32.42760000	0.00000000
196	3031.08230000	87.22290000	0.00000000
197	3038.09350000	44.94640000	0.00000000
198	3038.50920000	19.56420000	0.00000000
199	3041.03460000	37.29680000	0.00000000
200	3046.65900000	29.14580000	0.00000000
201	3047.38810000	22.11230000	0.00000000

202	3048.61410000	35.65630000	0.00000000
203	3053.36920000	32.43130000	0.00000000
204	3056.78420000	75.24710000	0.00000000
205	3060.01080000	18.83600000	0.00000000
206	3081.03770000	7.63460000	0.00000000
207	3088.33210000	151.57870000	0.00000000
208	3093.12640000	15.35580000	0.00000000
209	3097.46430000	49.70210000	0.00000000
210	3099.23170000	11.95280000	0.00000000
211	3102.17080000	9.88840000	0.00000000
212	3103.44950000	27.44710000	0.00000000
213	3104.27730000	28.60810000	0.00000000
214	3106.54040000	15.69150000	0.00000000
215	3112.83200000	94.62080000	0.00000000
216	3112.96460000	36.49250000	0.00000000
217	3114.05090000	73.32580000	0.00000000
218	3116.17820000	65.86950000	0.00000000
219	3121.78910000	51.10220000	0.00000000
220	3125.11100000	57.82020000	0.00000000
221	3140.19880000	31.85210000	0.00000000
222	3147.32740000	1.17980000	0.00000000
223	3150.02210000	26.44370000	0.00000000
224	3157.83240000	0.40890000	0.00000000
225	3161.85840000	9.81890000	0.00000000
226	3164.53290000	14.78780000	0.00000000
227	3167.35470000	35.59360000	0.00000000
228	3172.93800000	11.66350000	0.00000000
229	3181.07340000	51.41000000	0.00000000
230	3184.65390000	42.02040000	0.00000000
231	3192.25590000	16.69420000	0.00000000
232	3194.40070000	25.22460000	0.00000000
233	3217.12180000	18.32800000	0.00000000
234	3233.71830000	0.59770000	0.00000000

Thermochemistry Values

Tetrahydrofuran:

	Temperature	
	298 K	195 K
ε_0	–232.532329517 a.u.	–232.532329517 a.u.
ε_{ZPE}	0.116264 a.u.	0.116264 a.u.
E_{tot}	0.121182 a.u.	0.118899 a.u.
H_{corr}	0.122126 a.u.	0.119516 a.u.
G_{corr}	0.088062 a.u.	0.099305 a.u.
S_{tot}	71.694 calmol^{-1}K^{-1}	65.040 calmol^{-1}K^{-1}
$\varepsilon_0 + \varepsilon_{ZPE}$	–232.416065 a.u.	–232.416066 a.u.
$\varepsilon_0 + E_{tot}$	–232.411148 a.u.	–232.413431 a.u.
$\varepsilon_0 + H_{corr}$	–232.410204 a.u.	–232.412814 a.u.
$\varepsilon_0 + G_{corr}$	–232.444268 a.u.	–232.433025 a.u.

Lithiated Dihydroquinoline with Two THF Molecules (Figure 6i):

	Temperature	
	298 K	195 K
ε_0	–1452.45684804 a.u.	–1452.45684804 a.u.
ε_{ZPE}	0.589382 a.u.	0.589382 a.u.
E_{tot}	0.624030 a.u.	0.605960 a.u.
H_{corr}	0.624974 a.u.	0.606578 a.u.
G_{corr}	0.518606 a.u.	0.551585 a.u.
S_{tot}	223.871 calmol^{-1}K^{-1}	176.966 calmol^{-1}K^{-1}
$\varepsilon_0 + \varepsilon_{ZPE}$	–1451.867466 a.u.	–1451.867466 a.u.
$\varepsilon_0 + E_{tot}$	–1451.832818 a.u.	–1451.850888 a.u.
$\varepsilon_0 + H_{corr}$	–1451.831874 a.u.	–1451.850270 a.u.
$\varepsilon_0 + G_{corr}$	–1451.938242 a.u.	–1451.905263 a.u.

Lithiated Dihydroquinoline with Three THF Molecules (Figure 6ii):

	Temperature	
	298 K	195 K
ε_0	–1685.00264580 a.u.	–1685.00264580 a.u.
ε_{ZPE}	0.708028 a.u.	0.708028 a.u.
E_{tot}	0.748898 a.u.	0.727666 a.u.
H_{corr}	0.749842 a.u.	0.728283 a.u.
G_{corr}	0.630831 a.u.	0.667530 a.u.
S_{tot}	250.481 calmol^{-1}K^{-1}	195.503 calmol^{-1}K^{-1}
$\varepsilon_0 + \varepsilon_{ZPE}$	–1684.294617 a.u.	–1684.294618 a.u.
$\varepsilon_0 + E_{tot}$	–1684.253748 a.u.	–1684.274980 a.u.
$\varepsilon_0 + H_{corr}$	–1684.252804 a.u.	–1684.274363 a.u.
$\varepsilon_0 + G_{corr}$	–1684.371815 a.u.	–1684.335116 a.u.

Acetone:

	Temperature	
	298 K	195 K
ε_0	–193.227857154 a.u.	–193.227857154 a.u.
ε_{ZPE}	0.083224 a.u.	0.083224 a.u.
E_{tot}	0.088551 a.u.	0.086131 a.u.
H_{corr}	0.089495 a.u.	0.086749 a.u.
G_{corr}	0.055298 a.u.	0.066568 a.u.
S_{tot}	71.974 calmol^{-1}K^{-1}	64.940 calmol^{-1}K^{-1}
$\varepsilon_0 + \varepsilon_{ZPE}$	–193.144633 a.u.	–193.144633 a.u.
$\varepsilon_0 + E_{tot}$	–193.139306 a.u.	–193.141726 a.u.
$\varepsilon_0 + H_{corr}$	–193.138362 a.u.	–193.141108 a.u.
$\varepsilon_0 + G_{corr}$	–193.172559 a.u.	–193.161289 a.u.

Acetone Quenched Dihydroquinoline Above C-4 Position:

	Temperature	
	298 K	195 K
ε_0	–1878.26450462 a.u.	–1878.26450462 a.u.
ε_{ZPE}	0.798035 a.u.	0.798035 a.u.
E_{tot}	0.843561 a.u.	0.819751 a.u.
H_{corr}	0.844505 a.u.	0.820369 a.u.
G_{corr}	0.715954 a.u.	0.755420 a.u.
S_{tot}	270.560 calmol^{-1}K^{-1}	209.005 calmol^{-1}K^{-1}
$\varepsilon_0 + \varepsilon_{ZPE}$	–1877.466470 a.u.	–1877.466470 a.u.
$\varepsilon_0 + E_{tot}$	–1877.420943 a.u.	–1877.444754 a.u.
$\varepsilon_0 + H_{corr}$	–1877.419999 a.u.	–1877.444136 a.u.
$\varepsilon_0 + G_{corr}$	–1877.548551 a.u.	–1877.509085 a.u.

Acetone Quenched Dihydroquinoline Below C-4 Position (Figure 6iii):

	Temperature	
	298 K	195 K
ε_0	–1878.25709953 a.u.	–1878.25709953 a.u.
ε_{ZPE}	0.797466 a.u.	0.797466 a.u.
E_{tot}	0.843321 a.u.	0.819434 a.u.
H_{corr}	0.844265 a.u.	0.820051 a.u.
G_{corr}	0.714103 a.u.	0.754113 a.u.
S_{tot}	273.949 calmol^{-1}K^{-1}	212.187 calmol^{-1}K^{-1}
$\varepsilon_0 + \varepsilon_{ZPE}$	–1877.459634 a.u.	–1877.459634 a.u.
$\varepsilon_0 + E_{tot}$	–1877.413778 a.u.	–1877.437666 a.u.
$\varepsilon_0 + H_{corr}$	–1877.412834 a.u.	–1877.437049 a.u.
$\varepsilon_0 + G_{corr}$	–1877.542996 a.u.	–1877.502987 a.u.

Lithiated Dihydroquinoline with Three THF Molecules Above C2 Carbon of Dihydroquinoline:

	Temperature	
	298 K	195 K
ε_0	–1684.99681103 a.u.	–1684.99681103 a.u.
ε_{ZPE}	0.708334 a.u.	0.708334 a.u.
E_{tot}	0.749211 a.u.	0.728016 a.u.
H_{corr}	0.750156 a.u.	0.728633 a.u.
G_{corr}	0.629905 a.u.	0.667040 a.u.
S_{tot}	253.089 calmol^{-1}K^{-1}	198.205 calmol^{-1}K^{-1}
$\varepsilon_0 + \varepsilon_{ZPE}$	–1684.288477 a.u.	–1684.288477 a.u.
$\varepsilon_0 + E_{tot}$	–1684.247600 a.u.	–1684.268795 a.u.
$\varepsilon_0 + H_{corr}$	–1684.246655 a.u.	–1684.268178 a.u.
$\varepsilon_0 + G_{corr}$	–1684.366906 a.u.	–1684.329771 a.u.

Lithiated Dihydroquinoline with Two THF & One Acetone Molecules Above C2 Carbon of Dihydroquinoline:

	Temperature	
	298 K	195 K
ε_0	–1645.70220632 a.u.	–1645.70220632 a.u.
ε_{ZPE}	0.674980 a.u.	0.674980 a.u.
E_{tot}	0.716082 a.u.	0.694742 a.u.
H_{corr}	0.717026 a.u.	0.695359 a.u.
G_{corr}	0.598857 a.u.	0.635252 a.u.
S_{tot}	248.708 calmol^{-1}K^{-1}	193.426 calmol^{-1}K^{-1}
$\varepsilon_0 + \varepsilon_{ZPE}$	–1645.027227 a.u.	–1645.027226 a.u.
$\varepsilon_0 + E_{tot}$	–1644.986124 a.u.	–1645.007464 a.u.
$\varepsilon_0 + H_{corr}$	–1644.985180 a.u.	–1645.006847 a.u.
$\varepsilon_0 + G_{corr}$	–1645.103349 a.u.	–1645.066954 a.u.

Acetone Quenched Dihydroquinoline Above C-2 Position:

	Temperature	
	298 K	195 K
ε_0	–1645.68981787 a.u.	–1645.68981787 a.u.
ε_{ZPE}	0.678817 a.u.	0.678817 a.u.
E_{tot}	0.717842 a.u.	0.697138 a.u.
H_{corr}	0.718786 a.u.	0.697755 a.u.
G_{corr}	0.606028 a.u.	0.640670 a.u.
S_{tot}	237.318 calmol^{-1}K^{-1}	183.699 calmol^{-1}K^{-1}
$\varepsilon_0 + \varepsilon_{ZPE}$	–1645.011001 a.u.	–1645.011001 a.u.
$\varepsilon_0 + E_{tot}$	–1644.971976 a.u.	–1644.992680 a.u.
$\varepsilon_0 + H_{corr}$	–1644.971032 a.u.	–1644.992063 a.u.
$\varepsilon_0 + G_{corr}$	–1645.083789 a.u.	–1645.049148 a.u.
